# Effects of HiPIMS Duty Cycle on Plasma Discharge and the Properties of Cu Film

**DOI:** 10.3390/ma17102311

**Published:** 2024-05-13

**Authors:** Yongjie Ren, Heda Bai, Xincheng Liu, Jin Li, Xiangli Liu

**Affiliations:** 1 School of Materials Science and Engineering, Harbin Institute of Technology (Shenzhen), Shenzhen 518055, China; 22s155111@stu.hit.edu.cn (Y.R.); 20b955008@stu.hit.edu.cn (H.B.); 22s155058@stu.hit.edu.cn (X.L.); 2 Institute of Special Environments Physical Sciences, Harbin Institute of Technology (Shenzhen), Shenzhen 518055, China; jinli2019@hit.edu.cn

**Keywords:** Cu film, HiPIMS, plasma, duty cycle, grain size, electrical resistivity

## Abstract

In this paper, Cu thin films were deposited on Si (100) substrates by the high−power impulse magnetron sputtering (HiIPMS) technique, and the effects of different duty cycles (from 2.25% to 5.25%) on the plasma discharge characteristics, microstructure, and electrical properties of Cu thin films were investigated. The results of the target current test show that the peak target current remains stable under 2.25% and 3% duty cycle conditions. Under the conditions of a 4.5% and 5.25% duty cycle, the target peak current shows a decreasing trend. The average power of the target shows a rising trend with the increase in the duty cycle, while the peak power of the target shows a decreasing trend with the increase in the duty cycle. The results of OES show that with the increase in the duty cycle, the total peak intensity of copper and argon emissions shows an overall increasing trend. The duty cycle from 3% to 4.5% change in copper and argon emission peak total intensity change is not obvious. The deposition rate and surface morphology of the copper film were investigated by scanning electron microscopy, and the deposition rate of the copper film increased with the increase in the duty cycle, which was mainly due to the increase in the average power. The surface roughness of the copper film was evaluated by atomic force microscopy. X−ray diffraction (XRD) was used to analyze the grain size and texture of the Cu film, and the results showed that the average grain size of the Cu film increased from 38 nm to 59 nm on the (111) and (200) crystal planes. Four−probe square resistance test copper film resistivity in 2.25%, 3% low duty cycle conditions of the copper film resistivity is generally higher than 4.5%, 5.25% high duty cycle conditions, the copper film resistivity shows the trend of change is mainly affected by the copper film grain size and the (111) face of the double effect of the optimal orientation. The lowest resistivity of the copper film measured under the 4.5% duty cycle condition is 1.7005 μΩ·cm, which is close to the intrinsic resistivity of the copper film of 1.67 μΩ·cm.

## 1. Introduction

Due to its low resistivity, good thermal conductivity, strong oxidation resistance, thermal conductivity, and corrosion resistance [1,2], copper film has been widely used in the electronics industry and the new energy industry [3,4,5,6]. Especially in the field of ultra−large−scale integrated (ULSI) circuits, silicon wafers are often used as substrate materials [7], and copper thin films are used as interconnect materials due to their excellent properties [8]. In order to improve the conductivity of the surface of a silicon crystal, a thin film of copper needs to be deposited on its surface.

Many deposition techniques, such as physical vapor deposition [9,10,11], chemical vapor deposition [12,13,14], and electroplating [15,16,17], have been reported to be used to prepare copper thin films, among which magnetron sputtering technology has been widely used to prepare copper thin films due to its good process repeatability and easy industrialization [18,19]. Magnetron sputtering technology can be divided into direct current magnetron sputtering technology [10,20], radio−frequency magnetron sputtering technology [21,22] and high−power pulsed magnetron sputtering technology according to the different power supplies applied to the target. Compared with the first two, high−power pulsed magnetron sputtering technology can sputter target particles with a high ionization rate and control the yield of high−energy ions by adjusting process parameters to optimize the crystallinity, micromorphology, and properties of the film [23,24,25,26]. In addition, target particles with high ionization rates have certain diffraction properties, which are conducive to the deposition of uniform thin films on the surface of substrates with complex structures with high aspect ratios [27,28], such as the metallization of silicon−based semiconductor devices.

Several experiments have shown that Cu films with better grain crystallinity [29], larger grain size [30], and preferred crystal orientation (200) [31] usually have lower resistivity, which in turn improves the mean time to failure (MTTF) in the Cu damascene process [32]. Most of the physical vapor deposition techniques prepare copper films with too high resistivity, such as Choi [33] using radio frequency magnetron sputtering to prepare copper films with a minimum resistivity of 8 μΩ·cm and Gotoh [30] using ion beam assisted deposition to prepare copper films with a minimum resistivity of 3 Ω·cm, which are difficult to cope with the mean time to failure (MTTF) in the Cu damascene process. In HiPIMS, the energy of the ions impinging on the substrate can be easily controlled by duty cycle adjustment, which has been shown to increase the possibility of controlling the crystalline phase, microstructure, and chemical composition of the resulting films [34,35,36,37,38].

Hard coatings with excellent mechanical properties obtained by adjusting the duty cycle of the HiPIMS technique are widely studied in the scientific community [35,36,39], while the preparation of high−performance thin films on the surface of silicon−based semiconductors has rarely been investigated. Therefore, Cu films were prepared using the HiPIMS technique at different duty cycles in this work. The duty cycles were obtained by varying the pulse width while keeping the frequency constant. The effect of different duty cycles on the plasma discharge characteristics and the electrical conductivity of the Cu films was investigated.

## 2. Materials and Methods

### 2.1. Sample Preparation

**Figure 1:**
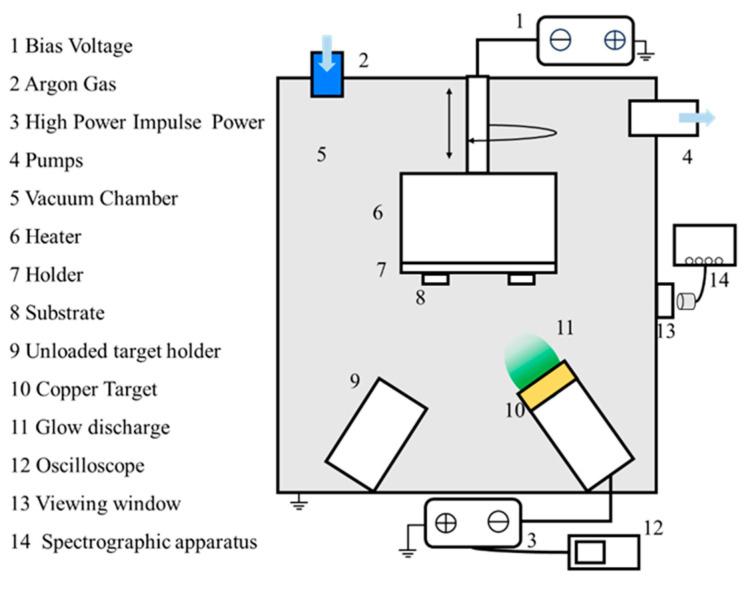
Schematic of the deposition apparatus.

All deposition was carried out in a magnetron sputter coater (DTS−200) with a rectangular vacuum chamber containing three magnetron cathodes, one of which contained a round cake copper target (99.99% purity, 2″ diameter). The layout of the specific coating equipment is shown in Figure 1. During normal operation of the coating equipment, the vacuum chamber is first pumped to below 10 Pa by a mechanical pump and then vacuumed to a background air pressure of 3 × 10^−3^ Pa by a vertical turbomolecular pump. A non−equilibrium magnetic field is applied above the copper target by a magnetron to bind the electrons above the target. A high−power pulsed power supply was applied to the copper target using a constant voltage mode power supply with a pulsed negative voltage of −500 V. The power supply was applied to the copper target with a constant−voltage mode power supply. In this research topic, the HiPIMS technology process parameters for pulse length range from 75 μs to 175 μs with a constant frequency of 300 Hz, corresponding to a duty cycle of 2.25% to 5.25% and a peak current of 20A to 9A. During the deposition process, the vacuum pressure was maintained at 0.5 Pa. The argon (Ar, 99.999%) gas flow was maintained at 100 sccm, and its flux was controlled by a mass flow controller. Copper films were deposited by HiPIMS with duty cycles of 2.25%, 3%, 4.5%, and 5.25% (pulse lengths of 75 μs, 100 μs, 150 μs, and 175 μs, respectively), corresponding to Sample 1, Sample 2, Sample 3, and Sample 4.

Copper thin films were deposited on 20 mm × 20 mm × 1 mm silicon wafers ((100) crystal−oriented P−type doped with a resistivity less than 0.0015 Ω·cm) substrates. Prior to each deposition, a negative bias was applied to the substrate for sputter cleaning. The substrates were mounted on a sample holder with a target distance of 75 mm from the substrate. The heating of all substrates was maintained at 50 °C during the experiments, and no DC bias voltage was applied to all substrates. The deposition parameters involved in the specific experiments are shown in Table 1.

### 2.2. Characterization

During the deposition process, in order to understand the real−time discharge voltage and discharge current of the copper target, a digital oscilloscope (TBS100X) was connected to the HiPIMS power supply box to collect the voltage and current signals. Four−channel plasma emission spectroscopy was used to determine the plasma species and intensity in the chamber at different duty cycles, especially the emission spectra of sputtered particles from the copper target (Cu). Spectroscopy is the measurement of the intensity of the emission spectrum from 200 nm to 1000 nm, in which the spectrometer probe is oriented directly above the target through a viewing window with a highly transmissive film.

The morphology and thickness of copper films deposited on silicon wafers were evaluated using an environmental scanning electron microscope (Gemini SEM360), where the thickness of the deposited copper film is controlled between 570 nm and 1560 nm. The copper film thickness was obtained by SEM by taking the arithmetic mean of multiple measurements on the sample cross−section. The three−dimensional micro−morphology and roughness of the copper thin film surfaces were characterized by atomic force microscopy (AFM, Bruker−ICON) in probe tapping mode, where the values of roughness were obtained by taking multiple 1 μm × 1 μm areas in the range of 5 μm × 5 μm, and then the arithmetic average of the multiple sets of roughness values was taken.

The crystal structure was analyzed by an X−ray diffractometer (Rigaku SmartLab (9 KW)) in grazing incidence mode (GI−XRD). X−ray diffractograms of copper thin films were acquired using a Cu Kα source (Kγ = 1.5406 Å) at an angle of 30° to 80° with the following measurement parameters: step = 0.02°, speed = 4 °/min, incident slit = 0.5 mm.

The square resistance of copper films was evaluated using a four−probe tester (RTS−9), and the film resistivity was calculated in conjunction with the measured thickness. The square resistance values were obtained by taking multiple measurements along the diagonal on a square film sample and then taking the arithmetic mean.

## 3. Results and Discussion

### 3.1. HiPIMS Deposited Copper Film Features

**Figure 2:**
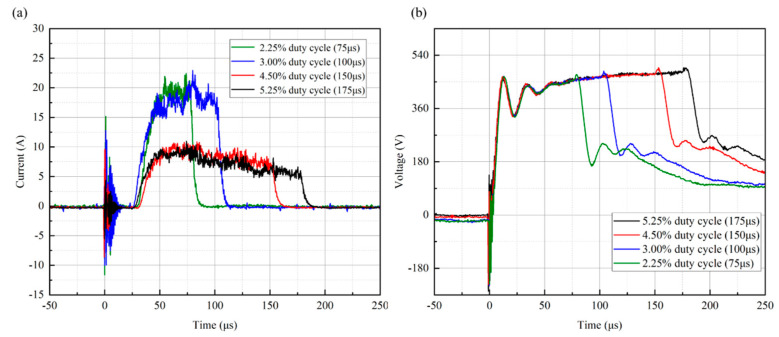
Voltage and discharge current waveforms of copper film targets deposited by HiPIMS under different duty cycles: (**a**) target current and (**b**) target voltage.

The voltage and discharge current waveforms of copper thin film targets deposited by HiPIMS technology under different duty cycle conditions (ranging from 2.25% to 5.25%) are shown in Figure 2. Current and voltage curves with different shapes for different duty cycle conditions can be found, which are mainly determined by the nature of the target glow discharge and the size of the capacitor in the high−power pulsed supply [39]. As shown in Figure 2a, after applying voltage to the target, the target current cannot be generated immediately for all samples, but there is a discharge reaction time of 25 μs. Anders et al. [40] also had a 20 μs delay in the target current when preparing copper films using the HiPIMS technique. This is mainly due to the time required for the ionization of argon atoms and the sputtering of target particles. The peak current shows an overall decreasing trend with increasing duty cycles, where the peak current decreases especially significantly (by about 1/2) when the duty cycle is increased from 3% to 4.5%. Under the conditions of 2.25% and 3% low duty cycles, the target peak discharge current remained at a stable value of 20 A. Under the conditions of 4.25% and 5.25% high duty cycles, the target peak discharge current showed a decreasing trend, i.e., it gradually decreased from the beginning of 9 A to 5. The trend of the variation between peak current and duty cycle is consistent with previous studies [35,41]. As shown in Figure 2b, the peak discharge voltage is less affected by the duty cycle.

The average power and peak power of the target are useful parameters for evaluating the efficiency of the magnetron sputtering deposition process, and the formulas for calculating the average power *(P_Av_*) and peak power *(P_Pk_*) under HiPIMS operation are shown in (1), (2), respectively:

(1) $$P_{Av}=\frac{1}{T}\int_{o}^{\tau }V_{T(t)}\cdot \;I_{T\left(t\right)}\cdot \;dt$$

(2) $$P_{Pk}=U_{Pk}\cdot \;I_{Pk}$$

**Figure 3:**
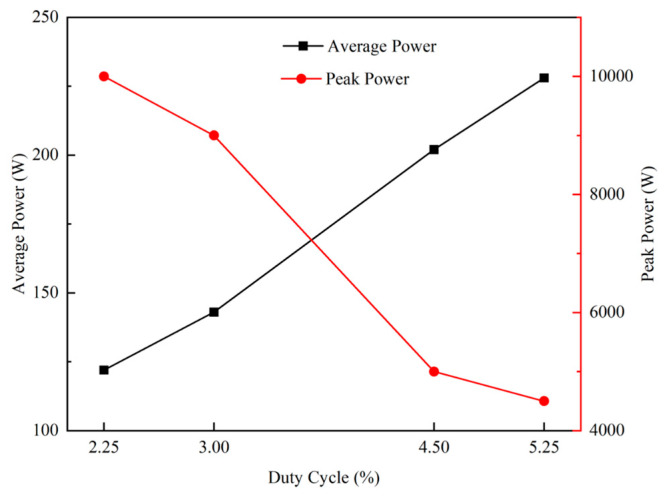
Variation in average power and peak power of the target with different duty cycles of HiPIMS.

In the above equation, *T* is the period of a pulse, *T* = 1/*f, f* is the frequency at which the pulse is repeated, *τ* is the pulse duration during the application of the voltage, *I_T_* is the target discharge current, and *V_T_* is the target voltage, which can be read from the power supply box. In the present study, HiPIMS was operated at a frequency of 300 Hz, so *T* = 3333 μs. *U_Pk_* is the peak target voltage, and *I_Pk_* is the peak target current. Therefore, the average power of the target and the peak power of the target versus duty cycle during the deposition of copper thin films by HiPIMS can be obtained, as shown in Figure 3. As the duty cycle increases (from 2.25% to 5.25%), the average power of the target increases, while the peak power of the target decreases continuously.

**Figure 4:**
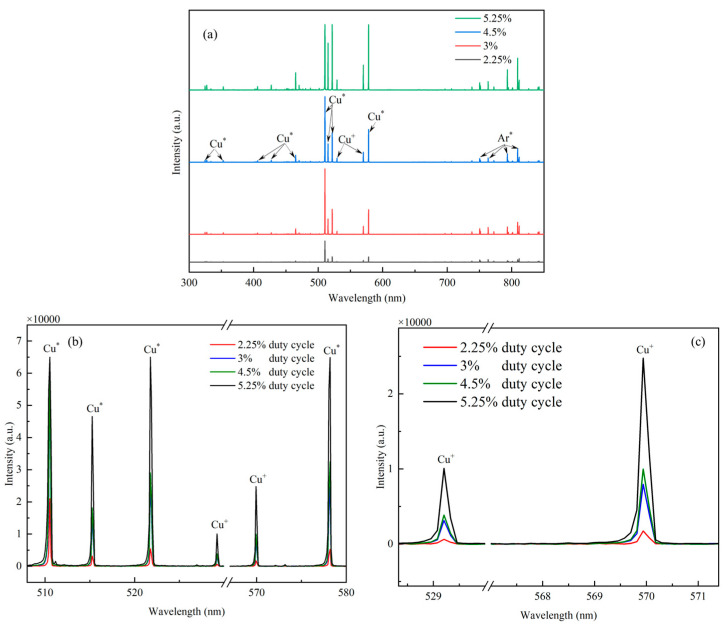
Four−channel plasma emission spectra under different duty cycle conditions: (**a**) full−band spectra from 200 nm to 1000 nm; (**b**) specific spectra from 307 nm to 580 nm; (**c**) specific spectra from 528 nm to 580 nm.

Four-channel plasma emission spectra were recorded in order to obtain information about the plasma species in HiPIMS discharges, especially sputtered copper ions and their peak emission intensity. Figure 4 shows the emission spectra of HiPIMS at different duty cycles, and the four-channel plasma emission spectra were collected during the application of pulsed voltage to HiPIMS. The calibration of the spectral emission of copper atoms and copper ions in Figure 4 was performed using the PLASUS SpecLine Database, which can be found in the Supplementary Material. As shown in Figure 4a, the emission spectra can be divided into two parts according to the type of plasma: the first part has a wavelength range of 600 nm to 850 nm and is dominated by strong argon (Ar) emission lines; the second part has a wavelength range of 300 nm to 600 nm and is dominated by copper (Cu) emission lines. From Figure 4a, it can be seen that the peak intensity of light emission from HiPIMS will be higher in high duty cycles than in low duty cycles. This is mainly due to the fact that a high duty cycle increases the glow discharge time per unit cycle of the copper target. In order to see more intuitively the condition of copper atoms and copper ions sputtered from the copper target under different duty cycle conditions, all the emission spectra of copper particles located between 505 nm and 580 nm are put in Figure 4b. In the wavelength spectral range from 505 nm to 580 nm, six particle optical emission lines can be observed, which come from excited copper atoms located at 511 nm (Cu|, neutral particles of copper atoms), 515 nm (Cu|, neutral particles of copper atoms), 522 nm (Cu|, neutral particles of copper atoms), 529 nm (Cu|, neutral particles of copper atoms), 570 nm (Cu||, monovalent ions of copper atoms), and 578 nm (Cu|, neutral particles of copper atoms). It can be observed that the overall intensity of the six emission spectral lines increases more significantly when the duty cycle is varied from 2.25% to 3%. It can be observed that the overall intensity of the six emission spectral lines increases more significantly when the duty cycle is varied from 2.25% to 3% and from 4.5% to 5.25%, while it varies very little when the duty cycle is varied from 3% to 4.5%. From 4.5% to 5.25%, while it varies very little when the duty cycle is varied from 3% to 4.5%. It can be seen in Figure 4c that the intensity of the copper ion emission spectrum increases with increasing duty cycle (from 2.25% to 5.25%), which indicates an increase in energetic copper ions sputtered from the target. Ganesan et al. [41] also found that the HiPIMS technique at a high duty cycle favours the increase in the plasma intensity in the cavity, which leads to an overall increase in the intensity of light emission from the particles in the cavity. This is mainly due to the fact that the increase in duty cycle is conducive to prolonging the discharge time of the HiPIMS technique in a single pulse, which results in more copper particles being sputtered in a single pulse, increasing the density of the plasma in the cavity.

### 3.2. Microstructure and Morphology

**Figure 5:**
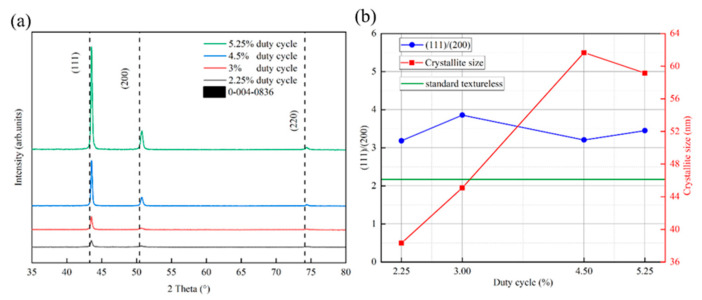
X−ray diffractograms, grain size, and peak intensity ratio of (111)/(200) under different duty cycle conditions: (**a**) X−ray diffractograms under different duty cycle conditions, and (**b**) grain size and peak intensity ratio of (111)/(200) versus duty cycle.

Figure 5 shows the XRD diffractograms of all the samples in the region of 30–80°, the grain size, and the peak intensity ratio of (111)/(200). As shown in Figure 5a, the diffraction peak intensities of each crystalline surface of the copper films (111), (200), and (220) increase with the increase in duty cycle, which is mainly due to the bombardment of the substrate surface by the target particles with high energies to improve the atomic mobility of the surface of the films, thus increasing the crystallinity. This is consistent with the result that the spectral emission intensity of target particles increases with increasing duty cycle in Figure 4a. A common phenomenon for all samples is that the peak position is shifted to a higher diffraction angle with respect to the reference peak position (vertical dashed position in Figure 5a), which was taken from JCPDS card number 0−004−0836. This may indicate the presence of tensile stresses in all the deposited films, and these results are in agreement with the previous results for copper films deposited using HiPIMS [42,43,44]. The tensile nature of the Cu films on silicon substrates may be due to the large difference in the coefficient of thermal expansion of Cu (17 × 10^−6^ K^−1^) compared to silicon (2.7 × 10^−6^ K^−1^). Compressive stress is commonly used in films grown using energetic ion bombardment due to densification of the film by adsorbed atom insertion at grain boundaries and atom blasting [45]. There may be competition between these two mechanisms, which leads to the final stress state of the film. Among all the crystalline surfaces of the copper film, the copper (111) crystalline surface has the highest diffraction peak intensity, which is mainly due to the fact that the densely arranged (111) crystalline surface has a relatively low surface free energy [46]. The grain sizes of the samples were calculated from the Scherrer equation, as shown in Figure 5b. The grain size of the copper film shows an elevated and then decreasing trend in relation to the duty cycle. The rising trend is mainly due to the increase in ion bombardment energy, which enhances atomic mobility and surface diffusion [32]. The decreasing trend may be due to the resputtering effect triggered by the target particles with too much energy, which removes the larger grains that have already crystallized. Figure 5b shows the relationship between the ratio of the integral intensities of the (111) and (200) diffracted lattice planes and the duty cycle. It is found that the integrated intensity ratio ((111)/(200)) of HiPIMS−deposited copper films is higher than 2.17, which is the value for standard untextured samples (JCPDS file 0−004−0836), regardless of the variation in the duty cycle (from 2.25% to 5.25%). This means that the volume fraction of (111)−oriented microcrystals is much larger than that of (200)−oriented microcrystals, resulting in a strong (111) texture.

**Figure 6:**
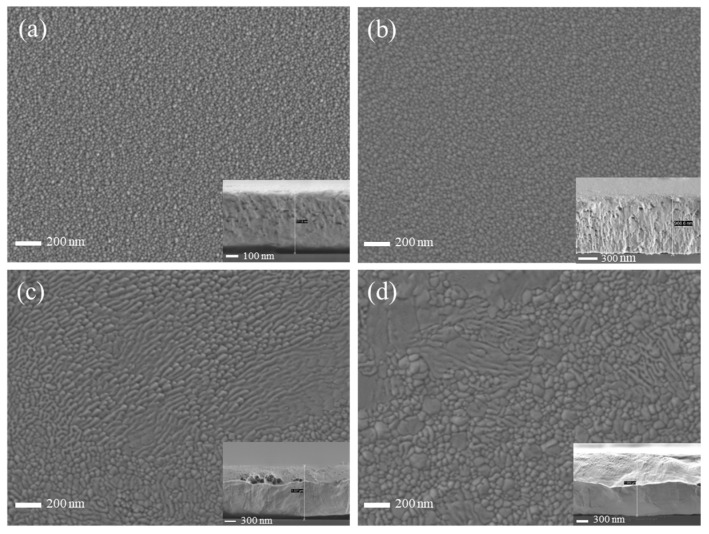
SEM surface morphology and cross−section of copper thin films under different duty cycle conditions: (**a**) 2.25% duty cycle; (**b**) 3% duty cycle; (**c**) 4.5% duty cycle; (**d**) 5.25% duty cycle.

Figure 6 shows the SEM images of copper films deposited by HiPIMS under different duty cycle conditions, and it is found that the particle size on the surface of the copper film shows an increasing trend with increasing duty cycle. This is mainly due to the high−energy target particles bombarding the surface of the copper film, which enhances atomic mobility and surface diffusion. This finding is consistent with the effect of different duty cycles of HiPIMS on the microstructure of AlCrN coatings studied by Liu et al. [35]. This also aids in verifying the general trend of the grain size variation with the duty cycle in Figure 5b. In particular, at duty cycles of 4.5% and 5.25%, the particles on the film surface are fully diffused and coarsened, and a large number of coarse particles are interspersed with each other, resulting in the unevenness of the film surface. Observation of the cross−section of the copper film under different duty cycle conditions reveals that the cross−section micro−morphology of the copper film is covered due to plastic deformation. The greater the thickness of the copper film, the more severe the plastic deformation. This is mainly due to the good plasticity of the copper film when the SEM cross−section was taken to prepare the sample.

**Figure 7:**
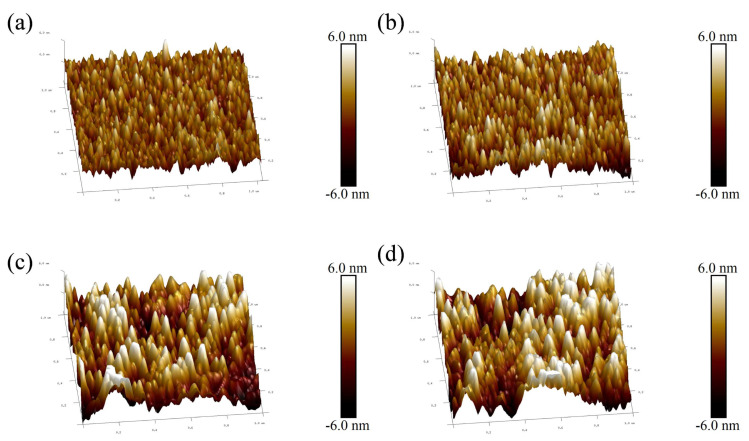
Surface morphology of AFM copper film under different duty cycle conditions: (**a**) 2.25% duty cycle; (**b**) 3% duty cycle; (**c**) 4.5% duty cycle; (**d**) 5.25% duty cycle.

**Figure 8:**
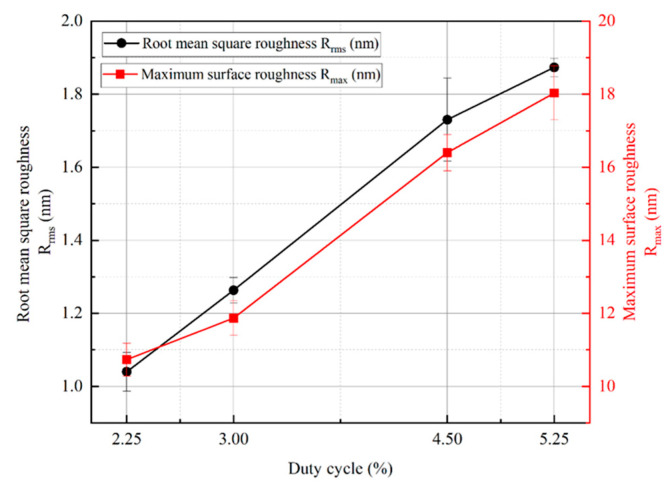
Variation in root mean square roughness, maximum roughness, and duty cycle of the copper film.

In order to obtain further information about the surface roughness of copper films, Figure 7 shows the surface roughness of copper films measured by AFM under different duty cycle conditions. As the duty cycle increases, the surface of the copper film becomes rough. The surface roughness values increased from Rrms 1.04 nm and Rmax 10.7 nm at a duty cycle of 2.25% to Ra 1.87 nm and Rmax 18 nm at a duty cycle of 5.25%, respectively. The trend of copper film surface roughness is mainly due to the growth of copper film surface particles, while the increase and coarsening of particles on the surface of the copper film can be well reflected in Figure 6. In order to visualize the relationship between the surface roughness of the copper film and the duty cycle, Figure 8 shows the trend graphs of the sample surface root mean square roughness and surface maximum roughness with different duty cycles, respectively. The deeper reason for the increase in copper film roughness with increasing duty cycle is mainly due to the increase in the overall energy of the sputtered particles, which further exacerbates the formation of three−dimensional islands during the Volmer–Weber growth mode [26,47].

### 3.3. Deposition Rate and Resistivity Properties

**Figure 9:**
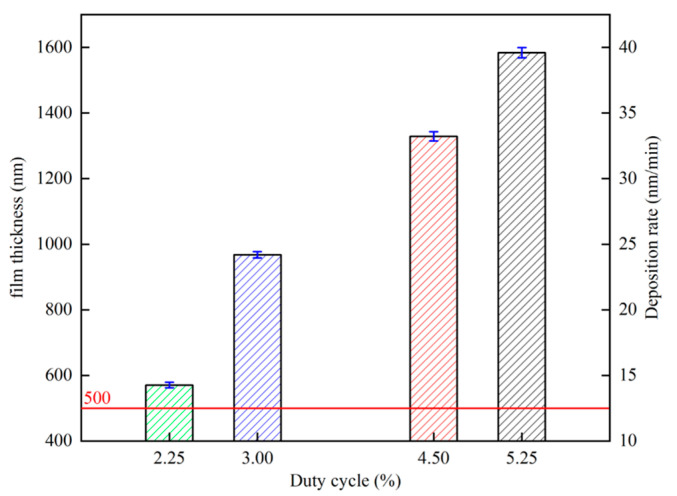
Variation in copper film thickness and deposition rate versus duty cycle.

Figure 9 shows the relationship between film thickness, deposition rate, and duty cycle, respectively, and the deposition rate rises with the increase in duty cycle. This is mainly due to the increase in the duty cycle caused by the increase in the average power of the target, which is the main factor affecting the deposition rate of HiPIMS coating [48]. With the increase in duty cycle, the film thickness increases, and the film thickness increases from 572 nm at 2.25% to 1596 nm at 5.25%. In the study of Cemin et al. [49], it was found that the thickness of the copper film is below 500 nm; the thickness is the main factor affecting its resistivity. When the copper film thickness is above 500 nm, the effect of thickness on the resistivity of the copper film is negligible. Therefore, the film thickness of all samples in this experiment was kept above 500 nm.

**Figure 10:**
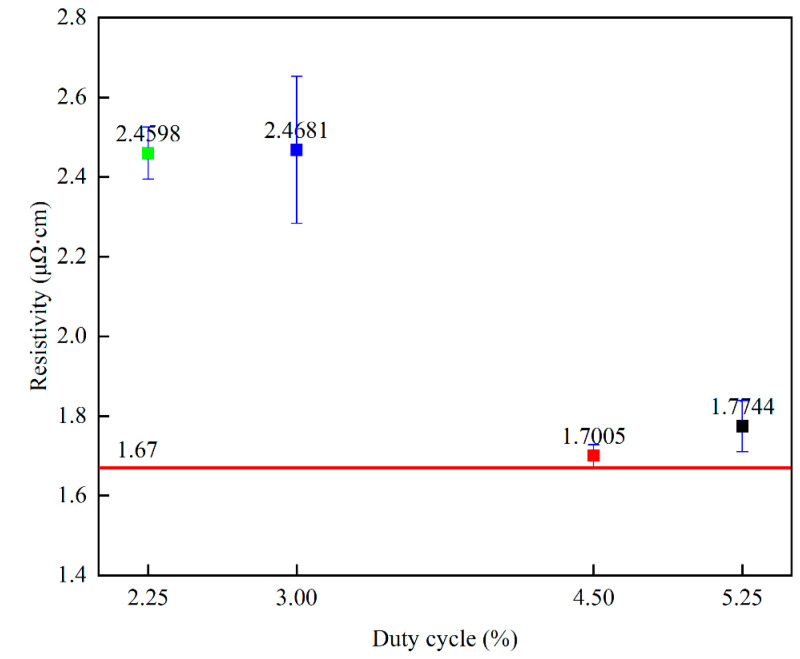
Variation in copper film resistivity versus duty cycle.

Figure 10 shows the relationship between resistivity and different duty cycles, with resistivity showing a decreasing trend as the duty cycle varied from 2.25% to 4.5%. Table 2 lists the values of the specific resistivity of copper films prepared by the HiPIMS technique with different duty cycles. The resistivity shows an increasing trend as the duty cycle varies from 4.5% to 5.25%. Previous experiments have shown that higher crystallinity, larger grain size [30], smaller (111) crystal plane weave [31], and higher film thickness of copper films are beneficial in reducing the resistivity of copper films [49]. Since the copper film thickness is greater than 500 nm, the first three influences are mainly considered in this experiment. During the increase in duty cycle from 2.25% to 4.5%, the crystallinity and crystal size of the copper film show an increasing trend, which can explain the decreasing trend of copper film resistivity. While the duty cycle increases from 4.5% to 5.25%, the crystal size of the copper film shows a decreasing trend, and the (111)/(200) crystal surface shows an increasing trend, which may be the reason for the increase in the resistivity of the copper film. The lowest copper film resistivity measured under the 4.5% duty cycle is 1.7005 μΩ·cm, which is very close to the intrinsic resistivity of the copper film of 1.67 μΩ·cm. Table 3 lists the resistivity of copper films deposited by different preparation methods (all copper film thicknesses are above 500 nm). It can be found that the resistivity of the copper films prepared in this experiment is much lower than that of those prepared by other methods, which is of great significance in the field of silicon−based semiconductors.

## 4. Conclusions

In this study, the preparation of copper films on silicon wafers by HiPIMS at different duty cycles (2.25% to 5.25%) has been explored. The detection of target voltage and current signals by a digital oscilloscope shows that the peak target current remains stable at 2.25% and 3% duty cycle conditions. Under the conditions of 4.5% and 5.25% duty cycles, the peak target current shows a decreasing trend. The average power of the target during HiPIMS deposition increases with the increase in the duty cycle, and the peak power of the target decreases with the increase in the duty cycle. The results of the OES show that with the increase in the duty cycle, the total intensity of the peak emission of copper and argon shows an overall increasing trend. The duty cycle changes from 3% to 4.5%, and the total intensity of copper and argon emission peaks does not change significantly.

X−ray diffractograms show that the Cu films deposited on the wafers are polycrystalline, and there are tensile stresses in the Cu films at duty cycles from 2.25% to 5.25%. The crystal size shows an increasing and then decreasing trend with an increasing duty cycle (2.25% to 5.25%). The relationship between the variation in the ratio of the integrated intensity of the diffraction peaks of the (111)/(200) facets in the Cu film and the duty cycle is not significant, but there is a selective orientation of the (111) facets in all the samples. Scanning electron microscopy showed that the thin copper facet particles were gradually coarsened by increasing the duty cycle from 2.25% to 5.25%. Atomic force microscopy results showed that the surface roughness value of copper film increased from Ra 1.04 nm at 2.25% to Ra 1.87 nm at 5.25% with an increasing duty cycle. The copper film deposition rate showed an increasing trend with an increasing duty cycle (2.25% to 5.25%). The four−probe square resistance test shows that the resistivity of the copper films at 2.25% and 3% low duty cycle conditions is generally higher than that at 4.5% and 5.25% high duty cycle conditions. This is mainly due to the dual influence of the grain size of the copper film and the (111) optimal orientation of the crystalline surfaces. In summary, the use of HiPIMS technology for the preparation of copper films at a duty cycle of 4.5% can take into account both the faster deposition rate (33 nm/min) and the preparation of highly conductive copper films (resistivity is 1.7005 μΩ·cm), which can provide a reference for the preparation of high−performance copper films for silicon−based semiconductors.

## Figures and Tables

**Table 1:** Details and parameters of sputtering.

Parameter	HiPIMS (Sample 1/2/3/4)
Target to substrate distance (mm)	75
Substrate temperature (°C)	50
Working pressure (Pa)	0.5
Substrate rotation speed (r/min)	10
Total deposition time (min)	40
pulse frequency (Hz)	300
Duty cycle (%)	2.25/3/4.5/5.25
Pulse−negative voltage (V)	500
Peak discharge current (A)	20/18/10/9

**Table 2:** Copper film resistivity under different duty cycle conditions.

Duty cycle (%)	2.25	3.00	4.50	5.25
Resistivity (μΩ·cm)	2.4598	2.4681	1.7005	1.7744
Intrinsic resistivity (μΩ·cm)	1.67	1.67	1.67	1.67

**Table 3:** Lowest copper film resistivity was obtained from different preparation methods.

Thin Film Materials	Preparation Method	Resistivity (μΩ·cm)	Bibliographic Reference
Copper film	HiPIMS	1.7005	present study
	RF magnetron sputtering	8	[33]
	IBAD	3	[30]
	DC magnetron sputtering	2.1	[31]
	HiPIMS	2.3	[32]

## Data Availability

The data in this study are available upon request.

## Supplementary Materials

The following supporting information can be downloaded at: https://www.mdpi.com/article/10.3390/ma17102311/s1, PLASUS SpecLine Database.

## References

[1] Ghosh S. (2019). Electroless Copper Deposition: A Critical Review. Thin Solid Films.

[2] Beaucarne G., Schubert G., Hoornstra J., Horzel J., Glunz S.W. (2012). Summary of the Third Workshop on Metallization for Crystalline Silicon Solar Cells. Energy Procedia.

[3] Yin S., Zhu W., Deng Y., Peng Y., Shen S., Tu Y. (2017). Enhanced Electrical Conductivity and Reliability for Flexible Copper Thin-Film Electrode by Introducing Aluminum Buffer Layer. Mater. Des..

[4] Gu C.D., You Y.H., Wang X.L., Tu J.P. (2012). Electrodeposition, Structural, and Corrosion Properties of Cu Films from a Stable Deep Eutectics System with Additive of Ethylene Diamine. Surf. Coat. Technol..

[5] Highly Conductive Copper Nano/Microparticles Ink via Flash Light Sintering for Printed Electronics-IOPscience. https://iopscience.iop.org/article/10.1088/0957-4484/25/26/265601.

[6] Luo L.-M., Lu Z.-L., Huang X.-M., Tan X.-Y., Ding X.-Y., Cheng J.-G., Zhu L., Wu Y.-C. (2014). Electroless Copper Plating on PC Engineering Plastic with a Novel Palladium-Free Surface Activation Process. Surf. Coat. Technol..

[7] Yoon S., Bang J., Lee H.-D., Oh J. (2016). Interfacial AlN Formation of Si/Ti/Al/Cu Ohmic Contact for AlGaN/GaN High-Electron-Mobility Transistors. Microelectron. Eng..

[8] Yu M., Zhang J., Li D., Meng Q., Li W. (2006). Internal Stress and Adhesion of Cu Film/Si Prepared by Both MEVVA and IBAD. Surf. Coat. Technol..

[9] A preliminary study on brazing properties of Ti/Ti and Ti/steel joints by copper film deposited by PVD process |Journal of Materials Engineering and Properties. https://link.springer.com/article/10.1007/s11665-012-0121-7.

[10] Mukherjee S.K., Joshi L., Barhai P.K. (2011). A Comparative Study of Nanocrystalline Cu Film Deposited Using Anodic Vacuum Arc and Dc Magnetron Sputtering. Surf. Coat. Technol..

[11] Wang B., Eberhardt W., Kück H. (2005). Adhesion of PVD Layers on Liquid Crystal Polymer Pretreated by Oxygen-Containing Plasma. Vacuum.

[12] Lin C.-L., Chen P.-S., Lin Y.-C., Tsui B.-Y., Chen M.-C. (2003). Via-Filling Capability of Copper Film by CVD. J. Electrochem. Soc..

[13] Borgharkar N.S., Griffin G.L., James A., Maverick A.W. (1998). Alcohol-Assisted Growth of Copper CVD Films. Thin Solid Films.

[14] Gandikota S., Voss S., Tao R., Duboust A., Cong D., Chen L.-Y., Ramaswami S., Carl D. (2000). Adhesion Studies of CVD Copper Metallization. Microelectron. Eng..

[15] Zhang P., Zhang L., Qu X. (2023). Anomalous Vertical Twins with High (2 2 0) Texture in Direct Current Electroplating Copper Film. Appl. Surf. Sci..

[16] Copper Electroplating for Future Ultralarge Scale Integration Interconnection|Journal of Vacuum Science & Technology A|AIP Publishing. https://pubs.aip.org/avs/jva/article/18/2/656/1067553/Copper-electroplating-for-future-ultralarge-scale.

[17] Miura S., Honma H. (2003). Advanced Copper Electroplating for Application of Electronics. Surf. Coat. Technol..

[18] Kelly P.J., Arnell R.D. (2000). Magnetron Sputtering: A Review of Recent Developments and Applications. Vacuum.

[19] Arnell R.D., Kelly P.J. (1999). Recent Advances in Magnetron Sputtering. Surf. Coat. Technol..

[20] Velicu I.-L., Tiron V., Rusu B.-G., Popa G. (2017). Copper Thin Films Deposited under Different Power Delivery Modes and Magnetron Configurations: A Comparative Study. Surf. Coat. Technol..

[21] Katayama T., Toyota H. (2020). RF Power Dependence on Structural and Electrical Properties of Cu Thin Films on a Glass Substrate Prepared Using Magnetron Sputtering with Multipolar Magnetic Plasma Confinement. IEEJ Trans. Sens. Micromachines.

[22] Saremi M., Yeganeh M. (2010). Investigation of Corrosion Behaviour of Nanostructured Copper Thin Film Produced by Radio Frequency Sputtering. Micro Nano Lett..

[23] Anders A. (2017). Tutorial: Reactive High Power Impulse Magnetron Sputtering (R-HiPIMS). J. Appl. Phys..

[24] Solovyev A.A., Semenov V.A., Oskirko V.O., Oskomov K.V., Zakharov A.N., Rabotkin S.V. (2017). Properties of Ultra-Thin Cu Films Grown by High Power Pulsed Magnetron Sputtering. Thin Solid Films.

[25] Insights into the Copper HiPIMS Discharge: Deposition Rate and Ionised Flux Fraction-IOPscience. https://iopscience.iop.org/article/10.1088/1361-6595/ad10ef/meta.

[26] Cemin F., Abadias G., Minea T., Furgeaud C., Brisset F., Solas D., Lundin D. (2017). Benefits of Energetic Ion Bombardment for Tailoring Stress and Microstructural Evolution during Growth of Cu Thin Films. Acta Mater..

[27] Alami J., Persson P.O.Å., Music D., Gudmundsson J.T., Bohlmark J., Helmersson U. (2005). Ion-Assisted Physical Vapor Deposition for Enhanced Film Properties on Nonflat Surfaces. J. Vac. Sci. Technol. A.

[28] Shimizu T., Komiya H., Watanabe T., Teranishi Y., Nagasaka H., Morikawa K., Yang M. (2014). HIPIMS Deposition of TiAlN Films on Inner Wall of Micro-Dies and Its Applicability in Micro-Sheet Metal Forming. Surf. Coat. Technol..

[29] Furuya R., Suzuki K., Miura H. Evaluation of the Crystallographic Quality of Electroplated Copper Thin-Film Interconnections Embedded in TSV Structures. Proceedings of the 2012 14th International Conference on Electronic Materials and Packaging (EMAP).

[30] Gotoh Y., Yoshii H., Amioka T., Kameyama K., Tsuji H., Ishikawa J. (1996). Structures and Properties of Copper Thin Films Prepared by Ion Beam Assisted Deposition. Thin Solid Films.

[31] Burnett A.F., Cech J.M. (1993). Relationship of Crystallographic Orientation and Impurities to Stress, Resistivity, and Morphology for Sputtered Copper Films. J. Vac. Sci. Technol. A Vac. Surf. Film..

[32] Wu B.H., Wu J., Jiang F., Ma D.L., Chen C.Z., Sun H., Leng Y.X., Huang N. (2017). Plasma Characteristics and Properties of Cu Films Prepared by High Power Pulsed Magnetron Sputtering. Vacuum.

[33] Choi H.M., Choi S.K., Anderson O., Bange K. (2000). Influence of Film Density on Residual Stress and Resistivity for Cu Thin Films Deposited by Bias Sputtering. Thin Solid Films.

[34] Tang J.-F., Huang S.-Y., Chen I.-H., Shen G.-L., Chang C.-L. (2023). Effects of Synchronous Bias Mode and Duty Cycle on Microstructure and Mechanical Properties of AlTiN Coatings Deposited via HiPIMS. Coatings.

[35] Liu Y., Ding J.C., Zhang B.R., Chen J.J., Tang C.R., Zhu R.Y., Zheng J. (2022). Effect of Duty Cycle on Microstructure and Mechanical Properties of AlCrN Coatings Deposited by HiPIMS. Vacuum.

[36] Chang C.-L., Luo G.-J., Yang F.-C., Tang J.-F. (2021). Effects of Duty Cycle on Microstructure of TiN Coatings Prepared Using CAE/HiPIMS. Vacuum.

[37] Evolution of Target Condition in Reactive HiPIMS as a Function of Duty Cycle: An Opportunity for Refractive Index Grading|Journal of Applied Physics|AIP Publishing. https://pubs.aip.org/aip/jap/article/121/17/171909/946383.

[38] Cedeño-Vente M.L., Mondragón-Rodríguez G.C., Camacho N., Gómez-Ovalle A.E., Gonzalez-Carmona J.M., Alvarado-Orozco J.M., Espinosa-Arbelaez D.G. (2021). Tailoring the Chemical Composition and Microstructure of CrxN Deposited by HiPIMS through Duty-Cycle Modifications. Surf. Coat. Technol..

[39] Wang L., Jin J., Zhu C., Li G., Kuang X., Huang K. (2019). Effects of HiPIMS Pulse-Length on Plasma Discharge and on the Properties of WC-DLC Coatings. Appl. Surf. Sci..

[40] Anders A., Andersson J., Ehiasarian A. (2007). High Power Impulse Magnetron Sputtering: Current-Voltage-Time Characteristics Indicate the Onset of Sustained Self-Sputtering. J. Appl. Phys..

[41] Ganesan R., Treverrow B., Murdoch B., Xie D., Ross A.E., Partridge J.G., Falconer I.S., McCulloch D.G., McKenzie D.R., Bilek M.M.M. (2016). Duty Cycle Control in Reactive High-Power Impulse Magnetron Sputtering of Hafnium and Niobium. J. Phys. D Appl. Phys..

[42] Wu B., Haehnlein I., Shchelkanov I., McLain J., Patel D., Uhlig J., Jurczyk B., Leng Y., Ruzic D.N. (2018). Cu Films Prepared by Bipolar Pulsed High Power Impulse Magnetron Sputtering. Vacuum.

[43] Viloan R.P.B., Helmersson U., Lundin D. (2021). Copper Thin Films Deposited Using Different Ion Acceleration Strategies in HiPIMS. Surf. Coat. Technol..

[44] Velicu I.-L., Ianoş G.-T., Porosnicu C., Mihăilă I., Burducea I., Velea A., Cristea D., Munteanu D., Tiron V. (2019). Energy-Enhanced Deposition of Copper Thin Films by Bipolar High Power Impulse Magnetron Sputtering. Surf. Coat. Technol..

[45] Davis C.A. (1993). A Simple Model for the Formation of Compressive Stress in Thin Films by Ion Bombardment. Thin Solid Films.

[46] Thompson C.V. (1988). Coarsening of Particles on a Planar Substrate: Interface Energy Anisotropy and Application to Grain Growth in Thin Films. Acta Metall..

[47] Suliali N.J., Goosen W.E., Janse van Vuuren A., Olivier E.J., Bakhit B., Högberg H., Darakchieva V., Botha J.R. (2022). Ti Thin Films Deposited by High-Power Impulse Magnetron Sputtering in an Industrial System: Process Parameters for a Low Surface Roughness. Vacuum.

[48] Tiron V., Velicu I.-L., Vasilovici O., Popa G. (2015). Optimization of Deposition Rate in HiPIMS by Controlling the Peak Target Current. J. Phys. D Appl. Phys..

[49] Low Electrical Resistivity in Thin and Ultrathin Copper Layers Grown by High Power Impulse Magnetron Sputtering|Journal of Vacuum Science & Technology A|AIP Publishing. https://pubs.aip.org/avs/jva/article/34/5/051506/245492.

