# Supplementary material for: Effects of HiPIMS Duty Cycle on Plasma Discharge and the Properties of Cu Film
Source: Materials (Basel). 2024 May 13;17(10):2311. doi: 10.3390/ma17102311 (PMC11123023; doi:10.3390/ma17102311)
Supplement: Supplementary file 1 [file materials-17-02311-s001.zip › materials-2993218-supplementary.pdf]

PLASUS SpecLine Database

Lower wavelength limit: 100 nm
Upper wavelength limit: 1000 nm

Lines found:

Line [nm]	Element	I (rel.)	Energy [eV] lower	-	upper	Transition lower	-	upper	Quantum number lower	-	upper	Comment
165.5320	Cu I	0		-		-		-				
168.8090	Cu I	0		-		-		-				
169.1080	Cu I	0		-		-		-				
170.3840	Cu I	0		-		-		-				
171.3360	Cu I	0		-		-		-				
172.5660	Cu I	0		-		-		-				
174.1570	Cu I	0		-		-		-				
177.4820	Cu I	0		-		-		-				
182.5350	Cu I	0		-		-		-				
202.4325	Cu I	110	0.00	-	6.12	4s 2S	-	5p 2P	1/2	-	1/2	
202.4338	Cu I	219	0.00	-	6.12	4s 2S	-	5p 2P	1/2	-	3/2	
213.8540	Cu I	19	1.39	-	7.18	4s2 2D	-	7p 2P	5/2	-	3/2	
216.5090	Cu I	165	0.00	-	5.72	4s 2S	-	4p' 2D	1/2	-	3/2	
217.8940	Cu I	268	0.00	-	5.69	4s 2S	-	4p' 2P	1/2	-	3/2	
218.1720	Cu I	160	0.00	-	5.68	4s 2S	-	4p' 2P	1/2	-	1/2	
219.9580	Cu I	75	1.39	-	7.02	4s2 2D	-	4p'' 2D	5/2	-	5/2	
219.9750	Cu I	913	1.64	-	7.28	4s2 2D	-	4p'' 2D	3/2	-	3/2	
221.4580	Cu I	29	1.39	-	6.99	4s2 2D	-	4p'' 2P	5/2	-	3/2	
221.5650	Cu I	0		-		-		-				
222.5700	Cu I	72	0.00	-	5.57	4s 2S	-	4p' 4D	1/2	-	1/2	
222.7780	Cu I	85	1.64	-	7.21	4s2 2D	-	4p'' 2F	3/2	-	5/2	
223.0080	Cu I	69	1.39	-	6.95	4s2 2D	-	4p'' 2F	5/2	-	7/2	
223.8450	Cu I	0		-		-		-				
224.4260	Cu I	7	0.00	-	5.52	4s 2S	-	4p' 4D	1/2	-	3/2	
226.0530	Cu I	0		-		-		-				
226.3080	Cu I	0		-		-		-				
229.3840	Cu I	29	1.39	-	6.79	4s2 2D	-	6p 2P	5/2	-	3/2	
230.3120	Cu I	0		-		-		-				
239.2630	Cu I	0		-		-		-				
240.6660	Cu I	0		-		-		-				
244.1640	Cu I	4	0.00	-	5.08	4s 2S	-	4p' 4P	1/2	-	1/2	
249.2150	Cu I	12	0.00	-	4.97	4s 2S	-	4p' 4P	1/2	-	3/2	
261.8370	Cu I	127	1.39	-	6.12	4s2 2D	-	5p 2P	5/2	-	3/2	

262.6690	Cu I	80	5.08	-	9.80	4p' 4P	-	5d' 4D	1/2	-	3/2
262.9968	Cu I	90	4.84	-	9.55	4p' 4P	-	5d' 4P	5/2	-	5/2
263.4931	Cu I	98	4.84	-	9.54	4p' 4P	-	5d' 4S	5/2	-	3/2
264.5309	Cu I	34	4.97	-	9.66	4p' 4P	-	5d' 4D	3/2	-	5/2
264.9839	Cu I	49	4.97	-	9.65	4p' 4P	-	5d' 4P	3/2	-	3/2
276.6370	Cu I	45	1.64	-	6.12	4s2 2D	-	5p 2P	3/2	-	3/2
282.4370	Cu I	57	1.39	-	5.78	4s2 2D	-	4p' 2D	5/2	-	5/2
284.6492	Cu I	11	5.08	-	9.43	4p' 4P	-	6s' 4D	1/2	-	1/2
285.8223	Cu I	67	4.84	-	9.17	4p' 4P	-	6s' 4D	5/2	-	7/2
285.8731	Cu I	1	1.39	-	5.72	4s2 2D	-	4p' 2D	5/2	-	3/2
288.2931	Cu I	9	1.39	-	5.69	4s2 2D	-	4p' 2P	5/2	-	3/2
289.0827	Cu I	88	5.51	-	9.79	4p' 4D	-	5d' 4G	5/2	-	7/2
289.1637	Cu I	78	5.52	-	9.81	4p' 4D	-	5d' 4F	3/2	-	5/2
293.1704	Cu I	7	5.08	-	9.30	4p' 4P	-	6s' 4D	1/2	-	3/2
293.3062	Cu I	13	4.84	-	9.06	4p' 4P	-	4d' 4G	5/2	-	7/2
296.1160	Cu I	40	1.39	-	5.57	4s2 2D	-	4p' 2F	5/2	-	7/2
297.8287	Cu I	121	5.40	-	9.56	4p' 4D	-	5d' 4F	7/2	-	9/2
297.9379	Cu I	103	5.40	-	9.56	4p' 4D	-	5d' 4D	7/2	-	7/2
299.7360	Cu I	10	1.64	-	5.78	4s2 2D	-	4p' 2D	3/2	-	5/2
299.8383	Cu I	1	1.39	-	5.52	4s2 2D	-	4p' 4D	5/2	-	3/2
301.0840	Cu I	11	1.39	-	5.51	4s2 2D	-	4p' 4D	5/2	-	5/2
301.2016	Cu I	78	4.97	-	9.09	4p' 4P	-	4d' 4F	3/2	-	5/2
301.4851	Cu I	33	5.10	-	9.21	4p' 4F	-	6s' 4D	7/2	-	5/2
302.1545	Cu I	108	5.07	-	9.17	4p' 4F	-	6s' 4D	9/2	-	7/2
302.2603	Cu I	70	4.84	-	8.94	4p' 4P	-	4d' 4F	5/2	-	7/2
303.0257	Cu I	21	4.97	-	9.06	4p' 4P	-	4d' 4D	3/2	-	3/2
303.6100	Cu I	14	1.64	-	5.72	4s2 2D	-	4p' 2D	3/2	-	3/2
304.4022	Cu I	22	5.10	-	9.17	4p' 4F	-	6s' 4D	7/2	-	7/2
305.2546	Cu I	11	5.15	-	9.21	4p' 4F	-	6s' 4D	5/2	-	5/2
306.3410	Cu I	9	1.64	-	5.69	4s2 2D	-	4p' 2P	3/2	-	3/2
306.8892	Cu I	0	1.64	-	5.68	4s2 2D	-	4p' 2P	3/2	-	1/2
307.3800	Cu I	2	1.39	-	5.42	4s2 2D	-	4p' 2F	5/2	-	5/2
308.8123	Cu I	75	5.08	-	9.09	4p' 4P	-	4d' 4F	1/2	-	3/2
309.3990	Cu I	4	1.39	-	5.40	4s2 2D	-	4p' 4D	5/2	-	7/2
309.9930	Cu I	296	4.84	-	8.84	4p' 4P	-	4d' 4D	5/2	-	7/2
310.8600	Cu I	0		-		-		-			
311.3473	Cu I	27	4.84	-	8.82	4p' 4P	-	4d' 2F	5/2	-	7/2
312.0437	Cu I	34	4.97	-	8.95	4p' 4P	-	4d' 2P	3/2	-	1/2
312.6110	Cu I	447	4.84	-	8.80	4p' 4P	-	4d' 4S	5/2	-	3/2
312.8692	Cu I	195	4.97	-	8.94	4p' 4P	-	4d' 4D	3/2	-	5/2
314.0313	Cu I	103	4.84	-	8.78	4p' 4P	-	4d' 2P	5/2	-	3/2
314.2436	Cu I	270	4.97	-	8.92	4p' 4P	-	4d' 4P	3/2	-	3/2

314.6814	Cu I	148	4.97	-	8.91	4p' 4P	-	4d' 4P	3/2	-	1/2
314.8341	Cu I	11	5.15	-	9.09	4p' 4F	-	4d' 4F	5/2	-	3/2
314.9510	Cu I	25	5.15	-	9.09	4p' 4F	-	4d' 4F	5/2	-	5/2
315.6630	Cu I	1	1.64	-	5.57	4s2 2D	-	4p' 4D	3/2	-	1/2
316.0059	Cu I	26	5.15	-	9.08	4p' 4F	-	4d' 4G	5/2	-	5/2
316.9675	Cu I	205	5.15	-	9.06	4p' 4F	-	4d' 4G	5/2	-	7/2
317.1649	Cu I	22	5.52	-	9.43	4p' 4D	-	6s' 4D	3/2	-	1/2
319.4100	Cu I	10	1.64	-	5.52	4s2 2D	-	4p' 4D	3/2	-	3/2
320.8230	Cu I	5	1.64	-	5.51	4s2 2D	-	4p' 4D	3/2	-	5/2
322.3420	Cu I	129	5.24	-	9.09	4p' 4F	-	4d' 4F	3/2	-	3/2
322.4646	Cu I	159	5.24	-	9.09	4p' 4F	-	4d' 4F	3/2	-	5/2
323.1166	Cu I	235	5.10	-	8.94	4p' 4F	-	4d' 4F	7/2	-	7/2
323.3895	Cu I	78	5.10	-	8.94	4p' 4F	-	4d' 4D	7/2	-	5/2
323.5706	Cu I	417	5.24	-	9.08	4p' 4F	-	4d' 4G	3/2	-	5/2
324.3160	Cu I	708	5.10	-	8.92	4p' 4F	-	4d' 4G	7/2	-	9/2
324.7540	Cu I	867	0.00	-	3.82	4s 2S	-	4p 2P	1/2	-	3/2
326.8283	Cu I	214	5.15	-	8.95	4p' 4F	-	4d' 2F	5/2	-	5/2
327.3960	Cu I	438	0.00	-	3.79	4s 2S	-	4p 2P	1/2	-	1/2
327.9812	Cu I	7	1.64	-	5.42	4s2 2D	-	4p' 2F	3/2	-	5/2
328.2720	Cu I	538	5.15	-	8.93	4p' 4F	-	4d' 2G	5/2	-	7/2
328.5079	Cu I	16	3.82	-	7.59	4p 2P	-	11d 2D	3/2	-	5/2
328.6189	Cu I	13	3.79	-	7.56	4p 2P	-	10d 2D	1/2	-	3/2
329.0540	Cu I	646	5.07	-	8.84	4p' 4F	-	4d' 4F	9/2	-	9/2
329.2390	Cu I	57	5.15	-	8.92	4p' 4F	-	4d' 4P	5/2	-	3/2
329.2823	Cu I	1	1.39	-	5.15	4s2 2D	-	4p' 4F	5/2	-	5/2
330.7950	Cu I	4366	5.07	-	8.82	4p' 4F	-	4d' 4G	9/2	-	11/2
331.3195	Cu I	23	3.82	-	7.56	4p 2P	-	10d 2D	3/2	-	5/2
331.3242	Cu I	3	3.82	-	7.56	4p 2P	-	10d 2D	3/2	-	3/2
331.7214	Cu I	324	5.10	-	8.84	4p' 4F	-	4d' 4F	7/2	-	9/2
331.9674	Cu I	219	5.10	-	8.84	4p' 4F	-	4d' 4D	7/2	-	7/2
332.5808	Cu I	19	3.79	-	7.51	4p 2P	-	9d 2D	1/2	-	3/2
332.9627	Cu I	92	5.10	-	8.83	4p' 4F	-	4d' 4P	7/2	-	5/2
333.5223	Cu I	94	5.10	-	8.82	4p' 4F	-	4d' 2F	7/2	-	7/2
333.7840	Cu I	6	1.39	-	5.10	4s2 2D	-	4p' 4F	5/2	-	7/2
334.9266	Cu I	240	5.24	-	8.95	4p' 4F	-	4d' 2F	3/2	-	5/2
335.3462	Cu I	33	3.82	-	7.51	4p 2P	-	9d 2D	3/2	-	5/2
335.3520	Cu I	4	3.82	-	7.51	4p 2P	-	9d 2D	3/2	-	3/2
335.4478	Cu I	110	5.24	-	8.94	4p' 4F	-	4d' 2D	3/2	-	3/2
336.5345	Cu I	282	5.10	-	8.79	4p' 4F	-	4d' 2G	7/2	-	9/2
337.5664	Cu I	34	5.15	-	8.83	4p' 4F	-	4d' 4P	5/2	-	5/2
338.1416	Cu I	182	5.15	-	8.82	4p' 4F	-	4d' 2F	5/2	-	7/2

338.4810	Cu I	34	5.15	-	8.82	4p' 4F	-	4d' 2D	5/2	-	5/2
338.5391	Cu I	29	3.79	-	7.45	4p 2P	-	8d 2D	1/2	-	3/2
339.2017	Cu I	14	5.42	-	9.08	4p' 2F	-	4d' 4G	5/2	-	5/2
339.6315	Cu I	14	5.15	-	8.80	4p' 4F	-	4d' 4S	5/2	-	3/2
341.3335	Cu I	121	5.68	-	9.31	4p' 2P	-	4d'' 2P	1/2	-	1/2
341.4013	Cu I	52	3.82	-	7.45	4p 2P	-	8d 2D	3/2	-	5/2
341.4108	Cu I	6	3.82	-	7.45	4p 2P	-	8d 2D	3/2	-	3/2
342.0144	Cu I	34	5.69	-	9.31	4p' 2P	-	4d'' 2P	3/2	-	1/2
343.3957	Cu I	1	3.79	-	7.40	4p 2P	-	9s 2S	1/2	-	1/2
343.6550	Cu I	39	5.72	-	9.33	4p' 2D	-	4d'' 2P	3/2	-	3/2
344.0520	Cu I	1	1.64	-	5.24	4s2 2D	-	4p' 4F	3/2	-	3/2
345.7850	Cu I	1	1.39	-	4.97	4s2 2D	-	4p' 4P	5/2	-	3/2
345.9408	Cu I	35	5.51	-	9.09	4p' 4D	-	4d' 4F	5/2	-	5/2
346.3508	Cu I	2	3.82	-	7.40	4p 2P	-	9s 2S	3/2	-	1/2
347.2139	Cu I	90	5.51	-	9.08	4p' 4D	-	4d' 4G	5/2	-	5/2
347.4569	Cu I	14	5.52	-	9.09	4p' 4D	-	4d' 4F	3/2	-	3/2
347.5994	Cu I	381	5.52	-	9.09	4p' 4D	-	4d' 4F	3/2	-	5/2
348.1606	Cu I	18	3.79	-	7.35	4p 2P	-	7d 2D	1/2	-	3/2
348.3760	Cu I	538	5.51	-	9.06	4p' 4D	-	4d' 4G	5/2	-	7/2
348.7563	Cu I	62	5.78	-	9.33	4p' 2D	-	4d'' 2P	5/2	-	3/2
348.8848	Cu I	59	5.52	-	9.08	4p' 4D	-	4d' 4G	3/2	-	5/2
349.8056	Cu I	58	5.40	-	8.94	4p' 4D	-	4d' 4F	7/2	-	7/2
350.0310	Cu I	53	5.52	-	9.06	4p' 4D	-	4d' 4D	3/2	-	3/2
350.7423	Cu I	22	5.40	-	8.93	4p' 4D	-	4d' 2G	7/2	-	7/2
351.1831	Cu I	88	3.82	-	7.35	4p 2P	-	7d 2D	3/2	-	5/2
351.1986	Cu I	10	3.82	-	7.35	4p 2P	-	7d 2D	3/2	-	3/2
351.2112	Cu I	332	5.40	-	8.92	4p' 4D	-	4d' 4G	7/2	-	9/2
351.7025	Cu I	46	5.42	-	8.95	4p' 2F	-	4d' 2F	5/2	-	5/2
352.0015	Cu I	289	5.57	-	9.09	4p' 4D	-	4d' 4F	1/2	-	3/2
352.4230	Cu I	490	5.42	-	8.94	4p' 2F	-	4d' 4F	5/2	-	7/2
352.7476	Cu I	209	5.42	-	8.94	4p' 2F	-	4d' 4D	5/2	-	5/2
353.0380	Cu I	3	1.64	-	5.15	4s2 2D	-	4p' 4F	3/2	-	5/2
353.3737	Cu I	240	5.42	-	8.93	4p' 2F	-	4d' 2G	5/2	-	7/2
354.4957	Cu I	76	5.42	-	8.92	4p' 2F	-	4d' 4P	5/2	-	3/2
354.6436	Cu I	30	5.57	-	9.06	4p' 4D	-	4d' 4D	1/2	-	3/2
356.6127	Cu I	4	3.79	-	7.26	4p 2P	-	8s 2S	1/2	-	1/2
359.4016	Cu I	1	1.39	-	4.84	4s2 2D	-	4p' 4P	5/2	-	5/2
359.8007	Cu I	7	3.82	-	7.26	4p 2P	-	8s 2S	3/2	-	1/2
359.9130	Cu I	2512	5.40	-	8.84	4p' 4D	-	4d' 4F	7/2	-	9/2
360.2030	Cu I	2512	5.40	-	8.84	4p' 4D	-	4d' 4D	7/2	-	7/2
360.9299	Cu I	1	1.64	-	5.08	4s2 2D	-	4p' 4P	3/2	-	1/2
361.0798	Cu I	92	5.51	-	8.94	4p' 4D	-	4d' 4F	5/2	-	7/2

361.3744	Cu I	240	5.40	-	8.83	4p' 4D	-	4d' 4P	7/2	-	5/2
361.4206	Cu I	100	5.51	-	8.94	4p' 4D	-	4d' 4D	5/2	-	5/2
362.0337	Cu I	80	5.40	-	8.82	4p' 4D	-	4d' 2F	7/2	-	7/2
362.1233	Cu I	240	5.52	-	8.95	4p' 4D	-	4d' 2F	3/2	-	5/2
362.4228	Cu I	76	5.40	-	8.82	4p' 4D	-	4d' 2D	7/2	-	5/2
362.7328	Cu I	209	5.52	-	8.94	4p' 4D	-	4d' 2D	3/2	-	3/2
362.9781	Cu I	17	5.42	-	8.84	4p' 2F	-	4d' 4D	5/2	-	7/2
363.2560	Cu I	59	5.51	-	8.92	4p' 4D	-	4d' 4P	5/2	-	3/2
363.5928	Cu I	166	5.68	-	9.09	4p' 2P	-	4d' 4F	1/2	-	3/2
364.1684	Cu I	55	5.42	-	8.83	4p' 2F	-	4d' 4P	5/2	-	5/2
364.5221	Cu I	108	5.69	-	9.09	4p' 2P	-	4d' 4F	3/2	-	5/2
364.8380	Cu I	45	5.42	-	8.82	4p' 2F	-	4d' 2F	5/2	-	7/2
365.0851	Cu I	22	5.52	-	8.92	4p' 4D	-	4d' 4P	3/2	-	3/2
365.2330	Cu I	264	5.42	-	8.82	4p' 2F	-	4d' 2D	5/2	-	5/2
365.4230	Cu I	96	3.79	-	7.18	4p 2P	-	6d 2D	1/2	-	3/2
365.5856	Cu I	142	5.40	-	8.79	4p' 4D	-	4d' 2G	7/2	-	9/2
365.6762	Cu I	67	5.52	-	8.91	4p' 4D	-	4d' 4P	3/2	-	1/2
365.9360	Cu I	86	5.69	-	9.08	4p' 2P	-	4d' 4G	3/2	-	5/2
366.5730	Cu I	43	5.42	-	8.80	4p' 2F	-	4d' 4S	5/2	-	3/2
367.1972	Cu I	75	5.69	-	9.06	4p' 2P	-	4d' 4D	3/2	-	3/2
368.7434	Cu I	127	3.82	-	7.18	4p 2P	-	6d 2D	3/2	-	5/2
368.7712	Cu I	20	3.82	-	7.18	4p 2P	-	6d 2D	3/2	-	3/2
369.5322	Cu I	23	5.57	-	8.93	4p' 2F	-	4d' 2G	7/2	-	7/2
369.9106	Cu I	23	5.72	-	9.08	4p' 2D	-	4d' 4G	3/2	-	5/2
370.0529	Cu I	121	5.57	-	8.92	4p' 2F	-	4d' 4G	7/2	-	9/2
371.1994	Cu I	58	5.72	-	9.06	4p' 2D	-	4d' 4D	3/2	-	3/2
372.0763	Cu I	0	1.64	-	4.97	4s2 2D	-	4p' 4P	3/2	-	3/2
372.1680	Cu I	19	5.51	-	8.84	4p' 4D	-	4d' 4D	5/2	-	7/2
373.4195	Cu I	76	5.51	-	8.83	4p' 4D	-	4d' 4P	5/2	-	5/2
374.1235	Cu I	126	5.51	-	8.82	4p' 4D	-	4d' 2F	5/2	-	7/2
374.5390	Cu I	78	5.51	-	8.82	4p' 4D	-	4d' 2D	5/2	-	5/2
375.9482	Cu I	42	5.51	-	8.80	4p' 4D	-	4d' 4S	5/2	-	3/2
376.4839	Cu I	18	5.52	-	8.82	4p' 4D	-	4d' 2D	3/2	-	5/2
377.1887	Cu I	96	5.78	-	9.06	4p' 2D	-	4d' 4G	5/2	-	7/2
378.0056	Cu I	13	5.51	-	8.78	4p' 4D	-	4d' 2P	5/2	-	3/2
379.7258	Cu I	16	5.57	-	8.84	4p' 2F	-	4d' 4F	7/2	-	9/2
379.9867	Cu I	27	5.52	-	8.78	4p' 4D	-	4d' 2P	3/2	-	3/2
380.0481	Cu I	45	5.57	-	8.84	4p' 2F	-	4d' 4D	7/2	-	7/2
380.5227	Cu I	78	5.69	-	8.95	4p' 2P	-	4d' 2P	3/2	-	1/2
381.2002	Cu I	41	5.69	-	8.94	4p' 2P	-	4d' 2D	3/2	-	3/2
381.3532	Cu I	22	5.57	-	8.83	4p' 2F	-	4d' 4P	7/2	-	5/2
381.7509	Cu I	12	5.69	-	8.94	4p' 2P	-	4d' 4D	3/2	-	5/2

382.0875	Cu I	54	5.57 -	8.82	4p' 2F	-	4d' 2F	7/2	-	7/2
382.5042	Cu I	13	3.79 -	7.03	4p 2P	-	7s 2S	1/2	-	1/2
383.7991	Cu I	12	5.69 -	8.92	4p' 2P	-	4d' 4P	3/2	-	3/2
386.0460	Cu I	230	5.57 -	8.79	4p' 2F	-	4d' 2G	7/2	-	9/2
386.1743	Cu I	14	3.82 -	7.03	4p 2P	-	7s 2S	3/2	-	1/2
388.1735	Cu I	18	5.72 -	8.92	4p' 2D	-	4d' 4P	3/2	-	3/2
388.8417	Cu I	14	5.72 -	8.91	4p' 2D	-	4d' 4P	3/2	-	1/2
392.1267	Cu I	39	5.78 -	8.94	4p' 2D	-	4d' 4F	5/2	-	7/2
392.5287	Cu I	47	5.78 -	8.94	4p' 2D	-	4d' 4D	5/2	-	5/2
393.3041	Cu I	23	5.78 -	8.93	4p' 2D	-	4d' 2G	5/2	-	7/2
394.6945	Cu I	15	5.78 -	8.92	4p' 2D	-	4d' 4P	5/2	-	3/2
395.1626	Cu I	6	5.69 -	8.83	4p' 2P	-	4d' 4P	3/2	-	5/2
396.4165	Cu I	20	5.69 -	8.82	4p' 2P	-	4d' 2D	3/2	-	5/2
397.5633	Cu I	12	4.97 -	8.09	4p' 4P	-	5s' 2D	3/2	-	3/2
397.9955	Cu I	8	5.69 -	8.80	4p' 2P	-	4d' 4S	3/2	-	3/2
399.3695	Cu I	4	5.68 -	8.78	4p' 2P	-	4d' 2P	1/2	-	3/2
399.8014	Cu I	8	5.72 -	8.83	4p' 2D	-	4d' 4P	3/2	-	5/2
400.3020	Cu I	37	5.69 -	8.78	4p' 2P	-	4d' 2P	3/2	-	3/2
401.0849	Cu I	12	5.72 -	8.82	4p' 2D	-	4d' 2D	3/2	-	5/2
401.5817	Cu I	3	3.79 -	6.87	4p 2P	-	4f 2F	1/2	-	5/2
402.2630	Cu I	140	3.79 -	6.87	4p 2P	-	5d 2D	1/2	-	3/2
402.7014	Cu I	39	5.72 -	8.80	4p' 2D	-	4d' 4S	3/2	-	3/2
405.0629	Cu I	31	5.72 -	8.78	4p' 2D	-	4d' 2P	3/2	-	3/2
405.2383	Cu I	17	5.78 -	8.84	4p' 2D	-	4d' 4D	5/2	-	7/2
406.2640	Cu I	312	3.82 -	6.87	4p 2P	-	5d 2D	3/2	-	5/2
406.3235	Cu I	48	3.82 -	6.87	4p 2P	-	5d 2D	3/2	-	3/2
406.9531	Cu I	3	4.84 -	7.88	4p' 4P	-	5s' 4D	5/2	-	3/2
407.3197	Cu I	17	4.97 -	8.02	4p' 4P	-	5s' 2D	3/2	-	5/2
407.5578	Cu I	105	5.78 -	8.82	4p' 2D	-	4d' 2F	5/2	-	7/2
408.0509	Cu I	76	5.78 -	8.82	4p' 2D	-	4d' 2D	5/2	-	5/2
410.4217	Cu I	16	4.97 -	7.99	4p' 4P	-	5s' 4D	3/2	-	1/2
411.1296	Cu I	13	5.08 -	8.09	4p' 4P	-	5s' 2D	1/2	-	3/2
412.1690	Cu I	29	5.78 -	8.78	4p' 2D	-	4d' 2P	5/2	-	3/2
412.3271	Cu I	48	5.42 -	8.43	4p' 2F	-	5s'' 2D	5/2	-	3/2
417.7740	Cu I	25	4.84 -	7.80	4p' 4P	-	5s' 4D	5/2	-	5/2
423.0941	Cu I	27	5.40 -	8.32	4p' 4D	-	5s'' 2D	7/2	-	5/2
424.2266	Cu I	36	5.51 -	8.43	4p' 4D	-	5s'' 2D	5/2	-	3/2
424.9000	Cu I	108	5.08 -	7.99	4p' 4P	-	5s' 4D	1/2	-	1/2
425.9454	Cu I	117	4.97 -	7.88	4p' 4P	-	5s' 4D	3/2	-	3/2
427.5110	Cu I	697	4.84 -	7.74	4p' 4P	-	5s' 4D	5/2	-	7/2
432.8742	Cu I	11	5.15 -	8.02	4p' 4F	-	5s' 2D	5/2	-	5/2

433.5984	Cu I	17	5.57	-	8.43	4p' 4D	-	5s'' 2D	1/2	-	3/2
435.4626	Cu I	18	5.24	-	8.09	4p' 4F	-	5s' 2D	3/2	-	3/2
437.8146	Cu I	228	4.97	-	7.80	4p' 4P	-	5s' 4D	3/2	-	5/2
441.5557	Cu I	73	5.08	-	7.88	4p' 4P	-	5s' 4D	1/2	-	3/2
448.0400	Cu I	19	3.79	-	6.55	4p 2P	-	6s 2S	1/2	-	1/2
450.7408	Cu I	458	5.57	-	8.32	4p' 2F	-	5s'' 2D	7/2	-	5/2
450.9400	Cu I	181	5.24	-	7.99	4p' 4F	-	5s' 4D	3/2	-	1/2
452.5127	Cu I	57	5.69	-	8.43	4p' 2P	-	5s'' 2D	3/2	-	3/2
453.0800	Cu I	57	3.82	-	6.55	4p 2P	-	6s 2S	3/2	-	1/2
453.9700	Cu I	316	5.15	-	7.88	4p' 4F	-	5s' 4D	5/2	-	3/2
458.7000	Cu I	487	5.10	-	7.80	4p' 4F	-	5s' 4D	7/2	-	5/2
464.2538	Cu I	96	5.42	-	8.09	4p' 2F	-	5s' 2D	5/2	-	3/2
465.1120	Cu I	1089	5.07	-	7.74	4p' 4F	-	5s' 4D	9/2	-	7/2
467.4780	Cu I	154	5.15	-	7.80	4p' 4F	-	5s' 4D	5/2	-	5/2
469.7470	Cu I	71	5.24	-	7.88	4p' 4F	-	5s' 4D	3/2	-	3/2
470.4600	Cu I	166	5.10	-	7.74	4p' 4F	-	5s' 4D	7/2	-	7/2
476.7416	Cu I	155	5.72	-	8.32	4p' 2D	-	5s'' 2D	3/2	-	5/2
477.6128	Cu I	17	5.42	-	8.02	4p' 2F	-	5s' 2D	5/2	-	5/2
479.7025	Cu I	9	5.15	-	7.74	4p' 4F	-	5s' 4D	5/2	-	7/2
484.2242	Cu I	14	5.24	-	7.80	4p' 4F	-	5s' 4D	3/2	-	5/2
486.6156	Cu I	324	5.78	-	8.32	4p' 2D	-	5s'' 2D	5/2	-	5/2
501.6629	Cu I	123	5.52	-	7.99	4p' 4D	-	5s' 4D	3/2	-	1/2
503.4254	Cu I	93	5.42	-	7.88	4p' 2F	-	5s' 4D	5/2	-	3/2
507.6125	Cu I	179	5.57	-	8.02	4p' 2F	-	5s' 2D	7/2	-	5/2
510.5540	Cu I	31	1.39	-	3.82	4s2 2D	-	4p 2P	5/2	-	3/2
511.1915	Cu I	83	5.57	-	7.99	4p' 4D	-	5s' 4D	1/2	-	1/2
514.4098	Cu I	166	5.40	-	7.80	4p' 4D	-	5s' 4D	7/2	-	5/2
515.3240	Cu I	1649	3.79	-	6.19	4p 2P	-	4d 2D	1/2	-	3/2
520.0897	Cu I	117	5.42	-	7.80	4p' 2F	-	5s' 4D	5/2	-	5/2
521.2774	Cu I	47	5.51	-	7.88	4p' 4D	-	5s' 4D	5/2	-	3/2
521.8200	Cu I	2993	3.82	-	6.19	4p 2P	-	4d 2D	3/2	-	5/2
522.0100	Cu I	357	3.82	-	6.19	4p 2P	-	4d 2D	3/2	-	3/2
525.0524	Cu I	141	5.52	-	7.88	4p' 4D	-	5s' 4D	3/2	-	3/2
529.2520	Cu I	367	5.40	-	7.74	4p' 4D	-	5s' 4D	7/2	-	7/2
535.2653	Cu I	24	5.42	-	7.74	4p' 2F	-	5s' 4D	5/2	-	7/2
535.4995	Cu I	71	5.57	-	7.88	4p' 4D	-	5s' 4D	1/2	-	3/2
536.0066	Cu I	22	5.68	-	7.99	4p' 2P	-	5s' 4D	1/2	-	1/2
537.6876	Cu I	6	5.69	-	7.99	4p' 2P	-	5s' 4D	3/2	-	1/2
539.1656	Cu I	136	5.51	-	7.80	4p' 4D	-	5s' 4D	5/2	-	5/2
543.2051	Cu I	78	5.52	-	7.80	4p' 4D	-	5s' 4D	3/2	-	5/2
546.3124	Cu I	16	5.72	-	7.99	4p' 2D	-	5s' 4D	3/2	-	1/2
555.4923	Cu I	23	5.51	-	7.74	4p' 4D	-	5s' 4D	5/2	-	7/2

560.9866	Cu I	3	5.35 -	7.56	5s 2S	-	11p 2P	1/2	-	3/2
560.9866	Cu I	2	5.35 -	7.56	5s 2S	-	11p 2P	1/2	-	1/2
570.0240	Cu I	5	1.64 -	3.82	4s2 2D	-	4p 2P	3/2	-	3/2
572.4250	Cu I	3	5.35 -	7.51	5s 2S	-	10p 2P	1/2	-	1/2
572.6513	Cu I	4	5.35 -	7.51	5s 2S	-	10p 2P	1/2	-	3/2
573.2323	Cu I	17	5.57 -	7.74	4p' 2F	-	5s' 4D	7/2	-	7/2
578.2130	Cu I	20	1.64 -	3.79	4s2 2D	-	4p 2P	3/2	-	1/2
589.8769	Cu I	4	5.35 -	7.45	5s 2S	-	9p 2P	1/2	-	1/2
590.3855	Cu I	7	5.35 -	7.45	5s 2S	-	9p 2P	1/2	-	3/2
617.6484	Cu I	10	5.35 -	7.36	5s 2S	-	8p 2P	1/2	-	1/2
619.4794	Cu I	15	5.35 -	7.35	5s 2S	-	8p 2P	1/2	-	3/2
622.3715	Cu I	47	6.95 -	8.94	4p'' 2F	-	4d' 4F	7/2	-	7/2
632.5370	Cu I	6	5.78 -	7.74	4p' 2D	-	5s' 4D	5/2	-	7/2
647.4223	Cu I	68	7.02 -	8.94	4p'' 2D	-	4d' 4F	5/2	-	7/2
648.5189	Cu I	46	7.02 -	8.94	4p'' 2D	-	4d' 4D	5/2	-	5/2
650.6381	Cu I	12	7.02 -	8.93	4p'' 2D	-	4d' 2G	5/2	-	7/2
654.4519	Cu I	18	7.02 -	8.92	4p'' 2D	-	4d' 4P	5/2	-	3/2
655.1017	Cu I	14	6.95 -	8.84	4p'' 2F	-	4d' 4F	7/2	-	9/2
658.3458	Cu I	22	7.21 -	9.09	4p'' 2F	-	4d' 4F	5/2	-	5/2
659.9604	Cu I	12	6.95 -	8.83	4p'' 2F	-	4d' 4P	7/2	-	5/2
662.1626	Cu I	100	6.95 -	8.82	4p'' 2F	-	4d' 2F	7/2	-	7/2
662.9719	Cu I	31	7.21 -	9.08	4p'' 2F	-	4d' 4G	5/2	-	5/2
667.2183	Cu I	124	7.21 -	9.06	4p'' 2F	-	4d' 4G	5/2	-	7/2
674.1418	Cu I	339	6.95 -	8.79	4p'' 2F	-	4d' 2G	7/2	-	9/2
674.9647	Cu I	35	5.35 -	7.18	5s 2S	-	7p 2P	1/2	-	3/2
688.1972	Cu I	31	7.02 -	8.83	4p'' 2D	-	4d' 4P	5/2	-	5/2
689.0890	Cu I	36	7.12 -	8.92	7p 2P	-	4d' 4P	1/2	-	3/2
690.5923	Cu I	166	7.02 -	8.82	4p'' 2D	-	4d' 2F	5/2	-	7/2
696.8351	Cu I	15	7.02 -	8.80	4p'' 2D	-	4d' 4S	5/2	-	3/2
699.9840	Cu I	3	5.35 -	7.12	5s 2S	-	7p 2P	1/2	-	1/2
715.4277	Cu I	23	7.21 -	8.94	4p'' 2F	-	4d' 4F	5/2	-	7/2
793.3130	Cu I	425	3.79 -	5.35	4p 2P	-	5s 2S	1/2	-	1/2
809.2630	Cu I	902	3.82 -	5.35	4p 2P	-	5s 2S	3/2	-	1/2
840.7743	Cu I	20	5.35 -	6.82	5s 2S	-	6p 2P	1/2	-	1/2
844.8877	Cu I	29	6.12 -	7.59	5p 2P	-	11d 2D	3/2	-	5/2
858.3653	Cu I	10	5.35 -	6.79	5s 2S	-	6p 2P	1/2	-	3/2
863.7384	Cu I	40	6.12 -	7.56	5p 2P	-	10d 2D	3/2	-	5/2
863.7705	Cu I	5	6.12 -	7.56	5p 2P	-	10d 2D	3/2	-	3/2
863.7936	Cu I	23	6.12 -	7.56	5p 2P	-	10d 2D	1/2	-	3/2
891.6487	Cu I	62	6.12 -	7.51	5p 2P	-	9d 2D	3/2	-	5/2
891.6901	Cu I	7	6.12 -	7.51	5p 2P	-	9d 2D	3/2	-	3/2
891.7147	Cu I	34	6.12 -	7.51	5p 2P	-	9d 2D	1/2	-	3/2

906.9615	Cu I	1	6.19 -	7.56	4d 2D	-	11p 2P	3/2	-	1/2
906.9615	Cu I	0	6.19 -	7.56	4d 2D	-	11p 2P	3/2	-	3/2
907.5261	Cu I	1	6.19 -	7.56	4d 2D	-	11p 2P	5/2	-	3/2
935.7766	Cu I	103	6.12 -	7.45	5p 2P	-	8d 2D	3/2	-	5/2
935.8484	Cu I	12	6.12 -	7.45	5p 2P	-	8d 2D	3/2	-	3/2
935.8756	Cu I	57	6.12 -	7.45	5p 2P	-	8d 2D	1/2	-	3/2
937.2399	Cu I	1	6.19 -	7.51	4d 2D	-	10p 2P	3/2	-	1/2
937.8466	Cu I	1	6.19 -	7.51	4d 2D	-	10p 2P	3/2	-	3/2
938.4502	Cu I	2	6.19 -	7.51	4d 2D	-	10p 2P	5/2	-	3/2
973.9236	Cu I	12	6.12 -	7.40	5p 2P	-	9s 2S	3/2	-	1/2
973.9531	Cu I	6	6.12 -	7.40	5p 2P	-	9s 2S	1/2	-	1/2
984.9516	Cu I	1	6.19 -	7.45	4d 2D	-	9p 2P	3/2	-	1/2
986.3704	Cu I	1	6.19 -	7.45	4d 2D	-	9p 2P	3/2	-	3/2
987.0382	Cu I	3	6.19 -	7.45	4d 2D	-	9p 2P	5/2	-	3/2
100.1013	Cu II	3	2.83 -	15.22	4s 3D	-	5p 3F	2	-	2
100.4055	Cu II	19	2.72 -	15.07	4s 3D	-	5p 3D	3	-	3
100.4435	Cu II	0	2.98 -	15.32	4s 3D	-	5p 1D	1	-	2
100.6984	Cu II	1	2.98 -	15.29	4s 3D	-	5p 3D	1	-	1
100.7001	Cu II	0	3.26 -	15.57	4s 1D	-	4p 5P	2	-	1
100.8569	Cu II	5	2.72 -	15.01	4s 3D	-	4p 1F	3	-	3
100.8728	Cu II	38	2.83 -	15.12	4s 3D	-	5p 3D	2	-	2
101.0269	Cu II	9	3.26 -	15.53	4s 1D	-	4p 5P	2	-	2
101.0453	Cu II	4	3.26 -	15.53	4s 1D	-	4p 5P	2	-	3
101.0639	Cu II	4	2.72 -	14.99	4s 3D	-	4p 1D	3	-	2
101.1436	Cu II	0	2.98 -	15.23	4s 3D	-	5p 1P	1	-	1
101.1521	Cu II	7	2.72 -	14.98	4s 3D	-	5p 3F	3	-	4
101.2597	Cu II	12	2.72 -	14.96	4s 3D	-	5p 3F	3	-	3
101.2683	Cu II	1	2.98 -	15.22	4s 3D	-	5p 3F	1	-	2
101.3400	Cu II	3	2.83 -	15.07	4s 3D	-	5p 3D	2	-	3
101.7998	Cu II	4	2.83 -	15.01	4s 3D	-	4p 1F	2	-	3
101.8064	Cu II	0	2.98 -	15.15	4s 3D	-	5p 3P	1	-	0
101.8707	Cu II	0	2.72 -	14.89	4s 3D	-	5p 3P	3	-	2
101.9655	Cu II	0	2.83 -	14.99	4s 3D	-	5p 3P	2	-	1
102.0108	Cu II	4	2.83 -	14.99	4s 3D	-	4p 1D	2	-	2
102.0580	Cu II	3	2.98 -	15.12	4s 3D	-	5p 3D	1	-	2
102.2102	Cu II	2	2.83 -	14.96	4s 3D	-	5p 3F	2	-	3
102.7831	Cu II	75	3.26 -	15.32	4s 1D	-	5p 1D	2	-	2
102.8328	Cu II	3	2.83 -	14.89	4s 3D	-	5p 3P	2	-	2
102.9751	Cu II	14	2.72 -	14.76	4s 3D	-	4p 3F	3	-	2
103.0263	Cu II	9	2.72 -	14.75	4s 3D	-	4p 1G	3	-	4
103.0500	Cu II	0	3.26 -	15.29	4s 1D	-	5p 3D	2	-	1

103.1766	Cu II	1	2.98 -	14.99	4s 3D	-	5p 3P	1	-	1
103.2230	Cu II	1	2.98 -	14.99	4s 3D	-	4p 1D	1	-	2
103.3568	Cu II	15	3.26 -	15.25	4s 1D	-	5p 1F	2	-	3
103.5163	Cu II	0	3.26 -	15.23	4s 1D	-	5p 1P	2	-	1
103.6470	Cu II	43	3.26 -	15.22	4s 1D	-	5p 3F	2	-	2
103.9348	Cu II	131	2.72 -	14.65	4s 3D	-	4p 3F	3	-	3
103.9582	Cu II	94	2.83 -	14.76	4s 3D	-	4p 3F	2	-	2
104.0648	Cu II	0	2.98 -	14.89	4s 3D	-	5p 3P	1	-	2
104.4519	Cu II	214	2.72 -	14.59	4s 3D	-	4p 3F	3	-	4
104.4744	Cu II	274	3.26 -	15.12	4s 1D	-	5p 3D	2	-	2
104.9364	Cu II	88	2.83 -	14.65	4s 3D	-	4p 3F	2	-	3
104.9755	Cu II	87	3.26 -	15.07	4s 1D	-	5p 3D	2	-	3
105.0154	Cu II	46	2.83 -	14.64	4s 3D	-	4p 3D	2	-	1
105.0403	Cu II	45	2.72 -	14.52	4s 3D	-	4p 3D	3	-	2
105.2175	Cu II	42	2.98 -	14.76	4s 3D	-	4p 3F	1	-	2
105.4690	Cu II	138	3.26 -	15.01	4s 1D	-	4p 1F	2	-	3
105.5797	Cu II	114	2.72 -	14.46	4s 3D	-	4p 3G	3	-	3
105.6468	Cu II	0	3.26 -	14.99	4s 1D	-	5p 3P	2	-	1
105.6955	Cu II	208	3.26 -	14.99	4s 1D	-	4p 1D	2	-	2
105.8799	Cu II	211	2.72 -	14.43	4s 3D	-	4p 3D	3	-	3
105.9096	Cu II	15	3.26 -	14.96	4s 1D	-	5p 3F	2	-	3
106.0634	Cu II	178	2.83 -	14.52	4s 3D	-	4p 3D	2	-	2
106.3005	Cu II	138	2.98 -	14.64	4s 3D	-	4p 3D	1	-	1
106.5782	Cu II	3	3.26 -	14.89	4s 1D	-	5p 3P	2	-	2
106.6134	Cu II	13	2.83 -	14.46	4s 3D	-	4p 3G	2	-	3
106.9195	Cu II	88	2.83 -	14.43	4s 3D	-	4p 3D	2	-	3
107.0311	Cu II	3	2.72 -	14.30	4s 3D	-	4p 3G	3	-	4
107.3745	Cu II	80	2.98 -	14.52	4s 3D	-	4p 3D	1	-	2
107.7876	Cu II	3	3.26 -	14.76	4s 1D	-	4p 3F	2	-	2
108.0463	Cu II	0	2.72 -	14.19	4s 3D	-	4p 5F	3	-	2
108.6110	Cu II	1	2.72 -	14.13	4s 3D	-	4p 5F	3	-	3
108.8035	Cu II	0	2.83 -	14.23	4s 3D	-	4p 5F	2	-	1
108.8395	Cu II	17	3.26 -	14.65	4s 1D	-	4p 3F	2	-	3
108.9245	Cu II	6	3.26 -	14.64	4s 1D	-	4p 3D	2	-	1
109.1292	Cu II	1	2.83 -	14.19	4s 3D	-	4p 5F	2	-	2
109.4402	Cu II	7	2.72 -	14.05	4s 3D	-	4p 5F	3	-	4
109.7053	Cu II	5	2.83 -	14.13	4s 3D	-	4p 5F	2	-	3
110.0525	Cu II	1	3.26 -	14.52	4s 1D	-	4p 3D	2	-	2
110.1836	Cu II	0	2.98 -	14.23	4s 3D	-	4p 5F	1	-	1
110.5022	Cu II	0	2.72 -	13.94	4s 3D	-	4p 5G	3	-	2
110.5176	Cu II	2	2.98 -	14.19	4s 3D	-	4p 5F	1	-	2
110.6447	Cu II	4	3.26 -	14.46	4s 1D	-	4p 3G	2	-	3

110.9744	Cu II	3	3.26 -	14.43	4s 1D	-	4p 3D	2	-	3
111.1758	Cu II	1	2.72 -	13.87	4s 3D	-	4p 5G	3	-	3
111.6351	Cu II	0	2.83 -	13.94	4s 3D	-	4p 5G	2	-	2
111.9947	Cu II	2	2.72 -	13.79	4s 3D	-	4p 5G	3	-	4
112.3226	Cu II	1	2.83 -	13.87	4s 3D	-	4p 5G	2	-	3
113.0053	Cu II	0	3.26 -	14.23	4s 1D	-	4p 5F	2	-	1
113.0774	Cu II	0	2.72 -	13.68	4s 3D	-	4p 5D	3	-	2
113.0885	Cu II	1	2.98 -	13.94	4s 3D	-	4p 5G	1	-	2
113.2792	Cu II	0	2.83 -	13.78	4s 3D	-	4p 5D	2	-	1
113.3567	Cu II	0	3.26 -	14.19	4s 1D	-	4p 5F	2	-	2
113.9785	Cu II	0	3.26 -	14.13	4s 1D	-	4p 5F	2	-	3
114.2640	Cu II	3	2.83 -	13.68	4s 3D	-	4p 5D	2	-	2
114.4856	Cu II	4	2.72 -	13.55	4s 3D	-	4p 5D	3	-	3
114.7760	Cu II	1	2.98 -	13.78	4s 3D	-	4p 5D	1	-	1
115.7021	Cu II	1	2.83 -	13.55	4s 3D	-	4p 5D	2	-	3
115.7872	Cu II	1	2.98 -	13.68	4s 3D	-	4p 5D	1	-	2
116.0630	Cu II	0	3.26 -	13.94	4s 1D	-	4p 5G	2	-	2
116.2601	Cu II	1	2.72 -	13.38	4s 3D	-	4p 5D	3	-	4
116.8063	Cu II	0	3.26 -	13.87	4s 1D	-	4p 5G	2	-	3
117.8411	Cu II	0	3.26 -	13.78	4s 1D	-	4p 5D	2	-	1
118.5343	Cu II	1	8.23 -	18.69	4p 3P	-	8s 3D	2	-	2
118.5899	Cu II	14	8.23 -	18.69	4p 3P	-	8s 3D	2	-	3
118.9073	Cu II	0	3.26 -	13.68	4s 1D	-	4p 5D	2	-	2
120.4653	Cu II	0	3.26 -	13.55	4s 1D	-	4p 5D	2	-	3
120.6769	Cu II	5	8.42 -	18.69	4p 3P	-	8s 3D	1	-	2
121.4555	Cu II	17	8.49 -	18.69	4p 3F	-	8s 3D	3	-	2
121.5138	Cu II	4	8.49 -	18.69	4p 3F	-	8s 3D	3	-	3
121.6193	Cu II	1	8.23 -	18.43	4p 3P	-	6d 3F	2	-	2
121.6361	Cu II	5	8.23 -	18.43	4p 3P	-	6d 1F	2	-	3
121.7415	Cu II	2	8.23 -	18.42	4p 3P	-	6d 1D	2	-	2
121.7984	Cu II	1	8.23 -	18.41	4p 3P	-	6d 3D	2	-	1
121.8575	Cu II	0	8.23 -	18.41	4p 3P	-	6d 3G	2	-	3
121.9334	Cu II	27	8.52 -	18.69	4p 3F	-	8s 3D	4	-	3
122.0363	Cu II	0	8.23 -	18.39	4p 3P	-	6d 1P	2	-	1
123.0223	Cu II	4	8.42 -	18.50	4p 3P	-	6d 1S	1	-	0
123.5873	Cu II	4	8.66 -	18.69	4p 3F	-	8s 3D	2	-	2
123.6477	Cu II	1	8.66 -	18.69	4p 3F	-	8s 3D	2	-	3
123.8759	Cu II	1	8.42 -	18.43	4p 3P	-	6d 3F	1	-	2
124.0027	Cu II	26	8.42 -	18.42	4p 3P	-	6d 1D	1	-	2
124.0618	Cu II	2	8.42 -	18.41	4p 3P	-	6d 3D	1	-	1
124.1964	Cu II	2	8.23 -	18.22	4p 3P	-	7s 1D	2	-	2

124.2738	Cu II	1	8.23 -	18.21	4p 3P	-	7s 3D	2	-	1
124.3086	Cu II	20	8.42 -	18.39	4p 3P	-	6d 1P	1	-	1
124.6965	Cu II	1	8.49 -	18.43	4p 3F	-	6d 3F	3	-	2
124.7142	Cu II	3	8.49 -	18.43	4p 3F	-	6d 1F	3	-	3
124.7561	Cu II	2	8.23 -	18.17	4p 3P	-	6d 3D	2	-	2
124.7852	Cu II	1	8.23 -	18.17	4p 3P	-	6d 3F	2	-	3
124.8249	Cu II	0	8.49 -	18.42	4p 3F	-	6d 1D	3	-	2
124.8792	Cu II	43	8.23 -	18.16	4p 3P	-	6d 3D	2	-	3
124.8942	Cu II	9	8.49 -	18.41	4p 3F	-	6d 1G	3	-	4
124.9469	Cu II	1	8.49 -	18.41	4p 3F	-	6d 3G	3	-	3
124.9856	Cu II	7	8.23 -	18.15	4p 3P	-	6d 3P	2	-	1
125.0048	Cu II	66	8.23 -	18.15	4p 3P	-	6d 3P	2	-	2
125.0923	Cu II	4	8.78 -	18.69	4p 3D	-	8s 3D	3	-	2
125.1542	Cu II	7	8.78 -	18.69	4p 3D	-	8s 3D	3	-	3
125.3181	Cu II	53	8.23 -	18.13	4p 3P	-	6d 3S	2	-	1
125.3374	Cu II	0	8.52 -	18.41	4p 3F	-	6d 1G	4	-	4
125.5157	Cu II	22	8.54 -	18.41	4p 3P	-	6d 3D	0	-	1
125.7683	Cu II	14	8.54 -	18.39	4p 3P	-	6d 1P	0	-	1
126.1215	Cu II	11	8.86 -	18.69	4p 3D	-	8s 3D	2	-	2
126.1845	Cu II	1	8.86 -	18.69	4p 3D	-	8s 3D	2	-	3
126.2925	Cu II	15	8.42 -	18.24	4p 3P	-	6d 3P	1	-	0
126.5506	Cu II	8	8.42 -	18.22	4p 3P	-	7s 1D	1	-	2
126.6310	Cu II	3	8.42 -	18.21	4p 3P	-	7s 3D	1	-	1
126.8032	Cu II	1	8.92 -	18.69	4p 1F	-	8s 3D	3	-	2
126.8669	Cu II	10	8.92 -	18.69	4p 1F	-	8s 3D	3	-	3
126.9446	Cu II	23	8.66 -	18.43	4p 3F	-	6d 3F	2	-	2
126.9630	Cu II	1	8.66 -	18.43	4p 3F	-	6d 1F	2	-	3
127.0352	Cu II	4	8.87 -	18.63	4s2 3F	-	4p 3F	3	-	2
127.0778	Cu II	3	8.66 -	18.42	4p 3F	-	6d 1D	2	-	2
127.1318	Cu II	18	8.42 -	18.17	4p 3P	-	6d 3D	1	-	2
127.1398	Cu II	3	8.66 -	18.41	4p 3F	-	6d 3D	2	-	1
127.2042	Cu II	102	8.66 -	18.41	4p 3F	-	6d 3G	2	-	3
127.3701	Cu II	18	8.42 -	18.15	4p 3P	-	6d 3P	1	-	1
127.3990	Cu II	1	8.66 -	18.39	4p 3F	-	6d 1P	2	-	1
127.4071	Cu II	2	8.49 -	18.22	4p 3F	-	7s 1D	3	-	2
127.4465	Cu II	2	8.23 -	17.96	4p 3P	-	7s 3D	2	-	2
127.5572	Cu II	30	8.23 -	17.95	4p 3P	-	7s 3D	2	-	3
127.7154	Cu II	6	8.42 -	18.13	4p 3P	-	6d 3S	1	-	1
127.9961	Cu II	6	8.49 -	18.17	4p 3F	-	6d 3D	3	-	2
128.0214	Cu II	1	8.49 -	18.17	4p 3F	-	6d 3F	3	-	4
128.0268	Cu II	47	8.49 -	18.17	4p 3F	-	6d 3F	3	-	3
128.1257	Cu II	7	8.49 -	18.16	4p 3F	-	6d 3D	3	-	3

128.1462	Cu II	7	8.54 -	18.21	4p 3P	-	7s 3D	0	-	1
128.2455	Cu II	165	8.49 -	18.15	4p 3F	-	6d 3G	3	-	4
128.2580	Cu II	6	8.49 -	18.15	4p 3F	-	6d 3P	3	-	2
128.4871	Cu II	59	8.52 -	18.17	4p 3F	-	6d 3F	4	-	4
128.4926	Cu II	1	8.52 -	18.17	4p 3F	-	6d 3F	4	-	3
128.5330	Cu II	2	8.78 -	18.43	4p 3D	-	6d 3F	3	-	2
128.5519	Cu II	20	8.78 -	18.43	4p 3D	-	6d 1F	3	-	3
128.5922	Cu II	13	8.52 -	18.16	4p 3F	-	6d 3D	4	-	3
128.6695	Cu II	2	8.78 -	18.42	4p 3D	-	6d 1D	3	-	2
128.7129	Cu II	12	8.52 -	18.15	4p 3F	-	6d 3G	4	-	4
128.7326	Cu II	2	9.06 -	18.69	4p 3D	-	8s 3D	1	-	2
128.7468	Cu II	84	8.78 -	18.41	4p 3D	-	6d 1G	3	-	4
128.7468	Cu II	226	8.52 -	18.15	4p 3F	-	6d 3G	4	-	5
128.7991	Cu II	5	8.78 -	18.41	4p 3D	-	6d 3G	3	-	3
128.9889	Cu II	4	9.02 -	18.63	4s2 3F	-	4p 3F	2	-	2
129.1503	Cu II	0	9.09 -	18.69	4p 1D	-	8s 3D	2	-	2
129.2163	Cu II	1	9.09 -	18.69	4p 1D	-	8s 3D	2	-	3
129.2568	Cu II	1	8.54 -	18.13	4p 3P	-	6d 3S	0	-	1
129.5561	Cu II	4	9.12 -	18.69	4p 1P	-	8s 3D	1	-	2
129.6199	Cu II	7	8.86 -	18.43	4p 3D	-	6d 3F	2	-	2
129.6391	Cu II	1	8.86 -	18.43	4p 3D	-	6d 1F	2	-	3
129.7550	Cu II	4	8.66 -	18.22	4p 3F	-	7s 1D	2	-	2
129.7587	Cu II	1	8.86 -	18.42	4p 3D	-	6d 1D	2	-	2
129.8234	Cu II	1	8.86 -	18.41	4p 3D	-	6d 3D	2	-	1
129.8395	Cu II	20	8.66 -	18.21	4p 3F	-	7s 3D	2	-	1
129.8905	Cu II	35	8.86 -	18.41	4p 3D	-	6d 3G	2	-	3
129.9268	Cu II	9	8.42 -	17.96	4p 3P	-	7s 3D	1	-	2
130.0937	Cu II	0	8.86 -	18.39	4p 3D	-	6d 1P	2	-	1
130.3401	Cu II	2	8.92 -	18.43	4p 1F	-	6d 3F	3	-	2
130.3594	Cu II	18	8.92 -	18.43	4p 1F	-	6d 1F	3	-	3
130.3660	Cu II	14	8.66 -	18.17	4p 3F	-	6d 3D	2	-	2
130.3978	Cu II	25	8.66 -	18.17	4p 3F	-	6d 3F	2	-	3
130.4804	Cu II	2	8.92 -	18.42	4p 1F	-	6d 1D	3	-	2
130.5004	Cu II	0	8.66 -	18.16	4p 3F	-	6d 3D	2	-	3
130.5561	Cu II	92	8.92 -	18.41	4p 1F	-	6d 1G	3	-	4
130.6137	Cu II	5	8.92 -	18.41	4p 1F	-	6d 3G	3	-	3
130.6166	Cu II	5	8.66 -	18.15	4p 3F	-	6d 3P	2	-	1
130.6376	Cu II	1	8.66 -	18.15	4p 3F	-	6d 3P	2	-	2
130.8297	Cu II	34	8.49 -	17.96	4p 3F	-	7s 3D	3	-	2
130.9463	Cu II	8	8.49 -	17.95	4p 3F	-	7s 3D	3	-	3
131.4149	Cu II	21	8.78 -	18.22	4p 3D	-	7s 1D	3	-	2
131.4337	Cu II	56	8.52 -	17.95	4p 3F	-	7s 3D	4	-	3

132.0418	Cu II	2	8.78 -	18.17	4p 3D	-	6d 3D	3	-	2
132.0686	Cu II	69	8.78 -	18.17	4p 3D	-	6d 3F	3	-	4
132.0744	Cu II	4	8.78 -	18.17	4p 3D	-	6d 3F	3	-	3
132.1796	Cu II	35	8.78 -	18.16	4p 3D	-	6d 3D	3	-	3
132.2633	Cu II	17	9.12 -	18.50	4p 1P	-	6d 1S	1	-	0
132.3071	Cu II	8	8.78 -	18.15	4p 3D	-	6d 3G	3	-	4
132.3204	Cu II	12	8.78 -	18.15	4p 3D	-	6d 3P	3	-	2
132.3794	Cu II	32	9.06 -	18.43	4p 3D	-	6d 3F	1	-	2
132.5242	Cu II	31	9.06 -	18.42	4p 3D	-	6d 1D	1	-	2
132.5513	Cu II	2	8.86 -	18.22	4p 3D	-	7s 1D	2	-	2
132.5917	Cu II	17	9.06 -	18.41	4p 3D	-	6d 3D	1	-	1
132.6395	Cu II	7	8.86 -	18.21	4p 3D	-	7s 3D	2	-	1
132.8212	Cu II	16	9.09 -	18.43	4p 1D	-	6d 3F	2	-	2
132.8413	Cu II	96	9.09 -	18.43	4p 1D	-	6d 1F	2	-	3
132.8736	Cu II	6	9.06 -	18.39	4p 3D	-	6d 1P	1	-	1
132.9670	Cu II	35	9.09 -	18.42	4p 1D	-	6d 1D	2	-	2
133.0349	Cu II	4	9.09 -	18.41	4p 1D	-	6d 3D	2	-	1
133.1053	Cu II	1	9.09 -	18.41	4p 1D	-	6d 3G	2	-	3
133.1891	Cu II	44	8.86 -	18.17	4p 3D	-	6d 3D	2	-	2
133.2223	Cu II	67	8.86 -	18.17	4p 3D	-	6d 3F	2	-	3
133.2504	Cu II	20	9.12 -	18.43	4p 1P	-	6d 3F	1	-	2
133.3045	Cu II	21	8.92 -	18.22	4p 1F	-	7s 1D	3	-	2
133.3067	Cu II	7	8.66 -	17.96	4p 3F	-	7s 3D	2	-	2
133.3187	Cu II	10	9.09 -	18.39	4p 1D	-	6d 1P	2	-	1
133.3293	Cu II	1	8.86 -	18.16	4p 3D	-	6d 3D	2	-	3
133.3971	Cu II	4	9.12 -	18.42	4p 1P	-	6d 1D	1	-	2
133.4278	Cu II	1	8.66 -	17.95	4p 3F	-	7s 3D	2	-	3
133.4506	Cu II	13	8.86 -	18.15	4p 3D	-	6d 3P	2	-	1
133.4655	Cu II	16	9.12 -	18.41	4p 1P	-	6d 3D	1	-	1
133.4726	Cu II	2	8.86 -	18.15	4p 3D	-	6d 3P	2	-	2
133.7511	Cu II	13	9.12 -	18.39	4p 1P	-	6d 1P	1	-	1
133.8298	Cu II	0	8.86 -	18.13	4p 3D	-	6d 3S	2	-	1
133.9495	Cu II	1	8.92 -	18.17	4p 1F	-	6d 3D	3	-	2
133.9771	Cu II	55	8.92 -	18.17	4p 1F	-	6d 3F	3	-	4
133.9831	Cu II	1	8.92 -	18.17	4p 1F	-	6d 3F	3	-	3
134.0914	Cu II	45	8.92 -	18.16	4p 1F	-	6d 3D	3	-	3
134.2226	Cu II	2	8.92 -	18.15	4p 1F	-	6d 3G	3	-	4
134.2363	Cu II	17	8.92 -	18.15	4p 1F	-	6d 3P	3	-	2
135.0594	Cu II	8	8.78 -	17.96	4p 3D	-	7s 3D	3	-	2
135.1428	Cu II	4	9.06 -	18.24	4p 3D	-	6d 3P	1	-	0
135.1837	Cu II	15	8.78 -	17.95	4p 3D	-	7s 3D	3	-	3

135.4384	Cu II	1	9.06 -	18.22	4p 3D	-	7s 1D	1	-	2
135.5305	Cu II	15	9.06 -	18.21	4p 3D	-	7s 3D	1	-	1
135.8773	Cu II	540	0.00 -	9.12	3d10 1S	-	4p 1P	0	-	1
135.9009	Cu II	24	9.09 -	18.22	4p 1D	-	7s 1D	2	-	2
135.9936	Cu II	5	9.09 -	18.21	4p 1D	-	7s 3D	2	-	1
136.0507	Cu II	3	9.12 -	18.24	4p 1P	-	6d 3P	1	-	0
136.1043	Cu II	6	9.06 -	18.17	4p 3D	-	6d 3D	1	-	2
136.2600	Cu II	22	8.86 -	17.96	4p 3D	-	7s 3D	2	-	2
136.3503	Cu II	11	9.12 -	18.22	4p 1P	-	7s 1D	1	-	2
136.3775	Cu II	7	9.06 -	18.15	4p 3D	-	6d 3P	1	-	1
136.3865	Cu II	1	8.86 -	17.95	4p 3D	-	7s 3D	2	-	3
136.4436	Cu II	1	9.12 -	18.21	4p 1P	-	7s 3D	1	-	1
136.5714	Cu II	0	9.09 -	18.17	4p 1D	-	6d 3D	2	-	2
136.6063	Cu II	0	9.09 -	18.17	4p 1D	-	6d 3F	2	-	3
136.7188	Cu II	2	9.09 -	18.16	4p 1D	-	6d 3D	2	-	3
136.7735	Cu II	1	9.06 -	18.13	4p 3D	-	6d 3S	1	-	1
136.7951	Cu II	34	0.00 -	9.06	3d10 1S	-	4p 3D	0	-	1
136.8464	Cu II	1	9.09 -	18.15	4p 1D	-	6d 3P	2	-	1
136.8695	Cu II	3	9.09 -	18.15	4p 1D	-	6d 3P	2	-	2
137.0252	Cu II	13	9.12 -	18.17	4p 1P	-	6d 3D	1	-	2
137.0560	Cu II	2	8.92 -	17.96	4p 1F	-	7s 3D	3	-	2
137.1840	Cu II	21	8.92 -	17.95	4p 1F	-	7s 3D	3	-	3
137.2451	Cu II	2	9.09 -	18.13	4p 1D	-	6d 3S	2	-	1
137.3021	Cu II	14	9.12 -	18.15	4p 1P	-	6d 3P	1	-	1
137.5074	Cu II	3	8.23 -	17.25	4p 3P	-	5d 3F	2	-	2
137.5502	Cu II	13	8.23 -	17.25	4p 3P	-	5d 1F	2	-	3
137.6031	Cu II	4	8.42 -	17.43	4p 3P	-	5d 1S	1	-	0
137.7035	Cu II	3	9.12 -	18.13	4p 1P	-	6d 3S	1	-	1
137.8122	Cu II	4	8.23 -	17.23	4p 3P	-	5d 1D	2	-	2
137.9698	Cu II	1	8.23 -	17.22	4p 3P	-	5d 3D	2	-	1
138.1208	Cu II	0	8.23 -	17.21	4p 3P	-	5d 3G	2	-	3
138.5534	Cu II	1	8.23 -	17.18	4p 3P	-	5d 1P	2	-	1
139.3128	Cu II	3	9.06 -	17.96	4p 3D	-	7s 3D	1	-	2
139.8021	Cu II	0	9.09 -	17.96	4p 1D	-	7s 3D	2	-	2
139.8642	Cu II	62	8.64 -	17.51	4s2 3F	-	6p 1F	4	-	3
139.9353	Cu II	2	9.09 -	17.95	4p 1D	-	7s 3D	2	-	3
140.2777	Cu II	7	9.12 -	17.96	4p 1P	-	7s 3D	1	-	2
140.3992	Cu II	2	8.42 -	17.25	4p 3P	-	5d 3F	1	-	2
140.7169	Cu II	75	8.42 -	17.23	4p 3P	-	5d 1D	1	-	2
140.8812	Cu II	8	8.42 -	17.22	4p 3P	-	5d 3D	1	-	1
141.4541	Cu II	1	8.49 -	17.25	4p 3F	-	5d 3F	3	-	2
141.4898	Cu II	58	8.42 -	17.18	4p 3P	-	5d 1P	1	-	1

141.4994	Cu II	8	8.49 -	17.25	4p 3F	-	5d 1F	3	-	3
141.5329	Cu II	0	8.23 -	17.00	4p 3P	-	5d 3D	2	-	2
141.6108	Cu II	1	8.23 -	16.99	4p 3P	-	5d 3F	2	-	3
141.7766	Cu II	1	8.49 -	17.23	4p 3F	-	5d 1D	3	-	2
141.8426	Cu II	137	8.23 -	16.98	4p 3P	-	5d 3D	2	-	3
141.9746	Cu II	24	8.49 -	17.22	4p 3F	-	5d 1G	3	-	4
142.0686	Cu II	0	8.52 -	17.25	4p 3F	-	5d 1F	4	-	3
142.1033	Cu II	0	8.49 -	17.21	4p 3F	-	5d 3G	3	-	3
142.1374	Cu II	25	8.23 -	16.96	4p 3P	-	5d 3P	2	-	1
142.1759	Cu II	215	8.23 -	16.96	4p 3P	-	5d 3P	2	-	2
142.4448	Cu II	154	8.87 -	17.57	4s2 3F	-	6p 3F	3	-	2
142.5476	Cu II	1	8.52 -	17.22	4p 3F	-	5d 1G	4	-	4
142.6774	Cu II	0	8.52 -	17.21	4p 3F	-	5d 3G	4	-	3
142.7591	Cu II	60	8.54 -	17.22	4p 3P	-	5d 3D	0	-	1
142.7829	Cu II	6	8.64 -	17.33	4s2 3F	-	6p 3D	4	-	3
142.8358	Cu II	127	8.87 -	17.55	4s2 3F	-	6p 1D	3	-	2
143.0243	Cu II	163	8.23 -	16.90	4p 3P	-	5d 3S	2	-	1
143.3840	Cu II	51	8.54 -	17.18	4p 3P	-	5d 1P	0	-	1
143.4770	Cu II	54	8.42 -	17.06	4p 3P	-	5d 3P	1	-	0
143.4904	Cu II	994	8.64 -	17.28	4s2 3F	-	6p 3F	4	-	4
143.5316	Cu II	320	8.87 -	17.51	4s2 3F	-	6p 1F	3	-	3
143.6236	Cu II	444	8.64 -	17.28	4s2 3F	-	6p 3F	4	-	3
144.2139	Cu II	4	8.23 -	16.83	4p 3P	-	6s 1D	2	-	2
144.3542	Cu II	70	8.66 -	17.25	4p 3F	-	5d 3F	2	-	2
144.4013	Cu II	2	8.66 -	17.25	4p 3F	-	5d 1F	2	-	3
144.4130	Cu II	1	8.23 -	16.82	4p 3P	-	6s 3D	2	-	1
144.5984	Cu II	61	8.42 -	17.00	4p 3P	-	5d 3D	1	-	2
144.6901	Cu II	9	8.66 -	17.23	4p 3F	-	5d 1D	2	-	2
144.8638	Cu II	11	8.66 -	17.22	4p 3F	-	5d 3D	2	-	1
144.9058	Cu II	580	9.02 -	17.57	4s2 3F	-	6p 3F	2	-	2
145.0304	Cu II	310	8.66 -	17.21	4p 3F	-	5d 3G	2	-	3
145.2294	Cu II	53	8.42 -	16.96	4p 3P	-	5d 3P	1	-	1
145.2696	Cu II	2	8.42 -	16.96	4p 3P	-	5d 3P	1	-	2
145.3104	Cu II	176	9.02 -	17.55	4s2 3F	-	6p 1D	2	-	2
145.5074	Cu II	1	8.66 -	17.18	4p 3F	-	5d 1P	2	-	1
145.5662	Cu II	41	8.64 -	17.16	4s2 3F	-	4p 3G	4	-	3
145.7176	Cu II	24	8.49 -	17.00	4p 3F	-	5d 3D	3	-	2
145.7796	Cu II	1	8.49 -	16.99	4p 3F	-	5d 3F	3	-	4
145.8002	Cu II	158	8.49 -	16.99	4p 3F	-	5d 3F	3	-	3
145.9412	Cu II	287	9.02 -	17.51	4s2 3F	-	6p 3D	2	-	1
146.0306	Cu II	49	9.02 -	17.51	4s2 3F	-	6p 1F	2	-	3
146.0459	Cu II	10	8.49 -	16.98	4p 3F	-	5d 3D	3	-	3

146.1554	Cu II	22	8.42 -	16.90	4p 3P	-	5d 3S	1	-	1
146.3752	Cu II	525	8.49 -	16.96	4p 3F	-	5d 3G	3	-	4
146.3838	Cu II	187	8.52 -	16.99	4p 3F	-	5d 3F	4	-	4
146.3993	Cu II	13	8.49 -	16.96	4p 3F	-	5d 3P	3	-	2
146.4046	Cu II	4	8.52 -	16.99	4p 3F	-	5d 3F	4	-	3
146.4117	Cu II	7	8.78 -	17.25	4p 3D	-	5d 3F	3	-	2
146.4602	Cu II	67	8.78 -	17.25	4p 3D	-	5d 1F	3	-	3
146.5036	Cu II	0	9.02 -	17.48	4s2 3F	-	6p 1P	2	-	1
146.5541	Cu II	541	8.64 -	17.10	4s2 3F	-	4p 3P	4	-	4
146.6070	Cu II	1362	8.87 -	17.33	4s2 3F	-	6p 3D	3	-	3
146.6524	Cu II	37	8.52 -	16.98	4p 3F	-	5d 3D	4	-	3
146.6728	Cu II	7	8.87 -	17.32	4s2 3F	-	6p 3D	3	-	2
146.7572	Cu II	6	8.78 -	17.23	4p 3D	-	5d 1D	3	-	2
146.9693	Cu II	254	8.78 -	17.22	4p 3D	-	5d 1G	3	-	4
146.9843	Cu II	33	8.52 -	16.96	4p 3F	-	5d 3G	4	-	4
147.0697	Cu II	712	8.52 -	16.95	4p 3F	-	5d 3G	4	-	5
147.1073	Cu II	12	8.78 -	17.21	4p 3D	-	5d 3G	3	-	3
147.2257	Cu II	0	8.54 -	16.96	4p 3P	-	5d 3P	0	-	1
147.2395	Cu II	9	0.00 -	8.42	3d10 1S	-	4p 3P	0	-	1
147.3530	Cu II	139	8.87 -	17.28	4s2 3F	-	6p 3F	3	-	4
147.3978	Cu II	20	8.42 -	16.83	4p 3P	-	6s 1D	1	-	2
147.4935	Cu II	17	8.87 -	17.28	4s2 3F	-	6p 3F	3	-	3
147.6059	Cu II	9	8.42 -	16.82	4p 3P	-	6s 3D	1	-	1
147.8236	Cu II	22	8.86 -	17.25	4p 3D	-	5d 3F	2	-	2
147.8731	Cu II	1	8.86 -	17.25	4p 3D	-	5d 1F	2	-	3
148.1544	Cu II	4	8.87 -	17.24	4s2 3F	-	4p 3D	3	-	2
148.1759	Cu II	1	8.86 -	17.23	4p 3D	-	5d 1D	2	-	2
148.1760	Cu II	1	9.06 -	17.43	4p 3D	-	5d 1S	1	-	0
148.1775	Cu II	1	8.54 -	16.90	4p 3P	-	5d 3S	0	-	1
148.3581	Cu II	3	8.86 -	17.22	4p 3D	-	5d 3D	2	-	1
148.4168	Cu II	2	8.64 -	17.00	4s2 3F	-	4p 3G	4	-	5
148.4263	Cu II	0	8.64 -	17.00	4s2 3F	-	4p 3G	4	-	3
148.5328	Cu II	120	8.86 -	17.21	4p 3D	-	5d 3G	2	-	3
148.5610	Cu II	5	8.49 -	16.83	4p 3F	-	6s 1D	3	-	2
148.5678	Cu II	4	8.23 -	16.58	4p 3P	-	6s 3D	2	-	2
148.7610	Cu II	6	8.92 -	17.25	4p 1F	-	5d 3F	3	-	2
148.7970	Cu II	43	8.66 -	17.00	4p 3F	-	5d 3D	2	-	2
148.8110	Cu II	54	8.92 -	17.25	4p 1F	-	5d 1F	3	-	3
148.8637	Cu II	85	8.23 -	16.56	4p 3P	-	6s 3D	2	-	3
148.8831	Cu II	87	8.66 -	16.99	4p 3F	-	5d 3F	2	-	3
149.0331	Cu II	0	8.86 -	17.18	4p 3D	-	5d 1P	2	-	1

149.1177	Cu II	4	8.92	-	17.23	4p 1F	-	5d 1D	3	-	2
149.1394	Cu II	2	8.66	-	16.98	4p 3F	-	5d 3D	2	-	3
149.2153	Cu II	248	9.02	-	17.33	4s2 3F	-	6p 3D	2	-	3
149.2682	Cu II	61	9.12	-	17.43	4p 1P	-	5d 1S	1	-	0
149.2834	Cu II	1	8.64	-	16.95	4s2 3F	-	4p 3G	4	-	4
149.2834	Cu II	1343	9.02	-	17.32	4s2 3F	-	6p 3D	2	-	2
149.3366	Cu II	302	8.92	-	17.22	4p 1F	-	5d 1G	3	-	4
149.4653	Cu II	13	8.66	-	16.96	4p 3F	-	5d 3P	2	-	1
149.4791	Cu II	13	8.92	-	17.21	4p 1F	-	5d 3G	3	-	3
149.5079	Cu II	1	8.66	-	16.96	4p 3F	-	5d 3P	2	-	2
149.5430	Cu II	329	8.87	-	17.16	4s2 3F	-	4p 3G	3	-	3
149.6687	Cu II	19	8.54	-	16.82	4p 3P	-	6s 3D	0	-	1
149.8417	Cu II	1	8.64	-	16.92	4s2 3F	-	4p 3D	4	-	3
150.1336	Cu II	17	9.02	-	17.28	4s2 3F	-	6p 3F	2	-	3
150.3368	Cu II	172	9.02	-	17.26	4s2 3F	-	6p 3P	2	-	1
150.4463	Cu II	0	8.66	-	16.90	4p 3F	-	5d 3S	2	-	1
150.4757	Cu II	0		-			-				
150.5388	Cu II	0		-			-				
150.5857	Cu II	111	8.87	-	17.10	4s2 3F	-	4p 3P	3	-	4
150.8185	Cu II	23	9.02	-	17.24	4s2 3F	-	4p 3D	2	-	2
150.8632	Cu II	0		-			-				
150.9840	Cu II	1	8.78	-	17.00	4p 3D	-	5d 3D	3	-	2
151.0506	Cu II	225	8.78	-	16.99	4p 3D	-	5d 3F	3	-	4
151.0727	Cu II	4	8.78	-	16.99	4p 3D	-	5d 3F	3	-	3
151.2174	Cu II	442	8.64	-	16.84	4s2 3F	-	4p 3F	4	-	4
151.2465	Cu II	118	8.87	-	17.07	4s2 3F	-	4p 1D	3	-	2
151.3366	Cu II	111	8.78	-	16.98	4p 3D	-	5d 3D	3	-	3
151.4234	Cu II	97	9.06	-	17.25	4p 3D	-	5d 3F	1	-	2
151.4492	Cu II	1672	8.64	-	16.83	4s2 3F	-	4p 3F	4	-	3
151.6901	Cu II	25	8.78	-	16.96	4p 3D	-	5d 3G	3	-	4
151.7160	Cu II	37	8.78	-	16.96	4p 3D	-	5d 3P	3	-	2
151.7631	Cu II	10	8.66	-	16.83	4p 3F	-	6s 1D	2	-	2
151.7930	Cu II	102	9.06	-	17.23	4p 3D	-	5d 1D	1	-	2
151.9492	Cu II	27	8.42	-	16.58	4p 3P	-	6s 3D	1	-	2
151.9837	Cu II	58	8.66	-	16.82	4p 3F	-	6s 3D	2	-	1
151.9843	Cu II	49	9.06	-	17.22	4p 3D	-	5d 3D	1	-	1
152.0017	Cu II	51	9.09	-	17.25	4p 1D	-	5d 3F	2	-	2
152.0540	Cu II	302	9.09	-	17.25	4p 1D	-	5d 1F	2	-	3
152.2577	Cu II	88	9.02	-	17.16	4s2 3F	-	4p 3G	2	-	3
152.3741	Cu II	110	9.09	-	17.23	4p 1D	-	5d 1D	2	-	2
152.4860	Cu II	141	8.86	-	17.00	4p 3D	-	5d 3D	2	-	2
152.5631	Cu II	78	8.87	-	17.00	4s2 3F	-	4p 3G	3	-	3

152.5641	Cu II	64	9.12	-	17.25	4p 1P	-	5d 3F	1	-	2
152.5764	Cu II	13	9.09	-	17.22	4p 1D	-	5d 3D	2	-	1
152.5764	Cu II	191	8.86	-	16.99	4p 3D	-	5d 3F	2	-	3
152.6928	Cu II	21	9.06	-	17.18	4p 3D	-	5d 1P	1	-	1
152.7516	Cu II	3	9.09	-	17.21	4p 1D	-	5d 3G	2	-	3
152.8456	Cu II	9	8.86	-	16.98	4p 3D	-	5d 3D	2	-	3
152.9393	Cu II	13	9.12	-	17.23	4p 1P	-	5d 1D	1	-	2
153.1335	Cu II	51	9.12	-	17.22	4p 1P	-	5d 3D	1	-	1
153.1856	Cu II	100	8.49	-	16.58	4p 3F	-	6s 3D	3	-	2
153.1879	Cu II	39	8.86	-	16.96	4p 3D	-	5d 3P	2	-	1
153.2131	Cu II	1726	8.87	-	16.96	4s2 3F	-	4p 3F	3	-	2
153.2326	Cu II	1	8.86	-	16.96	4p 3D	-	5d 3P	2	-	2
153.2808	Cu II	29	9.09	-	17.18	4p 1D	-	5d 1P	2	-	1
153.3986	Cu II	1245	9.02	-	17.10	4s2 3F	-	4p 3S	2	-	1
153.4627	Cu II	16	8.87	-	16.95	4s2 3F	-	4p 3G	3	-	4
153.4836	Cu II	1	8.92	-	17.00	4p 1F	-	5d 3D	3	-	2
153.5002	Cu II	21	8.49	-	16.56	4p 3F	-	6s 3D	3	-	3
153.5524	Cu II	159	8.92	-	16.99	4p 1F	-	5d 3F	3	-	4
153.5752	Cu II	3	8.92	-	16.99	4p 1F	-	5d 3F	3	-	3
153.7559	Cu II	4765	8.64	-	16.71	4s2 3F	-	4p 3H	4	-	4
153.8480	Cu II	141	8.92	-	16.98	4p 1F	-	5d 3D	3	-	3
153.8527	Cu II	37	9.12	-	17.18	4p 1P	-	5d 1P	1	-	1
154.0239	Cu II	451	9.02	-	17.07	4s2 3F	-	4p 1D	2	-	2
154.0389	Cu II	62	8.78	-	16.83	4p 3D	-	6s 1D	3	-	2
154.0588	Cu II	3098	8.87	-	16.92	4s2 3F	-	4p 3D	3	-	3
154.1703	Cu II	160	8.52	-	16.56	4p 3F	-	6s 3D	4	-	3
154.1756	Cu II	3266	10.59	-	18.63	4s2 1D	-	4p 3F	2	-	2
154.2133	Cu II	2	8.92	-	16.96	4p 1F	-	5d 3G	3	-	4
154.2186	Cu II	1	8.86	-	16.90	4p 3D	-	5d 3S	2	-	1
154.2401	Cu II	53	8.92	-	16.96	4p 1F	-	5d 3P	3	-	2
154.4677	Cu II	0		-			-			-	
154.7958	Cu II	0		-			-			-	
155.0097	Cu II	11	9.06	-	17.06	4p 3D	-	5d 3P	1	-	0
155.0653	Cu II	1	9.02	-	17.01	4s2 3F	-	4p 1P	2	-	1
155.1389	Cu II	0		-			-			-	
155.2296	Cu II	1	8.87	-	16.86	4s2 3F	-	4p 5S	3	-	2
155.2646	Cu II	8073	8.64	-	16.63	4s2 3F	-	4p 3H	4	-	5
155.3896	Cu II	4093	9.02	-	17.00	4s2 3F	-	4p 3G	2	-	3
155.5134	Cu II	5396	8.87	-	16.84	4s2 3F	-	4p 3F	3	-	4
155.5703	Cu II	2153	8.64	-	16.61	4s2 3F	-	4p 3D	4	-	3
155.6026	Cu II	5	8.86	-	16.83	4p 3D	-	6s 1D	2	-	2
155.7587	Cu II	5	8.87	-	16.83	4s2 3F	-	4p 3F	3	-	3

155.8345	Cu II	18	8.86	-	16.82	4p 3D	-	6s 3D	2	-	1
156.0639	Cu II	47	9.02	-	16.96	4s2 3F	-	4p 3F	2	-	2
156.2053	Cu II	2	9.12	-	17.06	4p 1P	-	5d 3P	1	-	0
156.3194	Cu II	14	9.06	-	17.00	4p 3D	-	5d 3D	1	-	2
156.5924	Cu II	21	8.66	-	16.58	4p 3F	-	6s 3D	2	-	2
156.6415	Cu II	58	8.92	-	16.83	4p 1F	-	6s 1D	3	-	2
156.9212	Cu II	1	8.66	-	16.56	4p 3F	-	6s 3D	2	-	3
156.9416	Cu II	1	9.09	-	17.00	4p 1D	-	5d 3D	2	-	2
156.9416	Cu II	643	9.02	-	16.92	4s2 3F	-	4p 3D	2	-	3
157.0315	Cu II	1	9.09	-	16.99	4p 1D	-	5d 3F	2	-	3
157.0571	Cu II	17	9.06	-	16.96	4p 3D	-	5d 3P	1	-	1
157.1041	Cu II	1	9.06	-	16.96	4p 3D	-	5d 3P	1	-	2
157.3167	Cu II	4	9.09	-	16.98	4p 1D	-	5d 3D	2	-	3
157.5353	Cu II	35	9.12	-	17.00	4p 1P	-	5d 3D	1	-	2
157.6793	Cu II	1	9.09	-	16.96	4p 1D	-	5d 3P	2	-	1
157.7267	Cu II	7	9.09	-	16.96	4p 1D	-	5d 3P	2	-	2
157.9492	Cu II	0		-		-		-			
158.0626	Cu II	0		-		-		-			
158.1407	Cu II	3	9.06	-	16.90	4p 3D	-	5d 3S	1	-	1
158.1567	Cu II	1	9.02	-	16.86	4s2 3F	-	4p 5S	2	-	2
158.1995	Cu II	929	8.87	-	16.71	4s2 3F	-	4p 3H	3	-	4
158.2846	Cu II	44	9.12	-	16.96	4p 1P	-	5d 3P	1	-	1
158.3324	Cu II	1	9.12	-	16.96	4p 1P	-	5d 3P	1	-	2
158.3682	Cu II	313	8.87	-	16.70	4s2 3F	-	4p 3D	3	-	2
158.7060	Cu II	3	9.02	-	16.83	4s2 3F	-	4p 3F	2	-	3
158.7715	Cu II	5	9.09	-	16.90	4p 1D	-	5d 3S	2	-	1
159.0165	Cu II	21	8.78	-	16.58	4p 3D	-	6s 3D	3	-	2
159.3556	Cu II	41	8.78	-	16.56	4p 3D	-	6s 3D	3	-	3
159.3852	Cu II	7	9.12	-	16.90	4p 1P	-	5d 3S	1	-	1
159.5963	Cu II	1	9.06	-	16.83	4p 3D	-	6s 1D	1	-	2
159.8402	Cu II	42	9.06	-	16.82	4p 3D	-	6s 3D	1	-	1
160.1210	Cu II	14	8.87	-	16.61	4s2 3F	-	4p 3D	3	-	3
160.2273	Cu II	63	9.02	-	16.75	4s2 3F	-	4p 3D	2	-	1
160.2388	Cu II	69	9.09	-	16.83	4p 1D	-	6s 1D	2	-	2
160.4848	Cu II	14	9.09	-	16.82	4p 1D	-	6s 3D	2	-	1
160.5281	Cu II	321	8.87	-	16.59	4s2 3F	-	4p 3P	3	-	2
160.6834	Cu II	62	8.86	-	16.58	4p 3D	-	6s 3D	2	-	2
160.8639	Cu II	33	9.12	-	16.83	4p 1P	-	6s 1D	1	-	2
161.0296	Cu II	2	8.86	-	16.56	4p 3D	-	6s 3D	2	-	3
161.1118	Cu II	2	9.12	-	16.82	4p 1P	-	6s 3D	1	-	1
161.4161	Cu II	7	9.02	-	16.70	4s2 3F	-	4p 3D	2	-	2

161.5825	Cu II	162	10.96	-	18.63	4s2 3P	-	4p 3F	2	-	2
161.7915	Cu II	4	8.92	-	16.58	4p 1F	-	6s 3D	3	-	2
162.1426	Cu II	59	8.92	-	16.56	4p 1F	-	6s 3D	3	-	3
162.2197	Cu II	164	10.99	-	18.63	4s2 3P	-	4p 3F	1	-	2
162.2428	Cu II	249	9.02	-	16.66	4s2 3F	-	4p 3P	2	-	1
162.3173	Cu II	23	8.64	-	16.28	4s2 3F	-	4p 5D	4	-	3
163.0268	Cu II	0		-		-		-			
163.2373	Cu II	1	9.02	-	16.61	4s2 3F	-	4p 3D	2	-	3
163.6605	Cu II	19	9.02	-	16.59	4s2 3F	-	4p 3P	2	-	2
164.9458	Cu II	9	9.06	-	16.58	4p 3D	-	6s 3D	1	-	2
165.6322	Cu II	0	9.09	-	16.58	4p 1D	-	6s 3D	2	-	2
166.0001	Cu II	4	9.09	-	16.56	4p 1D	-	6s 3D	2	-	3
166.3002	Cu II	17	9.12	-	16.58	4p 1P	-	6s 3D	1	-	2
167.2776	Cu II	1	8.87	-	16.28	4s2 3F	-	4p 5D	3	-	3
168.3155	Cu II	516	8.64	-	16.01	4s2 3F	-	4p 3D	4	-	3
168.3159	Cu II	14	8.87	-	16.24	4s2 3F	-	4p 5D	3	-	2
169.2811	Cu II	2	8.64	-	15.97	4s2 3F	-	4p 3F	4	-	4
169.9095	Cu II	16	8.87	-	16.17	4s2 3F	-	4p 3P	3	-	2
169.9102	Cu II	31	8.64	-	15.94	4s2 3F	-	4p 3F	4	-	3
170.6817	Cu II	1	9.02	-	16.28	4s2 3F	-	4p 5D	2	-	3
171.7721	Cu II	1	9.02	-	16.24	4s2 3F	-	4p 5D	2	-	2
171.7721	Cu II	4	9.02	-	16.23	4s2 3F	-	4p 5D	2	-	1
173.4227	Cu II	2	9.02	-	16.17	4s2 3F	-	4p 3P	2	-	2
173.6551	Cu II	36	8.87	-	16.01	4s2 3F	-	4p 3D	3	-	3
174.4516	Cu II	173	8.87	-	15.98	4s2 3F	-	4p 3D	3	-	2
174.6832	Cu II	1	8.87	-	15.97	4s2 3F	-	4p 3F	3	-	4
175.3281	Cu II	27	8.87	-	15.94	4s2 3F	-	4p 3D	3	-	2
175.3281	Cu II	28	9.02	-	16.09	4s2 3F	-	4p 3P	2	-	1
175.3532	Cu II	1	8.87	-	15.94	4s2 3F	-	4p 3F	3	-	3
177.3266	Cu II	2	9.02	-	16.01	4s2 3F	-	4p 3D	2	-	3
177.4768	Cu II	2	10.59	-	17.57	4s2 1D	-	6p 3F	2	-	2
178.0842	Cu II	10	10.59	-	17.55	4s2 1D	-	6p 1D	2	-	2
178.1572	Cu II	20	9.02	-	15.98	4s2 3F	-	4p 3D	2	-	2
179.0324	Cu II	1	10.59	-	17.51	4s2 1D	-	6p 3D	2	-	1
179.0660	Cu II	1	9.02	-	15.94	4s2 3F	-	4p 3D	2	-	2
179.0976	Cu II	1	9.02	-	15.94	4s2 3F	-	4p 3F	2	-	3
179.1670	Cu II	3	10.59	-	17.51	4s2 1D	-	6p 1F	2	-	3
179.8796	Cu II	3	10.59	-	17.48	4s2 1D	-	6p 1P	2	-	1
180.0978	Cu II	23	8.64	-	15.53	4s2 3F	-	4p 5P	4	-	3
183.4883	Cu II	1	8.42	-	15.18	4p 3P	-	4d 1S	1	-	0
183.9848	Cu II	3	10.59	-	17.33	4s2 1D	-	6p 3D	2	-	3
184.0884	Cu II	1	10.59	-	17.32	4s2 1D	-	6p 3D	2	-	2

185.3830	Cu II	1	10.59	-	17.28	4s2 1D	-	6p 3F	2	-	3
185.6929	Cu II	20	10.59	-	17.26	4s2 1D	-	6p 3P	2	-	1
186.1623	Cu II	6	8.87	-	15.53	4s2 3F	-	4p 5P	3	-	2
186.2248	Cu II	2	8.87	-	15.53	4s2 3F	-	4p 5P	3	-	3
186.4283	Cu II	23	10.59	-	17.24	4s2 1D	-	4p 3D	2	-	2
187.5746	Cu II	3	8.64	-	15.25	4s2 3F	-	5p 1F	4	-	3
188.0406	Cu II	4	10.96	-	17.55	4s2 3P	-	6p 1D	2	-	2
188.2208	Cu II	0	10.99	-	17.57	4s2 3P	-	6p 3F	1	-	2
188.6324	Cu II	2	10.59	-	17.16	4s2 1D	-	4p 3G	2	-	3
188.9041	Cu II	6	10.99	-	17.55	4s2 3P	-	6p 1D	1	-	2
189.2309	Cu II	1	9.02	-	15.57	4s2 3F	-	4p 5P	2	-	1
189.2483	Cu II	1	10.96	-	17.51	4s2 3P	-	6p 1F	2	-	3
189.9715	Cu II	1	10.99	-	17.51	4s2 3P	-	6p 3D	1	-	1
190.0436	Cu II	11	10.96	-	17.48	4s2 3P	-	6p 1P	2	-	1
190.3867	Cu II	0	10.59	-	17.10	4s2 1D	-	4p 3S	2	-	1
190.3881	Cu II	1	9.02	-	15.53	4s2 3F	-	4p 5P	2	-	2
190.4534	Cu II	0	9.02	-	15.53	4s2 3F	-	4p 5P	2	-	3
190.9256	Cu II	3	10.99	-	17.48	4s2 3P	-	6p 1P	1	-	1
191.1367	Cu II	5	11.03	-	17.51	4s2 3P	-	6p 3D	0	-	1
191.3509	Cu II	4	10.59	-	17.07	4s2 1D	-	4p 1D	2	-	2
191.8899	Cu II	9	8.23	-	14.70	4p 3P	-	4d 3F	2	-	2
192.0672	Cu II	73	8.23	-	14.69	4p 3P	-	4d 1F	2	-	3
192.1026	Cu II	4	11.03	-	17.48	4s2 3P	-	6p 1P	0	-	1
192.2143	Cu II	11	8.87	-	15.32	4s2 3F	-	5p 1D	3	-	2
192.9608	Cu II	28	10.59	-	17.01	4s2 1D	-	4p 1P	2	-	1
192.9751	Cu II	44	8.64	-	15.07	4s2 3F	-	5p 3D	4	-	3
193.2570	Cu II	14	8.23	-	14.65	4p 3P	-	4d 1D	2	-	2
193.4632	Cu II	1	10.59	-	17.00	4s2 1D	-	4p 3G	2	-	3
194.1389	Cu II	3	8.23	-	14.62	4p 3P	-	4d 3D	2	-	1
194.2303	Cu II	2	8.87	-	15.25	4s2 3F	-	5p 1F	3	-	3
194.4597	Cu II	77	2.72	-	9.09	4s 3D	-	4p 1D	3	-	2
194.5095	Cu II	4	10.59	-	16.96	4s2 1D	-	4p 3F	2	-	2
194.6317	Cu II	3	10.96	-	17.33	4s2 3P	-	6p 3D	2	-	3
194.6493	Cu II	1	8.64	-	15.01	4s2 3F	-	4p 1F	4	-	3
194.7477	Cu II	3	10.96	-	17.32	4s2 3P	-	6p 3D	2	-	2
194.8230	Cu II	2	8.23	-	14.60	4p 3P	-	4d 3G	2	-	3
195.2576	Cu II	4	8.87	-	15.22	4s2 3F	-	5p 3F	3	-	2
195.6740	Cu II	2	10.99	-	17.32	4s2 3P	-	6p 3D	1	-	2
195.7518	Cu II	7	8.64	-	14.98	4s2 3F	-	5p 3F	4	-	4
195.8747	Cu II	2	10.59	-	16.92	4s2 1D	-	4p 3D	2	-	3
196.1553	Cu II	1	8.64	-	14.96	4s2 3F	-	5p 3F	4	-	3
196.1971	Cu II	4	10.96	-	17.28	4s2 3P	-	6p 3F	2	-	3

196.5443	Cu II	1	10.96	-	17.26	4s2 3P	-	6p 3P	2	-	1
196.8012	Cu II	37	8.23	-	14.53	4p 3P	-	4d 1P	2	-	1
197.0495	Cu II	11	2.83	-	9.12	4s 3D	-	4p 1P	2	-	1
197.3683	Cu II	8	10.96	-	17.24	4s2 3P	-	4p 3D	2	-	2
197.4878	Cu II	8	10.99	-	17.26	4s2 3P	-	6p 3P	1	-	1
197.5686	Cu II	4	8.42	-	14.70	4p 3P	-	4d 3F	1	-	2
197.7027	Cu II	9	9.02	-	15.29	4s2 3F	-	5p 3D	2	-	1
197.7712	Cu II	1	10.59	-	16.86	4s2 1D	-	4p 5S	2	-	2
197.9956	Cu II	320	2.83	-	9.09	4s 3D	-	4p 1D	2	-	2
198.2149	Cu II	2	8.87	-	15.12	4s2 3F	-	5p 3D	3	-	2
198.3198	Cu II	5	10.99	-	17.24	4s2 3P	-	4p 3D	1	-	2
198.6308	Cu II	24	10.59	-	16.83	4s2 1D	-	4p 3F	2	-	3
198.7474	Cu II	0	11.03	-	17.26	4s2 3P	-	6p 3P	0	-	1
198.9855	Cu II	178	2.83	-	9.06	4s 3D	-	4p 3D	2	-	1
199.0180	Cu II	497	8.42	-	14.65	4p 3P	-	4d 1D	1	-	2
199.4260	Cu II	1	9.02	-	15.23	4s2 3F	-	5p 1P	2	-	1
199.6639	Cu II	8	8.49	-	14.70	4p 3F	-	4d 3F	3	-	2
199.8403	Cu II	1	10.96	-	17.16	4s2 3P	-	4p 3G	2	-	3
199.8559	Cu II	74	8.49	-	14.69	4p 3F	-	4d 1F	3	-	3
199.9116	Cu II	1	9.02	-	15.22	4s2 3F	-	5p 3F	2	-	2
199.9534	Cu II	95	8.42	-	14.62	4p 3P	-	4d 3D	1	-	1
199.9698	Cu II	1	8.87	-	15.07	4s2 3F	-	5p 3D	3	-	3
199.9698	Cu II	1189	2.72	-	8.92	4s 3D	-	4p 1F	3	-	3
200.0589	Cu II	7	8.23	-	14.43	4p 3P	-	4d 3D	2	-	2
200.3070	Cu II	3	8.23	-	14.42	4p 3P	-	4d 3F	2	-	3
200.9283	Cu II	3	8.52	-	14.69	4p 3F	-	4d 1F	4	-	3
200.9547	Cu II	7	10.59	-	16.75	4s2 1D	-	4p 3D	2	-	1
201.0795	Cu II	0	8.49	-	14.65	4p 3F	-	4d 1D	3	-	2
201.2981	Cu II	1211	8.23	-	14.39	4p 3P	-	4d 3D	2	-	3
201.5583	Cu II	31	2.98	-	9.12	4s 3D	-	4p 1P	1	-	1
201.6898	Cu II	39	2.72	-	8.86	4s 3D	-	4p 3D	3	-	2
201.7454	Cu II	1	10.96	-	17.10	4s2 3P	-	4p 3S	2	-	1
201.7610	Cu II	3	8.87	-	15.01	4s2 3F	-	4p 1F	3	-	3
202.2192	Cu II	116	8.49	-	14.62	4p 3F	-	4d 1G	3	-	4
202.5488	Cu II	260	2.98	-	9.09	4s 3D	-	4p 1D	1	-	2
202.5917	Cu II	5	8.87	-	14.99	4s2 3F	-	4p 1D	3	-	2
202.7134	Cu II	400	8.42	-	14.53	4p 3P	-	4d 1P	1	-	1
202.7172	Cu II	18	9.06	-	15.18	4p 3D	-	4d 1S	1	-	0
202.7401	Cu II	15	10.99	-	17.10	4s2 3P	-	4p 3S	1	-	1
202.7761	Cu II	31	8.49	-	14.60	4p 3F	-	4d 3G	3	-	3
202.8288	Cu II	3	10.96	-	17.07	4s2 3P	-	4p 1D	2	-	2

202.8290	Cu II	2	10.59	-	16.70	4s2 1D	-	4p 3D	2	-	2
202.8295	Cu II	0	8.64	-	14.75	4s2 3F	-	4p 1G	4	-	4
202.9463	Cu II	0	8.87	-	14.98	4s2 3F	-	5p 3F	3	-	4
202.9473	Cu II	7	9.02	-	15.12	4s2 3F	-	5p 3D	2	-	2
202.9949	Cu II	227	8.23	-	14.34	4p 3P	-	4d 3P	2	-	1
203.1036	Cu II	1863	8.23	-	14.34	4p 3P	-	4d 3P	2	-	2
203.3803	Cu II	2	8.87	-	14.96	4s2 3F	-	5p 3F	3	-	3
203.3843	Cu II	71	8.52	-	14.62	4p 3F	-	4d 1G	4	-	4
203.5854	Cu II	821	2.98	-	9.06	4s 3D	-	4p 3D	1	-	1
203.6920	Cu II	499	8.54	-	14.62	4p 3P	-	4d 3D	0	-	1
203.7127	Cu II	575	2.83	-	8.92	4s 3D	-	4p 1F	2	-	3
203.8343	Cu II	13	10.99	-	17.07	4s2 3P	-	4p 1D	1	-	2
203.9477	Cu II	3	8.52	-	14.60	4p 3F	-	4d 3G	4	-	3
204.0684	Cu II	27	11.03	-	17.10	4s2 3P	-	4p 3S	0	-	1
204.1366	Cu II	5	10.59	-	16.66	4s2 1D	-	4p 3P	2	-	1
204.3802	Cu II	788	2.72	-	8.78	4s 3D	-	4p 3D	3	-	3
204.6394	Cu II	11	10.96	-	17.01	4s2 3P	-	4p 1P	2	-	1
204.7678	Cu II	551	9.12	-	15.18	4p 1P	-	4d 1S	1	-	0
204.8480	Cu II	1	9.02	-	15.07	4s2 3F	-	5p 3D	2	-	3
205.2048	Cu II	1	10.96	-	17.00	4s2 3P	-	4p 3G	2	-	3
205.4253	Cu II	607	8.66	-	14.70	4p 3F	-	4d 3F	2	-	2
205.4418	Cu II	479	8.42	-	14.45	4p 3P	-	4d 3P	1	-	0
205.4980	Cu II	666	2.83	-	8.86	4s 3D	-	4p 3D	2	-	2
205.6285	Cu II	11	8.66	-	14.69	4p 3F	-	4d 1F	2	-	3
205.6630	Cu II	2	10.99	-	17.01	4s2 3P	-	4p 1P	1	-	1
205.7143	Cu II	5	10.59	-	16.61	4s2 1D	-	4p 3D	2	-	3
205.8614	Cu II	1	8.87	-	14.89	4s2 3F	-	5p 3P	3	-	2
206.2420	Cu II	624	8.42	-	14.43	4p 3P	-	4d 3D	1	-	2
206.3825	Cu II	2	8.64	-	14.65	4s2 3F	-	4p 3F	4	-	3
206.3830	Cu II	5	10.96	-	16.96	4s2 3P	-	4p 3F	2	-	2
206.3872	Cu II	8	10.59	-	16.59	4s2 1D	-	4p 3P	2	-	2
206.6261	Cu II	415	8.54	-	14.53	4p 3P	-	4d 1P	0	-	1
206.7364	Cu II	2	9.02	-	15.01	4s2 3F	-	4p 1F	2	-	3
206.9935	Cu II	97	8.66	-	14.65	4p 3F	-	4d 1D	2	-	2
207.0300	Cu II	0	11.03	-	17.01	4s2 3P	-	4p 1P	0	-	1
207.4210	Cu II	1	9.02	-	14.99	4s2 3F	-	5p 3P	2	-	1
207.4241	Cu II	40	10.99	-	16.96	4s2 3P	-	4p 3F	1	-	2
207.8663	Cu II	1325	8.23	-	14.20	4p 3P	-	4d 3S	2	-	1
207.9213	Cu II	4	10.96	-	16.92	4s2 3P	-	4p 3D	2	-	3
208.0060	Cu II	98	8.66	-	14.62	4p 3F	-	4d 3D	2	-	1
208.2916	Cu II	1	2.83	-	8.78	4s 3D	-	4p 3D	2	-	3
208.4324	Cu II	6	8.64	-	14.59	4s2 3F	-	4p 3F	4	-	4

208.5275	Cu II	226	8.49	-	14.43	4p 3F	-	4d 3D	3	-	2
208.5311	Cu II	19	2.72	-	8.66	4s 3D	-	4p 3F	3	-	2
208.5998	Cu II	2	8.49	-	14.43	4p 3F	-	4d 3F	3	-	4
208.7919	Cu II	2124	8.66	-	14.60	4p 3F	-	4d 3G	2	-	3
208.7971	Cu II	1325	8.49	-	14.42	4p 3F	-	4d 3F	3	-	3
209.3637	Cu II	425	8.42	-	14.34	4p 3P	-	4d 3P	1	-	1
209.4793	Cu II	57	8.42	-	14.34	4p 3P	-	4d 3P	1	-	2
209.6191	Cu II	64	8.78	-	14.70	4p 3D	-	4d 3F	3	-	2
209.8398	Cu II	652	8.78	-	14.69	4p 3D	-	4d 1F	3	-	3
209.8398	Cu II	1532	8.52	-	14.43	4p 3F	-	4d 3F	4	-	4
209.8742	Cu II	67	8.49	-	14.39	4p 3F	-	4d 3D	3	-	3
210.0394	Cu II	34	8.52	-	14.42	4p 3F	-	4d 3F	4	-	3
210.0603	Cu II	1	10.96	-	16.86	4s2 3P	-	4p 5S	2	-	2
210.4237	Cu II	2	8.87	-	14.76	4s2 3F	-	4p 3F	3	-	2
210.4797	Cu II	330	2.98	-	8.86	4s 3D	-	4p 3D	1	-	2
210.6379	Cu II	5	8.87	-	14.75	4s2 3F	-	4p 1G	3	-	4
211.0309	Cu II	53	10.96	-	16.83	4s2 3P	-	4p 3F	2	-	3
211.0436	Cu II	0	9.02	-	14.89	4s2 3F	-	5p 3P	2	-	2
211.0666	Cu II	2	8.66	-	14.53	4p 3F	-	4d 1P	2	-	1
211.1294	Cu II	307	8.52	-	14.39	4p 3F	-	4d 3D	4	-	3
211.1390	Cu II	0	10.99	-	16.86	4s2 3P	-	4p 5S	1	-	2
211.2100	Cu II	932	3.26	-	9.12	4s 1D	-	4p 1P	2	-	1
211.2522	Cu II	63	8.78	-	14.65	4p 3D	-	4d 1D	3	-	2
211.7310	Cu II	4529	8.49	-	14.34	4p 3F	-	4d 3G	3	-	4
211.8375	Cu II	77	8.49	-	14.34	4p 3F	-	4d 3P	3	-	2
212.2980	Cu II	994	3.26	-	9.09	4s 1D	-	4p 1D	2	-	2
212.5106	Cu II	1714	8.78	-	14.62	4p 3D	-	4d 1G	3	-	4
212.5267	Cu II	190	8.86	-	14.70	4p 3D	-	4d 3F	2	-	2
212.6044	Cu II	560	2.83	-	8.66	4s 3D	-	4p 3F	2	-	2
212.7443	Cu II	4	8.86	-	14.69	4p 3D	-	4d 1F	2	-	3
212.9741	Cu II	6	8.64	-	14.46	4s2 3F	-	4p 3G	4	-	3
213.0085	Cu II	271	8.52	-	14.34	4p 3F	-	4d 3G	4	-	4
213.1257	Cu II	108	8.78	-	14.60	4p 3D	-	4d 3G	3	-	3
213.2479	Cu II	10	10.99	-	16.80	4s2 3P	-	4p 3P	1	-	0
213.4341	Cu II	6012	8.52	-	14.33	4p 3F	-	4d 3G	4	-	5
213.4369	Cu II	13	3.26	-	9.06	4s 1D	-	4p 3D	2	-	1
213.5399	Cu II	4	8.54	-	14.34	4p 3P	-	4d 3P	0	-	1
213.5981	Cu II	2878	2.72	-	8.52	4s 3D	-	4p 3F	3	-	4
213.7304	Cu II	5	10.96	-	16.75	4s2 3P	-	4p 3D	2	-	1
214.1997	Cu II	14	8.64	-	14.43	4s2 3F	-	4p 3D	4	-	3
214.2057	Cu II	6	8.86	-	14.65	4p 3D	-	4d 1D	2	-	2
214.4704	Cu II	35	8.92	-	14.70	4p 1F	-	4d 3F	3	-	2

214.4722	Cu II	2	8.87	-	14.65	4s2 3F	-	4p 3F	3	-	3
214.5492	Cu II	258	8.42	-	14.20	4p 3P	-	4d 3S	1	-	1
214.6920	Cu II	379	8.92	-	14.69	4p 1F	-	4d 1F	3	-	3
214.8472	Cu II	3	10.99	-	16.75	4s2 3P	-	4p 3D	1	-	1
214.8984	Cu II	334	8.66	-	14.43	4p 3F	-	4d 3D	2	-	2
214.8984	Cu II	371	2.72	-	8.49	4s 3D	-	4p 3F	3	-	3
215.1809	Cu II	1236	8.66	-	14.42	4p 3F	-	4d 3F	2	-	3
215.2901	Cu II	10	8.86	-	14.62	4p 3D	-	4d 3D	2	-	1
215.8412	Cu II	2	9.02	-	14.76	4s2 3F	-	4p 3F	2	-	2
215.8519	Cu II	3	10.96	-	16.70	4s2 3P	-	4p 3D	2	-	2
216.1320	Cu II	1443	8.86	-	14.60	4p 3D	-	4d 3G	2	-	3
216.1803	Cu II	23	8.92	-	14.65	4p 1F	-	4d 1D	3	-	2
216.3250	Cu II	12	8.66	-	14.39	4p 3F	-	4d 3D	2	-	3
216.3394	Cu II	6	11.03	-	16.75	4s2 3P	-	4p 3D	0	-	1
216.6868	Cu II	2	8.87	-	14.59	4s2 3F	-	4p 3F	3	-	4
216.9909	Cu II	4	10.99	-	16.70	4s2 3P	-	4p 3D	1	-	2
217.3334	Cu II	3	10.96	-	16.66	4s2 3P	-	4p 3P	2	-	1
217.4982	Cu II	2972	8.92	-	14.62	4p 1F	-	4d 1G	3	-	4
217.6845	Cu II	0	10.59	-	16.28	4s2 1D	-	4p 5D	2	-	3
217.9410	Cu II	1000	2.98	-	8.66	4s 3D	-	4p 3F	1	-	2
218.0752	Cu II	15	8.64	-	14.33	4s2 3F	-	4p 3G	4	-	5
218.1426	Cu II	124	8.92	-	14.60	4p 1F	-	4d 3G	3	-	3
218.2858	Cu II	92	8.66	-	14.34	4p 3F	-	4d 3P	2	-	1
218.4115	Cu II	1	8.66	-	14.34	4p 3F	-	4d 3P	2	-	2
218.4882	Cu II	5	10.99	-	16.66	4s2 3P	-	4p 3P	1	-	1
218.5704	Cu II	2	8.86	-	14.53	4p 3D	-	4d 1P	2	-	1
218.9370	Cu II	28	8.54	-	14.20	4p 3P	-	4d 3S	0	-	1
218.9630	Cu II	564	3.26	-	8.92	4s 1D	-	4p 1F	2	-	3
219.0520	Cu II	9	11.85	-	17.51	4s2 1G	-	6p 1F	4	-	3
219.1224	Cu II	12	10.96	-	16.61	4s2 3P	-	4p 3D	2	-	3
219.2268	Cu II	1649	2.83	-	8.49	4s 3D	-	4p 3F	2	-	3
219.2355	Cu II	6	8.87	-	14.52	4s2 3F	-	4p 3D	3	-	2
219.4469	Cu II	0	10.59	-	16.24	4s2 1D	-	4p 5D	2	-	2
219.5683	Cu II	2372	8.78	-	14.43	4p 3D	-	4d 3F	3	-	4
219.7868	Cu II	13	8.78	-	14.42	4p 3D	-	4d 3F	3	-	3
219.8860	Cu II	16	10.96	-	16.59	4s2 3P	-	4p 3P	2	-	2
220.0315	Cu II	0	11.03	-	16.66	4s2 3P	-	4p 3P	0	-	1
220.0509	Cu II	854	9.06	-	14.70	4p 3D	-	4d 3F	1	-	2
220.1028	Cu II	2	9.02	-	14.65	4s2 3F	-	4p 3F	2	-	3
220.4506	Cu II	3	9.02	-	14.64	4s2 3F	-	4p 3D	2	-	1
220.9806	Cu II	860	8.78	-	14.39	4p 3D	-	4d 3D	3	-	3

221.0268	Cu II	579	3.26	-	8.86	4s 1D	-	4p 3D	2	-	2
221.0681	Cu II	0	10.99	-	16.59	4s2 3P	-	4p 3P	1	-	2
221.2748	Cu II	398	9.09	-	14.70	4p 1D	-	4d 3F	2	-	2
221.5106	Cu II	2553	9.09	-	14.69	4p 1D	-	4d 1F	2	-	3
221.5995	Cu II	0	8.87	-	14.46	4s2 3F	-	4p 3G	3	-	3
221.8108	Cu II	756	2.83	-	8.42	4s 3D	-	4p 3P	2	-	1
221.8512	Cu II	871	9.06	-	14.65	4p 3D	-	4d 1D	1	-	2
222.1648	Cu II	1	10.59	-	16.17	4s2 1D	-	4p 3P	2	-	2
222.4691	Cu II	531	9.12	-	14.70	4p 1P	-	4d 3F	1	-	2
222.6780	Cu II	1167	8.86	-	14.43	4p 3D	-	4d 3D	2	-	2
222.8868	Cu II	307	2.98	-	8.54	4s 3D	-	4p 3P	1	-	0
222.9266	Cu II	1	8.87	-	14.43	4s2 3F	-	4p 3D	3	-	3
222.9854	Cu II	1165	8.86	-	14.42	4p 3D	-	4d 3F	2	-	3
223.0146	Cu II	431	9.06	-	14.62	4p 3D	-	4d 3D	1	-	1
223.0400	Cu II	140	8.78	-	14.34	4p 3D	-	4d 3G	3	-	4
223.0953	Cu II	947	9.09	-	14.65	4p 1D	-	4d 1D	2	-	2
223.1583	Cu II	290	8.78	-	14.34	4p 3D	-	4d 3P	3	-	2
223.9285	Cu II	1	8.66	-	14.20	4p 3F	-	4d 3S	2	-	1
224.2143	Cu II	90	8.86	-	14.39	4p 3D	-	4d 3D	2	-	3
224.2618	Cu II	1413	3.26	-	8.78	4s 1D	-	4p 3D	2	-	3
224.2718	Cu II	109	9.09	-	14.62	4p 1D	-	4d 3D	2	-	1
224.3094	Cu II	170	9.12	-	14.65	4p 1P	-	4d 1D	1	-	2
224.7002	Cu II	1403	2.72	-	8.23	4s 3D	-	4p 3P	3	-	2
224.8127	Cu II	5	8.92	-	14.43	4p 1F	-	4d 3D	3	-	2
224.8967	Cu II	917	8.92	-	14.43	4p 1F	-	4d 3F	3	-	4
225.1224	Cu II	2	9.02	-	14.52	4s2 3F	-	4p 3D	2	-	2
225.1260	Cu II	13	8.92	-	14.42	4p 1F	-	4d 3F	3	-	3
225.1856	Cu II	19	9.09	-	14.60	4p 1D	-	4d 3G	2	-	3
225.3028	Cu II	1	10.59	-	16.09	4s2 1D	-	4p 3P	2	-	1
225.4988	Cu II	382	9.12	-	14.62	4p 1P	-	4d 3D	1	-	1
225.6855	Cu II	1	8.64	-	14.13	4s2 3F	-	4p 5F	4	-	3
226.3000	Cu II	2	11.85	-	17.33	4s2 1G	-	6p 3D	4	-	3
226.3213	Cu II	329	8.86	-	14.34	4p 3D	-	4d 3P	2	-	1
226.3786	Cu II	1234	8.92	-	14.39	4p 1F	-	4d 3D	3	-	3
226.4565	Cu II	3	8.86	-	14.34	4p 3D	-	4d 3P	2	-	2
226.5365	Cu II	192	9.06	-	14.53	4p 3D	-	4d 1P	1	-	1
227.4741	Cu II	27	8.23	-	13.68	4p 3P	-	5s 1D	2	-	2
227.6157	Cu II	2	9.02	-	14.46	4s2 3F	-	4p 3G	2	-	3
227.6258	Cu II	141	2.98	-	8.42	4s 3D	-	4p 3P	1	-	1
227.8338	Cu II	256	9.09	-	14.53	4p 1D	-	4d 1P	2	-	1
228.0830	Cu II	1	11.85	-	17.28	4s2 1G	-	6p 3F	4	-	4
228.0943	Cu II	3	8.87	-	14.30	4s2 3F	-	4p 3G	3	-	4

228.4199	Cu II	3	11.85	-	17.28	4s2 1G	-	6p 3F	4	-	3
228.5404	Cu II	2	8.92	-	14.34	4p 1F	-	4d 3G	3	-	4
228.6150	Cu II	4	10.59	-	16.01	4s2 1D	-	4p 3D	2	-	3
228.6645	Cu II	449	8.92	-	14.34	4p 1F	-	4d 3P	3	-	2
228.9417	Cu II	4	8.23	-	13.65	4p 3P	-	5s 3D	2	-	1
229.0161	Cu II	0	9.02	-	14.43	4s2 3F	-	4p 3D	2	-	3
229.1002	Cu II	320	9.12	-	14.53	4p 1P	-	4d 1P	1	-	1
229.2692	Cu II	4	3.26	-	8.66	4s 1D	-	4p 3F	2	-	2
229.2970	Cu II	1	8.64	-	14.05	4s2 3F	-	4p 5F	4	-	4
229.4368	Cu II	99	2.83	-	8.23	4s 3D	-	4p 3P	2	-	2
229.9489	Cu II	79	9.06	-	14.45	4p 3D	-	4d 3P	1	-	0
229.9979	Cu II	1	10.59	-	15.98	4s2 1D	-	4p 3D	2	-	2
230.9519	Cu II	53	9.06	-	14.43	4p 3D	-	4d 3D	1	-	2
231.5156	Cu II	1	10.59	-	15.94	4s2 1D	-	4p 3D	2	-	2
231.5683	Cu II	3	10.59	-	15.94	4s2 1D	-	4p 3F	2	-	3
232.3004	Cu II	33	9.09	-	14.43	4p 1D	-	4d 3D	2	-	2
232.3928	Cu II	5	8.86	-	14.20	4p 3D	-	4d 3S	2	-	1
232.5909	Cu II	1	9.12	-	14.45	4p 1P	-	4d 3P	1	-	0
232.6349	Cu II	3	9.09	-	14.42	4p 1D	-	4d 3F	2	-	3
232.7552	Cu II	1	10.96	-	16.28	4s2 3P	-	4p 5D	2	-	3
232.7567	Cu II	1	8.87	-	14.19	4s2 3F	-	4p 5F	3	-	2
233.3752	Cu II	8	11.85	-	17.16	4s2 1G	-	4p 3G	4	-	3
233.6171	Cu II	231	9.12	-	14.43	4p 1P	-	4d 3D	1	-	2
233.9728	Cu II	7	9.09	-	14.39	4p 1D	-	4d 3D	2	-	3
234.7710	Cu II	1	10.96	-	16.24	4s2 3P	-	4p 5D	2	-	2
234.7885	Cu II	0	10.96	-	16.23	4s2 3P	-	4p 5D	2	-	1
234.8733	Cu II	111	9.06	-	14.34	4p 3D	-	4d 3P	1	-	1
235.0189	Cu II	5	9.06	-	14.34	4p 3D	-	4d 3P	1	-	2
235.3944	Cu II	1	8.87	-	14.13	4s2 3F	-	4p 5F	3	-	3
235.5014	Cu II	131	8.42	-	13.68	4p 3P	-	5s 1D	1	-	2
235.6641	Cu II	7	2.98	-	8.23	4s 3D	-	4p 3P	1	-	2
235.9257	Cu II	1	11.85	-	17.10	4s2 1G	-	4p 3P	4	-	4
236.1191	Cu II	1	10.99	-	16.24	4s2 3P	-	4p 5D	1	-	2
236.1368	Cu II	0	10.99	-	16.23	4s2 3P	-	4p 5D	1	-	1
236.2682	Cu II	4	9.09	-	14.34	4p 1D	-	4d 3P	2	-	1
236.4154	Cu II	25	9.09	-	14.34	4p 1D	-	4d 3P	2	-	2
236.6979	Cu II	5	13.39	-	18.63	5s 3D	-	4p 3F	3	-	2
236.9890	Cu II	159	3.26	-	8.49	4s 1D	-	4p 3F	2	-	3
237.0534	Cu II	0	8.64	-	13.87	4s2 3F	-	4p 5G	4	-	3
237.0747	Cu II	75	8.42	-	13.65	4p 3P	-	5s 3D	1	-	1
237.6303	Cu II	392	9.12	-	14.34	4p 1P	-	4d 3P	1	-	1
237.7793	Cu II	6	9.12	-	14.34	4p 1P	-	4d 3P	1	-	2

237.8405	Cu II	1	9.02	-	14.23	4s2 3F	-	4p 5F	2	-	1
237.8845	Cu II	2	10.96	-	16.17	4s2 3P	-	4p 3P	2	-	2
237.9406	Cu II	1	11.03	-	16.23	4s2 3P	-	4p 5D	0	-	1
238.4859	Cu II	12	8.49	-	13.68	4p 3F	-	5s 1D	3	-	2
238.4945	Cu II	33	8.23	-	13.43	4p 3P	-	5s 3D	2	-	2
238.5095	Cu II	5	13.43	-	18.63	5s 3D	-	4p 3F	2	-	2
239.2686	Cu II	3	10.99	-	16.17	4s2 3P	-	4p 3P	1	-	2
239.3261	Cu II	0	8.87	-	14.05	4s2 3F	-	4p 5F	3	-	4
239.4030	Cu II	1	9.02	-	14.19	4s2 3F	-	4p 5F	2	-	2
240.0115	Cu II	24	3.26	-	8.42	4s 1D	-	4p 3P	2	-	1
240.3337	Cu II	723	8.23	-	13.39	4p 3P	-	5s 3D	2	-	3
240.7927	Cu II	2	11.85	-	17.00	4s2 1G	-	4p 3G	4	-	5
241.4189	Cu II	8	9.06	-	14.20	4p 3D	-	4d 3S	1	-	1
241.4857	Cu II	0	10.96	-	16.09	4s2 3P	-	4p 3P	2	-	1
242.1943	Cu II	0	9.02	-	14.13	4s2 3F	-	4p 5F	2	-	3
242.4435	Cu II	156	8.54	-	13.65	4p 3P	-	5s 3D	0	-	1
242.8928	Cu II	15	9.09	-	14.20	4p 1D	-	4d 3S	2	-	1
242.9122	Cu II	1	10.99	-	16.09	4s2 3P	-	4p 3P	1	-	1
243.0678	Cu II	1	11.85	-	16.95	4s2 1G	-	4p 3G	4	-	4
244.2640	Cu II	3	8.64	-	13.72	4s2 3F	-	4p 5G	4	-	5
244.3326	Cu II	26	9.12	-	14.20	4p 1P	-	4d 3S	1	-	1
244.4657	Cu II	0	8.87	-	13.94	4s2 3F	-	4p 5G	3	-	2
244.5672	Cu II	1	11.85	-	16.92	4s2 1G	-	4p 3D	4	-	3
244.8213	Cu II	2	11.03	-	16.09	4s2 3P	-	4p 3P	0	-	1
245.2948	Cu II	5	10.96	-	16.01	4s2 3P	-	4p 3D	2	-	3
246.8501	Cu II	63	8.66	-	13.68	4p 3F	-	5s 1D	2	-	2
246.8875	Cu II	1	10.96	-	15.98	4s2 3P	-	4p 3D	2	-	2
247.3334	Cu II	262	8.42	-	13.43	4p 3P	-	5s 3D	1	-	2
247.7882	Cu II	0	8.87	-	13.87	4s2 3F	-	4p 5G	3	-	3
248.2548	Cu II	0	11.85	-	16.84	4s2 1G	-	4p 3F	4	-	4
248.3787	Cu II	4	10.99	-	15.98	4s2 3P	-	4p 3D	1	-	2
248.5792	Cu II	484	8.66	-	13.65	4p 3F	-	5s 3D	2	-	1
248.6370	Cu II	1	10.96	-	15.94	4s2 3P	-	4p 3D	2	-	2
248.6978	Cu II	1	10.96	-	15.94	4s2 3P	-	4p 3F	2	-	3
248.8806	Cu II	1	11.85	-	16.83	4s2 1G	-	4p 3F	4	-	3
248.8954	Cu II	1	13.65	-	18.63	5s 3D	-	4p 3F	1	-	2
248.9700	Cu II	7	3.26	-	8.23	4s 1D	-	4p 3P	2	-	2
250.1495	Cu II	2	10.99	-	15.94	4s2 3P	-	4p 3D	1	-	2
250.6273	Cu II	865	8.49	-	13.43	4p 3F	-	5s 3D	3	-	2
250.6533	Cu II	2	13.68	-	18.63	5s 1D	-	4p 3F	2	-	2
250.8067	Cu II	0	10.59	-	15.53	4s2 1D	-	4p 5P	2	-	2

250.9201	Cu II	1	10.59	-	15.53	4s2 1D	-	4p 5P	2	-	3
251.8079	Cu II	1	9.02	-	13.94	4s2 3F	-	4p 5G	2	-	2
251.8949	Cu II	2	8.87	-	13.79	4s2 3F	-	4p 5G	3	-	4
252.6323	Cu II	1	8.64	-	13.55	4s2 3F	-	4p 5D	4	-	3
252.6593	Cu II	174	8.49	-	13.39	4p 3F	-	5s 3D	3	-	3
252.9304	Cu II	568	8.78	-	13.68	4p 3D	-	5s 1D	3	-	2
254.4805	Cu II	742	8.52	-	13.39	4p 3F	-	5s 3D	4	-	3
254.7686	Cu II	0	13.55	-	18.41	4p 5D	-	6d 1G	3	-	4
255.3343	Cu II	1	9.02	-	13.87	4s2 3F	-	4p 5G	2	-	3
257.1756	Cu II	66	8.86	-	13.68	4p 3D	-	5s 1D	2	-	2
257.4413	Cu II	1	8.87	-	13.68	4s2 3F	-	4p 5D	3	-	2
259.0529	Cu II	148	8.86	-	13.65	4p 3D	-	5s 3D	2	-	1
259.3579	Cu II	0	11.85	-	16.63	4s2 1G	-	4p 3H	4	-	5
259.8812	Cu II	195	8.66	-	13.43	4p 3F	-	5s 3D	2	-	2
260.0270	Cu II	450	8.92	-	13.68	4p 1F	-	5s 1D	3	-	2
260.3336	Cu II	0	9.02	-	13.78	4s2 3F	-	4p 5D	2	-	1
261.2412	Cu II	0	13.68	-	18.43	4p 5D	-	6d 1F	2	-	3
261.4413	Cu II	1	8.64	-	13.38	4s2 3F	-	4p 5D	4	-	4
261.7278	Cu II	0	13.68	-	18.42	4p 5D	-	6d 1D	2	-	2
261.9211	Cu II	5	10.59	-	15.32	4s2 1D	-	5p 1D	2	-	2
262.0666	Cu II	9	8.66	-	13.39	4p 3F	-	5s 3D	2	-	3
263.6620	Cu II	1	10.59	-	15.29	4s2 1D	-	5p 3D	2	-	1
264.5231	Cu II	0	14.34	-	19.03	4d 3G	-	6f 3H	4	-	4
264.5619	Cu II	3	14.34	-	19.03	4d 3G	-	6f 1H	4	-	5
264.8606	Cu II	1	8.87	-	13.55	4s2 3F	-	4p 5D	3	-	3
265.5965	Cu II	1	9.02	-	13.68	4s2 3F	-	4p 5D	2	-	2
265.6802	Cu II	0	10.59	-	15.25	4s2 1D	-	5p 1F	2	-	3
266.4604	Cu II	0	13.78	-	18.43	4p 5D	-	6d 3F	1	-	2
266.6291	Cu II	117	8.78	-	13.43	4p 3D	-	5s 3D	3	-	2
266.7370	Cu II	1	10.59	-	15.23	4s2 1D	-	5p 1P	2	-	1
267.0141	Cu II	0	14.05	-	18.69	4p 5F	-	8s 3D	4	-	3
267.0478	Cu II	0	13.78	-	18.42	4p 5D	-	6d 1D	1	-	2
267.4793	Cu II	0	14.39	-	19.03	4d 3D	-	6f 3H	3	-	4
267.6067	Cu II	3	10.59	-	15.22	4s2 1D	-	5p 3F	2	-	2
268.1341	Cu II	1	13.55	-	18.17	4p 5D	-	6d 3F	3	-	4
268.2750	Cu II	2	9.06	-	13.68	4p 3D	-	5s 1D	1	-	2
268.5924	Cu II	0	13.55	-	18.16	4p 5D	-	6d 3D	3	-	3
268.9300	Cu II	310	8.78	-	13.39	4p 3D	-	5s 3D	3	-	3
269.1746	Cu II	0	13.55	-	18.15	4p 5D	-	6d 3P	3	-	2
269.2494	Cu II	15	14.42	-	19.03	4d 3F	-	6f 3H	3	-	4
269.5780	Cu II	1	14.43	-	19.03	4d 3F	-	6f 3H	4	-	4
269.6184	Cu II	9	14.43	-	19.03	4d 3F	-	6f 1H	4	-	5

270.0962	Cu II	364	9.09	-	13.68	4p 1D	-	5s 1D	2	-	2
270.3184	Cu II	390	9.06	-	13.65	4p 3D	-	5s 3D	1	-	1
270.4516	Cu II	1	10.99	-	15.57	4s2 3P	-	4p 5P	1	-	1
270.9760	Cu II	0	14.20	-	18.77	4d 3S	-	6f 3D	1	-	2
271.0244	Cu II	1	10.96	-	15.53	4s2 3P	-	4p 5P	2	-	2
271.1569	Cu II	1	10.96	-	15.53	4s2 3P	-	4p 5P	2	-	3
271.1864	Cu II	45	14.20	-	18.77	4d 3S	-	6f 3P	1	-	2
271.3508	Cu II	372	8.86	-	13.43	4p 3D	-	5s 3D	2	-	2
271.7915	Cu II	0	14.13	-	18.69	4p 5F	-	8s 3D	3	-	2
271.8778	Cu II	301	9.12	-	13.68	4p 1P	-	5s 1D	1	-	2
272.1677	Cu II	107	9.09	-	13.65	4p 1D	-	5s 3D	2	-	1
272.8202	Cu II	0	11.03	-	15.57	4s2 3P	-	4p 5P	0	-	1
272.8225	Cu II	0	10.99	-	15.53	4s2 3P	-	4p 5P	1	-	2
272.8514	Cu II	0	13.87	-	18.41	4p 5G	-	6d 1G	3	-	4
273.1949	Cu II	11	10.59	-	15.12	4s2 1D	-	5p 3D	2	-	2
273.3479	Cu II	0	13.68	-	18.22	4p 5D	-	7s 1D	2	-	2
273.5004	Cu II	0	9.02	-	13.55	4s2 3F	-	4p 5D	2	-	3
273.7342	Cu II	16	8.86	-	13.39	4p 3D	-	5s 3D	2	-	3
273.9767	Cu II	13	9.12	-	13.65	4p 1P	-	5s 3D	1	-	1
274.5271	Cu II	57	8.92	-	13.43	4p 1F	-	5s 3D	3	-	2
274.5592	Cu II	0	8.87	-	13.38	4s2 3F	-	4p 5D	3	-	4
276.0748	Cu II	0	13.68	-	18.17	4p 5D	-	6d 3D	2	-	2
276.2175	Cu II	0	13.68	-	18.17	4p 5D	-	6d 3F	2	-	3
276.6500	Cu II	1	10.59	-	15.07	4s2 1D	-	5p 3D	2	-	3
276.9669	Cu II	490	8.92	-	13.39	4p 1F	-	5s 3D	3	-	3
277.2562	Cu II	0	13.94	-	18.41	4p 5G	-	6d 3G	2	-	3
279.1795	Cu II	390	14.33	-	18.77	4d 3G	-	6f 3H	5	-	6
279.2212	Cu II	6	14.33	-	18.77	4d 3G	-	6f 3H	5	-	5
279.5195	Cu II	20	14.34	-	18.77	4d 3P	-	6f 3D	2	-	2
279.5298	Cu II	86	14.34	-	18.77	4d 3P	-	6f 3F	2	-	3
279.5475	Cu II	0	13.78	-	18.21	4p 5D	-	7s 3D	1	-	1
279.5873	Cu II	5	14.34	-	18.77	4d 3G	-	6f 3F	4	-	3
279.6630	Cu II	0	11.85	-	16.28	4s2 1G	-	4p 5D	4	-	3
279.7156	Cu II	1	14.34	-	18.77	4d 3G	-	6f 3F	4	-	3
279.7256	Cu II	65	14.34	-	18.77	4d 3P	-	6f 3D	1	-	2
279.7267	Cu II	2	14.20	-	18.63	4d 3S	-	4p 3F	1	-	2
279.7434	Cu II	69	14.34	-	18.77	4d 3P	-	6f 3P	2	-	2
279.9528	Cu II	10	14.34	-	18.77	4d 3P	-	6f 3P	1	-	2
279.9529	Cu II	316	14.34	-	18.77	4d 3G	-	6f 3H	4	-	5
279.9680	Cu II	248	14.60	-	19.03	4d 3G	-	6f 3H	3	-	4
280.1050	Cu II	1	10.59	-	15.01	4s2 1D	-	4p 1F	2	-	3
281.0365	Cu II	8	14.62	-	19.03	4d 1G	-	6f 3H	4	-	4

281.0804	Cu II	317	14.62	-	19.03	4d 1G	-	6f 1H	4	-	5
281.2990	Cu II	0	13.55	-	17.95	4p 5D	-	7s 3D	3	-	3
281.3632	Cu II	1	10.59	-	14.99	4s2 1D	-	5p 3P	2	-	1
281.7085	Cu II	3	10.59	-	14.99	4s2 1D	-	4p 1D	2	-	2
282.5355	Cu II	0	14.30	-	18.69	4p 3G	-	8s 3D	4	-	3
282.8742	Cu II	0	13.79	-	18.17	4p 5G	-	6d 3F	4	-	4
282.8919	Cu II	10	14.39	-	18.77	4d 3D	-	6f 3F	3	-	3
283.0126	Cu II	6	14.39	-	18.77	4d 3D	-	6f 3D	3	-	2
283.0232	Cu II	90	14.39	-	18.77	4d 3D	-	6f 3F	3	-	3
283.2354	Cu II	0	10.59	-	14.96	4s2 1D	-	5p 3F	2	-	3
283.2422	Cu II	17	14.39	-	18.77	4d 3D	-	6f 3P	3	-	2
283.7368	Cu II	73	9.06	-	13.43	4p 3D	-	5s 3D	1	-	2
284.0492	Cu II	3	10.96	-	15.32	4s2 3P	-	5p 1D	2	-	2
284.1365	Cu II	0	13.79	-	18.15	4p 5G	-	6d 3G	4	-	5
284.8725	Cu II	61	14.42	-	18.77	4d 3F	-	6f 3F	3	-	3
284.9950	Cu II	8	14.42	-	18.77	4d 3F	-	6f 3D	3	-	2
285.0057	Cu II	2	14.42	-	18.77	4d 3F	-	6f 3F	3	-	3
285.2278	Cu II	0	14.42	-	18.77	4d 3F	-	6f 3P	3	-	2
285.2405	Cu II	2	14.43	-	18.77	4d 3F	-	6f 3F	4	-	3
285.3740	Cu II	11	14.43	-	18.77	4d 3F	-	6f 3F	4	-	3
285.3757	Cu II	117	14.43	-	18.77	4d 3D	-	6f 3F	2	-	3
285.4986	Cu II	51	14.43	-	18.77	4d 3D	-	6f 3D	2	-	2
285.5094	Cu II	17	14.43	-	18.77	4d 3D	-	6f 3F	2	-	3
285.6210	Cu II	6	14.43	-	18.77	4d 3F	-	6f 3H	4	-	5
285.7322	Cu II	8	14.43	-	18.77	4d 3D	-	6f 3P	2	-	2
285.7748	Cu II	4	9.09	-	13.43	4p 1D	-	5s 3D	2	-	2
285.8642	Cu II	0	14.69	-	19.03	4d 1F	-	6f 3H	3	-	4
286.0249	Cu II	1	10.99	-	15.32	4s2 3P	-	5p 1D	1	-	2
287.7100	Cu II	0		-			-			-	
287.7699	Cu II	97	9.12	-	13.43	4p 1P	-	5s 3D	1	-	2
288.0700	Cu II	0	10.59	-	14.89	4s2 1D	-	5p 3P	2	-	2
288.1022	Cu II	1	10.99	-	15.29	4s2 3P	-	5p 3D	1	-	1
288.4196	Cu II	29	9.09	-	13.39	4p 1D	-	5s 3D	2	-	3
288.4756	Cu II	1	10.96	-	15.25	4s2 3P	-	5p 1F	2	-	3
288.6769	Cu II	0	14.13	-	18.43	4p 5F	-	6d 1F	3	-	3
288.8401	Cu II	3	14.34	-	18.63	4d 3P	-	4p 3F	2	-	2
289.3776	Cu II	0	13.87	-	18.15	4p 5G	-	6d 3G	3	-	4
289.6085	Cu II	0	13.68	-	17.96	4p 5D	-	7s 3D	2	-	2
289.6434	Cu II	1	14.13	-	18.41	4p 5F	-	6d 1G	3	-	4
289.7220	Cu II	1	10.96	-	15.23	4s2 3P	-	5p 1P	2	-	1
290.5530	Cu II	1	14.43	-	18.69	4p 3D	-	8s 3D	3	-	2

290.7483	Cu II	1	10.96	-	15.22	4s2 3P	-	5p 3F	2	-	2
290.7916	Cu II	1	11.03	-	15.29	4s2 3P	-	5p 3D	0	-	1
290.8874	Cu II	2	14.43	-	18.69	4p 3D	-	8s 3D	3	-	3
291.7777	Cu II	1	10.99	-	15.23	4s2 3P	-	5p 1P	1	-	1
292.5717	Cu II	3	14.39	-	18.63	4d 3D	-	4p 3F	3	-	2
292.6475	Cu II	0	14.19	-	18.43	4p 5F	-	6d 3F	2	-	2
292.7923	Cu II	3	14.53	-	18.77	4d 1P	-	6f 3P	1	-	2
292.8186	Cu II	0	10.99	-	15.22	4s2 3P	-	5p 3F	1	-	2
292.8387	Cu II	0	14.46	-	18.69	4p 3G	-	8s 3D	3	-	2
293.1784	Cu II	1	14.46	-	18.69	4p 3G	-	8s 3D	3	-	3
294.0309	Cu II	1	14.19	-	18.41	4p 5F	-	6d 3G	2	-	3
294.5364	Cu II	0	11.03	-	15.23	4s2 3P	-	5p 1P	0	-	1
294.6907	Cu II	2	14.42	-	18.63	4d 3F	-	4p 3F	3	-	2
295.2292	Cu II	1	14.43	-	18.63	4d 3D	-	4p 3F	2	-	2
296.8988	Cu II	4	14.60	-	18.77	4d 3G	-	6f 3F	3	-	3
297.0319	Cu II	1	14.60	-	18.77	4d 3G	-	6f 3D	3	-	2
297.0436	Cu II	0	14.60	-	18.77	4d 3G	-	6f 3F	3	-	3
297.0714	Cu II	2	14.52	-	18.69	4p 3D	-	8s 3D	2	-	2
297.0825	Cu II	1	10.59	-	14.76	4s2 1D	-	4p 3F	2	-	2
297.3565	Cu II	1	10.96	-	15.12	4s2 3P	-	5p 3D	2	-	2
297.3647	Cu II	1	10.99	-	15.15	4s2 3P	-	5p 3P	1	-	0
297.4210	Cu II	1	14.52	-	18.69	4p 3D	-	8s 3D	2	-	3
297.5658	Cu II	0	13.79	-	17.95	4p 5G	-	7s 3D	4	-	3
297.9641	Cu II	1	11.85	-	16.01	4s2 1G	-	4p 3D	4	-	3
298.1007	Cu II	1	14.62	-	18.77	4d 1G	-	6f 3F	4	-	3
298.1786	Cu II	1	13.39	-	17.55	5s 3D	-	6p 1D	3	-	2
298.2466	Cu II	1	14.62	-	18.77	4d 1G	-	6f 3F	4	-	3
298.3766	Cu II	5	14.20	-	18.35	4d 3S	-	5f 3D	1	-	1
298.5163	Cu II	2	14.62	-	18.77	4d 1G	-	6f 3H	4	-	5
298.6335	Cu II	22	14.20	-	18.35	4d 3S	-	5f 1D	1	-	2
298.6368	Cu II	1	14.62	-	18.77	4d 3D	-	6f 3D	1	-	2
298.8924	Cu II	0	14.62	-	18.77	4d 3D	-	6f 3P	1	-	2
299.3268	Cu II	1	13.43	-	17.57	5s 3D	-	6p 3F	2	-	2
299.5224	Cu II	0	10.99	-	15.12	4s2 3P	-	5p 3D	1	-	2
300.6019	Cu II	1	14.05	-	18.17	4p 5F	-	6d 3F	4	-	4
300.6122	Cu II	3	14.65	-	18.77	4d 1D	-	6f 3F	2	-	3
300.7486	Cu II	1	14.65	-	18.77	4d 1D	-	6f 3D	2	-	2
300.7606	Cu II	3	14.65	-	18.77	4d 1D	-	6f 3F	2	-	3
301.0046	Cu II	0	11.85	-	15.97	4s2 1G	-	4p 3F	4	-	4
301.0078	Cu II	2	14.65	-	18.77	4d 1D	-	6f 3P	2	-	2
301.0591	Cu II	4	13.43	-	17.55	5s 3D	-	6p 1D	2	-	2
301.1780	Cu II	0	14.05	-	18.16	4p 5F	-	6d 3D	4	-	3

301.2278	Cu II	1	13.39	-	17.51	5s 3D	-	6p 1F	3	-	3
301.4543	Cu II	2	10.96	-	15.07	4s2 3P	-	5p 3D	2	-	3
301.8409	Cu II	0	14.05	-	18.15	4p 5F	-	6d 3G	4	-	4
302.0278	Cu II	1	14.05	-	18.15	4p 5F	-	6d 3G	4	-	5
302.2435	Cu II	4	14.59	-	18.69	4p 3F	-	8s 3D	4	-	3
302.7725	Cu II	1	14.53	-	18.63	4d 1P	-	4p 3F	1	-	2
302.8818	Cu II	0	13.87	-	17.96	4p 5G	-	7s 3D	3	-	2
303.0002	Cu II	1	11.85	-	15.94	4s2 1G	-	4p 3F	4	-	3
303.5319	Cu II	0	14.13	-	18.22	4p 5F	-	7s 1D	3	-	2
303.5381	Cu II	0	14.69	-	18.77	4d 1F	-	6f 3F	3	-	3
303.6771	Cu II	0	14.69	-	18.77	4d 1F	-	6f 3D	3	-	2
303.6894	Cu II	1	14.69	-	18.77	4d 1F	-	6f 3F	3	-	3
303.7801	Cu II	1	13.43	-	17.51	5s 3D	-	6p 3D	2	-	1
303.9415	Cu II	1	14.69	-	18.77	4d 1F	-	6f 3P	3	-	2
304.1337	Cu II	1	14.70	-	18.77	4d 3F	-	6f 3F	2	-	3
304.1678	Cu II	6	13.43	-	17.51	5s 3D	-	6p 1F	2	-	3
304.3865	Cu II	1	14.70	-	18.77	4d 3F	-	6f 3P	2	-	2
305.2158	Cu II	1	10.59	-	14.65	4s2 1D	-	4p 3F	2	-	3
305.5612	Cu II	0	10.96	-	15.01	4s2 3P	-	4p 1F	2	-	3
305.6173	Cu II	1	14.64	-	18.69	4p 3D	-	8s 3D	1	-	2
305.8849	Cu II	1	10.59	-	14.64	4s2 1D	-	4p 3D	2	-	1
306.2882	Cu II	3	14.65	-	18.69	4p 3F	-	8s 3D	3	-	2
306.6598	Cu II	3	14.65	-	18.69	4p 3F	-	8s 3D	3	-	3
306.8980	Cu II	0	14.13	-	18.17	4p 5F	-	6d 3D	3	-	2
307.0591	Cu II	0	10.96	-	14.99	4s2 3P	-	5p 3P	2	-	1
307.0744	Cu II	1	14.13	-	18.17	4p 5F	-	6d 3F	3	-	3
307.4704	Cu II	1	10.96	-	14.99	4s2 3P	-	4p 1D	2	-	2
307.5789	Cu II	1	14.60	-	18.63	4d 3G	-	4p 3F	3	-	2
307.6440	Cu II	0	14.13	-	18.16	4p 5F	-	6d 3D	3	-	3
307.9424	Cu II	1	14.34	-	18.36	4d 3P	-	5f 3G	2	-	3
307.9845	Cu II	0	14.33	-	18.35	4d 3G	-	5f 1H	5	-	5
308.0328	Cu II	0	14.19	-	18.22	4p 5F	-	7s 1D	2	-	2
308.1453	Cu II	2	14.34	-	18.36	4d 3G	-	5f 1G	4	-	4
308.1679	Cu II	0	14.34	-	18.36	4d 3G	-	5f 3G	4	-	3
308.2935	Cu II	1	14.34	-	18.36	4d 3P	-	5f 3F	2	-	2
308.3357	Cu II	1	14.13	-	18.15	4p 5F	-	6d 3G	3	-	4
308.3367	Cu II	5	14.34	-	18.36	4d 3P	-	5f 1F	2	-	3
308.4081	Cu II	0	14.13	-	18.15	4p 5F	-	6d 3P	3	-	2
308.5097	Cu II	0	14.19	-	18.21	4p 5F	-	7s 3D	2	-	1
308.5443	Cu II	2	14.34	-	18.36	4d 3P	-	5f 3F	1	-	2
308.5628	Cu II	0	14.34	-	18.36	4d 3G	-	5f 1F	4	-	3
308.7682	Cu II	1	14.34	-	18.35	4d 3P	-	5f 3D	2	-	1

308.8287	Cu II	0	14.34	-	18.35	4d 3G	-	5f 3H	4	-	4
308.8749	Cu II	10	14.34	-	18.35	4d 3G	-	5f 1H	4	-	5
309.0197	Cu II	1	14.34	-	18.35	4d 3P	-	5f 3D	1	-	1
309.0432	Cu II	1	14.34	-	18.35	4d 3P	-	5f 1D	2	-	2
309.2902	Cu II	1	10.96	-	14.96	4s2 3P	-	5p 3F	2	-	3
309.2951	Cu II	1	14.34	-	18.35	4d 3P	-	5f 1D	1	-	2
309.3002	Cu II	0	14.62	-	18.63	4d 3D	-	4p 3F	1	-	2
309.3691	Cu II	0	10.99	-	14.99	4s2 3P	-	5p 3P	1	-	1
309.7866	Cu II	1	10.99	-	14.99	4s2 3P	-	4p 1D	1	-	2
309.8235	Cu II	0	14.43	-	18.43	4p 3D	-	6d 3F	3	-	2
309.9329	Cu II	1	14.43	-	18.43	4p 3D	-	6d 1F	3	-	3
310.6180	Cu II	0	14.43	-	18.42	4p 3D	-	6d 1D	3	-	2
311.0472	Cu II	4	14.43	-	18.41	4p 3D	-	6d 1G	3	-	4
311.3745	Cu II	1	14.43	-	18.41	4p 3D	-	6d 3G	3	-	3
311.5661	Cu II	1	14.65	-	18.63	4d 1D	-	4p 3F	2	-	2
311.6818	Cu II	0	14.19	-	18.17	4p 5F	-	6d 3F	2	-	3
312.1642	Cu II	7	14.39	-	18.36	4d 3D	-	5f 1G	3	-	4
312.1874	Cu II	1	14.39	-	18.36	4d 3D	-	5f 3G	3	-	3
312.4238	Cu II	0	14.46	-	18.43	4p 3G	-	6d 3F	3	-	2
312.4722	Cu II	1	11.03	-	14.99	4s2 3P	-	5p 3P	0	-	1
312.5350	Cu II	1	14.46	-	18.43	4p 3G	-	6d 1F	3	-	3
312.5483	Cu II	0	14.39	-	18.36	4d 3D	-	5f 3F	3	-	2
312.5927	Cu II	1	14.39	-	18.36	4d 3D	-	5f 1F	3	-	3
312.8656	Cu II	1	14.39	-	18.35	4d 3D	-	5f 3H	3	-	4
313.2317	Cu II	0	14.46	-	18.42	4p 3G	-	6d 1D	3	-	2
313.3188	Cu II	0	14.39	-	18.35	4d 3D	-	5f 1D	3	-	2
313.6681	Cu II	2	14.46	-	18.41	4p 3G	-	6d 1G	3	-	4
314.0010	Cu II	0	14.46	-	18.41	4p 3G	-	6d 3G	3	-	3
314.5777	Cu II	0	14.42	-	18.36	4d 3F	-	5f 1G	3	-	4
314.6012	Cu II	6	14.42	-	18.36	4d 3F	-	5f 3G	3	-	3
314.7101	Cu II	1	14.69	-	18.63	4d 1F	-	4p 3F	3	-	2
314.8539	Cu II	1	14.75	-	18.69	4p 1G	-	8s 3D	4	-	3
314.9407	Cu II	2	14.76	-	18.69	4p 3F	-	8s 3D	2	-	2
314.9531	Cu II	1	10.59	-	14.52	4s2 1D	-	4p 3D	2	-	2
314.9677	Cu II	1	14.42	-	18.36	4d 3F	-	5f 3F	3	-	2
315.0265	Cu II	4	14.43	-	18.36	4d 3F	-	5f 1G	4	-	4
315.0500	Cu II	1	14.43	-	18.36	4d 3F	-	5f 3G	4	-	3
315.0642	Cu II	1	10.96	-	14.89	4s2 3P	-	5p 3P	2	-	2
315.1051	Cu II	14	13.39	-	17.33	5s 3D	-	6p 3D	3	-	3
315.1873	Cu II	1	14.70	-	18.63	4d 3F	-	4p 3F	2	-	2
315.2150	Cu II	1	14.43	-	18.36	4d 3D	-	5f 3G	2	-	3

315.2900	Cu II	44	14.42	-	18.35	4d 3F	-	5f 3H	3	-	4
315.3336	Cu II	1	14.76	-	18.69	4p 3F	-	8s 3D	2	-	3
315.4094	Cu II	1	13.39	-	17.32	5s 3D	-	6p 3D	3	-	2
315.4628	Cu II	1	14.43	-	18.36	4d 3F	-	5f 1F	4	-	3
315.5829	Cu II	1	14.43	-	18.36	4d 3D	-	5f 3F	2	-	2
315.6282	Cu II	18	14.43	-	18.36	4d 3D	-	5f 1F	2	-	3
315.7408	Cu II	1	14.43	-	18.35	4d 3F	-	5f 3H	4	-	4
315.7890	Cu II	27	14.43	-	18.35	4d 3F	-	5f 1H	4	-	5
315.8673	Cu II	18	13.65	-	17.57	5s 3D	-	6p 3F	1	-	2
316.0804	Cu II	1	14.43	-	18.35	4d 3D	-	5f 3D	2	-	1
316.3685	Cu II	4	14.43	-	18.35	4d 3D	-	5f 1D	2	-	2
317.2462	Cu II	0	14.52	-	18.43	4p 3D	-	6d 3F	2	-	2
317.2467	Cu II	1	14.05	-	17.95	4p 5F	-	7s 3D	4	-	3
317.3609	Cu II	7	14.52	-	18.43	4p 3D	-	6d 1F	2	-	3
317.4967	Cu II	1	10.99	-	14.89	4s2 3P	-	5p 3P	1	-	2
317.6311	Cu II	0	14.20	-	18.10	4d 3S	-	5f 3D	1	-	2
317.7970	Cu II	1	13.65	-	17.55	5s 3D	-	6p 1D	1	-	2
317.9316	Cu II	1	14.20	-	18.10	4d 3S	-	5f 1P	1	-	1
317.9784	Cu II	61	14.45	-	18.35	4d 3P	-	5f 3D	0	-	1
318.0793	Cu II	3	14.52	-	18.42	4p 3D	-	6d 1D	2	-	2
318.2172	Cu II	148	14.20	-	18.09	4d 3S	-	5f 3P	1	-	2
318.3236	Cu II	7	13.43	-	17.33	5s 3D	-	6p 3D	2	-	3
318.4682	Cu II	0	14.52	-	18.41	4p 3D	-	6d 3D	2	-	1
318.4841	Cu II	180	14.20	-	18.09	4d 3S	-	5f 3P	1	-	1
318.5725	Cu II	35	13.39	-	17.28	5s 3D	-	6p 3F	3	-	4
318.6015	Cu II	76	14.20	-	18.09	4d 3S	-	5f 3P	1	-	0
318.6341	Cu II	6	13.43	-	17.32	5s 3D	-	6p 3D	2	-	2
318.7039	Cu II	0	13.68	-	17.57	5s 1D	-	6p 3F	2	-	2
318.8726	Cu II	5	14.52	-	18.41	4p 3D	-	6d 3G	2	-	3
319.2301	Cu II	15	13.39	-	17.28	5s 3D	-	6p 3F	3	-	3
319.8546	Cu II	1	10.59	-	14.46	4s2 1D	-	4p 3G	2	-	3
320.1001	Cu II	1	14.52	-	18.39	4p 3D	-	6d 1P	2	-	1
320.4184	Cu II	0	14.30	-	18.17	4p 3G	-	6d 3F	4	-	4
320.6685	Cu II	11	13.68	-	17.55	5s 1D	-	6p 1D	2	-	2
320.8305	Cu II	10	13.65	-	17.51	5s 3D	-	6p 3D	1	-	1
321.0730	Cu II	0	14.30	-	18.16	4p 3G	-	6d 3D	4	-	3
321.1274	Cu II	1	14.64	-	18.50	4p 3D	-	6d 1S	1	-	0
321.8264	Cu II	0	14.30	-	18.15	4p 3G	-	6d 3G	4	-	4
322.0389	Cu II	1	14.30	-	18.15	4p 3G	-	6d 3G	4	-	5
322.3434	Cu II	4	13.39	-	17.24	5s 3D	-	4p 3D	3	-	2
322.5339	Cu II	8	13.43	-	17.28	5s 3D	-	6p 3F	2	-	3
322.6267	Cu II	1	10.59	-	14.43	4s2 1D	-	4p 3D	2	-	3

323.4734	Cu II	6	13.43	-	17.26	5s 3D	-	6p 3P	2	-	1
323.5623	Cu II	1	13.65	-	17.48	5s 3D	-	6p 1P	1	-	1
323.7141	Cu II	1	14.13	-	17.96	4p 5F	-	7s 3D	3	-	2
323.7573	Cu II	2	13.68	-	17.51	5s 1D	-	6p 3D	2	-	1
324.0669	Cu II	0	14.59	-	18.41	4p 3F	-	6d 1G	4	-	4
324.1977	Cu II	13	13.68	-	17.51	5s 1D	-	6p 1F	2	-	3
324.2174	Cu II	1	14.53	-	18.36	4d 1P	-	5f 3F	1	-	2
324.4293	Cu II	0	14.13	-	17.95	4p 5F	-	7s 3D	3	-	3
324.7424	Cu II	70	14.53	-	18.35	4d 1P	-	5f 3D	1	-	1
325.0466	Cu II	326	14.53	-	18.35	4d 1P	-	5f 1D	1	-	2
325.7122	Cu II	2	13.43	-	17.24	5s 3D	-	4p 3D	2	-	2
325.7443	Cu II	3	14.89	-	18.69	5p 3P	-	8s 3D	2	-	2
325.8764	Cu II	0	10.96	-	14.76	4s2 3P	-	4p 3F	2	-	2
326.1647	Cu II	49	14.89	-	18.69	5p 3P	-	8s 3D	2	-	3
326.5394	Cu II	4	13.68	-	17.48	5s 1D	-	6p 1P	2	-	1
327.0112	Cu II	12	14.64	-	18.43	4p 3D	-	6d 3F	1	-	2
327.1211	Cu II	2	14.43	-	18.22	4p 3D	-	7s 1D	3	-	2
327.7794	Cu II	0	14.65	-	18.43	4p 3F	-	6d 3F	3	-	2
327.8965	Cu II	5	14.64	-	18.42	4p 3D	-	6d 1D	1	-	2
327.9019	Cu II	1	14.65	-	18.43	4p 3F	-	6d 1F	3	-	3
328.1697	Cu II	198	14.33	-	18.11	4d 3G	-	5f 3G	5	-	5
328.2011	Cu II	17	14.33	-	18.11	4d 3G	-	5f 3G	5	-	4
328.2602	Cu II	4	14.33	-	18.10	4d 3G	-	5f 3F	5	-	4
328.3098	Cu II	8	14.64	-	18.41	4p 3D	-	6d 3D	1	-	1
328.4794	Cu II	1	10.99	-	14.76	4s2 3P	-	4p 3F	1	-	2
328.9921	Cu II	1	13.39	-	17.16	5s 3D	-	4p 3G	3	-	3
329.0418	Cu II	1283	14.33	-	18.10	4d 3G	-	5f 3H	5	-	6
329.0424	Cu II	21	14.34	-	18.10	4d 3P	-	5f 3D	2	-	3
329.1060	Cu II	20	14.33	-	18.10	4d 3G	-	5f 3H	5	-	5
329.1494	Cu II	1	14.65	-	18.41	4p 3F	-	6d 1G	3	-	4
329.1808	Cu II	3	14.34	-	18.11	4d 3G	-	5f 3G	4	-	5
329.2123	Cu II	3	14.34	-	18.11	4d 3G	-	5f 3G	4	-	4
329.2718	Cu II	171	14.34	-	18.10	4d 3G	-	5f 3F	4	-	4
329.2998	Cu II	15	14.34	-	18.10	4d 3G	-	5f 3D	4	-	3
329.3075	Cu II	3	14.60	-	18.36	4d 3G	-	5f 1G	3	-	4
329.3333	Cu II	91	14.60	-	18.36	4d 3G	-	5f 3G	3	-	3
329.4335	Cu II	63	14.34	-	18.10	4d 3P	-	5f 3D	2	-	2
329.5102	Cu II	283	14.34	-	18.10	4d 3P	-	5f 3F	2	-	3
329.5159	Cu II	0	14.65	-	18.41	4p 3F	-	6d 3G	3	-	3
329.7199	Cu II	212	14.34	-	18.10	4d 3P	-	5f 3D	1	-	2
329.7349	Cu II	5	14.60	-	18.36	4d 3G	-	5f 3F	3	-	2
329.7569	Cu II	10	14.34	-	18.10	4d 3P	-	5f 1P	2	-	1

329.7684	Cu II	1	14.34	-	18.10	4d 3G	-	5f 3F	4	-	3
329.7843	Cu II	0	14.60	-	18.36	4d 3G	-	5f 1F	3	-	3
330.0211	Cu II	1	14.46	-	18.22	4p 3G	-	7s 1D	3	-	2
330.0438	Cu II	147	14.34	-	18.10	4d 3P	-	5f 1P	1	-	1
330.0444	Cu II	0	14.64	-	18.39	4p 3D	-	6d 1P	1	-	1
330.0641	Cu II	224	14.34	-	18.09	4d 3P	-	5f 3P	2	-	2
330.0881	Cu II	817	14.60	-	18.35	4d 3G	-	5f 3H	3	-	4
330.1229	Cu II	1038	14.34	-	18.10	4d 3G	-	5f 3H	4	-	5
330.3512	Cu II	83	14.34	-	18.09	4d 3P	-	5f 3P	2	-	1
330.3515	Cu II	31	14.34	-	18.09	4d 3P	-	5f 3P	1	-	2
330.6392	Cu II	2	14.34	-	18.09	4d 3P	-	5f 3P	1	-	1
330.7657	Cu II	21	14.34	-	18.09	4d 3P	-	5f 3P	1	-	0
330.7868	Cu II	118	14.62	-	18.36	4d 1G	-	5f 1G	4	-	4
330.8127	Cu II	4	14.62	-	18.36	4d 1G	-	5f 3G	4	-	3
331.0340	Cu II	1	14.43	-	18.17	4p 3D	-	6d 3D	3	-	2
331.2027	Cu II	20	14.43	-	18.17	4p 3D	-	6d 3F	3	-	4
331.2393	Cu II	1	14.43	-	18.17	4p 3D	-	6d 3F	3	-	3
331.2678	Cu II	6	14.62	-	18.36	4d 1G	-	5f 1F	4	-	3
331.5744	Cu II	24	14.62	-	18.35	4d 1G	-	5f 3H	4	-	4
331.6276	Cu II	1040	14.62	-	18.35	4d 1G	-	5f 1H	4	-	5
331.7139	Cu II	327	14.62	-	18.36	4d 3D	-	5f 3F	1	-	2
331.9021	Cu II	13	14.43	-	18.16	4p 3D	-	6d 3D	3	-	3
332.1553	Cu II	58	14.96	-	18.69	5p 3F	-	8s 3D	3	-	2
332.2635	Cu II	65	14.62	-	18.35	4d 3D	-	5f 3D	1	-	1
332.5021	Cu II	1	13.43	-	17.16	5s 3D	-	4p 3G	2	-	3
332.5819	Cu II	18	14.62	-	18.35	4d 3D	-	5f 1D	1	-	2
332.5924	Cu II	6	14.96	-	18.69	5p 3F	-	8s 3D	3	-	3
332.7073	Cu II	1	14.43	-	18.15	4p 3D	-	6d 3G	3	-	4
332.7916	Cu II	5	14.43	-	18.15	4p 3D	-	6d 3P	3	-	2
333.7594	Cu II	88	14.98	-	18.69	5p 3F	-	8s 3D	4	-	3
333.8036	Cu II	548	14.39	-	18.11	4d 3D	-	5f 3G	3	-	4
333.8648	Cu II	3	14.39	-	18.10	4d 3D	-	5f 3F	3	-	4
333.8936	Cu II	30	14.39	-	18.10	4d 3D	-	5f 3D	3	-	3
333.9085	Cu II	39	14.65	-	18.36	4d 1D	-	5f 3G	2	-	3
334.0041	Cu II	0	14.46	-	18.17	4p 3G	-	6d 3D	3	-	2
334.0832	Cu II	2	13.39	-	17.10	5s 3D	-	4p 3P	3	-	4
334.1759	Cu II	9	14.46	-	18.17	4p 3G	-	6d 3F	3	-	4
334.2131	Cu II	0	14.46	-	18.17	4p 3G	-	6d 3F	3	-	3
334.2800	Cu II	32	14.99	-	18.69	4p 1D	-	8s 3D	2	-	2
334.2964	Cu II	18	14.39	-	18.10	4d 3D	-	5f 3D	3	-	2
334.3214	Cu II	9	14.65	-	18.36	4d 1D	-	5f 3F	2	-	2

334.3722	Cu II	485	14.65	-	18.36	4d 1D	-	5f 1F	2	-	3
334.3753	Cu II	294	14.39	-	18.10	4d 3D	-	5f 3F	3	-	3
334.7228	Cu II	2	14.99	-	18.69	4p 1D	-	8s 3D	2	-	3
334.7676	Cu II	27	14.99	-	18.69	5p 3P	-	8s 3D	1	-	2
334.8797	Cu II	9	14.65	-	18.35	4d 1D	-	5f 3D	2	-	1
334.8879	Cu II	7	14.46	-	18.16	4p 3G	-	6d 3D	3	-	3
334.9457	Cu II	54	14.39	-	18.09	4d 3D	-	5f 3P	3	-	2
335.2031	Cu II	114	14.65	-	18.35	4d 1D	-	5f 1D	2	-	2
335.4067	Cu II	2	14.52	-	18.22	4p 3D	-	7s 1D	2	-	2
335.6887	Cu II	1	10.96	-	14.65	4s2 3P	-	4p 3F	2	-	3
335.7077	Cu II	1	14.46	-	18.15	4p 3G	-	6d 3G	3	-	4
335.7935	Cu II	3	14.46	-	18.15	4p 3G	-	6d 3P	3	-	2
335.9721	Cu II	4	14.52	-	18.21	4p 3D	-	7s 3D	2	-	1
336.5648	Cu II	19	14.42	-	18.11	4d 3F	-	5f 3G	3	-	4
336.6269	Cu II	652	14.42	-	18.10	4d 3F	-	5f 3F	3	-	4
336.6562	Cu II	200	14.42	-	18.10	4d 3F	-	5f 3D	3	-	3
337.0069	Cu II	0	11.85	-	15.53	4s2 1G	-	4p 5P	4	-	3
337.0150	Cu II	32	15.01	-	18.69	4p 1F	-	8s 3D	3	-	3
337.0454	Cu II	842	14.43	-	18.11	4d 3F	-	5f 3G	4	-	5
337.0657	Cu II	24	14.42	-	18.10	4d 3F	-	5f 3D	3	-	2
337.0785	Cu II	275	14.43	-	18.11	4d 3F	-	5f 3G	4	-	4
337.1408	Cu II	8	14.43	-	18.10	4d 3F	-	5f 3F	4	-	4
337.1459	Cu II	5	14.42	-	18.10	4d 3F	-	5f 3F	3	-	3
337.1702	Cu II	6	14.43	-	18.10	4d 3F	-	5f 3D	4	-	3
337.3539	Cu II	1	13.39	-	17.07	5s 3D	-	4p 1D	3	-	2
337.3591	Cu II	382	14.43	-	18.10	4d 3D	-	5f 3D	2	-	3
337.4442	Cu II	2	13.65	-	17.32	5s 3D	-	6p 3D	1	-	2
337.4952	Cu II	737	14.69	-	18.36	4d 1F	-	5f 1G	3	-	4
337.5223	Cu II	26	14.69	-	18.36	4d 1F	-	5f 3G	3	-	3
337.6614	Cu II	36	14.43	-	18.10	4d 3F	-	5f 3F	4	-	3
337.7084	Cu II	6	14.76	-	18.43	4p 3F	-	6d 3F	2	-	2
337.7258	Cu II	1	14.42	-	18.09	4d 3F	-	5f 3P	3	-	2
337.7704	Cu II	167	14.43	-	18.10	4d 3D	-	5f 3D	2	-	2
337.8383	Cu II	1	14.76	-	18.43	4p 3F	-	6d 1F	2	-	3
337.8509	Cu II	54	14.43	-	18.10	4d 3D	-	5f 3F	2	-	3
337.9441	Cu II	8	14.69	-	18.36	4d 1F	-	5f 3F	3	-	2
337.9939	Cu II	1	13.43	-	17.10	5s 3D	-	4p 3S	2	-	1
337.9960	Cu II	156	14.69	-	18.36	4d 1F	-	5f 1F	3	-	3
338.0331	Cu II	19	14.43	-	18.10	4d 3F	-	5f 3H	4	-	5
338.0712	Cu II	510	14.70	-	18.36	4d 3F	-	5f 3G	2	-	3
338.1103	Cu II	24	14.43	-	18.10	4d 3D	-	5f 1P	2	-	1
338.3152	Cu II	1	14.69	-	18.35	4d 1F	-	5f 3H	3	-	4

338.4332	Cu II	26	14.43	-	18.09	4d 3D	-	5f 3P	2	-	2
338.4945	Cu II	135	14.70	-	18.36	4d 3F	-	5f 3F	2	-	2
338.5465	Cu II	10	14.70	-	18.36	4d 3F	-	5f 1F	2	-	3
338.7352	Cu II	1	14.43	-	18.09	4d 3D	-	5f 3P	2	-	1
338.8451	Cu II	9	14.69	-	18.35	4d 1F	-	5f 1D	3	-	2
339.0668	Cu II	11	14.70	-	18.35	4d 3F	-	5f 3D	2	-	1
339.0935	Cu II	1	14.76	-	18.41	4p 3F	-	6d 3D	2	-	1
339.2744	Cu II	1	10.99	-	14.64	4s2 3P	-	4p 3D	1	-	1
339.3984	Cu II	5	14.70	-	18.35	4d 3F	-	5f 1D	2	-	2
339.3991	Cu II	0	14.30	-	17.95	4p 3G	-	7s 3D	4	-	3
339.5216	Cu II	10	14.52	-	18.17	4p 3D	-	6d 3D	2	-	2
339.5519	Cu II	17	14.76	-	18.41	4p 3F	-	6d 3G	2	-	3
339.7375	Cu II	16	14.52	-	18.17	4p 3D	-	6d 3F	2	-	3
340.2830	Cu II	63	14.45	-	18.10	4d 3P	-	5f 1P	0	-	1
340.3285	Cu II	1	13.68	-	17.33	5s 1D	-	6p 3D	2	-	3
340.4349	Cu II	1	14.52	-	18.16	4p 3D	-	6d 3D	2	-	3
340.6835	Cu II	0	13.68	-	17.32	5s 1D	-	6p 3D	2	-	2
340.9160	Cu II	15	14.45	-	18.09	4d 3P	-	5f 3P	0	-	1
340.9442	Cu II	1	14.76	-	18.39	4p 3F	-	6d 1P	2	-	1
341.0455	Cu II	1	13.43	-	17.07	5s 3D	-	4p 1D	2	-	2
341.2270	Cu II	5	14.52	-	18.15	4p 3D	-	6d 3P	2	-	1
341.3707	Cu II	4	14.52	-	18.15	4p 3D	-	6d 3P	2	-	2
341.6936	Cu II	11	15.07	-	18.69	5p 3D	-	8s 3D	3	-	2
342.1562	Cu II	31	15.07	-	18.69	5p 3D	-	8s 3D	3	-	3
342.8766	Cu II	1	13.65	-	17.26	5s 3D	-	6p 3P	1	-	1
343.0101	Cu II	1	11.03	-	14.64	4s2 3P	-	4p 3D	0	-	1
343.7178	Cu II	2	14.52	-	18.13	4p 3D	-	6d 3S	2	-	1
343.9774	Cu II	1	13.39	-	17.00	5s 3D	-	4p 3G	3	-	3
344.4137	Cu II	2	14.64	-	18.24	4p 3D	-	6d 3P	1	-	0
345.1454	Cu II	1	13.68	-	17.28	5s 1D	-	6p 3F	2	-	3
345.3931	Cu II	1	13.65	-	17.24	5s 3D	-	4p 3D	1	-	2
346.0044	Cu II	14	14.59	-	18.17	4p 3F	-	6d 3F	4	-	4
346.0443	Cu II	1	14.59	-	18.17	4p 3F	-	6d 3F	4	-	3
346.1952	Cu II	1	13.43	-	17.01	5s 3D	-	4p 1P	2	-	1
346.3410	Cu II	2	14.64	-	18.22	4p 3D	-	7s 1D	1	-	2
346.7679	Cu II	3	14.59	-	18.16	4p 3F	-	6d 3D	4	-	3
346.9439	Cu II	6	14.64	-	18.21	4p 3D	-	7s 3D	1	-	1
347.1155	Cu II	12	15.12	-	18.69	5p 3D	-	8s 3D	2	-	2
347.2028	Cu II	1	14.65	-	18.22	4p 3F	-	7s 1D	3	-	2
347.3002	Cu II	2	13.39	-	16.96	5s 3D	-	4p 3F	3	-	2
347.5049	Cu II	1	10.96	-	14.52	4s2 3P	-	4p 3D	2	-	2
347.5929	Cu II	1	15.12	-	18.69	5p 3D	-	8s 3D	2	-	3

347.6469	Cu II	3	14.59	-	18.15	4p 3F	-	6d 3G	4	-	4
347.8163	Cu II	0	13.43	-	17.00	5s 3D	-	4p 3G	2	-	3
347.8949	Cu II	52	14.59	-	18.15	4p 3F	-	6d 3G	4	-	5
348.3830	Cu II	8	14.53	-	18.09	4d 1P	-	5f 3P	1	-	2
348.7029	Cu II	11	14.53	-	18.09	4d 1P	-	5f 3P	1	-	1
348.7875	Cu II	0	13.68	-	17.24	5s 1D	-	4p 3D	2	-	2
348.8437	Cu II	5	14.53	-	18.09	4d 1P	-	5f 3P	1	-	0
350.1613	Cu II	1	14.89	-	18.43	5p 3P	-	6d 3F	2	-	2
350.3010	Cu II	4	14.89	-	18.43	5p 3P	-	6d 1F	2	-	3
350.4664	Cu II	2	10.99	-	14.52	4s2 3P	-	4p 3D	1	-	2
350.6836	Cu II	3	14.43	-	17.96	4p 3D	-	7s 3D	3	-	2
350.7302	Cu II	2	14.64	-	18.17	4p 3D	-	6d 3D	1	-	2
351.1764	Cu II	2	14.89	-	18.42	5p 3P	-	6d 1D	2	-	2
351.2140	Cu II	8	13.43	-	16.96	5s 3D	-	4p 3F	2	-	2
351.5230	Cu II	9	14.43	-	17.95	4p 3D	-	7s 3D	3	-	3
351.6140	Cu II	2	14.65	-	18.17	4p 3F	-	6d 3D	3	-	2
351.6506	Cu II	1	14.89	-	18.41	5p 3P	-	6d 3D	2	-	1
351.6780	Cu II	2	13.39	-	16.92	5s 3D	-	4p 3D	3	-	3
351.8044	Cu II	4	14.65	-	18.17	4p 3F	-	6d 3F	3	-	4
351.8457	Cu II	14	14.65	-	18.17	4p 3F	-	6d 3F	3	-	3
352.1437	Cu II	0	14.89	-	18.41	5p 3P	-	6d 3G	2	-	3
352.5504	Cu II	2	14.64	-	18.15	4p 3D	-	6d 3P	1	-	1
352.5937	Cu II	11	14.65	-	18.16	4p 3F	-	6d 3D	3	-	3
353.4808	Cu II	1	14.60	-	18.11	4d 3G	-	5f 3G	3	-	4
353.4814	Cu II	1	10.96	-	14.46	4s2 3P	-	4p 3G	2	-	3
353.5025	Cu II	49	14.65	-	18.15	4p 3F	-	6d 3G	3	-	4
353.5493	Cu II	32	14.60	-	18.10	4d 3G	-	5f 3F	3	-	4
353.5816	Cu II	12	14.60	-	18.10	4d 3G	-	5f 3D	3	-	3
353.5976	Cu II	6	14.65	-	18.15	4p 3F	-	6d 3P	3	-	2
353.6413	Cu II	1	14.89	-	18.39	5p 3P	-	6d 1P	2	-	1
354.0186	Cu II	1	14.46	-	17.96	4p 3G	-	7s 3D	3	-	2
354.0334	Cu II	2	14.60	-	18.10	4d 3G	-	5f 3D	3	-	2
354.1219	Cu II	1	14.60	-	18.10	4d 3G	-	5f 3F	3	-	3
354.8741	Cu II	6	14.46	-	17.95	4p 3G	-	7s 3D	3	-	3
355.1489	Cu II	21	14.62	-	18.11	4d 1G	-	5f 3G	4	-	5
355.1857	Cu II	9	14.62	-	18.11	4d 1G	-	5f 3G	4	-	4
355.2099	Cu II	1	14.64	-	18.13	4p 3D	-	6d 3S	1	-	1
355.2549	Cu II	3	14.62	-	18.10	4d 1G	-	5f 3F	4	-	4
355.2875	Cu II	1	14.62	-	18.10	4d 1G	-	5f 3D	4	-	3
355.6917	Cu II	0	13.43	-	16.92	5s 3D	-	4p 3D	2	-	3
355.8330	Cu II	2	14.62	-	18.10	4d 1G	-	5f 3F	4	-	3

356.2458	Cu II	5	14.62	-	18.10	4d 1G	-	5f 3H	4	-	5
356.3158	Cu II	1	14.62	-	18.10	4d 3D	-	5f 3D	1	-	2
356.5759	Cu II	2	15.22	-	18.69	5p 3F	-	8s 3D	2	-	2
356.5851	Cu II	2	13.68	-	17.16	5s 1D	-	4p 3G	2	-	3
356.6940	Cu II	1	14.62	-	18.10	4d 3D	-	5f 1P	1	-	1
356.8701	Cu II	2	10.96	-	14.43	4s2 3P	-	4p 3D	2	-	3
357.0535	Cu II	1	14.62	-	18.09	4d 3D	-	5f 3P	1	-	2
357.0797	Cu II	1	15.22	-	18.69	5p 3F	-	8s 3D	2	-	3
357.5375	Cu II	1	14.62	-	18.09	4d 3D	-	5f 3P	1	-	0
357.8406	Cu II	1	13.39	-	16.86	5s 3D	-	4p 5S	3	-	2
358.1317	Cu II	4	15.23	-	18.69	5p 1P	-	8s 3D	1	-	2
358.3632	Cu II	3	14.76	-	18.22	4p 3F	-	7s 1D	2	-	2
358.6390	Cu II	0	14.96	-	18.42	5p 3F	-	6d 1D	3	-	2
358.8608	Cu II	10	14.65	-	18.10	4d 1D	-	5f 3D	2	-	3
359.0088	Cu II	5	14.76	-	18.21	4p 3F	-	7s 3D	2	-	1
359.0764	Cu II	0	14.98	-	18.43	5p 3F	-	6d 1F	4	-	3
359.2113	Cu II	1	14.96	-	18.41	5p 3F	-	6d 1G	3	-	4
359.2352	Cu II	11	13.65	-	17.10	5s 3D	-	4p 3S	1	-	1
359.3261	Cu II	3	14.65	-	18.10	4d 1D	-	5f 3D	2	-	2
359.3534	Cu II	1	13.39	-	16.84	5s 3D	-	4p 3F	3	-	4
359.4173	Cu II	7	14.65	-	18.10	4d 1D	-	5f 3F	2	-	3
359.6478	Cu II	1	14.96	-	18.41	5p 3F	-	6d 3G	3	-	3
359.7108	Cu II	1	14.65	-	18.10	4d 1D	-	5f 1P	2	-	1
360.0183	Cu II	0	13.55	-	16.99	4p 5D	-	5d 3F	3	-	4
360.0439	Cu II	6	14.99	-	18.43	4p 1D	-	6d 3F	2	-	2
360.0547	Cu II	1	15.25	-	18.69	5p 1F	-	8s 3D	3	-	2
360.0764	Cu II	5	14.65	-	18.09	4d 1D	-	5f 3P	2	-	2
360.1916	Cu II	24	14.99	-	18.43	4p 1D	-	6d 1F	2	-	3
360.2232	Cu II	8	14.52	-	17.96	4p 3D	-	7s 3D	2	-	2
360.4182	Cu II	1	14.65	-	18.09	4d 1D	-	5f 3P	2	-	1
360.5684	Cu II	1	15.25	-	18.69	5p 1F	-	8s 3D	3	-	3
360.5729	Cu II	1	14.98	-	18.41	5p 3F	-	6d 1G	4	-	4
360.6096	Cu II	1	14.99	-	18.43	5p 3P	-	6d 3F	1	-	2
360.6659	Cu II	18	13.39	-	16.83	5s 3D	-	4p 3F	3	-	3
361.1090	Cu II	3	14.52	-	17.95	4p 3D	-	7s 3D	2	-	3
361.1173	Cu II	11	14.99	-	18.42	4p 1D	-	6d 1D	2	-	2
361.6187	Cu II	1	14.99	-	18.41	4p 1D	-	6d 3D	2	-	1
361.6478	Cu II	0	13.55	-	16.98	4p 5D	-	5d 3D	3	-	3
361.6863	Cu II	26	14.99	-	18.42	5p 3P	-	6d 1D	1	-	2
361.9970	Cu II	0	13.43	-	16.86	5s 3D	-	4p 5S	2	-	2
362.1401	Cu II	1	14.99	-	18.41	4p 1D	-	6d 3G	2	-	3
362.1893	Cu II	2	14.99	-	18.41	5p 3P	-	6d 3D	1	-	1

362.6310	Cu II	2	14.75	-	18.17	4p 1G	-	6d 3F	4	-	4
362.6748	Cu II	0	14.75	-	18.17	4p 1G	-	6d 3F	4	-	3
362.6844	Cu II	1	13.65	-	17.07	5s 3D	-	4p 1D	1	-	2
362.6975	Cu II	2	15.01	-	18.43	4p 1F	-	6d 3F	3	-	2
362.8474	Cu II	15	15.01	-	18.43	4p 1F	-	6d 1F	3	-	3
362.9087	Cu II	2	13.68	-	17.10	5s 1D	-	4p 3S	2	-	1
362.9318	Cu II	5	14.69	-	18.11	4d 1F	-	5f 3G	3	-	4
363.0382	Cu II	1	14.69	-	18.10	4d 1F	-	5f 3D	3	-	3
363.0646	Cu II	9	14.76	-	18.17	4p 3F	-	6d 3D	2	-	2
363.3115	Cu II	17	14.76	-	18.17	4p 3F	-	6d 3F	2	-	3
363.4697	Cu II	1	14.75	-	18.16	4p 1G	-	6d 3D	4	-	3
363.5144	Cu II	1	14.69	-	18.10	4d 1F	-	5f 3D	3	-	2
363.6077	Cu II	2	14.69	-	18.10	4d 1F	-	5f 3F	3	-	3
363.6733	Cu II	0	14.70	-	18.10	4d 3F	-	5f 3D	2	-	3
363.7242	Cu II	3	14.99	-	18.39	4p 1D	-	6d 1P	2	-	1
363.7868	Cu II	1	15.01	-	18.42	4p 1F	-	6d 1D	3	-	2
364.1092	Cu II	1	14.76	-	18.16	4p 3F	-	6d 3D	2	-	3
364.1792	Cu II	4	11.85	-	15.25	4s2 1G	-	5p 1F	4	-	3
364.2448	Cu II	1	14.70	-	18.10	4d 3F	-	5f 3F	2	-	3
364.2823	Cu II	1	14.69	-	18.09	4d 1F	-	5f 3P	3	-	2
364.3015	Cu II	19	14.99	-	18.39	5p 3P	-	6d 1P	1	-	1
364.3756	Cu II	51	15.01	-	18.41	4p 1F	-	6d 1G	3	-	4
364.4355	Cu II	1	14.75	-	18.15	4p 1G	-	6d 3G	4	-	4
364.7080	Cu II	5	14.75	-	18.15	4p 1G	-	6d 3G	4	-	5
364.8248	Cu II	3	15.01	-	18.41	4p 1F	-	6d 3G	3	-	3
364.8886	Cu II	4	13.43	-	16.83	5s 3D	-	4p 3F	2	-	3
364.9218	Cu II	1	14.70	-	18.09	4d 3F	-	5f 3P	2	-	2
365.0154	Cu II	5	14.76	-	18.15	4p 3F	-	6d 3P	2	-	1
365.1799	Cu II	5	14.76	-	18.15	4p 3F	-	6d 3P	2	-	2
365.2728	Cu II	1	14.70	-	18.09	4d 3F	-	5f 3P	2	-	1
366.4291	Cu II	4	13.68	-	17.07	5s 1D	-	4p 1D	2	-	2
367.1963	Cu II	1	15.32	-	18.69	5p 1D	-	8s 3D	2	-	2
367.7306	Cu II	1	15.32	-	18.69	5p 1D	-	8s 3D	2	-	3
367.8670	Cu II	2	14.76	-	18.13	4p 3F	-	6d 3S	2	-	1
368.2425	Cu II	21	14.59	-	17.95	4p 3F	-	7s 3D	4	-	3
368.5139	Cu II	0	13.65	-	17.01	5s 3D	-	4p 1P	1	-	1
368.6555	Cu II	1	15.07	-	18.43	5p 3D	-	6d 3F	3	-	2
368.8138	Cu II	4	15.07	-	18.43	5p 3D	-	6d 1F	3	-	3
369.7844	Cu II	1	15.07	-	18.42	5p 3D	-	6d 1D	3	-	2
369.8435	Cu II	0	14.20	-	17.55	4d 3S	-	6p 1D	1	-	2
370.3928	Cu II	24	15.07	-	18.41	5p 3D	-	6d 1G	3	-	4
370.8570	Cu II	1	15.07	-	18.41	5p 3D	-	6d 3G	3	-	3

372.3806	Cu II	5	13.68	-	17.01	5s 1D	-	4p 1P	2	-	1
372.4176	Cu II	2	14.89	-	18.22	5p 3P	-	7s 1D	2	-	2
372.8658	Cu II	2	14.64	-	17.96	4p 3D	-	7s 3D	1	-	2
373.0344	Cu II	2	13.43	-	16.75	5s 3D	-	4p 3D	2	-	1
373.1148	Cu II	1	14.89	-	18.21	5p 3P	-	7s 3D	2	-	1
373.8648	Cu II	12	14.65	-	17.96	4p 3F	-	7s 3D	3	-	2
373.9583	Cu II	0	14.20	-	17.51	4d 3S	-	6p 3D	1	-	1
374.0329	Cu II	3	13.39	-	16.71	5s 3D	-	4p 3H	3	-	4
374.2059	Cu II	6	13.65	-	16.96	5s 3D	-	4p 3F	1	-	2
374.2568	Cu II	1	13.68	-	17.00	5s 1D	-	4p 3G	2	-	3
374.8190	Cu II	12	14.65	-	17.95	4p 3F	-	7s 3D	3	-	3
374.8318	Cu II	0	13.68	-	16.99	4p 5D	-	5d 3F	2	-	3
374.9776	Cu II	2	13.39	-	16.70	5s 3D	-	4p 3D	3	-	2
374.9783	Cu II	60	15.12	-	18.43	5p 3D	-	6d 3F	2	-	2
375.1385	Cu II	48	15.12	-	18.43	5p 3D	-	6d 1F	2	-	3
376.1427	Cu II	11	15.12	-	18.42	5p 3D	-	6d 1D	2	-	2
376.6867	Cu II	10	15.12	-	18.41	5p 3D	-	6d 3D	2	-	1
377.2526	Cu II	85	15.12	-	18.41	5p 3D	-	6d 3G	2	-	3
377.4975	Cu II	14	14.89	-	18.17	5p 3P	-	6d 3D	2	-	2
377.7645	Cu II	4	14.89	-	18.17	5p 3P	-	6d 3F	2	-	3
378.1936	Cu II	6	13.68	-	16.96	5s 1D	-	4p 3F	2	-	2
378.6270	Cu II	191	14.89	-	18.16	5p 3P	-	6d 3D	2	-	3
378.9719	Cu II	8	15.12	-	18.39	5p 3D	-	6d 1P	2	-	1
379.5442	Cu II	2	13.43	-	16.70	5s 3D	-	4p 3D	2	-	2
379.6070	Cu II	25	14.89	-	18.15	5p 3P	-	6d 3P	2	-	1
379.6169	Cu II	69	15.23	-	18.50	5p 1P	-	6d 1S	1	-	0
379.7849	Cu II	286	14.89	-	18.15	5p 3P	-	6d 3P	2	-	2
380.1556	Cu II	92	15.15	-	18.41	5p 3P	-	6d 3D	0	-	1
380.8210	Cu II	1	14.96	-	18.22	5p 3F	-	7s 1D	3	-	2
381.8879	Cu II	88	14.99	-	18.24	5p 3P	-	6d 3P	1	-	0
382.4832	Cu II	61	15.15	-	18.39	5p 3P	-	6d 1P	0	-	1
382.6921	Cu II	234	14.89	-	18.13	5p 3P	-	6d 3S	2	-	1
383.4883	Cu II	0	14.34	-	17.57	4d 3P	-	6p 3F	1	-	2
383.6165	Cu II	13	14.99	-	18.22	4p 1D	-	7s 1D	2	-	2
384.1482	Cu II	3	13.43	-	16.66	5s 3D	-	4p 3P	2	-	1
384.2587	Cu II	8	14.99	-	18.22	5p 3P	-	7s 1D	1	-	2
384.3564	Cu II	1	14.99	-	18.21	4p 1D	-	7s 3D	2	-	1
384.9582	Cu II	24	13.39	-	16.61	5s 3D	-	4p 3D	3	-	3
385.0010	Cu II	5	14.99	-	18.21	5p 3P	-	7s 3D	1	-	1
385.1103	Cu II	5	11.85	-	15.07	4s2 1G	-	5p 3D	4	-	3
385.9432	Cu II	0	14.34	-	17.55	4d 3P	-	6p 1D	2	-	2

386.0240	Cu II	23	15.29	-	18.50	5p 3D	-	6d 1S	1	-	0
386.0425	Cu II	65	15.22	-	18.43	5p 3F	-	6d 3F	2	-	2
386.1344	Cu II	25	14.96	-	18.17	5p 3F	-	6d 3D	3	-	2
386.2124	Cu II	50	15.22	-	18.43	5p 3F	-	6d 1F	2	-	3
386.3362	Cu II	1	14.34	-	17.55	4d 3P	-	6p 1D	1	-	2
386.3640	Cu II	26	14.96	-	18.17	5p 3F	-	6d 3F	3	-	4
386.4137	Cu II	191	14.96	-	18.17	5p 3F	-	6d 3F	3	-	3
386.6304	Cu II	19	15.01	-	18.22	4p 1F	-	7s 1D	3	-	2
386.8371	Cu II	7	14.76	-	17.96	4p 3F	-	7s 3D	2	-	2
387.1332	Cu II	2	14.75	-	17.95	4p 1G	-	7s 3D	4	-	3
387.2768	Cu II	34	15.22	-	18.42	5p 3F	-	6d 1D	2	-	2
387.3161	Cu II	6	14.96	-	18.16	5p 3F	-	6d 3D	3	-	3
387.3207	Cu II	9	13.39	-	16.59	5s 3D	-	4p 3P	3	-	2
387.8535	Cu II	8	15.22	-	18.41	5p 3F	-	6d 3D	2	-	1
387.8588	Cu II	3	14.76	-	17.95	4p 3F	-	7s 3D	2	-	3
387.8667	Cu II	4	15.23	-	18.43	5p 1P	-	6d 3F	1	-	2
387.9397	Cu II	239	14.98	-	18.17	5p 3F	-	6d 3F	4	-	4
387.9898	Cu II	3	14.98	-	18.17	5p 3F	-	6d 3F	4	-	3
388.4131	Cu II	658	14.96	-	18.15	5p 3F	-	6d 3G	3	-	4
388.4534	Cu II	488	15.22	-	18.41	5p 3F	-	6d 3G	2	-	3
388.5279	Cu II	10	14.96	-	18.15	5p 3F	-	6d 3P	3	-	2
388.8996	Cu II	51	14.98	-	18.16	5p 3F	-	6d 3D	4	-	3
389.0087	Cu II	174	14.99	-	18.17	4p 1D	-	6d 3D	2	-	2
389.1127	Cu II	182	15.23	-	18.42	5p 1P	-	6d 1D	1	-	2
389.2924	Cu II	285	14.99	-	18.17	4p 1D	-	6d 3F	2	-	3
389.6691	Cu II	140	14.99	-	18.17	5p 3P	-	6d 3D	1	-	2
389.6949	Cu II	10	15.23	-	18.41	5p 1P	-	6d 3D	1	-	1
389.7726	Cu II	2	13.43	-	16.61	5s 3D	-	4p 3D	2	-	3
390.0056	Cu II	47	14.98	-	18.15	5p 3F	-	6d 3G	4	-	4
390.1233	Cu II	16	15.25	-	18.43	5p 1F	-	6d 3F	3	-	2
390.2082	Cu II	2	14.99	-	18.16	4p 1D	-	6d 3D	2	-	3
390.2766	Cu II	1	15.22	-	18.39	5p 3F	-	6d 1P	2	-	1
390.2968	Cu II	154	15.25	-	18.43	5p 1F	-	6d 1F	3	-	3
390.3177	Cu II	925	14.98	-	18.15	5p 3F	-	6d 3G	4	-	5
390.5114	Cu II	67	15.18	-	18.35	4d 1S	-	5f 3D	0	-	1
390.7264	Cu II	1	13.68	-	16.86	5s 1D	-	4p 5S	2	-	2
390.8284	Cu II	1	14.34	-	17.51	4d 3P	-	6p 3D	1	-	1
391.2491	Cu II	53	14.99	-	18.15	4p 1D	-	6d 3P	2	-	1
391.3838	Cu II	12	15.25	-	18.42	5p 1F	-	6d 1D	3	-	2
391.4306	Cu II	2	14.34	-	17.51	4d 3G	-	6p 1F	4	-	3
391.4381	Cu II	9	14.99	-	18.15	4p 1D	-	6d 3P	2	-	2
391.8381	Cu II	5	11.85	-	15.01	4s2 1G	-	4p 1F	4	-	3

391.9172	Cu II	144	14.99	-	18.15	5p 3P	-	6d 3P	1	-	1
391.9360	Cu II	0	13.79	-	16.95	4p 5G	-	5d 3G	4	-	5
392.0654	Cu II	727	15.25	-	18.41	5p 1F	-	6d 1G	3	-	4
392.1412	Cu II	147	15.23	-	18.39	5p 1P	-	6d 1P	1	-	1
392.1948	Cu II	0	13.43	-	16.59	5s 3D	-	4p 3P	2	-	2
392.3450	Cu II	202	15.01	-	18.17	4p 1F	-	6d 3F	3	-	4
392.3963	Cu II	7	15.01	-	18.17	4p 1F	-	6d 3F	3	-	3
392.5856	Cu II	34	15.25	-	18.41	5p 1F	-	6d 3G	3	-	3
393.3268	Cu II	168	15.01	-	18.16	4p 1F	-	6d 3D	3	-	3
393.4119	Cu II	7	15.07	-	18.22	5p 3D	-	7s 1D	3	-	2
393.4614	Cu II	1	14.42	-	17.57	4d 3F	-	6p 3F	3	-	2
393.5956	Cu II	3	13.65	-	16.80	5s 3D	-	4p 3P	1	-	0
394.0972	Cu II	3	13.68	-	16.83	5s 1D	-	4p 3F	2	-	3
394.4219	Cu II	0	14.43	-	17.57	4d 3D	-	6p 3F	2	-	2
394.4582	Cu II	32	15.01	-	18.15	4p 1F	-	6d 3G	3	-	4
394.5272	Cu II	0	14.99	-	18.13	4p 1D	-	6d 3S	2	-	1
394.5577	Cu II	222	15.29	-	18.43	5p 3D	-	6d 3F	1	-	2
394.5766	Cu II	67	15.01	-	18.15	4p 1F	-	6d 3P	3	-	2
395.2065	Cu II	34	14.99	-	18.13	5p 3P	-	6d 3S	1	-	1
395.8471	Cu II	56	15.29	-	18.42	5p 3D	-	6d 1D	1	-	2
396.4497	Cu II	133	15.29	-	18.41	5p 3D	-	6d 3D	1	-	1
396.4600	Cu II	1	14.42	-	17.55	4d 3F	-	6p 1D	3	-	2
397.4352	Cu II	1	14.43	-	17.55	4d 3D	-	6p 1D	2	-	2
397.9912	Cu II	1	11.85	-	14.96	4s2 1G	-	5p 3F	4	-	3
398.5214	Cu II	67	15.32	-	18.43	5p 1D	-	6d 3F	2	-	2
398.7024	Cu II	332	15.32	-	18.43	5p 1D	-	6d 1F	2	-	3
398.9817	Cu II	0	15.29	-	18.39	5p 3D	-	6d 1P	1	-	1
399.0778	Cu II	4	13.65	-	16.75	5s 3D	-	4p 3D	1	-	1
399.0850	Cu II	5	15.07	-	18.17	5p 3D	-	6d 3D	3	-	2
399.3302	Cu II	309	15.07	-	18.17	5p 3D	-	6d 3F	3	-	4
399.3834	Cu II	8	15.07	-	18.17	5p 3D	-	6d 3F	3	-	3
399.8369	Cu II	119	15.32	-	18.42	5p 1D	-	6d 1D	2	-	2
400.3476	Cu II	189	15.07	-	18.16	5p 3D	-	6d 3D	3	-	3
400.4516	Cu II	13	15.32	-	18.41	5p 1D	-	6d 3D	2	-	1
400.6165	Cu II	44	15.12	-	18.22	5p 3D	-	7s 1D	2	-	2
401.0912	Cu II	1	15.32	-	18.41	5p 1D	-	6d 3G	2	-	3
401.4235	Cu II	8	15.12	-	18.21	5p 3D	-	7s 3D	2	-	1
401.5196	Cu II	16	15.07	-	18.15	5p 3D	-	6d 3G	3	-	4
401.6423	Cu II	63	15.07	-	18.15	5p 3D	-	6d 3P	3	-	2
401.6713	Cu II	0	13.87	-	16.96	4p 5G	-	5d 3G	3	-	4
401.7760	Cu II	0	14.13	-	17.22	4p 5F	-	5d 1G	3	-	4
401.8686	Cu II	1	14.42	-	17.51	4d 3F	-	6p 1F	3	-	3

402.1908	Cu II	0	14.43	-	17.51	4d 3D	-	6p 3D	2	-	1
402.8706	Cu II	0	14.43	-	17.51	4d 3D	-	6p 1F	2	-	3
403.0353	Cu II	34	15.32	-	18.39	5p 1D	-	6d 1P	2	-	1
403.2647	Cu II	10	14.89	-	17.96	5p 3P	-	7s 3D	2	-	2
403.6163	Cu II	1	13.68	-	16.75	5s 1D	-	4p 3D	2	-	1
404.2547	Cu II	0	14.20	-	17.26	4d 3S	-	6p 3P	1	-	1
404.3484	Cu II	0	-		-		-				
404.3751	Cu II	150	14.89	-	17.95	5p 3P	-	7s 3D	2	-	3
405.2689	Cu II	1	14.45	-	17.51	4d 3P	-	6p 3D	0	-	1
405.3653	Cu II	33	15.15	-	18.21	5p 3P	-	7s 3D	0	-	1
405.3945	Cu II	0	14.19	-	17.25	4p 5F	-	5d 3F	2	-	2
406.5009	Cu II	50	15.12	-	18.17	5p 3D	-	6d 3D	2	-	2
406.5373	Cu II	1	13.65	-	16.70	5s 3D	-	4p 3D	1	-	2
406.8106	Cu II	82	15.12	-	18.17	5p 3D	-	6d 3F	2	-	3
407.8108	Cu II	1	15.12	-	18.16	5p 3D	-	6d 3D	2	-	3
408.0018	Cu II	0	14.53	-	17.57	4d 1P	-	6p 3F	1	-	2
408.9480	Cu II	15	15.12	-	18.15	5p 3D	-	6d 3P	2	-	1
409.1544	Cu II	3	15.12	-	18.15	5p 3D	-	6d 3P	2	-	2
410.7744	Cu II	0	14.19	-	17.21	4p 5F	-	5d 3G	2	-	3
411.2270	Cu II	0	14.53	-	17.55	4d 1P	-	6p 1D	1	-	2
411.2481	Cu II	5	13.68	-	16.70	5s 1D	-	4p 3D	2	-	2
411.8239	Cu II	0	13.65	-	16.66	5s 3D	-	4p 3P	1	-	1
412.5307	Cu II	0	15.12	-	18.13	5p 3D	-	6d 3S	2	-	1
412.5935	Cu II	1	15.23	-	18.24	5p 1P	-	6d 3P	1	-	0
413.1363	Cu II	178	14.96	-	17.96	5p 3F	-	7s 3D	3	-	2
413.2710	Cu II	3	15.22	-	18.22	5p 3F	-	7s 1D	2	-	2
414.1297	Cu II	131	15.22	-	18.21	5p 3F	-	7s 3D	2	-	1
414.3017	Cu II	14	14.96	-	17.95	5p 3F	-	7s 3D	3	-	3
414.7812	Cu II	1	14.34	-	17.33	4d 3P	-	6p 3D	2	-	3
415.1904	Cu II	5	14.34	-	17.33	4d 3G	-	6p 3D	4	-	3
415.3623	Cu II	61	15.23	-	18.22	5p 1P	-	7s 1D	1	-	2
415.7637	Cu II	1	14.34	-	17.32	4d 3P	-	6p 3D	1	-	2
416.1140	Cu II	261	14.98	-	17.95	5p 3F	-	7s 3D	4	-	3
416.2297	Cu II	23	15.23	-	18.21	5p 1P	-	7s 3D	1	-	1
416.3205	Cu II	0	14.53	-	17.51	4d 1P	-	6p 3D	1	-	1
416.4284	Cu II	112	14.99	-	17.96	4p 1D	-	7s 3D	2	-	2
416.6588	Cu II	1	13.68	-	16.66	5s 1D	-	4p 3P	2	-	1
416.6948	Cu II	1	15.15	-	18.13	5p 3P	-	6d 3S	0	-	1
416.7779	Cu II	4	14.60	-	17.57	4d 3G	-	6p 3F	3	-	2
417.1851	Cu II	83	14.99	-	17.96	5p 3P	-	7s 3D	1	-	2
417.6125	Cu II	5	14.99	-	17.95	4p 1D	-	7s 3D	2	-	3

417.9512	Cu II	194	15.25	-	18.22	5p 1F	-	7s 1D	3	-	2
419.5358	Cu II	10	15.22	-	18.17	5p 3F	-	6d 3D	2	-	2
419.5772	Cu II	10	14.33	-	17.28	4d 3G	-	6p 3F	5	-	4
419.8656	Cu II	11	15.22	-	18.17	5p 3F	-	6d 3F	2	-	3
419.9446	Cu II	0	14.62	-	17.57	4d 3D	-	6p 3F	1	-	2
419.9821	Cu II	1	15.01	-	17.96	4p 1F	-	7s 3D	3	-	2
420.1440	Cu II	0	14.60	-	17.55	4d 3G	-	6p 1D	3	-	2
420.1730	Cu II	3	15.29	-	18.24	5p 3D	-	6d 3P	1	-	0
420.9312	Cu II	1	15.22	-	18.16	5p 3F	-	6d 3D	2	-	3
421.0822	Cu II	0	14.05	-	16.99	4p 5F	-	5d 3F	4	-	4
421.0857	Cu II	0	13.65	-	16.59	5s 3D	-	4p 3P	1	-	2
421.1866	Cu II	100	15.01	-	17.95	4p 1F	-	7s 3D	3	-	3
421.2314	Cu II	1	14.34	-	17.28	4d 3G	-	6p 3F	4	-	4
421.6912	Cu II	16	15.23	-	18.17	5p 1P	-	6d 3D	1	-	2
421.9583	Cu II	1	14.34	-	17.28	4d 3P	-	6p 3F	2	-	3
422.1428	Cu II	3	15.22	-	18.15	5p 3F	-	6d 3P	2	-	1
422.3628	Cu II	1	15.22	-	18.15	5p 3F	-	6d 3P	2	-	2
422.3818	Cu II	1	14.34	-	17.28	4d 3G	-	6p 3F	4	-	3
422.5195	Cu II	2	14.39	-	17.33	4d 3D	-	6p 3D	3	-	3
423.0449	Cu II	26	15.29	-	18.22	5p 3D	-	7s 1D	1	-	2
423.2837	Cu II	2	13.68	-	16.61	5s 1D	-	4p 3D	2	-	3
423.3130	Cu II	0	14.05	-	16.98	4p 5F	-	5d 3D	4	-	3
423.3622	Cu II	0	14.62	-	17.55	4d 3D	-	6p 1D	1	-	2
423.9447	Cu II	66	15.29	-	18.21	5p 3D	-	7s 3D	1	-	1
424.0412	Cu II	1	14.34	-	17.26	4d 3P	-	6p 3P	1	-	1
424.1323	Cu II	1	14.65	-	17.57	4d 1D	-	6p 3F	2	-	2
424.3251	Cu II	20	15.23	-	18.15	5p 1P	-	6d 3P	1	-	1
424.3598	Cu II	0	15.25	-	18.17	5p 1F	-	6d 3D	3	-	2
424.6371	Cu II	0	15.25	-	18.17	5p 1F	-	6d 3F	3	-	4
424.6972	Cu II	2	15.25	-	18.17	5p 1F	-	6d 3F	3	-	3
424.6985	Cu II	38	15.18	-	18.10	4d 1S	-	5f 1P	0	-	1
425.1019	Cu II	31	14.20	-	17.11	4d 3S	-	4f 3D	1	-	1
425.5635	Cu II	166	14.20	-	17.11	4d 3S	-	4f 1D	1	-	2
425.6849	Cu II	8	15.18	-	18.09	4d 1S	-	5f 3P	0	-	1
425.7876	Cu II	3	15.25	-	18.16	5p 1F	-	6d 3D	3	-	3
426.0914	Cu II	0	14.05	-	16.96	4p 5F	-	5d 3G	4	-	4
426.1418	Cu II	0	13.68	-	16.59	5s 1D	-	4p 3P	2	-	2
426.7616	Cu II	1	11.85	-	14.75	4s2 1G	-	4p 1G	4	-	4
426.8101	Cu II	1	14.05	-	16.95	4p 5F	-	5d 3G	4	-	5
426.9530	Cu II	2	14.42	-	17.33	4d 3F	-	6p 3D	3	-	3
427.1136	Cu II	4	15.25	-	18.15	5p 1F	-	6d 3G	3	-	4
427.1898	Cu II	0	14.20	-	17.10	4d 3S	-	4p 3S	1	-	1

427.2120	Cu II	0	15.53	-	18.43	4p 5P	-	6d 1F	3	-	3
427.2525	Cu II	1	15.25	-	18.15	5p 1F	-	6d 3P	3	-	2
427.3330	Cu II	0	15.53	-	18.43	4p 5P	-	6d 3F	2	-	2
427.5118	Cu II	3	14.42	-	17.32	4d 3F	-	6p 3D	3	-	2
427.5412	Cu II	1	15.53	-	18.43	4p 5P	-	6d 1F	2	-	3
427.6048	Cu II	100	15.32	-	18.22	5p 1D	-	7s 1D	2	-	2
427.6187	Cu II	1	14.65	-	17.55	4d 1D	-	6p 1D	2	-	2
427.7800	Cu II	2	14.43	-	17.33	4d 3F	-	6p 3D	4	-	3
427.9962	Cu II	30	15.07	-	17.96	5p 3D	-	7s 3D	3	-	2
428.0842	Cu II	1	14.43	-	17.33	4d 3D	-	6p 3D	2	-	3
428.1836	Cu II	2	15.23	-	18.13	5p 1P	-	6d 3S	1	-	1
428.5243	Cu II	14	15.32	-	18.21	5p 1D	-	7s 3D	2	-	1
428.6460	Cu II	1	14.43	-	17.32	4d 3D	-	6p 3D	2	-	2
428.7043	Cu II	2	14.62	-	17.51	4d 1G	-	6p 1F	4	-	3
428.7626	Cu II	1	14.62	-	17.51	4d 3D	-	6p 3D	1	-	1
428.7772	Cu II	1	14.39	-	17.28	4d 3D	-	6p 3F	3	-	4
428.8459	Cu II	1	15.53	-	18.42	4p 5P	-	6d 1D	2	-	2
429.1084	Cu II	0	13.39	-	16.28	5s 3D	-	4p 5D	3	-	3
429.2470	Cu II	90	15.07	-	17.95	5p 3D	-	7s 3D	3	-	3
429.3320	Cu II	1	15.53	-	18.41	4p 5P	-	6d 1G	3	-	4
429.5533	Cu II	0	15.53	-	18.41	4p 5P	-	6d 3D	2	-	1
429.6119	Cu II	0	15.29	-	18.17	5p 3D	-	6d 3D	1	-	2
429.9693	Cu II	2	14.39	-	17.28	4d 3D	-	6p 3F	3	-	3
429.9799	Cu II	1	14.69	-	17.57	4d 1F	-	6p 3F	3	-	2
430.8711	Cu II	1	14.70	-	17.57	4d 3F	-	6p 3F	2	-	2
432.0761	Cu II	0	14.20	-	17.07	4d 3S	-	4p 1D	1	-	2
432.5274	Cu II	0	15.53	-	18.39	4p 5P	-	6d 1P	2	-	1
432.5768	Cu II	0	15.29	-	18.15	5p 3D	-	6d 3P	1	-	2
433.1290	Cu II	1	14.65	-	17.51	4d 1D	-	6p 3D	2	-	1
433.2822	Cu II	0	15.57	-	18.43	4p 5P	-	6d 3F	1	-	2
433.3437	Cu II	0	14.42	-	17.28	4d 3F	-	6p 3F	3	-	4
433.5634	Cu II	1	14.69	-	17.55	4d 1F	-	6p 1D	3	-	2
434.0126	Cu II	0	14.13	-	16.99	4p 5F	-	5d 3F	3	-	3
434.1957	Cu II	3	14.43	-	17.28	4d 3F	-	6p 3F	4	-	4
434.3153	Cu II	1	15.32	-	18.17	5p 1D	-	6d 3D	2	-	2
434.4696	Cu II	1	14.70	-	17.55	4d 3F	-	6p 1D	2	-	2
434.5614	Cu II	0	14.42	-	17.28	4d 3F	-	6p 3F	3	-	3
434.6688	Cu II	2	15.32	-	18.17	5p 1D	-	6d 3F	2	-	3
435.0990	Cu II	1	13.43	-	16.28	5s 3D	-	4p 5D	2	-	3
435.4182	Cu II	2	14.43	-	17.28	4d 3F	-	6p 3F	4	-	3
435.8110	Cu II	2	15.32	-	18.16	5p 1D	-	6d 3D	2	-	3
436.0099	Cu II	0	13.39	-	16.24	5s 3D	-	4p 5D	3	-	2

436.5370	Cu II	24	15.12	-	17.96	5p 3D	-	7s 3D	2	-	2
437.1099	Cu II	0	15.32	-	18.15	5p 1D	-	6d 3P	2	-	1
437.3458	Cu II	3	15.32	-	18.15	5p 1D	-	6d 3P	2	-	2
437.4498	Cu II	0	14.43	-	17.26	4d 3D	-	6p 3P	2	-	1
437.8384	Cu II	2	15.12	-	17.95	5p 3D	-	7s 3D	2	-	3
439.1159	Cu II	0	14.43	-	17.25	4p 3D	-	5d 3F	3	-	2
439.1491	Cu II	1	14.13	-	16.96	4p 5F	-	5d 3G	3	-	4
439.1831	Cu II	0	14.34	-	17.16	4d 3P	-	4p 3G	2	-	3
439.5526	Cu II	1	14.43	-	17.25	4p 3D	-	5d 1F	3	-	3
439.6419	Cu II	3	14.34	-	17.16	4d 3G	-	4p 3G	4	-	3
440.0400	Cu II	1	14.69	-	17.51	4d 1F	-	6p 1F	3	-	3
440.1590	Cu II	2	14.70	-	17.51	4d 3F	-	6p 3D	2	-	1
440.3507	Cu II	0	14.42	-	17.24	4d 3F	-	4p 3D	3	-	2
440.3751	Cu II	0	14.20	-	17.01	4d 3S	-	4p 1P	1	-	1
440.9734	Cu II	0	14.70	-	17.51	4d 3F	-	6p 1F	2	-	3
441.0937	Cu II	1	14.45	-	17.26	4d 3P	-	6p 3P	0	-	1
441.2055	Cu II	2	15.32	-	18.13	5p 1D	-	6d 3S	2	-	1
441.9083	Cu II	4	14.34	-	17.14	4d 3P	-	4f 3G	2	-	3
442.2396	Cu II	0	14.43	-	17.23	4p 3D	-	5d 1D	3	-	2
442.2583	Cu II	0	13.43	-	16.23	5s 3D	-	4p 5D	2	-	1
442.3728	Cu II	1	14.34	-	17.14	4d 3G	-	4f 3G	4	-	3
442.7981	Cu II	2	11.85	-	14.65	4s2 1G	-	4p 3F	4	-	3
443.1911	Cu II	10	14.34	-	17.14	4d 3G	-	4f 1G	4	-	4
443.5691	Cu II	4	14.34	-	17.13	4d 3P	-	4f 3F	2	-	2
443.9639	Cu II	0	14.64	-	17.43	4p 3D	-	5d 1S	1	-	0
444.0883	Cu II	11	14.34	-	17.13	4d 3P	-	4f 3F	1	-	2
444.1717	Cu II	3	14.43	-	17.22	4p 3D	-	5d 1G	3	-	4
444.1896	Cu II	0	14.33	-	17.12	4d 3G	-	4f 3H	5	-	4
444.4124	Cu II	2	14.33	-	17.12	4d 3G	-	4f 1H	5	-	5
444.4831	Cu II	27	14.34	-	17.13	4d 3P	-	4f 1F	2	-	3
444.7329	Cu II	0	14.53	-	17.32	4d 1P	-	6p 3D	1	-	2
444.8045	Cu II	0	14.46	-	17.25	4p 3G	-	5d 1F	3	-	3
444.9531	Cu II	1	14.34	-	17.13	4d 3G	-	4f 1F	4	-	3
445.4350	Cu II	0	14.43	-	17.21	4p 3D	-	5d 3G	3	-	3
446.0439	Cu II	0	14.34	-	17.12	4d 3G	-	4f 3H	4	-	4
446.2686	Cu II	80	14.34	-	17.12	4d 3G	-	4f 1H	4	-	5
446.5110	Cu II	1	14.34	-	17.11	4d 3P	-	4f 3D	2	-	1
446.8708	Cu II	1	13.39	-	16.17	5s 3D	-	4p 3P	3	-	2
446.9036	Cu II	9	14.33	-	17.10	4d 3G	-	4p 3P	5	-	4
447.0202	Cu II	4	14.34	-	17.11	4d 3P	-	4f 1D	2	-	2
447.0371	Cu II	2	14.34	-	17.11	4d 3P	-	4f 3D	1	-	1

447.5476	Cu II	1	14.34	-	17.11	4d 3P	-	4f 1D	1	-	2
448.5280	Cu II	0	14.20	-	16.96	4d 3S	-	4p 3F	1	-	2
448.7807	Cu II	1	14.34	-	17.10	4d 3G	-	4p 3P	4	-	4
448.8150	Cu II	0	14.34	-	17.10	4d 3P	-	4p 3S	2	-	1
449.3466	Cu II	1	14.34	-	17.10	4d 3P	-	4p 3S	1	-	1
449.5353	Cu II	1	14.46	-	17.22	4p 3G	-	5d 1G	3	-	4
449.8552	Cu II	0	15.94	-	18.69	4p 3F	-	8s 3D	3	-	2
450.6002	Cu II	0	-		-		-				
450.7026	Cu II	1	14.39	-	17.14	4d 3D	-	4f 3G	3	-	3
450.8294	Cu II	0	14.46	-	17.21	4p 3G	-	5d 3G	3	-	3
451.5520	Cu II	53	14.39	-	17.14	4d 3D	-	4f 1G	3	-	4
451.6049	Cu II	5	15.22	-	17.96	5p 3F	-	7s 3D	2	-	2
452.3416	Cu II	0	11.85	-	14.59	4s2 1G	-	4p 3F	4	-	4
452.4303	Cu II	1	14.39	-	17.13	4d 3D	-	4f 3F	3	-	2
452.8528	Cu II	1	14.42	-	17.16	4d 3F	-	4p 3G	3	-	3
452.9979	Cu II	1	15.22	-	17.95	5p 3F	-	7s 3D	2	-	3
453.3714	Cu II	0	13.43	-	16.17	5s 3D	-	4p 3P	2	-	2
453.3813	Cu II	4	14.39	-	17.13	4d 3D	-	4f 1F	3	-	3
454.1032	Cu II	10	15.23	-	17.96	5p 1P	-	7s 3D	1	-	2
454.1256	Cu II	1	14.43	-	17.16	4d 3D	-	4p 3G	2	-	3
454.2117	Cu II	1	14.34	-	17.07	4d 3P	-	4p 1D	2	-	2
454.2171	Cu II	1	14.53	-	17.26	4d 1P	-	6p 3P	1	-	1
454.5138	Cu II	1	14.39	-	17.12	4d 3D	-	4f 3H	3	-	4
454.5470	Cu II	0	14.60	-	17.33	4d 3G	-	6p 3D	3	-	3
454.6436	Cu II	4	14.52	-	17.25	4p 3D	-	5d 1F	2	-	3
454.7562	Cu II	1	14.34	-	17.07	4d 3P	-	4p 1D	1	-	2
455.1451	Cu II	0	15.97	-	18.69	4p 3F	-	8s 3D	4	-	3
455.1805	Cu II	4	14.60	-	17.32	4d 3G	-	6p 3D	3	-	2
455.5920	Cu II	0	-		-		-				
455.7508	Cu II	51	14.42	-	17.14	4d 3F	-	4f 3G	3	-	3
456.0212	Cu II	0	14.39	-	17.11	4d 3D	-	4f 1D	3	-	2
456.6193	Cu II	1	14.42	-	17.14	4d 3F	-	4f 1G	3	-	4
456.6933	Cu II	1	14.43	-	17.14	4d 3F	-	4f 3G	4	-	3
456.7254	Cu II	0	15.98	-	18.69	4p 3D	-	8s 3D	2	-	3
457.0400	Cu II	5	14.43	-	17.14	4d 3D	-	4f 3G	2	-	3
457.1995	Cu II	1	15.25	-	17.96	5p 1F	-	7s 3D	3	-	2
457.3559	Cu II	1	14.39	-	17.10	4d 3D	-	4p 3P	3	-	4
457.3701	Cu II	3	14.62	-	17.33	4d 1G	-	6p 3D	4	-	3
457.5175	Cu II	4	14.42	-	17.13	4d 3F	-	4f 3F	3	-	2
457.5189	Cu II	2	14.52	-	17.23	4p 3D	-	5d 1D	2	-	2
457.5654	Cu II	33	14.43	-	17.14	4d 3F	-	4f 1G	4	-	4
458.6273	Cu II	2	15.25	-	17.95	5p 1F	-	7s 3D	3	-	3

458.8167	Cu II	12	14.43	-	17.13	4d 3D	-	4f 3F	2	-	2
458.9602	Cu II	1	14.62	-	17.32	4d 3D	-	6p 3D	1	-	2
459.2614	Cu II	0	14.52	-	17.22	4p 3D	-	5d 3D	2	-	1
459.4438	Cu II	4	14.43	-	17.13	4d 3F	-	4f 1F	4	-	3
459.6482	Cu II	305	14.42	-	17.12	4d 3F	-	4f 3H	3	-	4
459.6906	Cu II	0		-			-			-	
459.7947	Cu II	130	14.43	-	17.13	4d 3D	-	4f 1F	2	-	3
460.6069	Cu II	5	14.43	-	17.12	4d 3F	-	4f 3H	4	-	4
460.8465	Cu II	174	14.43	-	17.12	4d 3F	-	4f 1H	4	-	5
460.9397	Cu II	4	14.52	-	17.21	4p 3D	-	5d 3G	2	-	3
460.9510	Cu II	0	15.53	-	18.22	4p 5P	-	7s 1D	2	-	2
461.0205	Cu II	0	14.30	-	16.99	4p 3G	-	5d 3F	4	-	4
461.4337	Cu II	0	16.01	-	18.69	4p 3D	-	8s 3D	3	-	2
461.9650	Cu II	4	14.43	-	17.11	4d 3D	-	4f 3D	2	-	1
462.5102	Cu II	30	14.43	-	17.11	4d 3D	-	4f 1D	2	-	2
462.5551	Cu II	0	14.42	-	17.10	4d 3F	-	4p 3P	3	-	4
463.1805	Cu II	0	14.60	-	17.28	4d 3G	-	6p 3F	3	-	3
463.3017	Cu II	0	15.29	-	17.96	5p 3D	-	7s 3D	1	-	2
463.3087	Cu II	0	14.65	-	17.33	4d 1D	-	6p 3D	2	-	3
463.5076	Cu II	0	14.39	-	17.07	4d 3D	-	4p 1D	3	-	2
463.5259	Cu II	3	14.43	-	17.10	4d 3F	-	4p 3P	4	-	4
463.9585	Cu II	1	14.34	-	17.01	4d 3P	-	4p 1P	1	-	1
463.9668	Cu II	0	14.65	-	17.32	4d 1D	-	6p 3D	2	-	2
464.4317	Cu II	1	14.43	-	17.10	4d 3D	-	4p 3S	2	-	1
464.7116	Cu II	0	14.62	-	17.28	4d 1G	-	6p 3F	4	-	4
464.9271	Cu II	0	14.20	-	16.86	4d 3S	-	4f 3D	1	-	2
465.7942	Cu II	1	14.52	-	17.18	4p 3D	-	5d 1P	2	-	1
466.0307	Cu II	495	14.45	-	17.11	4d 3P	-	4f 3D	0	-	1
466.1122	Cu II	3	14.62	-	17.28	4d 1G	-	6p 3F	4	-	3
466.1363	Cu II	13	14.20	-	16.86	4d 3S	-	4f 1P	1	-	1
466.2649	Cu II	1	14.20	-	16.86	4d 3S	-	4p 5S	1	-	2
466.3007	Cu II	0	14.34	-	17.00	4d 3P	-	4p 3G	2	-	3
466.6326	Cu II	1	13.43	-	16.09	5s 3D	-	4p 3P	2	-	1
467.1702	Cu II	1205	14.20	-	16.85	4d 3S	-	4f 3P	1	-	2
467.3577	Cu II	611	14.20	-	16.85	4d 3S	-	4f 3P	1	-	0
467.8953	Cu II	1	14.30	-	16.95	4p 3G	-	5d 3G	4	-	5
468.1994	Cu II	1439	14.20	-	16.85	4d 3S	-	4f 3P	1	-	1
468.5411	Cu II	1	14.45	-	17.10	4d 3P	-	4p 3S	0	-	1
468.7764	Cu II	0	15.32	-	17.96	5p 1D	-	7s 3D	2	-	2
468.8485	Cu II	1	14.42	-	17.07	4d 3F	-	4p 1D	3	-	2
469.0678	Cu II	1	14.62	-	17.26	4d 3D	-	6p 3P	1	-	1
469.7633	Cu II	1	14.60	-	17.24	4d 3G	-	4p 3D	3	-	2

470.2129	Cu II	1	14.43	-	17.07	4d 3D	-	4p 1D	2	-	2
470.2776	Cu II	2	15.32	-	17.95	5p 1D	-	7s 3D	2	-	3
470.2952	Cu II	1	14.69	-	17.33	4d 1F	-	6p 3D	3	-	3
471.2043	Cu II	1	14.59	-	17.22	4p 3F	-	5d 1G	4	-	4
471.3616	Cu II	0	14.70	-	17.33	4d 3F	-	6p 3D	2	-	3
472.0428	Cu II	2	14.70	-	17.32	4d 3F	-	6p 3D	2	-	2
472.2815	Cu II	0	14.65	-	17.28	4d 1D	-	6p 3F	2	-	3
472.4279	Cu II	1	14.34	-	16.96	4d 3P	-	4p 3F	2	-	2
473.0169	Cu II	2	14.34	-	16.96	4d 3P	-	4p 3F	1	-	2
473.7540	Cu II	1	13.39	-	16.01	5s 3D	-	4p 3D	3	-	3
474.2886	Cu II	0	11.85	-	14.46	4s2 1G	-	4p 3G	4	-	3
474.2987	Cu II	1	14.65	-	17.26	4d 1D	-	6p 3P	2	-	1
474.4592	Cu II	10	14.64	-	17.25	4p 3D	-	5d 3F	1	-	2
475.8433	Cu II	0	-		-		-				
476.0780	Cu II	0	14.65	-	17.25	4p 3F	-	5d 3F	3	-	2
476.1034	Cu II	1	14.39	-	17.00	4d 3D	-	4p 3G	3	-	3
476.5913	Cu II	1	14.65	-	17.25	4p 3F	-	5d 1F	3	-	3
476.6740	Cu II	0	14.20	-	16.80	4d 3S	-	4p 3P	1	-	0
477.2787	Cu II	1	13.68	-	16.28	5s 1D	-	4p 5D	2	-	3
477.2966	Cu II	1	14.53	-	17.13	4d 1P	-	4f 3F	1	-	2
478.0146	Cu II	0	10.96	-	13.55	4s2 3P	-	4p 5D	2	-	3
478.1080	Cu II	4	14.64	-	17.23	4p 3D	-	5d 1D	1	-	2
479.2712	Cu II	1	13.65	-	16.24	5s 3D	-	4p 5D	1	-	2
479.5434	Cu II	1	14.69	-	17.28	4d 1F	-	6p 3F	3	-	3
479.7308	Cu II	2	13.39	-	15.98	5s 3D	-	4p 3D	3	-	2
479.7519	Cu II	0	14.65	-	17.23	4p 3F	-	5d 1D	3	-	2
480.0112	Cu II	6	14.64	-	17.22	4p 3D	-	5d 3D	1	-	1
480.0582	Cu II	1	14.43	-	17.01	4d 3D	-	4p 1P	2	-	1
480.4092	Cu II	0	11.85	-	14.43	4s2 1G	-	4p 3D	4	-	3
480.5653	Cu II	1	14.34	-	16.92	4d 3P	-	4p 3D	2	-	3
480.6522	Cu II	0	14.70	-	17.28	4d 3F	-	6p 3F	2	-	3
480.7046	Cu II	564	14.53	-	17.11	4d 1P	-	4f 3D	1	-	1
481.0666	Cu II	1	13.43	-	16.01	5s 3D	-	4p 3D	2	-	3
481.1147	Cu II	0	14.34	-	16.92	4d 3G	-	4p 3D	4	-	3
481.2948	Cu II	2613	14.53	-	17.11	4d 1P	-	4f 1D	1	-	2
481.4868	Cu II	5	13.39	-	15.97	5s 3D	-	4p 3F	3	-	4
482.0265	Cu II	1	14.65	-	17.22	4p 3F	-	5d 1G	3	-	4
482.4926	Cu II	0	14.39	-	16.96	4d 3D	-	4p 3F	3	-	2
482.7416	Cu II	1	14.70	-	17.26	4d 3F	-	6p 3P	2	-	1
482.7933	Cu II	0	14.43	-	17.00	4d 3F	-	4p 3G	4	-	3
482.9335	Cu II	87	14.33	-	16.90	4d 3G	-	4f 3G	5	-	4

482.9980	Cu II	0	14.43	-	17.00	4p 3D	-	5d 3D	3	-	2
483.3760	Cu II	1	14.53	-	17.10	4d 1P	-	4p 3S	1	-	1
483.5146	Cu II	0	14.65	-	17.21	4p 3F	-	5d 3G	3	-	3
483.6799	Cu II	17	14.43	-	16.99	4p 3D	-	5d 3F	3	-	4
483.9067	Cu II	0	14.43	-	16.99	4p 3D	-	5d 3F	3	-	3
484.4500	Cu II	0	14.45	-	17.01	4d 3P	-	4p 1P	0	-	1
484.7382	Cu II	1	14.20	-	16.75	4d 3S	-	4p 3D	1	-	1
485.1262	Cu II	48	14.34	-	16.90	4d 3G	-	4f 3G	4	-	4
485.4988	Cu II	1567	14.33	-	16.88	4d 3G	-	4f 3G	5	-	5
485.9070	Cu II	0	13.68	-	16.23	5s 1D	-	4p 5D	2	-	1
486.1561	Cu II	72	14.33	-	16.88	4d 3G	-	4f 3F	5	-	4
486.3803	Cu II	1	13.39	-	15.94	5s 3D	-	4p 3D	3	-	2
486.6130	Cu II	1	13.39	-	15.94	5s 3D	-	4p 3F	3	-	3
486.6256	Cu II	11	14.43	-	16.98	4p 3D	-	5d 3D	3	-	3
487.1523	Cu II	0	14.64	-	17.18	4p 3D	-	5d 1P	1	-	1
487.2205	Cu II	2	14.62	-	17.16	4d 1G	-	4p 3G	4	-	3
487.2306	Cu II	1	13.43	-	15.98	5s 3D	-	4p 3D	2	-	2
487.3304	Cu II	727	14.60	-	17.14	4d 3G	-	4f 3G	3	-	3
487.7149	Cu II	15	14.34	-	16.88	4d 3G	-	4f 3G	4	-	5
487.7447	Cu II	0	14.70	-	17.24	4d 3F	-	4p 3D	2	-	2
488.2826	Cu II	4	14.42	-	16.96	4d 3F	-	4p 3F	3	-	2
488.3235	Cu II	24	14.60	-	17.14	4d 3G	-	4f 1G	3	-	4
488.3783	Cu II	1331	14.34	-	16.88	4d 3G	-	4f 3F	4	-	4
489.3469	Cu II	0	14.46	-	17.00	4p 3G	-	5d 3D	3	-	2
489.3508	Cu II	36	14.60	-	17.13	4d 3G	-	4f 3F	3	-	2
489.6416	Cu II	1	14.53	-	17.07	4d 1P	-	4p 1D	1	-	2
489.7627	Cu II	6	14.43	-	16.96	4d 3D	-	4p 3F	2	-	2
490.0469	Cu II	8	14.46	-	16.99	4p 3G	-	5d 3F	3	-	4
490.1427	Cu II	119	14.34	-	16.87	4d 3P	-	4f 3D	2	-	3
490.2797	Cu II	1	14.46	-	16.99	4p 3G	-	5d 3F	3	-	3
490.3008	Cu II	1	14.43	-	16.96	4p 3D	-	5d 3G	3	-	4
490.5715	Cu II	4	14.43	-	16.96	4p 3D	-	5d 3P	3	-	2
490.5767	Cu II	25	14.62	-	17.14	4d 1G	-	4f 3G	4	-	3
490.6567	Cu II	440	14.34	-	16.86	4d 3P	-	4f 3D	2	-	2
490.7142	Cu II	118	14.34	-	16.87	4d 3G	-	4f 3D	4	-	3
490.9039	Cu II	168	14.33	-	16.85	4d 3G	-	4f 3H	5	-	5
490.9836	Cu II	1	14.39	-	16.92	4d 3D	-	4p 3D	3	-	3
491.1063	Cu II	0	16.17	-	18.69	4p 3P	-	8s 3D	2	-	3
491.2366	Cu II	2297	14.34	-	16.86	4d 3P	-	4f 3F	2	-	3
491.2921	Cu II	1722	14.34	-	16.86	4d 3P	-	4f 3D	1	-	2
491.5832	Cu II	927	14.62	-	17.14	4d 1G	-	4f 1G	4	-	4
491.7892	Cu II	6532	14.60	-	17.12	4d 3G	-	4f 3H	3	-	4

491.8106	Cu II	6	14.34	-	16.86	4d 3G	-	4f 3F	4	-	3
491.8376	Cu II	0		-			-			-	
492.0036	Cu II	104	14.34	-	16.86	4d 3P	-	4f 1P	2	-	1
492.1469	Cu II	1	14.34	-	16.86	4d 3P	-	4p 5S	2	-	2
492.6424	Cu II	1178	14.34	-	16.86	4d 3P	-	4f 1P	1	-	1
492.6819	Cu II	0	14.05	-	16.56	4p 5F	-	6s 3D	4	-	3
492.7861	Cu II	0	14.34	-	16.86	4d 3P	-	4p 5S	1	-	2
493.0709	Cu II	7	14.46	-	16.98	4p 3G	-	5d 3D	3	-	3
493.1556	Cu II	1837	14.34	-	16.85	4d 3P	-	4f 3P	2	-	2
493.1698	Cu II	8223	14.34	-	16.85	4d 3G	-	4f 3H	4	-	5
493.3075	Cu II	1	14.33	-	16.84	4d 3G	-	4p 3F	5	-	4
493.5545	Cu II	0	14.60	-	17.11	4d 3G	-	4f 1D	3	-	2
493.7221	Cu II	2619	14.62	-	17.13	4d 3D	-	4f 3F	1	-	2
493.7520	Cu II	50	14.62	-	17.13	4d 1G	-	4f 1F	4	-	3
493.7975	Cu II	201	14.34	-	16.85	4d 3P	-	4f 3P	1	-	2
493.9653	Cu II	0	14.65	-	17.16	4d 1D	-	4p 3G	2	-	3
494.0070	Cu II	160	14.34	-	16.85	4d 3P	-	4f 3P	1	-	0
494.0911	Cu II	0	13.43	-	15.94	5s 3D	-	4p 3D	2	-	2
494.3026	Cu II	631	14.34	-	16.85	4d 3P	-	4f 3P	2	-	1
494.3312	Cu II	2	13.43	-	15.94	5s 3D	-	4p 3F	2	-	3
494.9474	Cu II	4	14.34	-	16.85	4d 3P	-	4f 3P	1	-	1
495.0955	Cu II	200	14.62	-	17.12	4d 1G	-	4f 3H	4	-	4
495.1621	Cu II	3829	14.39	-	16.90	4d 3D	-	4f 3G	3	-	4
495.3724	Cu II	8280	14.62	-	17.12	4d 1G	-	4f 1H	4	-	5
495.5956	Cu II	0	14.34	-	16.84	4d 3G	-	4p 3F	4	-	4
495.7879	Cu II	1	14.20	-	16.70	4d 3S	-	4p 3D	1	-	2
496.8445	Cu II	1	14.46	-	16.96	4p 3G	-	5d 3G	3	-	4
496.9805	Cu II	0	14.42	-	16.92	4d 3F	-	4p 3D	3	-	3
497.1225	Cu II	3	14.46	-	16.96	4p 3G	-	5d 3P	3	-	2
497.3142	Cu II	8	14.76	-	17.25	4p 3F	-	5d 3F	2	-	2
497.3695	Cu II	509	14.62	-	17.11	4d 3D	-	4f 3D	1	-	1
497.4154	Cu II	382	14.65	-	17.14	4d 1D	-	4f 3G	2	-	3
497.5066	Cu II	4	14.34	-	16.83	4d 3P	-	4p 3F	2	-	3
497.7905	Cu II	0	15.94	-	18.43	4p 3F	-	6d 3F	3	-	2
497.8743	Cu II	1	14.76	-	17.25	4p 3F	-	5d 1F	2	-	3
498.0014	Cu II	122	14.62	-	17.11	4d 3D	-	4f 1D	1	-	2
498.0729	Cu II	1	15.94	-	18.43	4p 3F	-	6d 1F	3	-	3
498.0955	Cu II	0	14.34	-	16.83	4d 3G	-	4p 3F	4	-	3
498.1014	Cu II	2	14.43	-	16.92	4d 3F	-	4p 3D	4	-	3
498.3170	Cu II	0	15.94	-	18.43	4p 3D	-	6d 1F	2	-	3
498.4696	Cu II	0	14.62	-	17.10	4d 1G	-	4p 3P	4	-	4
498.5139	Cu II	0	14.43	-	16.92	4d 3D	-	4p 3D	2	-	3

498.5506	Cu II	556	14.39	-	16.88	4d 3D	-	4f 3F	3	-	4
499.3553	Cu II	1	13.68	-	16.17	5s 1D	-	4p 3P	2	-	2
499.5206	Cu II	59	14.65	-	17.13	4d 1D	-	4f 3F	2	-	2
500.0904	Cu II	0	15.94	-	18.42	4p 3D	-	6d 1D	2	-	2
500.2300	Cu II	5	14.62	-	17.10	4d 3D	-	4p 3S	1	-	1
500.3265	Cu II	2	14.53	-	17.01	4d 1P	-	4p 1P	1	-	1
500.6801	Cu II	3767	14.65	-	17.13	4d 1D	-	4f 1F	2	-	3
500.9569	Cu II	1	15.94	-	18.41	4p 3F	-	6d 1G	3	-	4
500.9851	Cu II	183	14.39	-	16.87	4d 3D	-	4f 3D	3	-	3
501.0525	Cu II	0	15.94	-	18.41	4p 3D	-	6d 3D	2	-	1
501.2620	Cu II	1055	14.42	-	16.90	4d 3F	-	4f 3G	3	-	4
501.2816	Cu II	12	14.52	-	17.00	4p 3D	-	5d 3D	2	-	2
501.3245	Cu II	1	14.76	-	17.23	4p 3F	-	5d 1D	2	-	2
501.5220	Cu II	123	14.39	-	16.86	4d 3D	-	4f 3D	3	-	2
501.8064	Cu II	0	15.94	-	18.41	4p 3F	-	6d 3G	3	-	3
501.9149	Cu II	1	14.69	-	17.16	4d 1F	-	4p 3G	3	-	3
502.0541	Cu II	1	15.94	-	18.41	4p 3D	-	6d 3G	2	-	3
502.1279	Cu II	2366	14.39	-	16.86	4d 3D	-	4f 3F	3	-	3
502.2604	Cu II	17	14.52	-	16.99	4p 3D	-	5d 3F	2	-	3
502.3358	Cu II	0	14.60	-	17.07	4d 3G	-	4p 1D	3	-	2
502.4023	Cu II	1803	14.43	-	16.90	4d 3F	-	4f 3G	4	-	4
502.5853	Cu II	0	14.75	-	17.22	4p 1G	-	5d 1G	4	-	4
503.0791	Cu II	1	14.39	-	16.86	4d 3D	-	4p 5S	3	-	2
503.1297	Cu II	0	14.70	-	17.16	4d 3F	-	4p 3G	2	-	3
503.2546	Cu II	64	14.65	-	17.11	4d 1D	-	4f 3D	2	-	1
503.4173	Cu II	2	14.76	-	17.22	4p 3F	-	5d 3D	2	-	1
503.6731	Cu II	1	14.20	-	16.66	4d 3S	-	4p 3P	1	-	1
503.9016	Cu II	908	14.65	-	17.11	4d 1D	-	4f 1D	2	-	2
504.1331	Cu II	437	14.39	-	16.85	4d 3D	-	4f 3P	3	-	2
504.4278	Cu II	0	14.34	-	16.80	4d 3P	-	4p 3P	1	-	0
504.7348	Cu II	4286	14.42	-	16.88	4d 3F	-	4f 3F	3	-	4
505.0655	Cu II	0	11.85	-	14.30	4s2 1G	-	4p 3G	4	-	4
505.1793	Cu II	6715	14.43	-	16.88	4d 3F	-	4f 3G	4	-	5
505.1901	Cu II	1	14.52	-	16.98	4p 3D	-	5d 3D	2	-	3
505.2046	Cu II	1	15.98	-	18.43	4p 3D	-	6d 3F	2	-	2
505.4347	Cu II	23	14.76	-	17.21	4p 3F	-	5d 3G	2	-	3
505.4773	Cu II	172	14.69	-	17.14	4d 1F	-	4f 3G	3	-	3
505.4955	Cu II	0	15.98	-	18.43	4p 3D	-	6d 1F	2	-	3
505.8910	Cu II	431	14.43	-	16.88	4d 3F	-	4f 3F	4	-	4
506.0740	Cu II	0	14.39	-	16.84	4d 3D	-	4p 3F	3	-	4
506.1833	Cu II	4	14.65	-	17.10	4d 1D	-	4p 3S	2	-	1

506.5459	Cu II	5808	14.69	-	17.14	4d 1F	-	4f 1G	3	-	4
506.7094	Cu II	3982	14.70	-	17.14	4d 3F	-	4f 3G	2	-	3
506.7629	Cu II	0	14.13	-	16.58	4p 5F	-	6s 3D	3	-	2
506.9431	Cu II	1	14.62	-	17.07	4d 3D	-	4p 1D	1	-	2
507.2302	Cu II	1578	14.42	-	16.87	4d 3F	-	4f 3D	3	-	3
507.6515	Cu II	59	14.69	-	17.13	4d 1F	-	4f 3F	3	-	2
507.7807	Cu II	185	14.42	-	16.86	4d 3F	-	4f 3D	3	-	2
508.1106	Cu II	1	13.65	-	16.09	5s 3D	-	4p 3P	1	-	1
508.2526	Cu II	29	14.99	-	17.43	5p 3P	-	5d 1S	1	-	0
508.3106	Cu II	0	15.98	-	18.41	4p 3D	-	6d 3D	2	-	1
508.3979	Cu II	35	14.43	-	16.87	4d 3F	-	4f 3D	4	-	3
508.4018	Cu II	23	14.42	-	16.86	4d 3F	-	4f 3F	3	-	3
508.6810	Cu II	10	14.39	-	16.83	4d 3D	-	4p 3F	3	-	3
508.8277	Cu II	3077	14.43	-	16.87	4d 3D	-	4f 3D	2	-	3
508.8490	Cu II	1256	14.69	-	17.13	4d 1F	-	4f 1F	3	-	3
508.8942	Cu II	1057	14.70	-	17.13	4d 3F	-	4f 3F	2	-	2
508.9498	Cu II	6	14.52	-	16.96	4p 3D	-	5d 3P	2	-	1
509.3415	Cu II	1	15.98	-	18.41	4p 3D	-	6d 3G	2	-	3
509.3816	Cu II	1334	14.43	-	16.86	4d 3D	-	4f 3D	2	-	2
509.4442	Cu II	3	14.52	-	16.96	4p 3D	-	5d 3P	2	-	2
509.5749	Cu II	284	14.43	-	16.86	4d 3F	-	4f 3F	4	-	3
510.0067	Cu II	350	14.43	-	16.86	4d 3D	-	4f 3F	2	-	3
510.0977	Cu II	120	14.70	-	17.13	4d 3F	-	4f 1F	2	-	3
510.2761	Cu II	1	14.69	-	17.12	4d 1F	-	4f 3H	3	-	4
510.4576	Cu II	1	14.42	-	16.85	4d 3F	-	4f 3P	3	-	2
510.8334	Cu II	183	14.43	-	16.86	4d 3D	-	4f 1P	2	-	1
510.8768	Cu II	1	14.53	-	16.96	4d 1P	-	4p 3F	1	-	2
511.0341	Cu II	122	14.43	-	16.85	4d 3F	-	4f 3H	4	-	5
511.2775	Cu II	1	14.76	-	17.18	4p 3F	-	5d 1P	2	-	1
511.5537	Cu II	3	14.64	-	17.06	4p 3D	-	5d 3P	1	-	0
512.0754	Cu II	170	14.43	-	16.85	4d 3D	-	4f 3P	2	-	2
512.1769	Cu II	73	14.69	-	17.11	4d 1F	-	4f 1D	3	-	2
512.3058	Cu II	0	16.01	-	18.43	4p 3D	-	6d 1F	3	-	3
512.4476	Cu II	0	-		-		-				
512.4806	Cu II	0	15.98	-	18.39	4p 3D	-	6d 1P	2	-	1
512.7702	Cu II	84	14.70	-	17.11	4d 3F	-	4f 3D	2	-	1
512.7733	Cu II	0	14.34	-	16.75	4d 3P	-	4p 3D	2	-	1
513.0583	Cu II	2	14.65	-	17.07	4d 1D	-	4p 1D	2	-	2
513.3122	Cu II	1	14.43	-	16.85	4d 3D	-	4f 3P	2	-	1
513.4419	Cu II	41	14.70	-	17.11	4d 3F	-	4f 1D	2	-	2
513.4673	Cu II	1	14.34	-	16.75	4d 3P	-	4p 3D	1	-	1
513.6394	Cu II	0	14.43	-	16.84	4d 3F	-	4p 3F	4	-	4

514.5120	Cu II	0	16.28	-	18.69	4p 5D	-	8s 3D	3	-	3
515.1207	Cu II	0	14.42	-	16.83	4d 3F	-	4p 3F	3	-	3
515.3574	Cu II	0	16.01	-	18.41	4p 3D	-	6d 1G	3	-	4
515.7258	Cu II	1	14.43	-	16.83	4p 3D	-	6s 1D	3	-	2
515.8093	Cu II	450	14.45	-	16.86	4d 3P	-	4f 1P	0	-	1
515.8111	Cu II	8	14.70	-	17.10	4d 3F	-	4p 3S	2	-	1
515.9097	Cu II	23	14.59	-	16.99	4p 3F	-	5d 3F	4	-	4
516.1678	Cu II	1	14.59	-	16.99	4p 3F	-	5d 3F	4	-	3
516.3251	Cu II	11	14.43	-	16.83	4d 3F	-	4p 3F	4	-	3
516.7683	Cu II	0	14.43	-	16.83	4d 3D	-	4p 3F	2	-	3
517.5965	Cu II	3	14.20	-	16.59	4d 3S	-	4p 3P	1	-	2
518.3367	Cu II	144	14.45	-	16.85	4d 3P	-	4f 3P	0	-	1
518.4053	Cu II	1	14.62	-	17.01	4d 3D	-	4p 1P	1	-	1
519.2624	Cu II	5	14.59	-	16.98	4p 3F	-	5d 3D	4	-	3
520.5107	Cu II	2	14.52	-	16.90	4p 3D	-	5d 3S	2	-	1
520.8215	Cu II	1	14.62	-	17.00	4d 1G	-	4p 3G	4	-	3
521.3980	Cu II	2	14.33	-	16.71	4d 3G	-	4p 3H	5	-	4
521.6396	Cu II	4	14.69	-	17.07	4d 1F	-	4p 1D	3	-	2
522.9519	Cu II	1	14.70	-	17.07	4d 3F	-	4p 1D	2	-	2
522.9707	Cu II	1	14.46	-	16.83	4p 3G	-	6s 1D	3	-	2
523.4493	Cu II	4	14.59	-	16.96	4p 3F	-	5d 3G	4	-	4
523.9549	Cu II	0	14.34	-	16.71	4d 3G	-	4p 3H	4	-	4
524.5342	Cu II	87	14.59	-	16.95	4p 3F	-	5d 3G	4	-	5
524.7114	Cu II	3	14.60	-	16.96	4d 3G	-	4p 3F	3	-	2
524.7976	Cu II	3	14.89	-	17.25	5p 3P	-	5d 3F	2	-	2
524.8019	Cu II	2	14.65	-	17.01	4d 1D	-	4p 1P	2	-	1
525.1544	Cu II	1	14.34	-	16.70	4d 3P	-	4p 3D	2	-	2
525.4214	Cu II	23	14.89	-	17.25	5p 3P	-	5d 1F	2	-	3
526.1046	Cu II	3	14.64	-	17.00	4p 3D	-	5d 3D	1	-	2
526.9991	Cu II	0	-		-		-				
527.6525	Cu II	0	-		-		-				
528.0958	Cu II	5	14.65	-	17.00	4p 3F	-	5d 3D	3	-	2
528.9110	Cu II	6	14.65	-	16.99	4p 3F	-	5d 3F	3	-	4
529.1823	Cu II	32	14.65	-	16.99	4p 3F	-	5d 3F	3	-	3
529.2654	Cu II	6	14.89	-	17.23	5p 3P	-	5d 1D	2	-	2
529.4369	Cu II	0	16.09	-	18.43	4p 3P	-	6d 3F	1	-	2
529.7404	Cu II	1	14.62	-	16.96	4d 3D	-	4p 3F	1	-	2
531.5987	Cu II	2	14.89	-	17.22	5p 3P	-	5d 3D	2	-	1
531.7610	Cu II	0	16.09	-	18.42	4p 3P	-	6d 1D	1	-	2
532.2606	Cu II	0	14.53	-	16.86	4d 1P	-	4f 3D	1	-	2
532.4355	Cu II	14	14.65	-	16.98	4p 3F	-	5d 3D	3	-	3
532.6292	Cu II	0	13.65	-	15.98	5s 3D	-	4p 3D	1	-	2

533.2596	Cu II	2	14.43	-	16.75	4d 3D	-	4p 3D	2	-	1
533.8459	Cu II	1	14.53	-	16.86	4d 1P	-	4f 1P	1	-	1
533.8487	Cu II	1	14.89	-	17.21	5p 3P	-	5d 3G	2	-	3
534.0097	Cu II	0	14.34	-	16.66	4d 3P	-	4p 3P	2	-	1
534.5574	Cu II	3	14.64	-	16.96	4p 3D	-	5d 3P	1	-	1
534.7624	Cu II	1	14.34	-	16.66	4d 3P	-	4p 3P	1	-	1
535.1029	Cu II	0	14.64	-	16.96	4p 3D	-	5d 3P	1	-	2
535.1581	Cu II	1	14.70	-	17.01	4d 3F	-	4p 1P	2	-	1
535.2025	Cu II	46	14.53	-	16.85	4d 1P	-	4f 3P	1	-	2
535.4486	Cu II	36	14.53	-	16.85	4d 1P	-	4f 3P	1	-	0
535.6809	Cu II	0	14.39	-	16.71	4d 3D	-	4p 3H	3	-	4
536.4216	Cu II	3	14.65	-	16.96	4d 1D	-	4p 3F	2	-	2
536.5537	Cu II	76	14.53	-	16.85	4d 1P	-	4f 3P	1	-	1
536.6246	Cu II	1	14.52	-	16.83	4p 3D	-	6s 1D	2	-	2
536.8383	Cu II	98	14.65	-	16.96	4p 3F	-	5d 3G	3	-	4
537.1628	Cu II	9	14.65	-	16.96	4p 3F	-	5d 3P	3	-	2
537.6207	Cu II	1	14.39	-	16.70	4d 3D	-	4p 3D	3	-	2
537.6475	Cu II	0	14.69	-	17.00	4d 1F	-	4p 3G	3	-	3
538.6806	Cu II	0	14.62	-	16.92	4d 1G	-	4p 3D	4	-	3
538.6843	Cu II	1	14.45	-	16.75	4d 3P	-	4p 3D	0	-	1
539.3938	Cu II	2	14.52	-	16.82	4p 3D	-	6s 3D	2	-	1
539.7295	Cu II	38	14.60	-	16.90	4d 3G	-	4f 3G	3	-	4
540.3711	Cu II	11	14.89	-	17.18	5p 3P	-	5d 1P	2	-	1
540.7445	Cu II	4	13.68	-	15.98	5s 1D	-	4p 3D	2	-	2
540.8385	Cu II	7	13.65	-	15.94	5s 3D	-	4p 3D	1	-	2
541.6398	Cu II	1	14.96	-	17.25	5p 3F	-	5d 3F	3	-	2
542.3042	Cu II	3	14.96	-	17.25	5p 3F	-	5d 1F	3	-	3
542.8272	Cu II	0	14.42	-	16.71	4d 3F	-	4p 3H	3	-	4
543.7144	Cu II	45	14.62	-	16.90	4d 1G	-	4f 3G	4	-	4
543.7579	Cu II	165	14.60	-	16.88	4d 3G	-	4f 3F	3	-	4
544.0071	Cu II	1	15.94	-	18.22	4p 3F	-	7s 1D	3	-	2
544.1648	Cu II	1	14.43	-	16.71	4d 3F	-	4p 3H	4	-	4
544.8193	Cu II	1	14.42	-	16.70	4d 3F	-	4p 3D	3	-	2
544.9406	Cu II	4	14.34	-	16.61	4d 3P	-	4p 3D	2	-	3
545.4136	Cu II	1	14.98	-	17.25	5p 3F	-	5d 1F	4	-	3
545.6472	Cu II	0	14.34	-	16.61	4d 3G	-	4p 3D	4	-	3
545.7888	Cu II	1	15.94	-	18.21	4p 3D	-	7s 3D	2	-	1
545.8094	Cu II	8	14.69	-	16.96	4d 1F	-	4p 3F	3	-	2
546.4002	Cu II	1	14.96	-	17.23	5p 3F	-	5d 1D	3	-	2
546.6552	Cu II	91	14.60	-	16.87	4d 3G	-	4f 3D	3	-	3
546.6626	Cu II	1	14.43	-	16.70	4d 3D	-	4p 3D	2	-	2

546.9683	Cu II	117	14.62	-	16.88	4d 1G	-	4f 3G	4	-	5
547.2463	Cu II	1	14.70	-	16.96	4d 3F	-	4p 3F	2	-	2
547.2946	Cu II	12	14.60	-	16.86	4d 3G	-	4f 3D	3	-	2
547.3124	Cu II	9	14.99	-	17.25	4p 1D	-	5d 3F	2	-	2
547.3256	Cu II	1	14.64	-	16.90	4p 3D	-	5d 3S	1	-	1
547.6103	Cu II	0	16.17	-	18.43	4p 3P	-	6d 3F	2	-	2
547.7128	Cu II	2	14.53	-	16.80	4d 1P	-	4p 3P	1	-	0
547.8027	Cu II	40	14.62	-	16.88	4d 1G	-	4f 3F	4	-	4
547.9521	Cu II	1	16.17	-	18.43	4p 3P	-	6d 1F	2	-	3
547.9908	Cu II	55	14.99	-	17.25	4p 1D	-	5d 1F	2	-	3
548.0162	Cu II	1	14.60	-	16.86	4d 3G	-	4f 3F	3	-	3
548.2527	Cu II	0	14.30	-	16.56	4p 3G	-	6s 3D	4	-	3
548.6205	Cu II	2	14.99	-	17.25	5p 3P	-	5d 3F	1	-	2
549.2078	Cu II	0	13.68	-	15.94	5s 1D	-	4p 3D	2	-	2
549.3527	Cu II	1	14.96	-	17.22	5p 3F	-	5d 1G	3	-	4
549.5046	Cu II	6	13.68	-	15.94	5s 1D	-	4p 3F	2	-	3
549.6869	Cu II	4	14.34	-	16.59	4d 3P	-	4p 3P	2	-	2
550.0972	Cu II	0	16.17	-	18.42	4p 3P	-	6d 1D	2	-	2
550.4845	Cu II	1	14.34	-	16.59	4d 3P	-	4p 3P	1	-	2
550.7434	Cu II	4	14.62	-	16.87	4d 1G	-	4f 3D	4	-	3
551.2864	Cu II	5	14.96	-	17.21	5p 3F	-	5d 3G	3	-	3
552.1249	Cu II	11	14.62	-	16.86	4d 1G	-	4f 3F	4	-	3
552.1735	Cu II	44	14.99	-	17.23	4p 1D	-	5d 1D	2	-	2
552.5436	Cu II	9	14.98	-	17.22	5p 3F	-	5d 1G	4	-	4
552.7680	Cu II	4	14.62	-	16.86	4d 3D	-	4f 3D	1	-	2
552.8740	Cu II	1	15.98	-	18.22	4p 3D	-	7s 1D	2	-	2
553.4677	Cu II	7	15.01	-	17.25	4p 1F	-	5d 3F	3	-	2
553.5050	Cu II	136	14.99	-	17.23	5p 3P	-	5d 1D	1	-	2
553.7667	Cu II	4	14.75	-	16.99	4p 1G	-	5d 3F	4	-	4
553.8384	Cu II	40	14.62	-	16.85	4d 1G	-	4f 3H	4	-	5
554.0640	Cu II	0	14.75	-	16.99	4p 1G	-	5d 3F	4	-	3
554.1615	Cu II	66	15.01	-	17.25	4p 1F	-	5d 1F	3	-	3
554.3539	Cu II	25	14.76	-	17.00	4p 3F	-	5d 3D	2	-	2
554.4120	Cu II	0	15.98	-	18.21	4p 3D	-	7s 3D	2	-	1
554.4781	Cu II	6	14.62	-	16.86	4d 3D	-	4f 1P	1	-	1
554.4999	Cu II	1	14.98	-	17.21	5p 3F	-	5d 3G	4	-	3
554.7136	Cu II	1	14.99	-	17.22	4p 1D	-	5d 3D	2	-	1
554.9147	Cu II	0	15.94	-	18.17	4p 3F	-	6d 3D	3	-	2
555.2177	Cu II	0	15.94	-	18.17	4p 3D	-	6d 3D	2	-	2
555.4919	Cu II	1	15.94	-	18.17	4p 3F	-	6d 3F	3	-	3
555.5513	Cu II	43	14.76	-	16.99	4p 3F	-	5d 3F	2	-	3
555.7954	Cu II	0	15.94	-	18.17	4p 3D	-	6d 3F	2	-	3

555.8310	Cu II	1	14.60	-	16.83	4d 3G	-	4p 3F	3	-	3
555.9417	Cu II	1	14.62	-	16.85	4d 3D	-	4f 3P	1	-	2
556.0573	Cu II	23	14.99	-	17.22	5p 3P	-	5d 3D	1	-	1
556.2073	Cu II	1	14.62	-	16.85	4d 3D	-	4f 3P	1	-	0
556.2647	Cu II	1	14.43	-	16.66	4d 3D	-	4p 3P	2	-	1
557.1639	Cu II	2	14.99	-	17.21	4p 1D	-	5d 3G	2	-	3
557.3586	Cu II	0	15.94	-	18.16	4p 3F	-	6d 3D	3	-	3
557.3998	Cu II	1	14.62	-	16.85	4d 3D	-	4f 3P	1	-	1
557.6313	Cu II	1	14.75	-	16.98	4p 1G	-	5d 3D	4	-	3
557.6642	Cu II	0	15.94	-	18.16	4p 3D	-	6d 3D	2	-	3
558.3760	Cu II	10	14.39	-	16.61	4d 3D	-	4p 3D	3	-	3
558.3867	Cu II	1	14.53	-	16.75	4d 1P	-	4p 3D	1	-	1
558.4393	Cu II	5	15.01	-	17.23	4p 1F	-	5d 1D	3	-	2
559.1379	Cu II	2	14.76	-	16.98	4p 3F	-	5d 3D	2	-	3
559.3772	Cu II	76	14.65	-	16.87	4d 1D	-	4f 3D	2	-	3
559.6329	Cu II	1	15.94	-	18.15	4p 3F	-	6d 3G	3	-	4
559.8713	Cu II	0	15.94	-	18.15	4p 3F	-	6d 3P	3	-	2
560.0467	Cu II	24	14.65	-	16.86	4d 1D	-	4f 3D	2	-	2
560.0581	Cu II	5	14.62	-	16.83	4d 1G	-	4p 3F	4	-	3
560.1797	Cu II	0	15.94	-	18.15	4p 3D	-	6d 3P	2	-	2
560.8023	Cu II	31	14.65	-	16.86	4d 1D	-	4f 3F	2	-	3
561.0310	Cu II	0	16.01	-	18.22	4p 3D	-	7s 1D	3	-	2
561.5236	Cu II	141	15.01	-	17.22	4p 1F	-	5d 1G	3	-	4
561.8021	Cu II	3	14.65	-	16.86	4d 1D	-	4f 1P	2	-	1
561.9890	Cu II	0	14.65	-	16.86	4d 1D	-	4p 5S	2	-	2
562.0782	Cu II	28	14.69	-	16.90	4d 1F	-	4f 3G	3	-	4
562.1701	Cu II	1	14.45	-	16.66	4d 3P	-	4p 3P	0	-	1
562.2207	Cu II	1	15.97	-	18.17	4p 3F	-	6d 3F	4	-	4
562.4626	Cu II	1	14.75	-	16.96	4p 1G	-	5d 3G	4	-	4
563.3047	Cu II	26	14.65	-	16.85	4d 1D	-	4f 3P	2	-	2
563.3603	Cu II	2	14.39	-	16.59	4d 3D	-	4p 3P	3	-	2
563.5441	Cu II	4	15.01	-	17.21	4p 1F	-	5d 3G	3	-	3
563.7155	Cu II	15	14.75	-	16.95	4p 1G	-	5d 3G	4	-	5
563.7470	Cu II	13	14.76	-	16.96	4p 3F	-	5d 3P	2	-	1
564.1265	Cu II	488	15.23	-	17.43	5p 1P	-	5d 1S	1	-	0
564.1438	Cu II	0	15.98	-	18.17	4p 3D	-	6d 3D	2	-	2
564.2392	Cu II	1	15.97	-	18.16	4p 3F	-	6d 3D	4	-	3
564.2724	Cu II	4	14.99	-	17.18	4p 1D	-	5d 1P	2	-	1
564.3537	Cu II	9	14.76	-	16.96	4p 3F	-	5d 3P	2	-	2
564.7403	Cu II	0	15.98	-	18.17	4p 3D	-	6d 3F	2	-	3
564.8017	Cu II	4	14.65	-	16.85	4d 1D	-	4f 3P	2	-	1
564.8524	Cu II	0	16.24	-	18.43	4p 5D	-	6d 3F	2	-	2

565.1710	Cu II	1	14.64	-	16.83	4p 3D	-	6s 1D	1	-	2
565.2160	Cu II	0	16.24	-	18.43	4p 5D	-	6d 1F	2	-	3
565.6629	Cu II	102	14.99	-	17.18	5p 3P	-	5d 1P	1	-	1
566.1451	Cu II	1	14.42	-	16.61	4d 3F	-	4p 3D	3	-	3
566.4484	Cu II	3	14.69	-	16.88	4d 1F	-	4f 3F	3	-	4
566.5277	Cu II	0	15.94	-	18.13	4p 3D	-	6d 3S	2	-	1
566.5701	Cu II	1	15.97	-	18.15	4p 3F	-	6d 3G	4	-	4
566.6699	Cu II	1	15.98	-	18.16	4p 3D	-	6d 3D	2	-	3
567.2290	Cu II	3	15.97	-	18.15	4p 3F	-	6d 3G	4	-	5
567.4695	Cu II	1	14.65	-	16.83	4p 3F	-	6s 1D	3	-	2
567.4703	Cu II	1	15.07	-	17.25	5p 3D	-	5d 3F	3	-	2
567.6001	Cu II	11	14.43	-	16.61	4d 3F	-	4p 3D	4	-	3
568.1358	Cu II	0	14.43	-	16.61	4d 3D	-	4p 3D	2	-	3
568.1997	Cu II	3	15.07	-	17.25	5p 3D	-	5d 1F	3	-	3
568.2434	Cu II	3	14.64	-	16.82	4p 3D	-	6s 3D	1	-	1
568.8678	Cu II	0	15.98	-	18.15	4p 3D	-	6d 3P	2	-	1
569.2674	Cu II	1	15.98	-	18.15	4p 3D	-	6d 3P	2	-	2
569.4525	Cu II	2	14.62	-	16.80	4d 3D	-	4p 3P	1	-	0
569.5933	Cu II	1	14.69	-	16.87	4d 1F	-	4f 3D	3	-	3
570.0288	Cu II	1	16.24	-	18.41	4p 5D	-	6d 3G	2	-	3
570.2875	Cu II	1	14.69	-	16.86	4d 1F	-	4f 3D	3	-	2
571.0710	Cu II	11	14.69	-	16.86	4d 1F	-	4f 3F	3	-	3
571.1583	Cu II	1	14.70	-	16.87	4d 3F	-	4f 3D	2	-	3
571.2697	Cu II	1	14.42	-	16.59	4d 3F	-	4p 3P	3	-	2
571.8563	Cu II	1	14.70	-	16.86	4d 3F	-	4f 3D	2	-	2
572.3016	Cu II	0	14.69	-	16.86	4d 1F	-	4p 5S	3	-	2
572.6393	Cu II	0	16.01	-	18.17	4p 3D	-	6d 3D	3	-	2
572.6441	Cu II	7	14.70	-	16.86	4d 3F	-	4f 3F	2	-	3
573.1000	Cu II	1	14.53	-	16.70	4d 1P	-	4p 3D	1	-	2
573.1444	Cu II	1	16.01	-	18.17	4p 3D	-	6d 3F	3	-	4
573.2539	Cu II	1	16.01	-	18.17	4p 3D	-	6d 3F	3	-	3
573.2967	Cu II	1	14.43	-	16.59	4d 3D	-	4p 3P	2	-	2
573.6661	Cu II	3	14.69	-	16.85	4d 1F	-	4f 3P	3	-	2
573.6867	Cu II	0	14.70	-	16.86	4d 3F	-	4f 1P	2	-	1
573.8816	Cu II	0	14.70	-	16.86	4d 3F	-	4p 5S	2	-	2
575.2422	Cu II	0	16.01	-	18.16	4p 3D	-	6d 3D	3	-	3
575.2536	Cu II	5	14.70	-	16.85	4d 3F	-	4f 3P	2	-	2
575.8242	Cu II	1	15.98	-	18.13	4p 3D	-	6d 3S	2	-	1
575.9421	Cu II	80	15.07	-	17.22	5p 3D	-	5d 1G	3	-	4
576.1218	Cu II	2	14.43	-	16.58	4p 3D	-	6s 3D	3	-	2
576.6048	Cu II	0	16.09	-	18.24	4p 3P	-	6d 3P	1	-	0

576.8148	Cu II	2	14.70	-	16.85	4d 3F	-	4f 3P	2	-	1
577.2513	Cu II	0	16.28	-	18.43	4p 5D	-	6d 1F	3	-	3
577.6651	Cu II	1	16.01	-	18.15	4p 3D	-	6d 3G	3	-	4
577.9662	Cu II	5	14.76	-	16.90	4p 3F	-	5d 3S	2	-	1
578.0679	Cu II	2	15.07	-	17.21	5p 3D	-	5d 3G	3	-	3
578.3920	Cu II	134	15.29	-	17.43	5p 3D	-	5d 1S	1	-	0
579.5623	Cu II	1	14.69	-	16.83	4d 1F	-	4p 3F	3	-	3
580.0970	Cu II	0	13.43	-	15.57	5s 3D	-	4p 5P	2	-	1
580.1120	Cu II	1	13.39	-	15.53	5s 3D	-	4p 5P	3	-	2
580.5989	Cu II	6	14.43	-	16.56	4p 3D	-	6s 3D	3	-	3
580.7191	Cu II	2	13.39	-	15.53	5s 3D	-	4p 5P	3	-	3
580.9995	Cu II	0	14.62	-	16.75	4d 3D	-	4p 3D	1	-	1
581.1287	Cu II	1	16.28	-	18.41	4p 5D	-	6d 1G	3	-	4
581.1826	Cu II	0	14.70	-	16.83	4d 3F	-	4p 3F	2	-	3
582.2721	Cu II	0	16.28	-	18.41	4p 5D	-	6d 3G	3	-	3
582.5826	Cu II	271	15.12	-	17.25	5p 3D	-	5d 3F	2	-	2
583.3515	Cu II	152	15.12	-	17.25	5p 3D	-	5d 1F	2	-	3
583.6622	Cu II	0	14.53	-	16.66	4d 1P	-	4p 3P	1	-	1
583.7315	Cu II	0	16.09	-	18.21	4p 3P	-	7s 3D	1	-	1
585.1778	Cu II	0	14.46	-	16.58	4p 3G	-	6s 3D	3	-	2
588.0936	Cu II	12	15.12	-	17.23	5p 3D	-	5d 1D	2	-	2
588.7212	Cu II	4	14.89	-	17.00	5p 3P	-	5d 3D	2	-	2
589.0460	Cu II	2	14.65	-	16.75	4d 1D	-	4p 3D	2	-	1
589.5798	Cu II	0	16.59	-	18.69	4p 3P	-	8s 3D	2	-	2
589.7971	Cu II	4	14.46	-	16.56	4p 3G	-	6s 3D	3	-	3
590.0718	Cu II	0	14.89	-	16.99	5p 3P	-	5d 3F	2	-	3
590.9584	Cu II	3	16.59	-	18.69	4p 3P	-	8s 3D	2	-	3
590.9757	Cu II	52	15.12	-	17.22	5p 3D	-	5d 3D	2	-	1
591.1146	Cu II	0	13.43	-	15.53	5s 3D	-	4p 5P	2	-	2
591.7450	Cu II	0	13.43	-	15.53	5s 3D	-	4p 5P	2	-	3
592.9639	Cu II	0	14.62	-	16.71	4d 1G	-	4p 3H	4	-	4
593.7577	Cu II	418	15.12	-	17.21	5p 3D	-	5d 3G	2	-	3
594.1196	Cu II	1295	14.89	-	16.98	5p 3P	-	5d 3D	2	-	3
594.5299	Cu II	0	16.09	-	18.17	4p 3P	-	6d 3D	1	-	2
595.1395	Cu II	1	16.61	-	18.69	4p 3D	-	8s 3D	3	-	2
596.5442	Cu II	9	16.61	-	18.69	4p 3D	-	8s 3D	3	-	3
596.9456	Cu II	1	14.62	-	16.70	4d 3D	-	4p 3D	1	-	2
597.7852	Cu II	0	16.95	-	19.03	5d 3G	-	6f 1H	5	-	5
597.9019	Cu II	2	14.76	-	16.83	4p 3F	-	6s 1D	2	-	2
598.8309	Cu II	564	14.99	-	17.06	5p 3P	-	5d 3P	1	-	0
599.0015	Cu II	0	16.96	-	19.03	5d 3G	-	6f 3H	4	-	4
599.2007	Cu II	3	16.96	-	19.03	5d 3G	-	6f 1H	4	-	5

599.3260	Cu II	211	14.89	-	16.96	5p 3P	-	5d 3P	2	-	1
599.5587	Cu II	548	15.15	-	17.22	5p 3P	-	5d 3D	0	-	1
599.7789	Cu II	0	16.09	-	18.15	4p 3P	-	6d 3P	1	-	1
600.0120	Cu II	1991	14.89	-	16.96	5p 3P	-	5d 3P	2	-	2
600.2305	Cu II	25	16.56	-	18.63	6s 3D	-	4p 3F	3	-	2
601.3416	Cu II	7	14.76	-	16.82	4p 3F	-	6s 3D	2	-	1
601.8373	Cu II	21	15.12	-	17.18	5p 3D	-	5d 1P	2	-	1
602.1245	Cu II	1	14.70	-	16.75	4d 3F	-	4p 3D	2	-	1
602.3264	Cu II	6	14.52	-	16.58	4p 3D	-	6s 3D	2	-	2
602.4416	Cu II	1	14.53	-	16.59	4d 1P	-	4p 3P	1	-	2
604.0650	Cu II	1	16.17	-	18.22	4p 3P	-	7s 1D	2	-	2
604.5799	Cu II	0	16.98	-	19.03	5d 3D	-	6f 3H	3	-	4
605.0918	Cu II	26	16.58	-	18.63	6s 3D	-	4p 3F	2	-	2
605.4431	Cu II	1	14.65	-	16.70	4d 1D	-	4p 3D	2	-	2
607.2218	Cu II	3	14.52	-	16.56	4p 3D	-	6s 3D	2	-	3
607.5169	Cu II	0	16.09	-	18.13	4p 3P	-	6d 3S	1	-	1
608.0343	Cu II	0	-		-		-				
608.4139	Cu II	1	14.62	-	16.66	4d 3D	-	4p 3P	1	-	1
608.7481	Cu II	1	16.66	-	18.69	4p 3P	-	8s 3D	1	-	2
608.8298	Cu II	5	16.99	-	19.03	5d 3F	-	6f 3H	3	-	4
609.1893	Cu II	0	16.99	-	19.03	5d 3F	-	6f 3H	4	-	4
609.3952	Cu II	3	16.99	-	19.03	5d 3F	-	6f 1H	4	-	5
609.7325	Cu II	515	15.22	-	17.25	5p 3F	-	5d 3F	2	-	2
609.9990	Cu II	195	14.96	-	17.00	5p 3F	-	5d 3D	3	-	2
610.5746	Cu II	196	15.22	-	17.25	5p 3F	-	5d 1F	2	-	3
610.7412	Cu II	455	15.15	-	17.18	5p 3P	-	5d 1P	0	-	1
611.0872	Cu II	99	14.96	-	16.99	5p 3F	-	5d 3F	3	-	4
611.4493	Cu II	1316	14.96	-	16.99	5p 3F	-	5d 3F	3	-	3
612.4378	Cu II	1	15.94	-	17.96	4p 3F	-	7s 3D	3	-	2
612.8068	Cu II	0	15.94	-	17.96	4p 3D	-	7s 3D	2	-	2
614.2956	Cu II	25	15.23	-	17.25	5p 1P	-	5d 3F	1	-	2
615.0025	Cu II	1	15.94	-	17.95	4p 3F	-	7s 3D	3	-	3
615.0384	Cu II	1695	14.98	-	16.99	5p 3F	-	5d 3F	4	-	4
615.3746	Cu II	0	15.94	-	17.95	4p 3D	-	7s 3D	2	-	3
615.4051	Cu II	34	14.98	-	16.99	5p 3F	-	5d 3F	4	-	3
615.4222	Cu II	1480	14.89	-	16.90	5p 3P	-	5d 3S	2	-	1
615.7076	Cu II	1	14.60	-	16.61	4d 3G	-	4p 3D	3	-	3
615.7717	Cu II	177	15.22	-	17.23	5p 3F	-	5d 1D	2	-	2
615.7968	Cu II	15	14.96	-	16.98	5p 3F	-	5d 3D	3	-	3
617.2037	Cu II	929	14.99	-	17.00	4p 1D	-	5d 3D	2	-	2
617.4291	Cu II	3	14.69	-	16.70	4d 1F	-	4p 3D	3	-	2
617.5439	Cu II	0	16.17	-	18.17	4p 3P	-	6d 3D	2	-	2

618.2587	Cu II	0	16.17	-	18.17	4p 3P	-	6d 3F	2	-	3
618.6884	Cu II	1446	14.99	-	16.99	4p 1D	-	5d 3F	2	-	3
618.7420	Cu II	0	16.23	-	18.24	4p 5D	-	6d 3P	1	-	0
618.8676	Cu II	929	14.99	-	17.00	5p 3P	-	5d 3D	1	-	2
618.9322	Cu II	56	15.22	-	17.22	5p 3F	-	5d 3D	2	-	1
619.2684	Cu II	1	14.70	-	16.70	4d 3F	-	4p 3D	2	-	2
619.8092	Cu II	333	14.98	-	16.98	5p 3F	-	5d 3D	4	-	3
619.9751	Cu II	111	15.25	-	17.25	5p 1F	-	5d 3F	3	-	2
620.4261	Cu II	1217	15.23	-	17.23	5p 1P	-	5d 1D	1	-	2
620.5720	Cu II	1	16.17	-	18.16	4p 3P	-	6d 3D	2	-	3
620.6789	Cu II	1	16.70	-	18.69	4p 3D	-	8s 3D	2	-	2
620.8457	Cu II	1105	15.25	-	17.25	5p 1F	-	5d 1F	3	-	3
620.8987	Cu II	3	14.62	-	16.61	4d 1G	-	4p 3D	4	-	3
621.6939	Cu II	4426	14.96	-	16.96	5p 3F	-	5d 3G	3	-	4
621.7735	Cu II	0	14.60	-	16.59	4d 3G	-	4p 3P	3	-	2
621.9844	Cu II	3492	15.22	-	17.21	5p 3F	-	5d 3G	2	-	3
622.1291	Cu II	47	14.96	-	16.96	5p 3F	-	5d 3P	3	-	2
622.2069	Cu II	1	16.70	-	18.69	4p 3D	-	8s 3D	2	-	3
623.1395	Cu II	25	14.99	-	16.98	4p 1D	-	5d 3D	2	-	3
623.2090	Cu II	0	16.17	-	18.15	4p 3P	-	6d 3P	2	-	1
623.3905	Cu II	2	15.97	-	17.95	4p 3F	-	7s 3D	4	-	3
623.6347	Cu II	90	15.23	-	17.22	5p 1P	-	5d 3D	1	-	1
623.6886	Cu II	1	16.17	-	18.15	4p 3P	-	6d 3P	2	-	2
623.6988	Cu II	0	15.98	-	17.96	4p 3D	-	7s 3D	2	-	2
624.8255	Cu II	3	16.71	-	18.69	4p 3H	-	8s 3D	4	-	3
624.9897	Cu II	0	16.23	-	18.22	4p 5D	-	7s 1D	1	-	2
625.0424	Cu II	17	15.01	-	17.00	4p 1F	-	5d 3D	3	-	2
625.7837	Cu II	292	14.98	-	16.96	5p 3F	-	5d 3G	4	-	4
626.1848	Cu II	1143	15.01	-	16.99	4p 1F	-	5d 3F	3	-	4
626.2200	Cu II	78	15.25	-	17.23	5p 1F	-	5d 1D	3	-	2
626.3589	Cu II	1	15.98	-	17.95	4p 3D	-	7s 3D	2	-	3
626.5651	Cu II	139	15.01	-	16.99	4p 1F	-	5d 3F	3	-	3
627.0806	Cu II	0	16.24	-	18.21	4p 5D	-	7s 3D	2	-	1
627.3349	Cu II	6383	14.98	-	16.95	5p 3F	-	5d 3G	4	-	5
627.6673	Cu II	29	14.59	-	16.56	4p 3F	-	6s 3D	4	-	3
628.8696	Cu II	290	14.99	-	16.96	4p 1D	-	5d 3P	2	-	1
629.6245	Cu II	14	14.99	-	16.96	4p 1D	-	5d 3P	2	-	2
629.7407	Cu II	0	14.20	-	16.17	4d 3S	-	4p 3P	1	-	2
630.1009	Cu II	5035	15.25	-	17.22	5p 1F	-	5d 1G	3	-	4
630.5972	Cu II	906	14.99	-	16.96	5p 3P	-	5d 3P	1	-	1
630.8561	Cu II	2	15.22	-	17.18	5p 3F	-	5d 1P	2	-	1

631.1309	Cu II	929	15.01	-	16.98	4p 1F	-	5d 3D	3	-	3
631.2492	Cu II	1443	15.29	-	17.25	5p 3D	-	5d 3F	1	-	2
631.3563	Cu II	27	14.99	-	16.96	5p 3P	-	5d 3P	1	-	2
631.5676	Cu II	1	16.17	-	18.13	4p 3P	-	6d 3S	2	-	1
631.6193	Cu II	1	14.70	-	16.66	4d 3F	-	4p 3P	2	-	1
632.6466	Cu II	212	15.25	-	17.21	5p 1F	-	5d 3G	3	-	3
634.0992	Cu II	1	16.01	-	17.96	4p 3D	-	7s 3D	3	-	2
635.7422	Cu II	927	15.23	-	17.18	5p 1P	-	5d 1P	1	-	1
637.3268	Cu II	317	15.01	-	16.96	4p 1F	-	5d 3G	3	-	4
637.5726	Cu II	0	16.27	-	18.21	4p 5D	-	7s 3D	0	-	1
637.7246	Cu II	367	15.29	-	17.23	5p 3D	-	5d 1D	1	-	2
637.7840	Cu II	388	15.01	-	16.96	4p 1F	-	5d 3P	3	-	2
638.0762	Cu II	15	14.89	-	16.83	5p 3P	-	6s 1D	2	-	2
638.2847	Cu II	0	14.65	-	16.59	4d 1D	-	4p 3P	2	-	2
638.5264	Cu II	2	14.64	-	16.58	4p 3D	-	6s 3D	1	-	2
638.8065	Cu II	1	14.34	-	16.28	4d 3G	-	4p 5D	4	-	3
638.9116	Cu II	1	16.75	-	18.69	4p 3D	-	8s 3D	1	-	2
639.4297	Cu II	0	16.23	-	18.17	4p 5D	-	6d 3D	1	-	2
639.8682	Cu II	1	16.28	-	18.22	4p 5D	-	7s 1D	3	-	2
640.3384	Cu II	479	15.18	-	17.11	4d 1S	-	4f 3D	0	-	1
641.1151	Cu II	858	15.29	-	17.22	5p 3D	-	5d 3D	1	-	1
641.4563	Cu II	533	15.32	-	17.25	5p 1D	-	5d 3F	2	-	2
641.4618	Cu II	23	14.65	-	16.58	4p 3F	-	6s 3D	3	-	2
641.9952	Cu II	2	14.89	-	16.82	5p 3P	-	6s 3D	2	-	1
642.3884	Cu II	2450	15.32	-	17.25	5p 1D	-	5d 1F	2	-	3
642.8079	Cu II	0	16.24	-	18.16	4p 5D	-	6d 3D	2	-	3
642.9595	Cu II	7	15.07	-	17.00	5p 3D	-	5d 3D	3	-	2
643.2409	Cu II	26	13.39	-	15.32	5s 3D	-	5p 1D	3	-	2
644.1684	Cu II	2224	15.07	-	16.99	5p 3D	-	5d 3F	3	-	4
644.2965	Cu II	0		-			-			-	
644.5708	Cu II	23	15.07	-	16.99	5p 3D	-	5d 3F	3	-	3
644.8559	Cu II	0		-			-			-	
644.9616	Cu II	0	14.69	-	16.61	4d 1F	-	4p 3D	3	-	3
645.5054	Cu II	0	16.23	-	18.15	4p 5D	-	6d 3P	1	-	1
645.6141	Cu II	1	13.65	-	15.57	5s 3D	-	4p 5P	1	-	1
646.1525	Cu II	0	16.24	-	18.15	4p 5D	-	6d 3P	2	-	2
646.6246	Cu II	1	14.99	-	16.90	4p 1D	-	5d 3S	2	-	1
647.0168	Cu II	18	14.65	-	16.56	4p 3F	-	6s 3D	3	-	3
648.1437	Cu II	867	15.32	-	17.23	5p 1D	-	5d 1D	2	-	2
648.4421	Cu II	259	14.99	-	16.90	5p 3P	-	5d 3S	1	-	1
649.4038	Cu II	1378	15.07	-	16.98	5p 3D	-	5d 3D	3	-	3
651.6207	Cu II	0	14.69	-	16.59	4d 1F	-	4p 3P	3	-	2

651.6463	Cu II	119	15.32	-	17.22	5p 1D	-	5d 3D	2	-	1
651.7317	Cu II	0	-		-		-				
653.0083	Cu II	0	-		-		-				
653.6697	Cu II	0	14.70	-	16.59	4d 3F	-	4p 3P	2	-	2
653.9179	Cu II	2	15.29	-	17.18	5p 3D	-	5d 1P	1	-	1
654.6131	Cu II	0	16.24	-	18.13	4p 5D	-	6d 3S	2	-	1
655.0122	Cu II	0	16.28	-	18.17	4p 5D	-	6d 3D	3	-	2
655.0304	Cu II	4	15.32	-	17.21	5p 1D	-	5d 3G	2	-	3
655.1286	Cu II	0	-		-		-				
655.6207	Cu II	0	14.20	-	16.09	4d 3S	-	4p 3P	1	-	1
655.6731	Cu II	0	16.28	-	18.17	4p 5D	-	6d 3F	3	-	4
655.8164	Cu II	1	16.28	-	18.17	4p 5D	-	6d 3F	3	-	3
655.9655	Cu II	189	15.07	-	16.96	5p 3D	-	5d 3G	3	-	4
656.4501	Cu II	463	15.07	-	16.96	5p 3D	-	5d 3P	3	-	2
656.7963	Cu II	1	13.43	-	15.32	5s 3D	-	5p 1D	2	-	2
657.5761	Cu II	0	13.68	-	15.57	5s 1D	-	4p 5P	2	-	1
657.7080	Cu II	0	-		-		-				
658.4200	Cu II	0	16.28	-	18.16	4p 5D	-	6d 3D	3	-	3
659.2901	Cu II	2	13.65	-	15.53	5s 3D	-	4p 5P	1	-	2
661.0514	Cu II	1	16.09	-	17.96	4p 3P	-	7s 3D	1	-	2
661.5961	Cu II	1	16.28	-	18.15	4p 5D	-	6d 3G	3	-	4
661.9294	Cu II	0	16.28	-	18.15	4p 5D	-	6d 3P	3	-	2
662.4292	Cu II	611	15.12	-	17.00	5p 3D	-	5d 3D	2	-	2
663.1474	Cu II	1	14.96	-	16.83	5p 3F	-	6s 1D	3	-	2
663.5167	Cu II	1	16.90	-	18.77	5d 3S	-	6f 3D	1	-	2
664.1396	Cu II	964	15.12	-	16.99	5p 3D	-	5d 3F	2	-	3
664.3113	Cu II	3	16.83	-	18.69	4p 3F	-	8s 3D	3	-	2
664.7799	Cu II	199	16.90	-	18.77	5d 3S	-	6f 3P	1	-	2
664.8774	Cu II	228	15.32	-	17.18	5p 1D	-	5d 1P	2	-	1
665.5945	Cu II	0	15.57	-	17.43	4p 5P	-	5d 1S	1	-	0
666.0620	Cu II	22	16.83	-	18.69	4p 3F	-	8s 3D	3	-	3
666.0962	Cu II	0	-		-		-				
666.3947	Cu II	19	13.39	-	15.25	5s 3D	-	5p 1F	3	-	3
667.0825	Cu II	0	14.42	-	16.28	4d 3F	-	4p 5D	3	-	3
667.8534	Cu II	9	13.43	-	15.29	5s 3D	-	5p 3D	2	-	1
669.1035	Cu II	0	14.43	-	16.28	4d 3F	-	4p 5D	4	-	3
669.2714	Cu II	43	15.12	-	16.98	5p 3D	-	5d 3D	2	-	3
669.8481	Cu II	0	14.43	-	16.28	4d 3D	-	4p 5D	2	-	3
671.6706	Cu II	29	14.99	-	16.83	4p 1D	-	6s 1D	2	-	2
671.7692	Cu II	8	13.68	-	15.53	5s 1D	-	4p 5P	2	-	2
672.5835	Cu II	3	13.68	-	15.53	5s 1D	-	4p 5P	2	-	3
673.5433	Cu II	1	16.66	-	18.50	4p 3P	-	6d 1S	1	-	0

673.6417	Cu II	60	14.99	-	16.83	5p 3P	-	6s 1D	1	-	2
674.1142	Cu II	0	16.86	-	18.69	4p 5S	-	8s 3D	2	-	2
674.7342	Cu II	0	16.59	-	18.43	4p 3P	-	6d 3F	2	-	2
675.2532	Cu II	1	16.59	-	18.43	4p 3P	-	6d 1F	2	-	3
675.6347	Cu II	1	15.18	-	17.01	4d 1S	-	4p 1P	0	-	1
675.8857	Cu II	151	15.12	-	16.96	5p 3D	-	5d 3P	2	-	1
675.9170	Cu II	1	16.86	-	18.69	4p 5S	-	8s 3D	2	-	3
676.0145	Cu II	6	14.99	-	16.82	4p 1D	-	6s 3D	2	-	1
676.7579	Cu II	0	15.12	-	16.96	5p 3D	-	5d 3P	2	-	2
677.0362	Cu II	0		-		-		-			
677.8896	Cu II	1	14.34	-	16.17	4d 3P	-	4p 3P	2	-	2
677.9440	Cu II	4	15.23	-	17.06	5p 1P	-	5d 3P	1	-	0
678.0113	Cu II	35	14.99	-	16.82	5p 3P	-	6s 3D	1	-	1
678.5136	Cu II	1	16.59	-	18.42	4p 3P	-	6d 1D	2	-	2
678.6485	Cu II	6	13.39	-	15.22	5s 3D	-	5p 3F	3	-	2
679.1030	Cu II	0	14.34	-	16.17	4d 3P	-	4p 3P	1	-	2
680.6216	Cu II	15	14.76	-	16.58	4p 3F	-	6s 3D	2	-	2
680.9546	Cu II	13	13.43	-	15.25	5s 3D	-	5p 1F	2	-	3
680.9647	Cu II	70	15.01	-	16.83	4p 1F	-	6s 1D	3	-	2
681.7116	Cu II	50	16.96	-	18.77	5d 3P	-	6f 3F	2	-	3
682.0258	Cu II	0	16.61	-	18.43	4p 3D	-	6d 3F	3	-	2
682.1336	Cu II	0	16.59	-	18.41	4p 3P	-	6d 3G	2	-	3
682.2350	Cu II	20	16.96	-	18.77	5d 3G	-	6f 3F	4	-	3
682.3202	Cu II	1679	16.95	-	18.77	5d 3G	-	6f 3H	5	-	6
682.4133	Cu II	106	16.96	-	18.77	5d 3P	-	6f 3D	2	-	2
682.4750	Cu II	344	16.96	-	18.77	5d 3P	-	6f 3F	2	-	3
682.5560	Cu II	1	16.61	-	18.43	4p 3D	-	6d 1F	3	-	3
682.5697	Cu II	26	16.95	-	18.77	5d 3G	-	6f 3H	5	-	5
682.9996	Cu II	1	16.96	-	18.77	5d 3G	-	6f 3F	4	-	3
683.0532	Cu II	1125	17.21	-	19.03	5d 3G	-	6f 3H	3	-	4
683.3024	Cu II	278	16.96	-	18.77	5d 3P	-	6f 3D	1	-	2
683.7494	Cu II	277	16.96	-	18.77	5d 3P	-	6f 3P	2	-	2
684.4157	Cu II	1368	16.96	-	18.77	5d 3G	-	6f 3H	4	-	5
684.6067	Cu II	6	14.75	-	16.56	4p 1G	-	6s 3D	4	-	3
684.6421	Cu II	42	16.96	-	18.77	5d 3P	-	6f 3P	1	-	2
685.4297	Cu II	2	16.82	-	18.63	6s 3D	-	4p 3F	1	-	2
685.8876	Cu II	0	16.61	-	18.42	4p 3D	-	6d 1D	3	-	2
686.0453	Cu II	33	17.22	-	19.03	5d 1G	-	6f 3H	4	-	4
686.3065	Cu II	1406	17.22	-	19.03	5d 1G	-	6f 1H	4	-	5
686.8791	Cu II	10	14.76	-	16.56	4p 3F	-	6s 3D	2	-	3
687.1357	Cu II	1	15.15	-	16.96	5p 3P	-	5d 3P	0	-	1

687.2231	Cu II	0	-		-			-				
687.7756	Cu II	1	16.59	-	18.39	4p 3P	-	6d 1P	2	-	1
687.9404	Cu II	93	13.43	-	15.23	5s 3D	-	5p 1P	2	-	1
687.9837	Cu II	9	16.61	-	18.41	4p 3D	-	6d 1G	3	-	4
689.4806	Cu II	44	16.98	-	18.77	5d 3D	-	6f 3F	3	-	3
689.5868	Cu II	1	16.61	-	18.41	4p 3D	-	6d 3G	3	-	3
689.6271	Cu II	0	16.17	-	17.96	4p 3P	-	7s 3D	2	-	2
689.9540	Cu II	7	16.83	-	18.63	6s 1D	-	4p 3F	2	-	2
690.1984	Cu II	24	16.98	-	18.77	5d 3D	-	6f 3D	3	-	2
690.2616	Cu II	387	16.98	-	18.77	5d 3D	-	6f 3F	3	-	3
691.5653	Cu II	71	16.98	-	18.77	5d 3D	-	6f 3P	3	-	2
692.8808	Cu II	1	16.17	-	17.95	4p 3P	-	7s 3D	2	-	3
693.7553	Cu II	108	13.43	-	15.22	5s 3D	-	5p 3F	2	-	2
695.0135	Cu II	273	16.99	-	18.77	5d 3F	-	6f 3F	3	-	3
695.2871	Cu II	0	-		-			-				
695.4819	Cu II	8	16.99	-	18.77	5d 3F	-	6f 3F	4	-	3
695.7429	Cu II	32	16.99	-	18.77	5d 3F	-	6f 3D	3	-	2
695.8070	Cu II	9	16.99	-	18.77	5d 3F	-	6f 3F	3	-	3
695.9973	Cu II	0	14.45	-	16.23	4d 3P	-	4p 5D	0	-	1
696.2765	Cu II	48	16.99	-	18.77	5d 3F	-	6f 3F	4	-	3
696.4274	Cu II	6	15.12	-	16.90	5p 3D	-	5d 3S	2	-	1
696.8966	Cu II	491	17.00	-	18.77	5d 3D	-	6f 3F	2	-	3
697.0214	Cu II	1	14.20	-	15.98	4d 3S	-	4p 3D	1	-	2
697.1318	Cu II	1	16.99	-	18.77	5d 3F	-	6f 3P	3	-	2
697.3642	Cu II	1	17.25	-	19.03	5d 1F	-	6f 3H	3	-	4
697.6299	Cu II	199	17.00	-	18.77	5d 3D	-	6f 3D	2	-	2
697.6944	Cu II	107	17.00	-	18.77	5d 3D	-	6f 3F	2	-	3
697.7572	Cu II	21	16.99	-	18.77	5d 3F	-	6f 3H	4	-	5
697.7563	Cu II	108	15.22	-	17.00	5p 3F	-	5d 3D	2	-	2
698.6521	Cu II	35	15.29	-	17.06	5p 3D	-	5d 3P	1	-	0
698.8061	Cu II	0	14.39	-	16.17	4d 3D	-	4p 3P	3	-	2
699.0264	Cu II	56	17.00	-	18.77	5d 3D	-	6f 3P	2	-	2
699.0547	Cu II	5	16.92	-	18.69	4p 3D	-	8s 3D	3	-	3
699.6543	Cu II	68	15.22	-	16.99	5p 3F	-	5d 3F	2	-	3
699.9578	Cu II	3	16.66	-	18.43	4p 3P	-	6d 3F	1	-	2
702.2860	Cu II	9	15.07	-	16.83	5p 3D	-	6s 1D	3	-	2
703.7385	Cu II	70	15.23	-	17.00	5p 1P	-	5d 3D	1	-	2
704.0259	Cu II	0	16.66	-	18.42	4p 3P	-	6d 1D	1	-	2
705.3523	Cu II	10	15.22	-	16.98	5p 3F	-	5d 3D	2	-	3
705.9342	Cu II	3	16.66	-	18.41	4p 3P	-	6d 3D	1	-	1
708.3776	Cu II	7	15.15	-	16.90	5p 3P	-	5d 3S	0	-	1
709.2964	Cu II	0	14.34	-	16.09	4d 3P	-	4p 3P	1	-	1

711.0170	Cu II	0	14.42	-	16.17	4d 3F	-	4p 3P	3	-	2
711.1472	Cu II	0	14.20	-	15.94	4d 3S	-	4p 3D	1	-	2
711.2023	Cu II	1	15.25	-	17.00	5p 1F	-	5d 3D	3	-	2
712.6817	Cu II	7	15.25	-	16.99	5p 1F	-	5d 3F	3	-	4
712.7030	Cu II	28	15.22	-	16.96	5p 3F	-	5d 3P	2	-	1
713.1743	Cu II	9	15.25	-	16.99	5p 1F	-	5d 3F	3	-	3
713.6728	Cu II	1	15.22	-	16.96	5p 3F	-	5d 3P	2	-	2
714.0026	Cu II	1	16.66	-	18.39	4p 3P	-	6d 1P	1	-	1
714.1597	Cu II	0	14.43	-	16.17	4d 3D	-	4p 3P	2	-	2
714.9917	Cu II	20	16.96	-	18.69	4p 3F	-	8s 3D	2	-	2
715.7757	Cu II	25	13.39	-	15.12	5s 3D	-	5p 3D	3	-	2
715.7780	Cu II	4	16.70	-	18.43	4p 3D	-	6d 3F	2	-	2
716.3620	Cu II	19	16.70	-	18.43	4p 3D	-	6d 1F	2	-	3
717.0202	Cu II	8	16.96	-	18.69	4p 3F	-	8s 3D	2	-	3
717.0337	Cu II	0	16.23	-	17.96	4p 5D	-	7s 3D	1	-	2
718.5547	Cu II	15	16.90	-	18.63	5d 3S	-	4p 3F	1	-	2
718.6088	Cu II	0	15.53	-	17.25	4p 5P	-	5d 3F	3	-	2
718.9454	Cu II	123	15.23	-	16.96	5p 1P	-	5d 3P	1	-	1
719.0955	Cu II	11	15.25	-	16.98	5p 1F	-	5d 3D	3	-	3
719.4896	Cu II	0	-		-		-				
719.5407	Cu II	3	15.53	-	17.25	4p 5P	-	5d 3F	2	-	2
719.7789	Cu II	1	15.53	-	17.25	4p 5P	-	5d 1F	3	-	3
719.9323	Cu II	1	15.23	-	16.96	5p 1P	-	5d 3P	1	-	2
720.0327	Cu II	7	16.70	-	18.42	4p 3D	-	6d 1D	2	-	2
720.7138	Cu II	12	15.53	-	17.25	4p 5P	-	5d 1F	2	-	3
720.7166	Cu II	0	16.24	-	17.95	4p 5D	-	7s 3D	2	-	3
722.0288	Cu II	1	16.70	-	18.41	4p 3D	-	6d 3D	2	-	1
724.1105	Cu II	0	16.70	-	18.41	4p 3D	-	6d 3G	2	-	3
725.5786	Cu II	167	15.12	-	16.83	5p 3D	-	6s 1D	2	-	2
725.8747	Cu II	0	16.71	-	18.41	4p 3H	-	6d 1G	4	-	4
726.0784	Cu II	1	15.29	-	17.00	5p 3D	-	5d 3D	1	-	2
727.0123	Cu II	0	15.53	-	17.23	4p 5P	-	5d 1D	3	-	2
727.1499	Cu II	19	15.25	-	16.96	5p 1F	-	5d 3G	3	-	4
727.7455	Cu II	5	15.25	-	16.96	5p 1F	-	5d 3P	3	-	2
727.9661	Cu II	5	15.53	-	17.23	4p 5P	-	5d 1D	2	-	2
730.4715	Cu II	2	16.70	-	18.39	4p 3D	-	6d 1P	2	-	1
730.6504	Cu II	84	15.12	-	16.82	5p 3D	-	6s 3D	2	-	1
731.6104	Cu II	2	17.00	-	18.69	4p 3G	-	8s 3D	3	-	3
732.2484	Cu II	5	15.53	-	17.22	4p 5P	-	5d 1G	3	-	4
732.3873	Cu II	1	15.53	-	17.22	4p 5P	-	5d 3D	2	-	1
732.6008	Cu II	917	13.43	-	15.12	5s 3D	-	5p 3D	2	-	2
733.1694	Cu II	64	14.89	-	16.58	5p 3P	-	6s 3D	2	-	2

735.5813	Cu II	1	15.22	-	16.90	5p 3F	-	5d 3S	2	-	1
735.6881	Cu II	1	15.53	-	17.21	4p 5P	-	5d 3G	3	-	3
736.5693	Cu II	1	15.57	-	17.25	4p 5P	-	5d 3F	1	-	2
736.6648	Cu II	0	15.53	-	17.21	4p 5P	-	5d 3G	2	-	3
736.6862	Cu II	1	16.28	-	17.96	4p 5D	-	7s 3D	3	-	2
736.7338	Cu II	3	17.01	-	18.69	4p 1P	-	8s 3D	1	-	2
738.2277	Cu II	250	15.18	-	16.86	4d 1S	-	4f 1P	0	-	1
739.6151	Cu II	10	15.32	-	17.00	5p 1D	-	5d 3D	2	-	2
739.9875	Cu II	1589	13.39	-	15.07	5s 3D	-	5p 3D	3	-	3
740.1354	Cu II	4	16.75	-	18.43	4p 3D	-	6d 3F	1	-	2
740.4002	Cu II	1	16.28	-	17.95	4p 5D	-	7s 3D	3	-	3
740.4354	Cu II	1325	14.89	-	16.56	5p 3P	-	6s 3D	2	-	3
740.7686	Cu II	16	16.96	-	18.63	5d 3P	-	4p 3F	2	-	2
741.7379	Cu II	0	14.34	-	16.01	4d 3P	-	4p 3D	2	-	3
741.7481	Cu II	40	15.32	-	16.99	5p 1D	-	5d 3F	2	-	3
741.8164	Cu II	0	16.96	-	18.63	5d 3P	-	4p 3F	1	-	2
742.0567	Cu II	209	13.65	-	15.32	5s 3D	-	5p 1D	1	-	2
742.2328	Cu II	4	15.23	-	16.90	5p 1P	-	5d 3S	1	-	1
742.2771	Cu II	1	15.29	-	16.96	5p 3D	-	5d 3P	1	-	1
743.0475	Cu II	1	14.34	-	16.01	4d 3G	-	4p 3D	4	-	3
743.3292	Cu II	1	15.29	-	16.96	5p 3D	-	5d 3P	1	-	2
743.4156	Cu II	63	15.18	-	16.85	4d 1S	-	4f 3P	0	-	1
743.8151	Cu II	283	15.15	-	16.82	5p 3P	-	6s 3D	0	-	1
744.4339	Cu II	1	14.62	-	16.28	4d 1G	-	4p 5D	4	-	3
744.6855	Cu II	14	16.75	-	18.42	4p 3D	-	6d 1D	1	-	2
745.4006	Cu II	1	15.57	-	17.23	4p 5P	-	5d 1D	1	-	2
746.8209	Cu II	1	16.75	-	18.41	4p 3D	-	6d 3D	1	-	1
747.6276	Cu II	0	14.43	-	16.09	4d 3D	-	4p 3P	2	-	1
748.1555	Cu II	11	15.32	-	16.98	5p 1D	-	5d 3D	2	-	3
749.1425	Cu II	2	15.53	-	17.18	4p 5P	-	5d 1P	2	-	1
749.4792	Cu II	0	16.85	-	18.50	4f 3P	-	6d 1S	1	-	0
749.9511	Cu II	15	16.98	-	18.63	5d 3D	-	4p 3F	3	-	2
750.0368	Cu II	1	15.57	-	17.22	4p 5P	-	5d 3D	1	-	1
754.8271	Cu II	1	16.86	-	18.50	4f 1P	-	6d 1S	1	-	0
755.8569	Cu II	6	16.75	-	18.39	4p 3D	-	6d 1P	1	-	1
756.2015	Cu II	1028	13.65	-	15.29	5s 3D	-	5p 3D	1	-	1
756.4305	Cu II	2	15.32	-	16.96	5p 1D	-	5d 3P	2	-	1
756.4939	Cu II	1	14.34	-	15.98	4d 3P	-	4p 3D	2	-	2
756.5016	Cu II	11	16.99	-	18.63	5d 3F	-	4p 3F	3	-	2
756.8484	Cu II	4	14.33	-	15.97	4d 3G	-	4p 3F	5	-	4
757.5232	Cu II	13	15.32	-	16.96	5p 1D	-	5d 3P	2	-	2

757.5789	Cu II	1	14.60	-	16.24	4d 3G	-	4p 5D	3	-	2
757.9033	Cu II	1888	13.68	-	15.32	5s 1D	-	5p 1D	2	-	2
757.9843	Cu II	627	13.43	-	15.07	5s 3D	-	5p 3D	2	-	3
758.0053	Cu II	1	14.34	-	15.98	4d 3P	-	4p 3D	1	-	2
758.3341	Cu II	0	14.45	-	16.09	4d 3P	-	4p 3P	0	-	1
758.7332	Cu II	8	17.00	-	18.63	5d 3D	-	4p 3F	2	-	2
761.1928	Cu II	4	17.07	-	18.69	4p 1D	-	8s 3D	2	-	2
762.2477	Cu II	1	14.34	-	15.97	4d 3G	-	4p 3F	4	-	4
762.5434	Cu II	1	16.59	-	18.22	4p 3P	-	7s 1D	2	-	2
763.4923	Cu II	7	17.07	-	18.69	4p 1D	-	8s 3D	2	-	3
765.2333	Cu II	1453	13.39	-	15.01	5s 3D	-	4p 1F	3	-	3
765.4721	Cu II	0	16.59	-	18.21	4p 3P	-	7s 3D	2	-	1
766.4648	Cu II	1535	14.96	-	16.58	5p 3F	-	6s 3D	3	-	2
766.8073	Cu II	10	16.80	-	18.41	4p 3P	-	6d 3D	0	-	1
766.8530	Cu II	0	14.39	-	16.01	4d 3D	-	4p 3D	3	-	3
767.1267	Cu II	1	15.29	-	16.90	5p 3D	-	5d 3S	1	-	1
767.6190	Cu II	0	15.57	-	17.18	4p 5P	-	5d 1P	1	-	1
768.1788	Cu II	63	15.22	-	16.83	5p 3F	-	6s 1D	2	-	2
768.2941	Cu II	0	14.62	-	16.23	4d 3D	-	4p 5D	1	-	1
771.8694	Cu II	4	16.61	-	18.22	4p 3D	-	7s 1D	3	-	2
772.6648	Cu II	390	13.68	-	15.29	5s 1D	-	5p 3D	2	-	1
773.1619	Cu II	0	14.34	-	15.94	4d 3P	-	4p 3D	2	-	2
773.8664	Cu II	1151	15.22	-	16.82	5p 3F	-	6s 3D	2	-	1
774.4097	Cu II	159	14.96	-	16.56	5p 3F	-	6s 3D	3	-	3
774.4369	Cu II	1	16.83	-	18.43	4p 3F	-	6d 3F	3	-	2
775.1206	Cu II	5	16.83	-	18.43	4p 3F	-	6d 1F	3	-	3
775.1753	Cu II	2	14.34	-	15.94	4d 3G	-	4p 3F	4	-	3
775.4362	Cu II	534	15.23	-	16.83	5p 1P	-	6s 1D	1	-	2
776.3365	Cu II	7	16.80	-	18.39	4p 3P	-	6d 1P	0	-	1
776.8468	Cu II	18	17.10	-	18.69	4p 3S	-	8s 3D	1	-	2
777.3202	Cu II	81	13.39	-	14.99	5s 3D	-	4p 1D	3	-	2
777.8738	Cu II	672	14.99	-	16.58	4p 1D	-	6s 3D	2	-	2
779.2931	Cu II	1	14.69	-	16.28	4d 1F	-	4p 5D	3	-	3
779.4199	Cu II	1	16.83	-	18.42	4p 3F	-	6d 1D	3	-	2
780.5184	Cu II	723	14.99	-	16.58	5p 3P	-	6s 3D	1	-	2
780.7659	Cu II	2416	14.98	-	16.56	5p 3F	-	6s 3D	4	-	3
780.7913	Cu II	187	17.10	-	18.69	4p 3F	-	8s 3D	4	-	3
781.2317	Cu II	196	15.23	-	16.82	5p 1P	-	6s 3D	1	-	1
781.5828	Cu II	1	14.42	-	16.01	4d 3F	-	4p 3D	3	-	3
782.0576	Cu II	344	13.65	-	15.23	5s 3D	-	5p 1P	1	-	1
782.1181	Cu II	3	17.18	-	18.77	5d 1P	-	6f 3P	1	-	2
782.1278	Cu II	33	16.83	-	18.41	4p 3F	-	6d 1G	3	-	4

782.2334	Cu II	0	14.65	-	16.24	4d 1D	-	4p 5D	2	-	2
782.2532	Cu II	8	15.32	-	16.90	5p 1D	-	5d 3S	2	-	1
782.5654	Cu II	4150	13.39	-	14.98	5s 3D	-	5p 3F	3	-	4
782.6358	Cu II	0	14.39	-	15.98	4d 3D	-	4p 3D	3	-	2
784.1487	Cu II	1	16.59	-	18.17	4p 3P	-	6d 3D	2	-	2
784.2004	Cu II	2	16.83	-	18.41	4p 3F	-	6d 3G	3	-	3
784.3586	Cu II	0	14.43	-	16.01	4d 3F	-	4p 3D	4	-	3
784.4950	Cu II	11	13.43	-	15.01	5s 3D	-	4p 1F	2	-	3
784.5081	Cu II	1812	15.25	-	16.83	5p 1F	-	6s 1D	3	-	2
784.8373	Cu II	6	16.66	-	18.24	4p 3P	-	6d 3P	1	-	0
785.3016	Cu II	3	16.59	-	18.17	4p 3P	-	6d 3F	2	-	3
785.3820	Cu II	0	14.43	-	16.01	4d 3D	-	4p 3D	2	-	3
785.9964	Cu II	0	15.18	-	16.75	4d 1S	-	4p 3D	0	-	1
786.0577	Cu II	49	14.99	-	16.56	4p 1D	-	6s 3D	2	-	3
787.3201	Cu II	1	14.39	-	15.97	4d 3D	-	4p 3F	3	-	4
787.7919	Cu II	1	16.86	-	18.43	4p 5S	-	6d 3F	2	-	2
788.4994	Cu II	1	16.86	-	18.43	4p 5S	-	6d 1F	2	-	3
789.0376	Cu II	18	16.59	-	18.16	4p 3P	-	6d 3D	2	-	3
789.0567	Cu II	253	13.39	-	14.96	5s 3D	-	5p 3F	3	-	3
789.5805	Cu II	1968	13.65	-	15.22	5s 3D	-	5p 3F	1	-	2
790.2553	Cu II	3084	13.68	-	15.25	5s 1D	-	5p 1F	2	-	3
790.3663	Cu II	7	15.01	-	16.58	4p 1F	-	6s 3D	3	-	2
790.9810	Cu II	0	14.60	-	16.17	4d 3G	-	4p 3P	3	-	2
792.9488	Cu II	1	16.86	-	18.42	4p 5S	-	6d 1D	2	-	2
793.3055	Cu II	6	16.59	-	18.15	4p 3P	-	6d 3P	2	-	1
793.4359	Cu II	2	17.21	-	18.77	5d 3G	-	6f 3F	3	-	3
794.0141	Cu II	1	16.61	-	18.17	4p 3D	-	6d 3D	3	-	2
794.0829	Cu II	33	16.59	-	18.15	4p 3P	-	6d 3P	2	-	2
794.3866	Cu II	1	17.21	-	18.77	5d 3G	-	6f 3D	3	-	2
794.4438	Cu II	1295	13.43	-	14.99	5s 3D	-	5p 3P	2	-	1
794.4703	Cu II	0	17.21	-	18.77	5d 3G	-	6f 3F	3	-	3
794.5894	Cu II	0	16.85	-	18.41	4f 3H	-	6d 1G	5	-	4
794.9167	Cu II	3	16.66	-	18.22	4p 3P	-	7s 1D	1	-	2
794.9855	Cu II	146	16.61	-	18.17	4p 3D	-	6d 3F	3	-	4
795.1962	Cu II	1	16.61	-	18.17	4p 3D	-	6d 3F	3	-	3
795.3704	Cu II	0	16.86	-	18.41	4p 5S	-	6d 3D	2	-	1
797.2033	Cu II	1197	13.43	-	14.99	5s 3D	-	4p 1D	2	-	2
797.4760	Cu II	0	17.22	-	18.77	5d 1G	-	6f 3F	4	-	3
797.8972	Cu II	0	16.86	-	18.41	4p 5S	-	6d 3G	2	-	3
797.9704	Cu II	0	14.53	-	16.09	4d 1P	-	4p 3P	1	-	1
797.9842	Cu II	1	14.42	-	15.98	4d 3F	-	4p 3D	3	-	2
798.0999	Cu II	1	16.66	-	18.21	4p 3P	-	7s 3D	1	-	1

798.2075	Cu II	0	16.87	-	18.42	4f 3D	-	6d 1D	3	-	2
798.5209	Cu II	1	17.22	-	18.77	5d 1G	-	6f 3F	4	-	3
798.8163	Cu II	775	15.01	-	16.56	4p 1F	-	6s 3D	3	-	3
799.0272	Cu II	109	16.61	-	18.16	4p 3D	-	6d 3D	3	-	3
799.2937	Cu II	0	16.86	-	18.41	4f 3D	-	6d 3D	2	-	1
799.4214	Cu II	0	17.22	-	18.77	5d 3D	-	6f 3D	1	-	2
799.6790	Cu II	964	13.68	-	15.23	5s 1D	-	5p 1P	2	-	1
799.9171	Cu II	0	16.85	-	18.39	4f 3P	-	6d 1P	1	-	1
800.4573	Cu II	2	17.22	-	18.77	5d 1G	-	6f 3H	4	-	5
800.4893	Cu II	0	14.39	-	15.94	4d 3D	-	4p 3D	3	-	2
801.2557	Cu II	0	17.22	-	18.77	5d 3D	-	6f 3P	1	-	2
801.9449	Cu II	1	14.43	-	15.98	4d 3D	-	4p 3D	2	-	2
802.3575	Cu II	0	14.69	-	16.24	4d 1F	-	4p 5D	3	-	2
802.3859	Cu II	0	16.85	-	18.39	4f 3P	-	6d 1P	0	-	1
802.6479	Cu II	218	15.29	-	16.83	5p 3D	-	6s 1D	1	-	2
802.8547	Cu II	0	14.42	-	15.97	4d 3F	-	4p 3F	3	-	4
803.7096	Cu II	2	16.61	-	18.15	4p 3D	-	6d 3G	3	-	4
803.7808	Cu II	2	17.23	-	18.77	5d 1D	-	6f 3F	2	-	3
804.2014	Cu II	40	16.61	-	18.15	4p 3D	-	6d 3P	3	-	2
804.7564	Cu II	1	17.23	-	18.77	5d 1D	-	6f 3D	2	-	2
804.8423	Cu II	1	17.23	-	18.77	5d 1D	-	6f 3F	2	-	3
805.6724	Cu II	0	14.70	-	16.23	4d 3F	-	4p 5D	2	-	1
805.7839	Cu II	1	14.43	-	15.97	4d 3F	-	4p 3F	4	-	4
806.6153	Cu II	1	17.23	-	18.77	5d 1D	-	6f 3P	2	-	2
806.8993	Cu II	23	16.59	-	18.13	4p 3P	-	6d 3S	2	-	1
807.4995	Cu II	187	17.16	-	18.69	4p 3G	-	8s 3D	3	-	2
807.5463	Cu II	105	13.68	-	15.22	5s 1D	-	5p 3F	2	-	2
808.8590	Cu II	548	15.29	-	16.82	5p 3D	-	6s 3D	1	-	1
809.5523	Cu II	2518	13.43	-	14.96	5s 3D	-	5p 3F	2	-	3
810.0878	Cu II	37	17.16	-	18.69	4p 3G	-	8s 3D	3	-	3
812.8116	Cu II	0	17.25	-	18.77	5d 1F	-	6f 3F	3	-	3
813.8093	Cu II	0	17.25	-	18.77	5d 1F	-	6f 3D	3	-	2
813.8971	Cu II	1	17.25	-	18.77	5d 1F	-	6f 3F	3	-	3
814.3089	Cu II	0	17.25	-	18.77	5d 3F	-	6f 3F	2	-	3
814.7623	Cu II	2	16.85	-	18.37	4f 3P	-	5g 3F	1	-	2
815.3833	Cu II	13	16.70	-	18.22	4p 3D	-	7s 1D	2	-	2
815.3984	Cu II	1	17.25	-	18.77	5d 3F	-	6f 3F	2	-	3
815.7103	Cu II	0	17.25	-	18.77	5d 1F	-	6f 3P	3	-	2
816.5532	Cu II	1	14.42	-	15.94	4d 3F	-	4p 3D	3	-	2
817.2093	Cu II	1	14.42	-	15.94	4d 3F	-	4p 3F	3	-	3
817.2182	Cu II	1	17.25	-	18.77	5d 3F	-	6f 3P	2	-	2

817.8961	Cu II	0	14.65	-	16.17	4d 1D	-	4p 3P	2	-	2
817.8979	Cu II	1	16.85	-	18.37	4f 3P	-	5g 3F	2	-	2
817.9027	Cu II	6	16.85	-	18.37	4f 3P	-	5g 1F	2	-	3
818.4237	Cu II	8	16.66	-	18.17	4p 3P	-	6d 3D	1	-	2
818.4532	Cu II	1	16.85	-	18.37	4f 3H	-	5g 1I	6	-	6
818.6487	Cu II	0	16.85	-	18.37	4f 3H	-	5g 3I	5	-	5
818.7329	Cu II	2	16.70	-	18.21	4p 3D	-	7s 3D	2	-	1
819.2229	Cu II	1038	15.32	-	16.83	5p 1D	-	6s 1D	2	-	2
819.2336	Cu II	346	15.07	-	16.58	5p 3D	-	6s 3D	3	-	2
819.4018	Cu II	0	16.92	-	18.43	4p 3D	-	6d 3F	3	-	2
820.1673	Cu II	1	16.92	-	18.43	4p 3D	-	6d 1F	3	-	3
820.2444	Cu II	0	14.43	-	15.94	4d 3F	-	4p 3F	4	-	3
820.9494	Cu II	7	16.86	-	18.37	4f 3F	-	5g 1G	3	-	4
820.9514	Cu II	1	16.86	-	18.37	4f 3F	-	5g 3G	3	-	3
821.0863	Cu II	1	16.86	-	18.37	4f 1P	-	5g 3F	1	-	2
821.3636	Cu II	0	14.43	-	15.94	4d 3D	-	4p 3F	2	-	3
822.5760	Cu II	5	16.86	-	18.37	4f 3D	-	5g 3G	2	-	3
823.2314	Cu II	0	16.86	-	18.37	4f 3F	-	5g 3F	3	-	2
823.2363	Cu II	1	16.86	-	18.37	4f 3F	-	5g 1F	3	-	3
823.2380	Cu II	1	16.87	-	18.37	4f 3D	-	5g 3H	3	-	4
823.5279	Cu II	447	13.65	-	15.15	5s 3D	-	5p 3P	1	-	0
824.0246	Cu II	0	16.87	-	18.37	4f 3D	-	5g 3G	3	-	3
824.8652	Cu II	1	16.86	-	18.37	4f 3D	-	5g 3F	2	-	2
824.8701	Cu II	0	16.86	-	18.37	4f 3D	-	5g 1F	2	-	3
825.6941	Cu II	107	15.32	-	16.82	5p 1D	-	6s 3D	2	-	1
827.7560	Cu II	2075	13.39	-	14.89	5s 3D	-	5p 3P	3	-	2
828.0167	Cu II	1	16.92	-	18.41	4p 3D	-	6d 1G	3	-	4
828.3160	Cu II	828	15.07	-	16.56	5p 3D	-	6s 3D	3	-	3
828.4036	Cu II	9	16.66	-	18.15	4p 3P	-	6d 3P	1	-	1
829.8951	Cu II	1	16.88	-	18.37	4f 3F	-	5g 1H	4	-	5
829.8973	Cu II	1	16.88	-	18.37	4f 3F	-	5g 3H	4	-	4
830.0039	Cu II	0	15.57	-	17.06	4p 5P	-	5d 3P	1	-	0
830.6966	Cu II	0	16.88	-	18.37	4f 3F	-	5g 3G	4	-	3
831.8175	Cu II	2	16.88	-	18.37	4f 3G	-	5g 1H	5	-	5
831.8197	Cu II	0	16.88	-	18.37	4f 3G	-	5g 3H	5	-	4
832.2018	Cu II	9	16.88	-	18.37	4f 3F	-	5g 3I	4	-	5
832.6208	Cu II	0	16.88	-	18.37	4f 3G	-	5g 1G	5	-	4
833.8107	Cu II	23	17.01	-	18.50	4p 1P	-	6d 1S	1	-	0
834.1327	Cu II	11	16.88	-	18.37	4f 3G	-	5g 1I	5	-	6
834.1350	Cu II	1	16.88	-	18.37	4f 3G	-	5g 3I	5	-	5
835.7039	Cu II	7	16.75	-	18.24	4p 3D	-	6d 3P	1	-	0
839.4575	Cu II	1	16.90	-	18.37	4f 3G	-	5g 1H	4	-	5

839.4597	Cu II	1	16.90	-	18.37	4f 3G	-	5g 3H	4	-	4
839.9228	Cu II	1	14.69	-	16.17	4d 1F	-	4p 3P	3	-	2
840.1352	Cu II	8	16.70	-	18.17	4p 3D	-	6d 3D	2	-	2
840.2756	Cu II	0	16.90	-	18.37	4f 3G	-	5g 1G	4	-	4
840.2906	Cu II	121	13.65	-	15.12	5s 3D	-	5p 3D	1	-	2
841.4587	Cu II	9	16.70	-	18.17	4p 3D	-	6d 3F	2	-	3
841.8178	Cu II	1	16.90	-	18.37	4f 3G	-	5g 3I	4	-	5
843.2381	Cu II	2	16.66	-	18.13	4p 3P	-	6d 3S	1	-	1
843.3302	Cu II	0	14.70	-	16.17	4d 3F	-	4p 3P	2	-	2
844.1951	Cu II	8	16.96	-	18.43	4p 3F	-	6d 3F	2	-	2
844.9671	Cu II	0	14.62	-	16.09	4d 3D	-	4p 3P	1	-	1
845.0076	Cu II	127	16.96	-	18.43	4p 3F	-	6d 1F	2	-	3
845.7496	Cu II	8	16.70	-	18.16	4p 3D	-	6d 3D	2	-	3
846.0165	Cu II	15	16.71	-	18.17	4p 3H	-	6d 3F	4	-	4
846.1946	Cu II	2	15.53	-	16.99	4p 5P	-	5d 3F	3	-	4
846.2551	Cu II	1	16.71	-	18.17	4p 3H	-	6d 3F	4	-	3
846.8891	Cu II	0	15.53	-	16.99	4p 5P	-	5d 3F	3	-	3
847.1417	Cu II	2	16.75	-	18.22	4p 3D	-	7s 1D	1	-	2
848.1836	Cu II	0	15.53	-	16.99	4p 5P	-	5d 3F	2	-	3
850.1196	Cu II	42	16.96	-	18.42	4p 3F	-	6d 1D	2	-	2
850.3396	Cu II	111	13.43	-	14.89	5s 3D	-	5p 3P	2	-	2
850.5585	Cu II	75	17.24	-	18.69	4p 3D	-	8s 3D	2	-	2
850.5952	Cu II	4	16.71	-	18.16	4p 3H	-	6d 3D	4	-	3
850.6550	Cu II	0	16.70	-	18.15	4p 3D	-	6d 3P	2	-	1
850.7579	Cu II	10	16.75	-	18.21	4p 3D	-	7s 3D	1	-	1
851.1061	Cu II	481	15.12	-	16.58	5p 3D	-	6s 3D	2	-	2
851.5488	Cu II	7	16.70	-	18.15	4p 3D	-	6d 3P	2	-	2
852.9036	Cu II	2	16.96	-	18.41	4p 3F	-	6d 3D	2	-	1
853.4306	Cu II	120	17.24	-	18.69	4p 3D	-	8s 3D	2	-	3
855.2519	Cu II	2	15.53	-	16.98	4p 5P	-	5d 3D	3	-	3
855.8099	Cu II	25	16.96	-	18.41	4p 3F	-	6d 3G	2	-	3
855.9034	Cu II	3	16.71	-	18.15	4p 3H	-	6d 3G	4	-	4
855.9833	Cu II	8	16.90	-	18.35	5d 3S	-	5f 3D	1	-	1
856.5722	Cu II	0	15.53	-	16.98	4p 5P	-	5d 3D	2	-	3
857.4080	Cu II	57	16.71	-	18.15	4p 3H	-	6d 3G	4	-	5
857.6295	Cu II	1	17.18	-	18.63	5d 1P	-	4p 3F	1	-	2
858.0995	Cu II	46	16.90	-	18.35	5d 3S	-	5f 1D	1	-	2
860.1532	Cu II	1	14.53	-	15.98	4d 1P	-	4p 3D	1	-	2
860.6679	Cu II	231	13.68	-	15.12	5s 1D	-	5p 3D	2	-	2
860.9134	Cu II	11	15.12	-	16.56	5p 3D	-	6s 3D	2	-	3
862.0938	Cu II	1	14.65	-	16.09	4d 1D	-	4p 3P	2	-	1
864.4931	Cu II	0	17.00	-	18.43	4p 3G	-	6d 3F	3	-	2

864.7093	Cu II	13	16.96	-	18.39	4p 3F	-	6d 1P	2	-	1
865.3452	Cu II	1	17.00	-	18.43	4p 3G	-	6d 1F	3	-	3
866.2136	Cu II	100	17.26	-	18.69	6p 3P	-	8s 3D	1	-	2
866.3046	Cu II	8	16.70	-	18.13	4p 3D	-	6d 3S	2	-	1
866.6693	Cu II	1	15.53	-	16.96	4p 5P	-	5d 3G	3	-	4
867.3960	Cu II	63	17.26	-	18.69	6p 3P	-	8s 3D	2	-	3
867.5154	Cu II	1	15.53	-	16.96	4p 5P	-	5d 3P	3	-	2
868.8739	Cu II	1	15.53	-	16.96	4p 5P	-	5d 3P	2	-	2
870.7070	Cu II	0	17.00	-	18.42	4p 3G	-	6d 1D	3	-	2
873.0232	Cu II	74	17.28	-	18.69	6p 3F	-	8s 3D	3	-	2
873.8909	Cu II	10	16.75	-	18.17	4p 3D	-	6d 3D	1	-	2
874.0877	Cu II	2	17.00	-	18.41	4p 3G	-	6d 1G	3	-	4
874.5886	Cu II	5	17.21	-	18.63	5d 3G	-	4p 3F	3	-	2
874.6725	Cu II	11	17.01	-	18.43	4p 1P	-	6d 3F	1	-	2
876.0494	Cu II	156	17.28	-	18.69	6p 3F	-	8s 3D	3	-	3
876.6771	Cu II	0	17.00	-	18.41	4p 3G	-	6d 3G	3	-	3
876.7914	Cu II	7	16.80	-	18.21	4p 3P	-	7s 3D	0	-	1
880.6953	Cu II	1	17.22	-	18.63	5d 3D	-	4p 3F	1	-	2
880.9028	Cu II	2	16.96	-	18.36	5d 3P	-	5f 3G	2	-	3
881.0341	Cu II	13	17.01	-	18.42	4p 1P	-	6d 1D	1	-	2
881.0399	Cu II	187	17.28	-	18.69	6p 3F	-	8s 3D	4	-	3
881.5924	Cu II	3	16.96	-	18.36	5d 3G	-	5f 1G	4	-	4
881.7673	Cu II	0	14.53	-	15.94	4d 1P	-	4p 3D	1	-	2
881.7770	Cu II	0	16.96	-	18.36	5d 3G	-	5f 3G	4	-	3
883.7822	Cu II	1	16.96	-	18.36	5d 3P	-	5f 3F	2	-	2
884.0246	Cu II	10	17.01	-	18.41	4p 1P	-	6d 3D	1	-	1
884.1372	Cu II	2	16.96	-	18.36	5d 3P	-	5f 1F	2	-	3
884.4877	Cu II	1	16.95	-	18.35	5d 3G	-	5f 1H	5	-	5
885.0178	Cu II	1	16.96	-	18.36	5d 3G	-	5f 1F	4	-	3
885.2741	Cu II	1	16.96	-	18.36	5d 3P	-	5f 3F	1	-	2
885.2788	Cu II	10	16.75	-	18.15	4p 3D	-	6d 3P	1	-	1
885.5624	Cu II	1	17.10	-	18.50	4p 3S	-	6d 1S	1	-	0
887.1745	Cu II	1	17.23	-	18.63	5d 1D	-	4p 3F	2	-	2
887.5899	Cu II	17	16.96	-	18.35	5d 3G	-	5f 1H	4	-	5
887.6943	Cu II	0	16.96	-	18.35	5d 3P	-	5f 3D	2	-	1
889.1994	Cu II	1	16.96	-	18.35	5d 3P	-	5f 3D	1	-	1
889.8982	Cu II	1	16.85	-	18.24	4f 3P	-	6d 3P	1	-	0
889.9704	Cu II	1	16.96	-	18.35	5d 3P	-	5f 1D	2	-	2
890.3985	Cu II	0	14.70	-	16.09	4d 3F	-	4p 3P	2	-	1
891.4833	Cu II	0	16.96	-	18.35	5d 3P	-	5f 1D	1	-	2
892.3816	Cu II	14	16.83	-	18.22	4p 3F	-	7s 1D	3	-	2

893.7289	Cu II	12	16.98	-	18.36	5d 3D	-	5f 1G	3	-	4
893.9186	Cu II	1	16.98	-	18.36	5d 3D	-	5f 3G	3	-	3
894.6713	Cu II	5	17.11	-	18.50	4f 3D	-	6d 1S	1	-	0
895.9155	Cu II	7	13.68	-	15.07	5s 1D	-	5p 3D	2	-	3
896.7140	Cu II	20	17.01	-	18.39	4p 1P	-	6d 1P	1	-	1
896.8838	Cu II	0	16.98	-	18.36	5d 3D	-	5f 3F	3	-	2
897.2494	Cu II	1	16.98	-	18.36	5d 3D	-	5f 1F	3	-	3
897.4477	Cu II	4	16.86	-	18.24	4f 1P	-	6d 3P	1	-	0
898.1894	Cu II	3	17.25	-	18.63	5d 1F	-	4p 3F	3	-	2
899.5018	Cu II	1	16.98	-	18.35	5d 3D	-	5f 3H	3	-	4
900.0180	Cu II	3	17.25	-	18.63	5d 3F	-	4p 3F	2	-	2
900.1106	Cu II	1	14.60	-	15.98	4d 3G	-	4p 3D	3	-	2
901.5654	Cu II	0	15.53	-	16.90	4p 5P	-	5d 3S	2	-	1
902.2410	Cu II	3	16.75	-	18.13	4p 3D	-	6d 3S	1	-	1
902.9344	Cu II	20	17.32	-	18.69	6p 3D	-	8s 3D	2	-	2
903.0475	Cu II	0	16.99	-	18.36	5d 3F	-	5f 1G	3	-	4
903.2411	Cu II	6	16.99	-	18.36	5d 3F	-	5f 3G	3	-	3
903.8384	Cu II	4	16.99	-	18.36	5d 3F	-	5f 1G	4	-	4
904.0324	Cu II	0	16.99	-	18.36	5d 3F	-	5f 3G	4	-	3
904.1511	Cu II	1	16.59	-	17.96	4p 3P	-	7s 3D	2	-	2
905.4374	Cu II	22	17.33	-	18.69	6p 3D	-	8s 3D	3	-	2
906.1719	Cu II	3	17.32	-	18.69	6p 3D	-	8s 3D	2	-	3
906.2686	Cu II	1	16.99	-	18.36	5d 3F	-	5f 3F	3	-	2
906.4242	Cu II	0	17.00	-	18.36	5d 3D	-	5f 3G	2	-	3
906.7975	Cu II	17	13.39	-	14.76	5s 3D	-	4p 3F	3	-	2
907.4392	Cu II	1	16.99	-	18.36	5d 3F	-	5f 1F	4	-	3
908.6928	Cu II	58	17.33	-	18.69	6p 3D	-	8s 3D	3	-	3
908.9418	Cu II	25	16.99	-	18.35	5d 3F	-	5f 3H	3	-	4
909.3633	Cu II	15	17.07	-	18.43	4p 1D	-	6d 3F	2	-	2
909.4731	Cu II	4	17.00	-	18.36	5d 3D	-	5f 3F	2	-	2
909.7431	Cu II	1	16.99	-	18.35	5d 3F	-	5f 3H	4	-	4
909.7520	Cu II	35	16.59	-	17.95	4p 3P	-	7s 3D	2	-	3
909.8491	Cu II	23	17.00	-	18.36	5d 3D	-	5f 1F	2	-	3
910.1435	Cu II	13	16.99	-	18.35	5d 3F	-	5f 1H	4	-	5
910.1609	Cu II	1	16.86	-	18.22	4p 5S	-	7s 1D	2	-	2
910.3062	Cu II	78	17.07	-	18.43	4p 1D	-	6d 1F	2	-	3
910.3234	Cu II	66	15.22	-	16.58	5p 3F	-	6s 3D	2	-	2
910.7881	Cu II	10	13.39	-	14.75	5s 3D	-	4p 1G	3	-	4
913.6165	Cu II	1	17.00	-	18.35	5d 3D	-	5f 3D	2	-	1
914.3364	Cu II	0	16.86	-	18.21	4p 5S	-	7s 3D	2	-	1
915.0116	Cu II	1	14.62	-	15.98	4d 3D	-	4p 3D	1	-	2
916.0277	Cu II	5	17.00	-	18.35	5d 3D	-	5f 1D	2	-	2

916.2416	Cu II	25	17.07	-	18.42	4p 1D	-	6d 1D	2	-	2
917.2923	Cu II	8	16.61	-	17.96	4p 3D	-	7s 3D	3	-	2
917.6050	Cu II	0	14.62	-	15.97	4d 1G	-	4p 3F	4	-	4
919.4763	Cu II	3	17.07	-	18.41	4p 1D	-	6d 3D	2	-	1
920.5323	Cu II	69	15.23	-	16.58	5p 1P	-	6s 3D	1	-	2
921.5519	Cu II	3	15.22	-	16.56	5p 3F	-	6s 3D	2	-	3
922.1142	Cu II	3	16.83	-	18.17	4p 3F	-	6d 3D	3	-	2
922.6737	Cu II	46	13.65	-	14.99	5s 3D	-	5p 3P	1	-	1
922.8549	Cu II	1	17.07	-	18.41	4p 1D	-	6d 3G	2	-	3
923.0578	Cu II	118	16.61	-	17.95	4p 3D	-	7s 3D	3	-	3
923.1421	Cu II	0	16.09	-	17.43	4p 3P	-	5d 1S	1	-	0
923.4246	Cu II	391	16.83	-	18.17	4p 3F	-	6d 3F	3	-	4
923.7088	Cu II	1	16.83	-	18.17	4p 3F	-	6d 3F	3	-	3
923.8071	Cu II	4	14.60	-	15.94	4d 3G	-	4p 3D	3	-	2
924.6470	Cu II	0	14.60	-	15.94	4d 3G	-	4p 3F	3	-	3
926.3975	Cu II	8	13.65	-	14.99	5s 3D	-	4p 1D	1	-	2
928.8822	Cu II	272	16.83	-	18.16	4p 3F	-	6d 3D	3	-	3
931.5749	Cu II	0	16.80	-	18.13	4p 3P	-	6d 3S	0	-	1
931.7944	Cu II	224	17.10	-	18.43	4p 3S	-	6d 3F	1	-	2
932.1412	Cu II	6	16.84	-	18.17	4p 3F	-	6d 3F	4	-	4
932.4309	Cu II	0	16.84	-	18.17	4p 3F	-	6d 3F	4	-	3
933.1896	Cu II	112	13.68	-	15.01	5s 1D	-	4p 1F	2	-	3
933.2118	Cu II	11	17.07	-	18.39	4p 1D	-	6d 1P	2	-	1
933.3272	Cu II	1	16.85	-	18.17	4f 3P	-	6d 3D	1	-	2
933.3448	Cu II	3	15.25	-	16.58	5p 1F	-	6s 3D	3	-	2
933.9714	Cu II	33	13.43	-	14.76	5s 3D	-	4p 3F	2	-	2
935.0054	Cu II	1	17.10	-	18.43	4p 3F	-	6d 1F	4	-	3
935.1291	Cu II	1	14.65	-	15.98	4d 1D	-	4p 3D	2	-	2
935.2162	Cu II	0	16.83	-	18.15	4p 3F	-	6d 3G	3	-	4
935.8822	Cu II	97	16.83	-	18.15	4p 3F	-	6d 3P	3	-	2
936.4043	Cu II	4	14.62	-	15.94	4d 1G	-	4p 3F	4	-	3
937.4440	Cu II	3	16.85	-	18.17	4f 3P	-	6d 3D	2	-	2
937.7027	Cu II	2	16.84	-	18.16	4p 3F	-	6d 3D	4	-	3
939.0176	Cu II	105	17.10	-	18.42	4p 3S	-	6d 1D	1	-	2
939.0922	Cu II	0	16.85	-	18.17	4f 3P	-	6d 3F	2	-	3
939.5098	Cu II	0	14.62	-	15.94	4d 3D	-	4p 3D	1	-	2
939.6266	Cu II	1	17.11	-	18.43	4f 1D	-	6d 3F	2	-	2
940.6333	Cu II	1	17.11	-	18.43	4f 1D	-	6d 1F	2	-	3
940.8455	Cu II	1	16.85	-	18.17	4f 3H	-	6d 3F	5	-	4
941.1105	Cu II	1	16.86	-	18.17	4p 5S	-	6d 3D	2	-	2
941.6350	Cu II	1	16.86	-	18.17	4f 1P	-	6d 3D	1	-	2
941.8846	Cu II	1	17.11	-	18.43	4f 3D	-	6d 3F	1	-	2

942.4154	Cu II	131	17.10	-	18.41	4p 3S	-	6d 3D	1	-	1
944.1580	Cu II	1	16.84	-	18.15	4p 3F	-	6d 3G	4	-	4
944.4397	Cu II	2	16.85	-	18.16	4f 3P	-	6d 3D	2	-	3
944.4573	Cu II	4	16.86	-	18.17	4f 3F	-	6d 3D	3	-	2
944.6769	Cu II	1	15.94	-	17.25	4p 3F	-	5d 3F	3	-	2
945.1521	Cu II	7	15.25	-	16.56	5p 1F	-	6s 3D	3	-	3
945.2204	Cu II	2	17.10	-	18.41	4p 3F	-	6d 1G	4	-	4
945.5552	Cu II	2	15.94	-	17.25	4p 3D	-	5d 3F	2	-	2
945.8320	Cu II	2	16.86	-	18.17	4f 3F	-	6d 3F	3	-	4
945.9891	Cu II	20	16.84	-	18.15	4p 3F	-	6d 3G	4	-	5
946.1303	Cu II	1	16.86	-	18.17	4f 3F	-	6d 3F	3	-	3
946.6082	Cu II	4	16.86	-	18.17	4f 3D	-	6d 3D	2	-	2
946.6999	Cu II	3	15.94	-	17.25	4p 3F	-	5d 1F	3	-	3
946.9722	Cu II	4	17.11	-	18.42	4f 1D	-	6d 1D	2	-	2
947.1124	Cu II	0	17.12	-	18.43	4f 3H	-	6d 1F	4	-	3
947.3011	Cu II	32	13.68	-	14.99	5s 1D	-	5p 3P	2	-	1
947.4346	Cu II	3	16.85	-	18.15	4f 3P	-	6d 3P	1	-	2
947.5820	Cu II	4	15.94	-	17.25	4p 3D	-	5d 1F	2	-	3
948.1613	Cu II	7	16.86	-	18.16	4p 5S	-	6d 3D	2	-	3
948.2492	Cu II	0	17.10	-	18.41	4p 3F	-	6d 3G	4	-	3
948.2889	Cu II	1	16.86	-	18.17	4f 3D	-	6d 3F	2	-	3
948.5271	Cu II	11	16.87	-	18.17	4f 3D	-	6d 3D	3	-	2
949.2656	Cu II	1	17.11	-	18.42	4f 3D	-	6d 1D	1	-	2
949.4018	Cu II	2	15.53	-	16.83	4p 5P	-	6s 1D	3	-	2
949.7855	Cu II	1	16.85	-	18.15	4f 3P	-	6d 3P	0	-	1
949.9137	Cu II	1	16.87	-	18.17	4f 3D	-	6d 3F	3	-	4
950.0262	Cu II	10	16.66	-	17.96	4p 3P	-	7s 3D	1	-	2
950.2145	Cu II	7	16.87	-	18.17	4f 3D	-	6d 3F	3	-	3
950.4279	Cu II	1	17.11	-	18.41	4f 1D	-	6d 3D	2	-	1
950.5608	Cu II	1	16.85	-	18.15	4f 3P	-	6d 3P	2	-	1
951.0291	Cu II	5	15.53	-	16.83	4p 5P	-	6s 1D	2	-	2
951.0369	Cu II	1	17.13	-	18.43	4f 1F	-	6d 3F	3	-	2
951.2268	Cu II	114	13.68	-	14.99	5s 1D	-	4p 1D	2	-	2
951.5585	Cu II	10	16.86	-	18.16	4f 3F	-	6d 3D	3	-	3
951.6771	Cu II	6	16.85	-	18.15	4f 3P	-	6d 3P	2	-	2
952.0683	Cu II	6	17.13	-	18.43	4f 1F	-	6d 1F	3	-	3
952.6179	Cu II	1	16.92	-	18.22	4p 3D	-	7s 1D	3	-	2
952.7381	Cu II	2	17.11	-	18.41	4f 3D	-	6d 3D	1	-	1
953.0892	Cu II	32	16.85	-	18.15	4f 3H	-	6d 3G	5	-	4
953.7420	Cu II	1	16.86	-	18.16	4f 3D	-	6d 3D	2	-	3
954.3309	Cu II	2	16.86	-	18.15	4p 5S	-	6d 3P	2	-	1

954.6923	Cu II	40	16.85	-	18.15	4f 3H	-	6d 3G	6	-	5
954.8703	Cu II	5	16.86	-	18.15	4f 1P	-	6d 3P	1	-	1
954.9552	Cu II	1	16.85	-	18.15	4f 3H	-	6d 3G	5	-	5
955.2485	Cu II	5	17.13	-	18.43	4f 3F	-	6d 3F	2	-	2
955.4561	Cu II	11	16.86	-	18.15	4p 5S	-	6d 3P	2	-	2
955.6899	Cu II	2	16.87	-	18.16	4f 3D	-	6d 3D	3	-	3
955.9967	Cu II	1	16.86	-	18.15	4f 1P	-	6d 3P	1	-	2
956.2890	Cu II	1	17.13	-	18.43	4f 3F	-	6d 1F	2	-	3
956.5611	Cu II	33	17.12	-	18.41	4f 1H	-	6d 1G	5	-	4
956.8501	Cu II	6	17.10	-	18.39	4p 3S	-	6d 1P	1	-	1
957.5952	Cu II	1	17.12	-	18.41	4f 3H	-	6d 1G	4	-	4
958.2067	Cu II	0	16.86	-	18.15	4f 3F	-	6d 3G	3	-	4
958.5627	Cu II	16	17.13	-	18.42	4f 1F	-	6d 1D	3	-	2
958.7911	Cu II	2	16.88	-	18.17	4f 3F	-	6d 3F	4	-	4
958.9058	Cu II	7	16.86	-	18.15	4f 3F	-	6d 3P	3	-	2
959.0975	Cu II	16	16.88	-	18.17	4f 3F	-	6d 3F	4	-	3
959.1336	Cu II	1	15.29	-	16.58	5p 3D	-	6s 3D	1	-	2
959.2529	Cu II	1	15.94	-	17.23	4p 3F	-	5d 1D	3	-	2
959.7612	Cu II	1	15.53	-	16.82	4p 5P	-	6s 3D	2	-	1
959.9846	Cu II	7	16.86	-	18.15	4f 3D	-	6d 3P	2	-	1
960.1585	Cu II	2	15.94	-	17.23	4p 3D	-	5d 1D	2	-	2
960.2369	Cu II	24	17.14	-	18.43	4f 1G	-	6d 1F	4	-	3
960.7039	Cu II	27	17.12	-	18.41	4f 3H	-	6d 3G	4	-	3
960.7315	Cu II	1	14.65	-	15.94	4d 1D	-	4p 3D	2	-	2
961.0897	Cu II	298	17.06	-	18.35	5d 3P	-	5f 3D	0	-	1
961.1231	Cu II	4	16.86	-	18.15	4f 3D	-	6d 3P	2	-	2
961.3580	Cu II	27	16.88	-	18.17	4f 3G	-	6d 3F	5	-	4
961.6399	Cu II	0	14.65	-	15.94	4d 1D	-	4p 3F	2	-	3
962.3961	Cu II	1	16.87	-	18.15	4f 3D	-	6d 3G	3	-	4
962.6617	Cu II	1	17.13	-	18.41	4f 1F	-	6d 1G	3	-	4
962.8414	Cu II	1	17.13	-	18.42	4f 3F	-	6d 1D	2	-	2
963.0428	Cu II	16	17.14	-	18.43	4f 3G	-	6d 3F	3	-	2
963.1014	Cu II	3	16.87	-	18.15	4f 3D	-	6d 3P	3	-	2
964.0344	Cu II	2	14.69	-	15.98	4d 1F	-	4p 3D	3	-	2
964.1004	Cu II	1	17.14	-	18.43	4f 3G	-	6d 1F	3	-	3
964.6760	Cu II	4	16.88	-	18.16	4f 3F	-	6d 3D	4	-	3
965.1110	Cu II	10	17.11	-	18.39	4f 1D	-	6d 1P	2	-	1
965.7362	Cu II	6	16.85	-	18.13	4f 3P	-	6d 3S	1	-	1
966.4141	Cu II	10	17.13	-	18.41	4f 3F	-	6d 3D	2	-	1
967.4933	Cu II	3	17.11	-	18.39	4f 3D	-	6d 1P	1	-	1
967.8649	Cu II	0	15.94	-	17.22	4p 3D	-	5d 3D	2	-	1
968.3897	Cu II	13	15.94	-	17.22	4p 3F	-	5d 1G	3	-	4

968.5258	Cu II	1	14.70	-	15.98	4d 3F	-	4p 3D	2	-	2
968.8619	Cu II	29	13.68	-	14.96	5s 1D	-	5p 3F	2	-	3
969.3369	Cu II	3	16.85	-	18.13	4f 3P	-	6d 3S	0	-	1
970.1446	Cu II	5	16.85	-	18.13	4f 3P	-	6d 3S	2	-	1
970.1471	Cu II	1	17.13	-	18.41	4f 3F	-	6d 3G	2	-	3
970.7606	Cu II	2	17.14	-	18.42	4f 3G	-	6d 1D	3	-	2
971.0139	Cu II	4	17.14	-	18.41	4f 1G	-	6d 1G	4	-	4
971.5094	Cu II	6	16.88	-	18.15	4f 3F	-	6d 3G	4	-	4
971.5774	Cu II	8	16.90	-	18.17	4f 3G	-	6d 3F	4	-	4
971.7396	Cu II	3	15.98	-	17.25	4p 3D	-	5d 3F	2	-	2
971.8921	Cu II	7	16.90	-	18.17	4f 3G	-	6d 3F	4	-	3
973.4483	Cu II	1	16.88	-	18.15	4f 3F	-	6d 3G	4	-	5
973.8804	Cu II	4	15.98	-	17.25	4p 3D	-	5d 1F	2	-	3
974.0719	Cu II	8	16.86	-	18.13	4p 5S	-	6d 3S	2	-	1
974.1450	Cu II	1	16.88	-	18.15	4f 3G	-	6d 3G	5	-	4
974.2105	Cu II	0	17.14	-	18.41	4f 1G	-	6d 3G	4	-	3
974.4146	Cu II	1	15.94	-	17.21	4p 3F	-	5d 3G	3	-	3
974.6338	Cu II	0	16.86	-	18.13	4f 1P	-	6d 3S	1	-	1
974.9648	Cu II	0	17.14	-	18.41	4f 3G	-	6d 1G	3	-	4
975.3491	Cu II	14	15.94	-	17.21	4p 3D	-	5d 3G	2	-	3
976.0944	Cu II	6	16.88	-	18.15	4f 3G	-	6d 3G	5	-	5
976.2441	Cu II	2	17.16	-	18.43	4p 3G	-	6d 3F	3	-	2
977.3309	Cu II	18	17.16	-	18.43	4p 3G	-	6d 1F	3	-	3
977.6209	Cu II	13	16.90	-	18.16	4f 3G	-	6d 3D	4	-	3
978.1874	Cu II	3	17.14	-	18.41	4f 3G	-	6d 3G	3	-	3
979.2475	Cu II	152	16.85	-	18.11	4f 3P	-	5g 1D	1	-	2
979.4067	Cu II	10	16.70	-	17.96	4p 3D	-	7s 3D	2	-	2
981.0050	Cu II	0	15.57	-	16.83	4p 5P	-	6s 1D	1	-	2
981.3213	Cu II	2704	16.85	-	18.11	4f 3P	-	5g 3D	1	-	2
981.3322	Cu II	1170	16.85	-	18.11	4f 3P	-	5g 3D	1	-	1
981.5993	Cu II	0	17.13	-	18.39	4f 3F	-	6d 1P	2	-	1
981.7399	Cu II	6	16.85	-	18.11	4f 3P	-	5g 3F	2	-	3
982.4241	Cu II	74	16.85	-	18.12	4f 3H	-	5g 3G	6	-	5
982.7025	Cu II	1	16.85	-	18.12	4f 3H	-	5g 3G	5	-	5
982.7047	Cu II	63	16.85	-	18.12	4f 3H	-	5g 3G	5	-	4
982.7978	Cu II	1521	16.85	-	18.11	4f 3H	-	5g 3H	6	-	6
982.8013	Cu II	20	16.85	-	18.11	4f 3H	-	5g 3H	6	-	5
982.8972	Cu II	16	15.32	-	16.58	5p 1D	-	6s 3D	2	-	2
983.0798	Cu II	11	16.85	-	18.11	4f 3H	-	5g 3H	5	-	6
983.0800	Cu II	1295	16.85	-	18.11	4f 3H	-	5g 3H	5	-	5
983.5296	Cu II	2	17.11	-	18.37	4f 1D	-	5g 3G	2	-	3
983.7804	Cu II	214	16.85	-	18.11	4f 3P	-	5g 1D	2	-	2

983.7837	Cu II	4624	16.85	-	18.11	4f 3P	-	5g 3D	2	-	3
983.9767	Cu II	0	16.85	-	18.11	4f 3H	-	5g 3F	5	-	4
984.1758	Cu II	1	17.16	-	18.42	4p 3G	-	6d 1D	3	-	2
984.6395	Cu II	1	16.90	-	18.15	4f 3G	-	6d 3G	4	-	4
985.0503	Cu II	1337	16.85	-	18.11	4f 3P	-	5g 3D	0	-	1
985.8733	Cu II	1660	16.85	-	18.11	4f 3P	-	5g 3D	2	-	2
985.8844	Cu II	176	16.85	-	18.11	4f 3P	-	5g 3D	2	-	1
985.9822	Cu II	7	16.70	-	17.95	4p 3D	-	7s 3D	2	-	3
986.1331	Cu II	173	16.85	-	18.11	4f 3H	-	5g 3I	6	-	6
986.2938	Cu II	45	16.96	-	18.22	4p 3F	-	7s 1D	2	-	2
986.6312	Cu II	1	16.90	-	18.15	4f 3G	-	6d 3G	4	-	5
986.8040	Cu II	461	17.11	-	18.37	4f 1D	-	5g 3F	2	-	2
986.8110	Cu II	6209	17.11	-	18.37	4f 1D	-	5g 1F	2	-	3
987.0804	Cu II	39	13.39	-	14.65	5s 3D	-	4p 3F	3	-	3
987.1696	Cu II	1	15.98	-	17.23	4p 3D	-	5d 1D	2	-	2
988.0765	Cu II	60	16.92	-	18.17	4p 3D	-	6d 3F	3	-	4
988.1461	Cu II	11	16.86	-	18.12	4f 3F	-	5g 3G	3	-	4
988.3716	Cu II	932	17.12	-	18.37	4f 1H	-	5g 1H	5	-	5
988.3747	Cu II	18	17.12	-	18.37	4f 1H	-	5g 3H	5	-	4
988.3969	Cu II	3027	16.86	-	18.11	4f 1P	-	5g 1D	1	-	2
988.4020	Cu II	3	16.92	-	18.17	4p 3D	-	6d 3F	3	-	3
988.4972	Cu II	52	17.16	-	18.41	4p 3G	-	6d 1G	3	-	4
989.2947	Cu II	3991	17.11	-	18.37	4f 3D	-	5g 3F	1	-	2
989.3512	Cu II	0	15.97	-	17.22	4p 3F	-	5d 1G	4	-	4
989.4322	Cu II	6472	16.86	-	18.11	4f 3F	-	5g 3F	3	-	4
989.4344	Cu II	296	16.86	-	18.11	4f 3F	-	5g 3F	3	-	3
989.4756	Cu II	10	17.12	-	18.37	4f 3H	-	5g 1H	4	-	5
989.4787	Cu II	768	17.12	-	18.37	4f 3H	-	5g 3H	4	-	4
989.5059	Cu II	28	17.12	-	18.37	4f 1H	-	5g 1G	5	-	4
990.2990	Cu II	1	15.57	-	16.82	4p 5P	-	6s 3D	1	-	1
990.5096	Cu II	629	16.86	-	18.11	4f 1P	-	5g 3D	1	-	2
990.5208	Cu II	337	16.86	-	18.11	4f 1P	-	5g 3D	1	-	1
990.6125	Cu II	1	17.12	-	18.37	4f 3H	-	5g 1G	4	-	4
990.6153	Cu II	23	17.12	-	18.37	4f 3H	-	5g 3G	4	-	3
991.1990	Cu II	37	16.96	-	18.21	4p 3F	-	7s 3D	2	-	1
991.2670	Cu II	1	14.69	-	15.94	4d 1F	-	4p 3D	3	-	2
991.5070	Cu II	88	16.86	-	18.11	4f 3F	-	5g 1D	3	-	2
991.5103	Cu II	2234	16.86	-	18.11	4f 3F	-	5g 3D	3	-	3
991.6452	Cu II	207	17.12	-	18.37	4f 1H	-	5g 3I	5	-	5
991.7954	Cu II	4820	16.86	-	18.11	4f 3D	-	5g 3F	2	-	3
991.8102	Cu II	1	17.16	-	18.41	4p 3G	-	6d 3G	3	-	3

992.2341	Cu II	1	14.69	-	15.94	4d 1F	-	4p 3F	3	-	3
992.5594	Cu II	0		-			-			-	
992.5740	Cu II	32	16.71	-	17.95	4p 3H	-	7s 3D	4	-	3
992.6020	Cu II	7080	16.87	-	18.12	4f 3D	-	5g 3G	3	-	4
993.6329	Cu II	207	16.86	-	18.11	4f 3F	-	5g 3D	3	-	2
993.8778	Cu II	1542	16.86	-	18.11	4f 3D	-	5g 1D	2	-	2
993.8812	Cu II	165	16.86	-	18.11	4f 3D	-	5g 3D	2	-	3
993.8998	Cu II	133	16.87	-	18.11	4f 3D	-	5g 3F	3	-	4
993.9019	Cu II	1897	16.87	-	18.11	4f 3D	-	5g 3F	3	-	3
994.3276	Cu II	54	16.92	-	18.16	4p 3D	-	6d 3D	3	-	3
994.8892	Cu II	10	17.13	-	18.37	4f 1F	-	5g 3H	3	-	4
995.3174	Cu II	1	15.98	-	17.22	4p 3D	-	5d 3D	2	-	1
995.9933	Cu II	147	16.87	-	18.11	4f 3D	-	5g 1D	3	-	2
995.9967	Cu II	21	16.87	-	18.11	4f 3D	-	5g 3D	3	-	3
996.0003	Cu II	10	15.32	-	16.56	5p 1D	-	6s 3D	2	-	3
996.0140	Cu II	0	16.86	-	18.11	4f 3D	-	5g 3D	2	-	2
996.0164	Cu II	1	14.70	-	15.94	4d 3F	-	4p 3D	2	-	2
996.0253	Cu II	102	16.86	-	18.11	4f 3D	-	5g 3D	2	-	1
996.0354	Cu II	7925	17.13	-	18.37	4f 1F	-	5g 1G	3	-	4
996.0382	Cu II	331	17.13	-	18.37	4f 1F	-	5g 3G	3	-	3
996.9929	Cu II	0	14.70	-	15.94	4d 3F	-	4p 3F	2	-	3
997.3431	Cu II	1	15.94	-	17.18	4p 3D	-	5d 1P	2	-	1
998.1386	Cu II	2	16.87	-	18.11	4f 3D	-	5g 3D	3	-	2
998.9362	Cu II	2	13.65	-	14.89	5s 3D	-	5p 3P	1	-	2
999.3965	Cu II	50	17.13	-	18.37	4f 1F	-	5g 3F	3	-	2
999.4037	Cu II	938	17.13	-	18.37	4f 1F	-	5g 1F	3	-	3
999.4774	Cu II	0	16.01	-	17.25	4p 3D	-	5d 1F	3	-	3
104.8880	Cu III	0		-			-			-	
118.6800	Cu III	0		-			-			-	
120.0960	Cu III	0		-			-			-	
121.9300	Cu III	0		-			-			-	
124.4380	Cu III	0		-			-			-	
127.9140	Cu III	0		-			-			-	
131.2390	Cu III	0		-			-			-	
133.2970	Cu III	0		-			-			-	
133.9480	Cu III	0		-			-			-	
136.3080	Cu III	0		-			-			-	
137.6790	Cu III	0		-			-			-	
137.7490	Cu III	0		-			-			-	
142.3480	Cu III	0		-			-			-	
148.1230	Cu III	0		-			-			-	
154.3460	Cu III	0		-			-			-	

159.3750	Cu III	0	-	-	-
164.2210	Cu III	0	-	-	-
167.9140	Cu III	0	-	-	-
170.2100	Cu III	0	-	-	-
172.2370	Cu III	0	-	-	-
174.1370	Cu III	0	-	-	-
176.8860	Cu III	0	-	-	-
184.0910	Cu III	0	-	-	-
197.1950	Cu III	0	-	-	-
201.3220	Cu III	0	-	-	-
215.7280	Cu III	0	-	-	-
229.9470	Cu III	0	-	-	-
236.8170	Cu III	0	-	-	-
239.1740	Cu III	0	-	-	-
240.5500	Cu III	0	-	-	-
241.2340	Cu III	0	-	-	-
244.4440	Cu III	0	-	-	-
246.8410	Cu III	0	-	-	-
248.2360	Cu III	0	-	-	-
248.6460	Cu III	0	-	-	-
250.8490	Cu III	0	-	-	-
252.2380	Cu III	0	-	-	-
253.8660	Cu III	0	-	-	-
256.6370	Cu III	0	-	-	-
257.3330	Cu III	0	-	-	-
260.9320	Cu III	0	-	-	-
264.3920	Cu III	0	-	-	-
269.6380	Cu III	0	-	-	-
275.1330	Cu III	0	-	-	-
281.2940	Cu III	0	-	-	-
297.8870	Cu III	0	-	-	-
354.8870	Cu III	0	-	-	-
363.9420	Cu III	0	-	-	-
370.2920	Cu III	0	-	-	-
374.4700	Cu III	0	-	-	-
374.8270	Cu III	0	-	-	-
375.2060	Cu III	0	-	-	-
377.6970	Cu III	0	-	-	-
379.0800	Cu III	0	-	-	-
380.4130	Cu III	0	-	-	-
380.9180	Cu III	0	-	-	-

388.1680	Cu III	0	-	-	-
395.3810	Cu III	0	-	-	-
409.0490	Cu III	0	-	-	-
428.3400	Cu III	0	-	-	-
435.1970	Cu III	0	-	-	-
435.2800	Cu III	0	-	-	-
435.5240	Cu III	0	-	-	-
437.0840	Cu III	0	-	-	-
437.1400	Cu III	0	-	-	-
437.3430	Cu III	0	-	-	-
437.7110	Cu III	0	-	-	-
438.6420	Cu III	0	-	-	-
492.7410	Cu III	0	-	-	-
509.4280	Cu III	0	-	-	-
516.8970	Cu III	0	-	-	-
520.8340	Cu III	0	-	-	-
521.9210	Cu III	0	-	-	-
526.8590	Cu III	0	-	-	-
531.7780	Cu III	0	-	-	-
536.9790	Cu III	0	-	-	-
541.8480	Cu III	0	-	-	-
549.4940	Cu III	0	-	-	-
557.3940	Cu III	0	-	-	-
560.9000	Cu III	0	-	-	-
570.2120	Cu III	0	-	-	-
576.8560	Cu III	0	-	-	-
585.0720	Cu III	0	-	-	-
596.5250	Cu III	0	-	-	-
610.0870	Cu III	0	-	-	-
636.9270	Cu III	0	-	-	-
651.2540	Cu III	0	-	-	-
664.4130	Cu III	0	-	-	-
679.3200	Cu III	0	-	-	-
105.6130	Cu IV	0	-	-	-
107.4720	Cu IV	0	-	-	-
109.1650	Cu IV	0	-	-	-
110.5500	Cu IV	0	-	-	-
111.9430	Cu IV	0	-	-	-
115.2180	Cu IV	0	-	-	-
122.7440	Cu IV	0	-	-	-
122.8870	Cu IV	0	-	-	-
125.8690	Cu IV	0	-	-	-

127.4840	Cu IV	0	-	-	-
129.3460	Cu IV	0	-	-	-
130.9410	Cu IV	0	-	-	-
132.1170	Cu IV	0	-	-	-
134.0080	Cu IV	0	-	-	-
135.0420	Cu IV	0	-	-	-
136.2050	Cu IV	0	-	-	-
137.2140	Cu IV	0	-	-	-
137.7820	Cu IV	0	-	-	-
138.8800	Cu IV	0	-	-	-
140.5490	Cu IV	0	-	-	-
141.5270	Cu IV	0	-	-	-
143.4340	Cu IV	0	-	-	-
144.9690	Cu IV	0	-	-	-
146.6180	Cu IV	0	-	-	-
148.2770	Cu IV	0	-	-	-
149.9810	Cu IV	0	-	-	-
151.5280	Cu IV	0	-	-	-
153.5120	Cu IV	0	-	-	-
155.1120	Cu IV	0	-	-	-
156.7350	Cu IV	0	-	-	-
158.3470	Cu IV	0	-	-	-
159.5120	Cu IV	0	-	-	-
160.8140	Cu IV	0	-	-	-
163.9750	Cu IV	0	-	-	-
165.0160	Cu IV	0	-	-	-
170.4370	Cu IV	0	-	-	-
179.7990	Cu IV	0	-	-	-
181.7560	Cu IV	0	-	-	-
181.9230	Cu IV	0	-	-	-
183.7040	Cu IV	0	-	-	-
184.9620	Cu IV	0	-	-	-
186.7240	Cu IV	0	-	-	-
191.8710	Cu IV	0	-	-	-
196.6310	Cu IV	0	-	-	-
109.7100	Cu V	0	-	-	-
110.6240	Cu V	0	-	-	-
111.3220	Cu V	0	-	-	-
112.1200	Cu V	0	-	-	-
112.8800	Cu V	0	-	-	-
113.3860	Cu V	0	-	-	-

114.2380	Cu V	0	-	-	-
114.9060	Cu V	0	-	-	-
115.7540	Cu V	0	-	-	-
116.7350	Cu V	0	-	-	-
117.6530	Cu V	0	-	-	-
118.3630	Cu V	0	-	-	-
119.2540	Cu V	0	-	-	-
120.1220	Cu V	0	-	-	-
120.4900	Cu V	0	-	-	-
121.4360	Cu V	0	-	-	-
122.1340	Cu V	0	-	-	-
123.0110	Cu V	0	-	-	-
123.9730	Cu V	0	-	-	-
124.6990	Cu V	0	-	-	-
125.3070	Cu V	0	-	-	-
126.0240	Cu V	0	-	-	-
126.9350	Cu V	0	-	-	-
127.8200	Cu V	0	-	-	-
128.6130	Cu V	0	-	-	-
129.2080	Cu V	0	-	-	-
129.9220	Cu V	0	-	-	-
130.9720	Cu V	0	-	-	-
131.8890	Cu V	0	-	-	-
132.3280	Cu V	0	-	-	-
132.9220	Cu V	0	-	-	-
